# A dataset of branched fatty acid esters of hydroxy fatty acids diversity in foods

**DOI:** 10.1038/s41597-023-02712-z

**Published:** 2023-11-10

**Authors:** Na An, Yu Wang, Dong-Xiao He, Peng-Cheng Mei, Quan-Fei Zhu, Yu-Qi Feng

**Affiliations:** 1 School of Bioengineering and Health, Wuhan Textile University, Wuhan, 430200 China; 2 Department of Chemistry, Wuhan University, Wuhan, 430072 China; 3 School of Public Health, Wuhan University, Wuhan, 430071 China; 4 Frontier Science Center for Immunology and Metabolism, Wuhan University, Wuhan, 430071 China

**Keywords:** Fatty acids, Small molecules

## Abstract

Branched fatty acid esters of hydroxy fatty acids (FAHFAs) are a class of bioactive lipids that show therapeutic potential for diabetes, anti-cancer and inflammation. These FAHFAs can be obtained through dietary intake, potentially improving human health. However, there is currently inadequate knowledge regarding the presence and variety of FAHFAs in different foods. Herein, we profile FAHFAs from 12 typical food samples and 4 medicinal food samples with the aid of our previous established chemical isotope labeling-assisted liquid chromatography-mass spectrometry method and build a comprehensive dataset of FAHFA diversity. The dataset comprised a total of 1207 regioisomers belonging to 298 different families, with over 100 families being newly discovered for the first time. Therefore, our findings contribute valuable insights into the molecular diversity and presence of FAHFA in a range of foods. This dataset serves as a foundation for further exploration of the nutritional and medicinal functions of FAHFAs.

## Background & Summary

Branched fatty acid esters of hydroxy fatty acids (FAHFAs) are a recently discovered class of natural lipids with important biological activities^1–3^. Research has shown that FAHFAs possess anti-diabetes^4–8^, anti-cancer^9,10^, anti-inflammatory^6,11^ activities, cardiovascular protective activities^12^, and hepato-protective activities^13,14^ in mammals. FAHFA bioactivities are closely related to human health with therapeutic potential for diabetes, anti-cancer and inflammatory diseases.

FAHFAs are widely present in nature from yeast to mammals^15–20^. A variety of foods such as tea^21^, breast milk^22^, olive oil^23^, and venison^24^ are all natural sources of FAHFAs. Furthermore, emerging evidence suggests that human could obtain FAHFAs through dietary intake^1,25^, thus changing the composition and levels of FAHFAs in the body, further improving health. Therefore, obtaining the FAHFA profile in different foods is of great significance for the food nutrition, and dietary guidance. Although some studies on FAHFA in foods have been performed in recent years^11,16,22–24,26,27^, most of these studies only focus on the content of a few known FAHFA molecules in foods. There is still a lack of the comprehensive exploration of diversity and composition of FAHFAs in foods.

To explore the diversity characteristics of the FAHFA and build a comprehensive global dataset, we screened and annotated FAHFAs from 12 typical food samples (3 algae, 1 fungus, 6 plant foods, and 2 animal foods) and 4 medicinal foods with anti-type 2 diabetes or anti-inflammatory function respectively, with the aid of chemical isotope labeling-assisted liquid chromatography-mass spectrometry (CIL-LC-MS) technique^28^. A total of 1207 FAHFA regioisomers belonging to 298 families were detected from these foods, of which more than 100 FAHFA families were discovered for the first time. Our data sheds light on the molecular diversity of FAHFAs and provides the useful information for further research on the effect of FAHFAs on nutritional and medicinal values of foods.

## Methods

### Study design

**Fig. 1:**
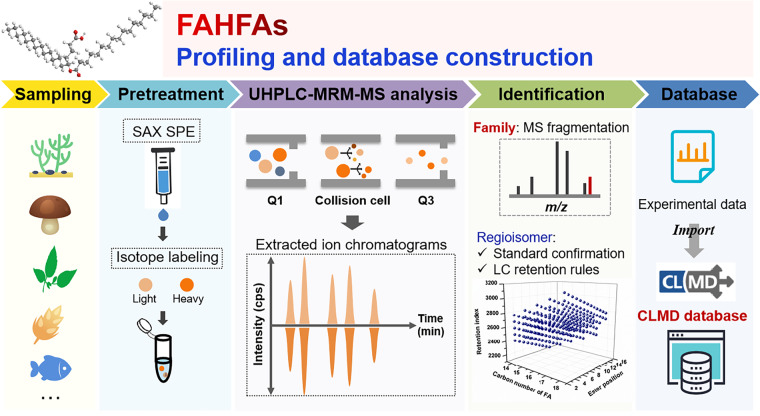
Overview of FAHFA profiling procedures from various food samples by CIL-LC-MRM MS method.

We employed the CIL-LC-MS approach to explore the FAHFA diversity from various types of foods. The overview procedures for FAHFA profiling in foods was shown in Fig. 1. Briefly, the Bligh and Dyer lipid extraction in combination with SAX-SPE (strong anion-exchange solid-phase extraction) was performed to extract and purify FAHFAs from the food samples, followed by the DMED (2-dimethylaminoethylamine) and *d*_4_-DMED labeling, respectively. Subsequently, the equal volume of DMED derivatives and *d*_4_-DMED derivatives were mixed and analyzed by LC-MS approach. The raw data acquired by LC-MS were extracted to screen FAHFA candidates according to the thresholds of peak pairs with identical retention time (RT) and similar peak height from the corresponding DMED- and *d*_4_-DMED- labeled channels. Finally, the screened FAHFA candidates were annotated by FAHFA standard verification and predicted retention index (RI) matching.

### Chemicals and regents

FAHFA standards were obtained from RC ChemTec Co., Ltd. (Wuhan, Hubei, China), Cayman Chemical (Ann Arbor, MI, USA), and XuKang Medical Science and Technology Co., Ltd. (Xiangtan, Hunan, China). Detailed information of FAHFA standards was listed in Table S1 in Supplementary information-1 and the abbreviation as well as normal name of FAHFA was presented in Table S2 in Supplementary information-2. Analytical-grade reagents acetonitrile (ACN), triethylamine (TEA), methanol (MeOH), 2-chloro-1-methylpyridinium iodide (CMPI), acetone, DMED, ammonium hydroxide, and formic acid were supplied by Sinopharm Chemical Reagent Co., Ltd. (Shanghai, China). *d*_4_-DMED and SAX SPE (3 mL, 200 mg) were purchased from Weltech Technology Co., Ltd (Wuhan, China). HPLC-grade reagents ACN, isopropanol (IPA), chloroform (CHCl_3_), MeOH, and acetone were obtained from Merck (Darmstadt, Germany).

### Sample collection and pretreatment

*Spirulina* and *Nostoc commune Vauch* were obtained from the Freshwater Algae Culture Collection at the Institute of Hydrobiology (Wuhan, China). Peanut, rice grains, wheat grains, and black sesame were provided by the Hubei Academy of Agricultural Sciences (Wuhan, China). Medicinal foods, including lotus plumule, Chinese yam, *Lycium chinense*, and *Coptis chinensis*, were purchased from Tongrentang Medicine Cooperation (China). Other food samples, *Lentinus edodes*, tomato, apple, kelp, egg, and fish were purchased from a local Walmart (Wuhan, China). The analysis of each species involved the pooling of a minimum of six samples into one composite sample.

**Fig. 2:**
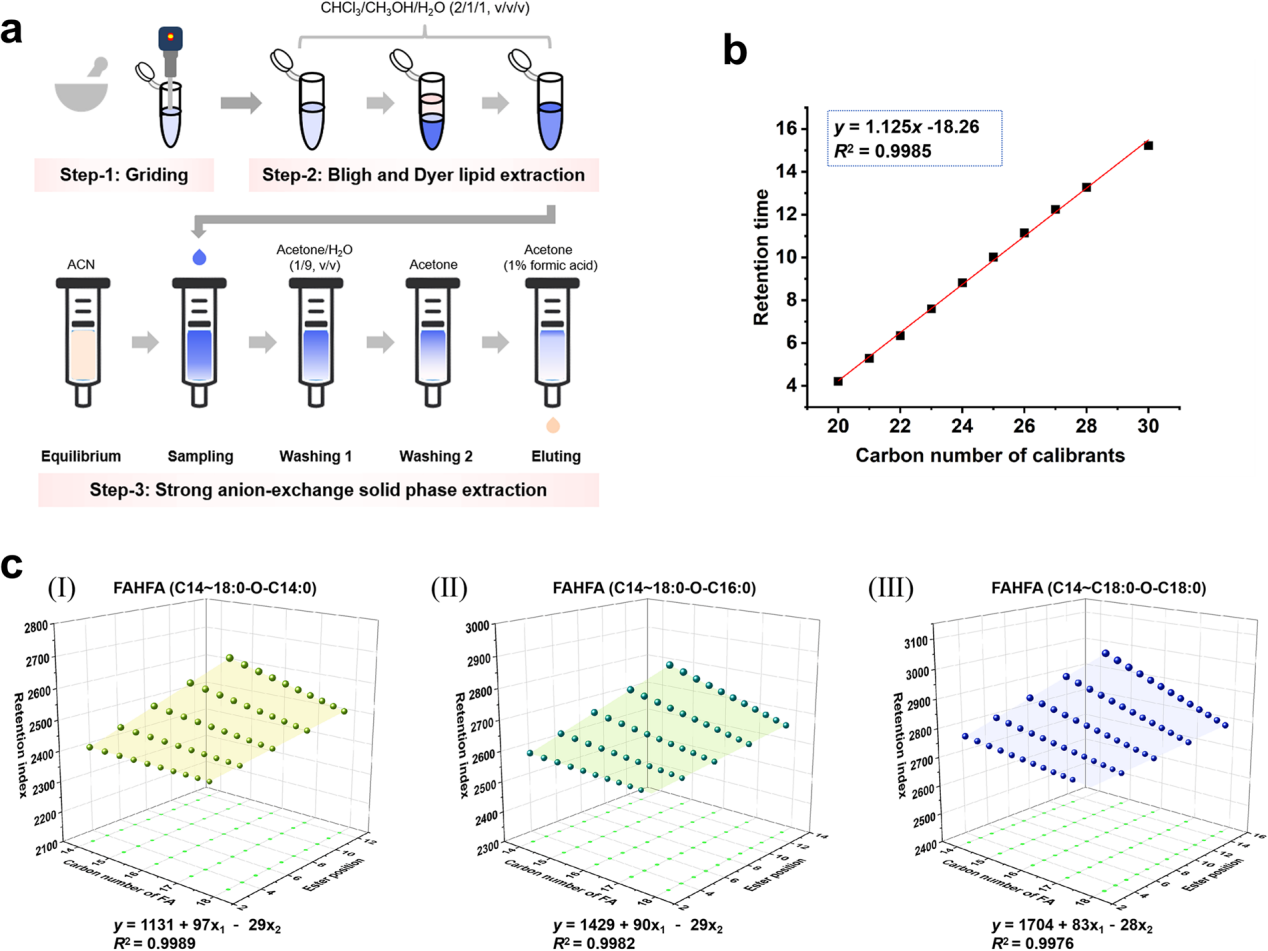
Schematic workflow of FAHFA extraction and identification. (**a**) Step-to-step workflow of griding, Bligh and Dyer lipid extraction and strong anion-exchange solid phase extraction to extract and purify FAHFAs; (**b**) Regressive curve of retention times of DMED-labeled normal saturated fatty acid standards with carbon chain length from C20 to C30 (calibrants) on C18 column vs the carbon number of fatty acids; (**c**) Prediction models of FAHFA (C14~18:0-O-C14:0), FAHFA (C14~18:0-O-C16:0), and FAHFA (C14~18:0-O-C18:0), based on the retention index, ester position, and carbon number of fatty acids. *y* denotes the retention index of FAHFA, x_1_ denotes the carbon number of FA, x_2_ denotes the ester position.

The pretreatment of food samples included FAHFA extraction, FAHFA purification, and chemical isotope labeling. FAHFA extraction and purification were performed by Bligh-Dyer lipid extraction^29^ and solid phase extraction, respectively (Fig. 2a), and DMED/*d*_4_-DMED reagent was used to label the FAHFA extracts^28^.

### LC-MS analysis

Analysis of FAHFA was performed on a UHPLC-QqQ-MS system consisting of a Shimadzu LC-30AD UHPLC system (Tokyo, Japan) with two 30AD pumps, a CTO-30A column oven, a SIL-30AC auto sampler as well as a DGU-30A5R degasser, and a Shimadzu MS-8045 mass spectrometer (Tokyo, Japan) equipped with an electrospray ionization (ESI) source (Turbo Ionspray). LC separation was conducted on an Acquity UPLC BEH C18 column (2.1 mm × 50 mm, 1.7 μm, Waters) with a flow rate of 0.4 mL/min at 40 °C. Phase A (formic acid in ACN/H_2_O, 0.1%, at the ratio of 6/4, v/v) and phase B (formic acid in IPA/ACN, 0.1%, at the ratio of 9/1, v/v) were employed as mobile phases. DMED-labeled FAHFA regioisomer separation was performed at a gradient of 0–26 min, 25%–90% B; 26–30 min, 90% B; and 30–31 min, 90%–25% B. FAHFA analysis was performed in multiple reaction monitoring (MRM) mode under positive ionization.

We simulated 529 FAHFA family structures (Table S2, NO 47–575) by permuting and combining 23 essential long-chain FAs (Table S2, NO 1–23) and 23 long-chain HFAs (Table S2, NO 24–46). Subsequently, a MRM transition list including 1058 MRM ion channels was constructed according to the fragmentation pattern of DMED/*d*_4_-DMED-labeled FAHFAs ([DMED-FAHFA]^+^  → [DMED-HFA−63]^+^ and [*d*_4_-DMED-FAHFA]^+^  → [*d*_4_-DMED-FA−67]^+^)^10,28^. These 1058 MRM ion channels were evenly assigned to 20 LC-MS acquisition methods to ensure the sensitivity for MRM MS analysis, and the detailed MRM parameters were presented in Table S3 (Supplementary information-3). Raw data acquisition and processing were conducted through LabSolutions software (version 5.53 sp2, Shimadzu, Tokyo, Japan).

### FAHFA retention index

LC-MS-based FAHFA analysis often suffers from RT shift due to slight change in the three-phase FAHFA separation system, thus resulting in misassignment of FAHFA regioisomers. To minimize the effect of RT shift and improve the reliability of the assignment of FAHFA peaks, the chemical labeling-based RPLC RI^30,31^ was introduced with DMED-labeled C20~C30 normal fatty acids used as calibrators. A linear regression approach was used to calibrate the RT drifts of FAHFA regioisomers on C18 column with calibrants. Based on the observation that DMED-labeled C20~C30 normal fatty acids exhibited a linear increase in RTs on C18 column with increasing carbon number (Fig. 2b), a linear regression equation (Eq. 1) was derived to analyze the relationship between carbon number of calibrants and RT. Subsequently, Eq. 2 was used to determine the RI of a given FAHFA regioisomer based on its measured RT.

1 $$y=kx+b$$

2 $$RI=100\times \left(RT-b\right)/k$$

where *y* denotes the RT of calibrants; *x* denotes the carbon number of calibrants; RT denotes the RT of the given FAHFA regioisomer; *k* and *b* are constants.

The application of the FAHFA RI provides a comparable chromatographic retention value for the FAHFA annotation of various samples.

### Construction of RI cycle-prediction model

Our previous studies have demonstrated that the retention of FAHFAs on the C18 column both follows the ester position rule and carbon number rule^32^. In order to expand the coverage of retention rule-assisted FAHFA annotation, three prediction models were constructed by using the cycle-prediction approach, including FAHFA (C14~18:0-O-C14:0), FAHFA (C14~18:0-O-C16:0), and FAHFA (C14~18:0-O-C18:0). As depicted in Fig. 2c, the three prediction models exhibit good correlation. The binary linear regression equations for FAHFA (C14~18:0-O-C14:0), FAHFA (C14~18:0-O-C16:0), and FAHFA (C14~18:0-O-C18:0) are *y* = 1131 + 97*x*_1_ - 29*x*_2_ with *R*^*2*^ = 0.9989, *y* = 1429 + 90*x*_1_ - 29*x*_2_ with *R*^*2*^ = 0.9982, and *y* = 1704 + 83*x*_1_ - 28*x*_2_ with *R*^2^ = 0.9976 respectively. Consequently, the RI of 167 FAHFA regioisomers from 15 families were obtained. Based on the predicted FAHFA RI values, FAHFA regioisomers detected from food samples could be annotated via RI matching.

### Profiling of FAHFAs in foods

**Fig. 3:**
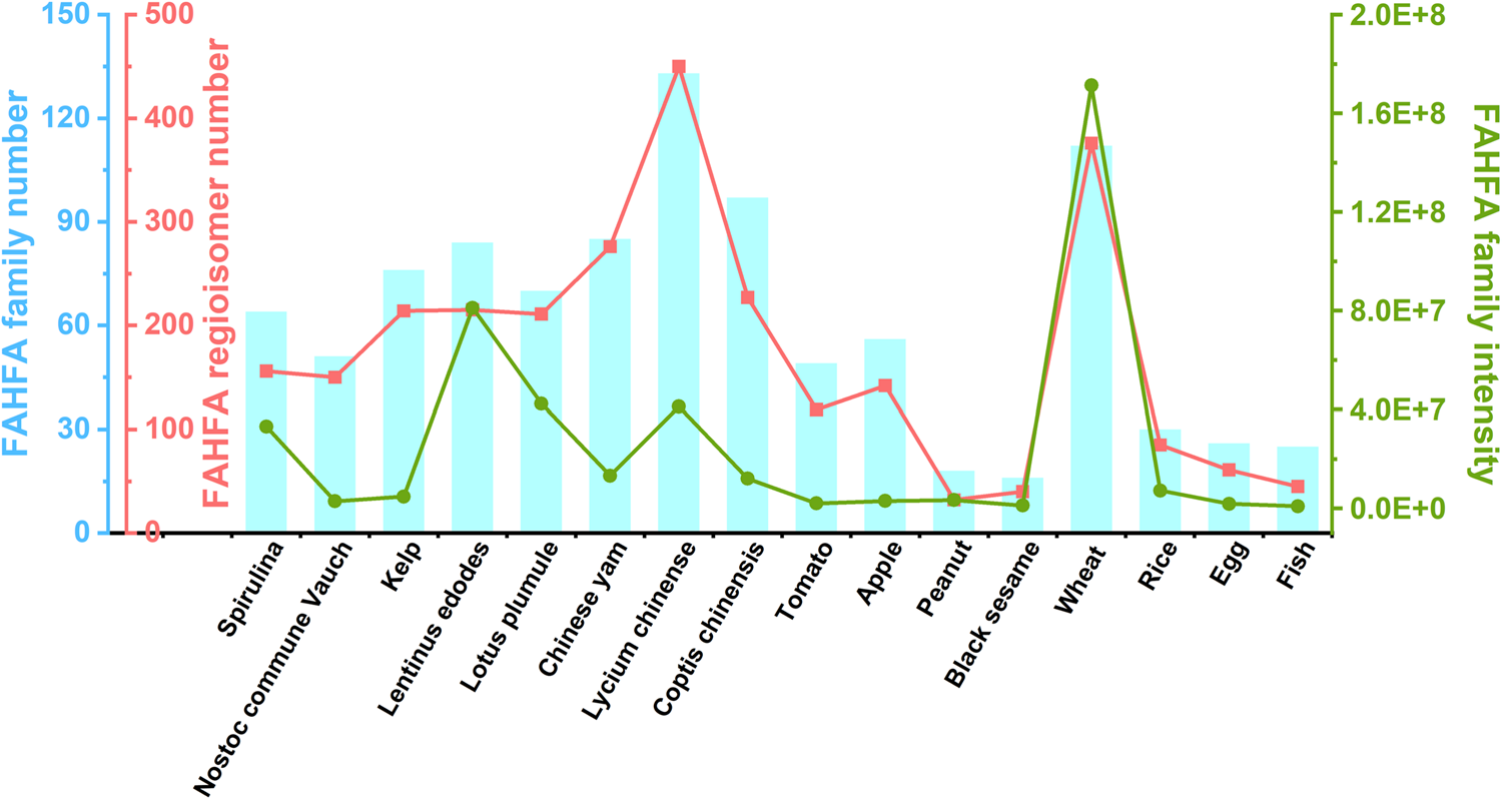
Distribution of FAHFA families and regioisomers in the 12 foods and 4 medicinal foods. Cyan histograms represent the number of FAHFA families; red line represents the number of FAHFA regioisomers; green line represents the total MS intensity of FAHFAs.

We profiled the FAHFAs from 12 food samples, including 3 algae (kelp, *Spirulina*, and *Nostoc commune Vauch*), 1 fungus (*Lentinus edodes*), 6 plant foods (tomato, apple, peanut, black sesame, wheat grains, and rice grains), and 2 animal foods (egg and fish). Additionally, 4 medicinal food samples (lotus plumule, Chinese yam, *Lycium chinense*, and *Coptis chinensis*) were also selected for FAHFA profiling because they have similar properties (anti-type 2 diabetes or anti-inflammatory) to FAHFA and are commonly consumed in daily life^33–36^. By using the CIL-LC-MRM MS method, a total of 1207 regioisomers belonging to 298 FAHFA families were detected from the above 12 food samples and 4 medicinal food samples (Table S4). Among them, 156 regioisomers (64 FAHFA families) were detected from *Spirulina*, 150 regioisomers (51 FAHFA families) from *Nostoc commune Vauch*, 214 regioisomers (76 FAHFA families) from kelp, 215 regioisomers (84 FAHFA families) from *Lentinus edodes*, 211 regioisomers (70 FAHFA families) from lotus plumule, 276 regioisomers (85 FAHFA families) from Chinese yam, 450 regioisomers (133 FAHFA families) from *Lycium chinense*, 227 regioisomers (97 FAHFA families) from *Coptis chinensis*,119 regioisomers (49 FAHFA families) from tomato, 142 regioisomers (56 FAHFA families) from apple, 32 regioisomers (18 FAHFA families) from peanut, 40 regioisomers (16 FAHFA families) from black sesame, 376 regioisomers (112 FAHFA families) from wheat grains, 85 regioisomers (30 FAHFA families) from rice grains, 61 regioisomers (26 FAHFA families) from egg, and 45 regioisomers (25 FAHFA families) from fish (*Carassius auratus*). The detailed information of these detected FAHFAs including their distribution and MS intensity readouts was presented in Fig. 3, Table S4 in Supplementary information-4, Table S5 in Supplementary information-5, Table S6 in Supplementary information-6, and Table S7 in Supplementary information-7. Subsequently, the detected FAHFA regioisomers were annotated by matching measured RI values with the predicted RI values. As a result, 132 FAHFA regioisomers were identified (Table S8 in Supplementary information-8).

## Data Records

The FAHFA data were acquired from 12 food samples and 4 medicinal food samples by CIL-LC-MRM MS method. The data of FAHFA standard used, hypothetical MRM ion transitions employed for FAHFA screening, as well as the FAHFA diversity in these foods are accessible on figshare in .xlsx format^37^.

Table S1 presents the FAHFA standards information, including the comprehensive details of FAHFA family, molecular formula, monoisotopic mass, SMILES number, InChIKey number and the structure of FAHFA.

Table S2 lists the abbreviations of fatty acids, hydroxy fatty acids and fatty acid esters of hydroxy fatty acids. It encompasses a total of 23 essential fatty acids (listed as NO 1–23), 23 hydroxy fatty acids (NO 24–46), and 529 FAHFA families (NO 47–575). Each entry in the table provides the corresponding full name, molecular formula, and the abbreviation.

Table S3 details the MRM transitions of the predicted FAHFAs in positive ion mode, which comprises a total of 1058 events. Each FAHFA event includes the *m/z* values of the precursor ion and product ion, ionization mode, collision energy and associated information on the predicted FAHFA structure.

Table S4 summarizes the distribution of FAHFAs (family and regioisomer) detected in 12 food samples (kelp, *Spirulina*, *Nostoc commune Vauch*, *Lentinus edodes*, tomato, apple, peanut, black sesame, wheat grains, and rice grains, egg, and fish) and 4 medicinal food samples (lotus plumule, Chinese yam, *Lycium chinense*, and *Coptis chinensis*).

Table S5 gives the detected FAHFA lists in 12 food samples and 4 medicinal food samples in detail. This table included 16 sheets, and each sheet offers comprehensive information about the detected FAHFA families from one specific food sample, including normal name, FAHFA ID, *m/z* of the DMED labeling and *d*_4_-DMED labeling, and the retention index for each regioisomer.

Table S6 summarizes the MS intensity of FAHFA families detected in 12 food samples (kelp, *Spirulina*, *Nostoc commune Vauch*, *Lentinus edodes*, tomato, apple, peanut, black sesame, wheat grains, and rice grains, egg, and fish) and 4 medicinal food samples (lotus plumule, Chinese yam, *Lycium chinense*, and *Coptis chinensis*).

Table S7 provides the abundance of the detected FAHFA regioisomers in 12 food samples and 4 medicinal food samples in detail. This table included 16 sheets, and each sheet offers comprehensive information about the detected FAHFA families from one specific food sample, including normal name, FAHFA ID, *m/z* of the DMED labeling and *d*_4_-DMED labeling, and the MS intensity for each regioisomer.

Table S8 presents the FAHFA name, MRM transition for both DMED labeling and *d*_4_-DMED labeling, retention index, monoisotopic mass (unlabeled), molecular formula, SMILES number and InChIKey number for each identified FAHFA regioisomer.

## Technical Validation

In previous studies, the CIL-LC-MS technique has been thoroughly applied to screen FAHFA molecules in both plant and animal samples^10,28^, ensuring its validity and accuracy. The prediction model employed for FAHFA regioisomer annotation based on the ester position rule and carbon number rule had also been validated elsewhere^27^.

### Supplementary information

Supplementary information-2 Table S2. Abbreviations of FAHFAs

Supplementary information-3 Table S3. MRM transitions list

Supplementary information-4 Table S4. Distribution of FAHFAs

Supplementary information-5 Table S5. Detected FAHFA lists

Supplementary information-6 Table S6. MS intensity of FAHFA familes

Supplementary information-7 Table S7. MS intensity of FAHFA regioisomers

Supplementary information-8 Table S8. Identified FAHFA regioisomers list

Supplementary information-1 Table S1. FAHFA standards list

## Data Availability

No custom code was made during the collection and validation of this dataset.
