# Supplementary material for: A dataset of branched fatty acid esters of hydroxy fatty acids diversity in foods
Source: Sci Data. 2023 Nov 10;10:790. doi: 10.1038/s41597-023-02712-z (PMC10638281; doi:10.1038/s41597-023-02712-z)
Supplement: Supplementary file 1 — Supplementary information-2 Table S2. Abbreviations of FAHFAs [file 41597_2023_2712_MOESM1_ESM.pdf]

Supplementary Table S2. Abbreviations of fatty acids, hydroxy fatty acids and fatty acid esters of hydroxy fatty acids

Essential fatty acids

Number	Abbreviation	Full name	Formula
1	MA	Myristic acid	C14H28O2
2	MO	Myristoleic acid	C14H26O2
3	PDA	Pentadecanoic acid	C15H30O2
4	PDEA	Pentadecenoic acid	C15H28O2
5	PO	Palmitoleic acid	C16H30O2
6	PA	Palmitic acid	C16H32O2
7	HDA	Heptadecanoic acid	C17H34O2
8	HDEA	Heptadecenoic acid	C17H32O2
9	SA	Stearic acid	C18H36O2
10	OA	Oleic acid	C18H34O2
11	LA	Linoleic acid	C18H32O2
12	ALA	α-Linolenic acid	C18H30O2
13	SDA	Stearidonic acid	C18H28O2
14	AA	Arachidic acid	C20H40O2
15	EA	Eicosenoic acid	C20H38O2
16	EDA	Eicosadienoic acid	C20H36O2
17	ETA	Eicosatrienoic acid	C20H34O2
18	ARA	Arachidonic acid	C20H32O2
19	EPA	Eicosapentaenoic acid	C20H30O2
20	BA	Behenic acid	C22H44O2
21	DEA	Docosenoic acid	C22H42O2
22	DDA	Docosadienoate	C22H40O2
23	DHA	Docosahexaenoic acid	C22H32O2

Hydroxy fatty acid

Number	Abbreviation	Full name	Formula
24	HMO	Hydroxy myristoleic acid	C14H26O3
25	HTDA	Hydroxy tetradecanoic acid	C14H24O2
26	HTTA	Hydroxy tetradecatrienoic acid	C14H22O2
27	HPO	Hydroxy palmitoleic acid	C16H30O3
28	HHDDA	Hydroxy hexadecadienoic acid	C16H28O3
29	HHTA	Hydroxy hexadecatrienoic acid	C16H26O3
30	HOA	Hydroxy oleic acid	C18H34O3
31	HLA	Hydroxy linoleic acid	C18H32O3
32	HALA	Hydroxy α-Linolenic acid	C18H30O3
33	HEA	Hydroxy eicosenoic acid	C20H38O3
34	HEDA	Hydroxy eicosadienoic acid	C20H36O3
35	HETA	Hydroxy eicosatrienoic acid	C20H34O3
36	HDHA	Hydroxy docosahexaenoic acid	C22H32O3
37	HARA	Hydroxy arachidonic acid	C20H32O3
38	HDA	Hydroxy dodecanoic acid	C12H24O3
39	HMA	Hydroxy myristic acid	C14H28O3
40	HPDA	Hydroxy pentadecanoic acid	C15H30O3
41	HPA	Hydroxy palmitic acid	C16H32O3
42	HHDA	Hydroxy heptadecanoic acid	C17H34O3
43	HSA	Hydroxy stearic acid	C18H36O3
44	HNDA	Hydroxy nonadecanoic acid	C19H38O3
45	HAA	Hydroxy arachidic acid	C20H40O3
46	HHEA	Hydroxy heneicosanoic acid	C21H42O3

Fatty acid esters of hydroxy fatty acid (FAHFA)

Number	Abbreviation	Full name	Normal name	Formula
47	MAHMO	Myristic acid-hydroxy myristoleic acid	FAHFA(14:0-O-14:1)	C28H52O4
48	MOHMO	Myristoleic acid-hydroxy myristoleic acid	FAHFA(14:1-O-14:1)	C28H50O4
49	PDAHMO	Pentadecanoic acid-hydroxy myristoleic acid	FAHFA(15:0-O-14:1)	C29H54O4
50	PDEAHMO	Pentadecenoic acid-hydroxy myristoleic acid	FAHFA(15:1-O-14:1)	C29H52O4
51	POHMO	Palmitoleic acid-hydroxy myristoleic acid	FAHFA(16:1-O-14:1)	C30H54O4
52	PAHMO	Palmitic acid-hydroxy myristoleic acid	FAHFA(16:0-O-14:1)	C30H56O4
53	HDAHMO	Heptadecanoic acid-hydroxy myristoleic acid	FAHFA(17:0-O-14:1)	C31H58O4
54	HDEAHMO	Heptadecenoic acid-hydroxy myristoleic acid	FAHFA(17:1-O-14:1)	C31H56O4
55	SAHMO	Stearic acid-hydroxy myristoleic acid	FAHFA(18:0-O-14:1)	C32H60O4
56	OAHMO	Oleic acid-hydroxy myristoleic acid	FAHFA(18:1-O-14:1)	C32H58O4
57	LAHMO	Linoleic acid-hydroxy myristoleic acid	FAHFA(18:2-O-14:1)	C32H56O4
58	ALAHMO	α-Linolenic acid-hydroxy myristoleic acid	FAHFA(18:3-O-14:1)	C32H54O4
59	SDAHMO	Stearidonic acid-hydroxy myristoleic acid	FAHFA(18:4-O-14:1)	C32H52O4
60	AAHMO	Arachidic acid-hydroxy myristoleic acid	FAHFA(20:0-O-14:1)	C34H64O4
61	EAHMO	Eicosenoic acid-hydroxy myristoleic acid	FAHFA(20:1-O-14:1)	C34H62O4
62	EDAHMO	Eicosadienoic acid-hydroxy myristoleic acid	FAHFA(20:2-O-14:1)	C34H60O4
63	ETAHMO	Eicosatrienoic acid-hydroxy myristoleic acid	FAHFA(20:3-O-14:1)	C34H58O4
64	ARAHMO	Arachidonic acid-hydroxy myristoleic acid	FAHFA(20:4-O-14:1)	C34H56O4
65	EPAHMO	Eicosapentaenoic acid-hydroxy myristoleic acid	FAHFA(20:5-O-14:1)	C34H54O4
66	BAHMO	Behenic acid-hydroxy myristoleic acid	FAHFA(22:0-O-14:1)	C36H68O4

67	DEAHMO	Docosenoic acid-hydroxy myristoleic acid	FAHFA(22:1-O-14:1)	C36H66O4
68	DDAHMO	Docosadienoate-hydroxy myristoleic acid	FAHFA(22:2-O-14:1)	C36H64O4
69	DHAHMO	Docosahexaenoic acid-hydroxy myristoleic acid	FAHFA(22:6-O-14:1)	C36H56O4
70	MAHTDA	Myristic acid-hydroxy tetradecadienoic acid	FAHFA(14:0-O-14:2)	C28H50O4
71	MOHTDA	Myristoleic acid-hydroxy tetradecadienoic acid	FAHFA(14:1-O-14:2)	C28H48O4
72	PDAHTDA	Pentadecanoic acid-hydroxy tetradecadienoic acid	FAHFA(15:0-O-14:2)	C29H52O4
73	PDEAHTDA	Pentadecenoic acid-hydroxy tetradecadienoic acid	FAHFA(15:1-O-14:2)	C29H50O4
74	POHTDA	Palmitoleic acid-hydroxy tetradecadienoic acid	FAHFA(16:1-O-14:2)	C30H52O4
75	PAHTDA	Palmitic acid-hydroxy tetradecadienoic acid	FAHFA(16:0-O-14:2)	C30H54O4
76	HDAHTDA	Heptadecanoic acid-hydroxy tetradecadienoic acid	FAHFA(17:0-O-14:2)	C31H56O4
77	HDEAHTDA	Heptadecenoic acid-hydroxy tetradecadienoic acid	FAHFA(17:1-O-14:2)	C31H54O4
78	SAHTDA	Stearic acid-hydroxy tetradecadienoic acid	FAHFA(18:0-O-14:2)	C32H58O4
79	OAHTDA	Oleic acid-hydroxy tetradecadienoic acid	FAHFA(18:1-O-14:2)	C32H56O4
80	LAHTDA	Linoleic acid-hydroxy tetradecadienoic acid	FAHFA(18:2-O-14:2)	C32H54O4
81	ALAHTDA	α-Linolenic acid-hydroxy tetradecadienoic acid	FAHFA(18:3-O-14:2)	C32H52O4
82	SDAHTDA	Stearidonic acid-hydroxy tetradecadienoic acid	FAHFA(18:4-O-14:2)	C32H50O4
83	AAHTDA	Arachidic acid-hydroxy tetradecadienoic acid	FAHFA(20:0-O-14:2)	C34H62O4
84	EAHTDA	Eicosenoic acid-hydroxy tetradecadienoic acid	FAHFA(20:1-O-14:2)	C34H60O4
85	EDAHTDA	Eicosadienoic acid-hydroxy tetradecadienoic acid	FAHFA(20:2-O-14:2)	C34H58O4
86	ETAHTDA	Eicosatrienoic acid-hydroxy tetradecadienoic acid	FAHFA(20:3-O-14:2)	C34H56O4
87	ARAHTDA	Arachidonic acid-hydroxy tetradecadienoic acid	FAHFA(20:4-O-14:2)	C34H54O4
88	EPAHTDA	Eicosapentaenoic acid-hydroxy tetradecadienoic acid	FAHFA(20:5-O-14:2)	C34H52O4
89	BAHTDA	Behenic acid-hydroxy tetradecadienoic acid	FAHFA(22:0-O-14:2)	C36H66O4
90	DEAHTDA	Docosenoic acid-hydroxy tetradecadienoic acid	FAHFA(22:1-O-14:2)	C36H64O4
91	DDAHTDA	Docosadienoate-hydroxy tetradecadienoic acid	FAHFA(22:2-O-14:2)	C36H62O4
92	DHAHTDA	Docosahexaenoic acid-hydroxy tetradecadienoic acid	FAHFA(22:6-O-14:2)	C36H54O4
93	MAHTTA	Myristic acid-hydroxy tetradecatrienoic acid	FAHFA(14:0-O-14:3)	C28H48O4
94	MOHTTA	Myristoleic acid-hydroxy tetradecatrienoic acid	FAHFA(14:1-O-14:3)	C28H46O4
95	PDAHTTA	Pentadecanoic acid-hydroxy tetradecatrienoic acid	FAHFA(15:0-O-14:3)	C29H50O4
96	PDEAHTTA	Pentadecenoic acid-hydroxy tetradecatrienoic acid	FAHFA(15:1-O-14:3)	C29H48O4
97	POHTTA	Palmitoleic acid-hydroxy tetradecatrienoic acid	FAHFA(16:1-O-14:3)	C30H50O4
98	PAHTTA	Palmitic acid-hydroxy tetradecatrienoic acid	FAHFA(16:0-O-14:3)	C30H52O4
99	HDAHTTA	Heptadecanoic acid-hydroxy tetradecatrienoic acid	FAHFA(17:0-O-14:3)	C31H54O4
100	HDEAHTTA	Heptadecenoic acid-hydroxy tetradecatrienoic acid	FAHFA(17:1-O-14:3)	C31H52O4
101	SAHTTA	Stearic acid-hydroxy tetradecatrienoic acid	FAHFA(18:0-O-14:3)	C32H56O4
102	OAHTTA	Oleic acid-hydroxy tetradecatrienoic acid	FAHFA(18:1-O-14:3)	C32H54O4
103	LAHTTA	Linoleic acid-hydroxy tetradecatrienoic acid	FAHFA(18:2-O-14:3)	C32H52O4
104	ALAHTTA	α-Linolenic acid-hydroxy tetradecatrienoic acid	FAHFA(18:3-O-14:3)	C32H50O4
105	SDAHTTA	Stearidonic acid-hydroxy tetradecatrienoic acid	FAHFA(18:4-O-14:3)	C32H48O4
106	AAHTTA	Arachidic acid-hydroxy tetradecatrienoic acid	FAHFA(20:0-O-14:3)	C34H60O4
107	EAHTTA	Eicosenoic acid-hydroxy tetradecatrienoic acid	FAHFA(20:1-O-14:3)	C34H58O4
108	EDAHTTA	Eicosadienoic acid-hydroxy tetradecatrienoic acid	FAHFA(20:2-O-14:3)	C34H56O4
109	ETAHTTA	Eicosatrienoic acid-hydroxy tetradecatrienoic acid	FAHFA(20:3-O-14:3)	C34H54O4
110	ARAHTTA	Arachidonic acid-hydroxy tetradecatrienoic acid	FAHFA(20:4-O-14:3)	C34H52O4
111	EPAHTTA	Eicosapentaenoic acid-hydroxy tetradecatrienoic acid	FAHFA(20:5-O-14:3)	C34H50O4
112	BAHTTA	Behenic acid-hydroxy tetradecatrienoic acid	FAHFA(22:0-O-14:3)	C36H64O4
113	DEAHTTA	Docosenoic acid-hydroxy tetradecatrienoic acid	FAHFA(22:1-O-14:3)	C36H62O4
114	DDAHTTA	Docosadienoate-hydroxy tetradecatrienoic acid	FAHFA(22:2-O-14:3)	C36H60O4
115	DHAHTTA	Docosahexaenoic acid-hydroxy tetradecatrienoic acid	FAHFA(22:6-O-14:3)	C36H52O4
116	MAHPO	Myristic acid-hydroxy palmitoleic acid	FAHFA(14:0-O-16:1)	C30H56O4
117	MOHPO	Myristoleic acid-hydroxy palmitoleic acid	FAHFA(14:1-O-16:1)	C30H54O4
118	PDAHPO	Pentadecanoic acid-hydroxy palmitoleic acid	FAHFA(15:0-O-16:1)	C31H58O4
119	PDEAHPO	Pentadecenoic acid-hydroxy palmitoleic acid	FAHFA(15:1-O-16:1)	C31H56O4
120	POHPO	Palmitoleic acid-hydroxy palmitoleic acid	FAHFA(16:1-O-16:1)	C32H58O4
121	PAHPO	Palmitic acid-hydroxy palmitoleic acid	FAHFA(16:0-O-16:1)	C32H60O4
122	HDAHPO	Heptadecanoic acid-hydroxy palmitoleic acid	FAHFA(17:0-O-16:1)	C33H62O4
123	HDEAHPO	Heptadecenoic acid-hydroxy palmitoleic acid	FAHFA(17:1-O-16:1)	C33H60O4
124	SAHPO	Stearic acid-hydroxy palmitoleic acid	FAHFA(18:0-O-16:1)	C34H64O4
125	OAHPO	Oleic acid-hydroxy palmitoleic acid	FAHFA(18:1-O-16:1)	C34H62O4
126	LAHPO	Linoleic acid-hydroxy palmitoleic acid	FAHFA(18:2-O-16:1)	C34H60O4
127	ALAHPO	α-Linolenic acid-hydroxy palmitoleic acid	FAHFA(18:3-O-16:1)	C34H58O4
128	SDAHPO	Stearidonic acid-hydroxy palmitoleic acid	FAHFA(18:4-O-16:1)	C34H56O4
129	AAHPO	Arachidic acid-hydroxy palmitoleic acid	FAHFA(20:0-O-16:1)	C36H68O4
130	EAHPO	Eicosenoic acid-hydroxy palmitoleic acid	FAHFA(20:1-O-16:1)	C36H66O4
131	EDAHPO	Eicosadienoic acid-hydroxy palmitoleic acid	FAHFA(20:2-O-16:1)	C36H64O4
132	ETAHPO	Eicosatrienoic acid-hydroxy palmitoleic acid	FAHFA(20:3-O-16:1)	C36H62O4
133	ARAHPO	Arachidonic acid-hydroxy palmitoleic acid	FAHFA(20:4-O-16:1)	C36H60O4
134	EPAHPO	Eicosapentaenoic acid-hydroxy palmitoleic acid	FAHFA(20:5-O-16:1)	C36H58O4
135	BAHPO	Behenic acid-hydroxy palmitoleic acid	FAHFA(22:0-O-16:1)	C38H72O4
136	DEAHPO	Docosenoic acid-hydroxy palmitoleic acid	FAHFA(22:1-O-16:1)	C38H70O4
137	DDAHPO	Docosadienoate-hydroxy palmitoleic acid	FAHFA(22:2-O-16:1)	C38H68O4
138	DHAHPO	Docosahexaenoic acid-hydroxy palmitoleic acid	FAHFA(22:6-O-16:1)	C38H60O4
139	MAHHDDA	Myristic acid-hydroxy hexadecadienoic acid	FAHFA(14:0-O-16:2)	C30H54O4
140	MOHHDDA	Myristoleic acid-hydroxy hexadecadienoic acid	FAHFA(14:1-O-16:2)	C30H52O4
141	PDAHHDDA	Pentadecanoic acid-hydroxy hexadecadienoic acid	FAHFA(15:0-O-16:2)	C31H56O4
142	PDEAHHDDA	Pentadecenoic acid-hydroxy hexadecadienoic acid	FAHFA(15:1-O-16:2)	C31H54O4
143	POHHDDA	Palmitoleic acid-hydroxy hexadecadienoic acid	FAHFA(16:1-O-16:2)	C32H56O4

144	PAHHDDA	Palmitic acid-hydroxy hexadecadienoic acid	FAHFA(16:0-O-16:2)	C32H58O4
145	HDAHHDDA	Heptadecanoic acid-hydroxy hexadecadienoic acid	FAHFA(17:0-O-16:2)	C33H60O4
146	HDEAHHDDA	Heptadecenoic acid-hydroxy hexadecadienoic acid	FAHFA(17:1-O-16:2)	C33H58O4
147	SAHHDDA	Stearic acid-hydroxy hexadecadienoic acid	FAHFA(18:0-O-16:2)	C34H62O4
148	OAHHDDA	Oleic acid-hydroxy hexadecadienoic acid	FAHFA(18:1-O-16:2)	C34H60O4
149	LAHHDDA	Linoleic acid-hydroxy hexadecadienoic acid	FAHFA(18:2-O-16:2)	C34H58O4
150	ALAHHDDA	α-Linolenic acid-hydroxy hexadecadienoic acid	FAHFA(18:3-O-16:2)	C34H56O4
151	SDAHHDDA	Stearidonic acid-hydroxy hexadecadienoic acid	FAHFA(18:4-O-16:2)	C34H54O4
152	AAHHDDA	Arachidic acid-hydroxy hexadecadienoic acid	FAHFA(20:0-O-16:2)	C36H66O4
153	EAHHDDA	Eicosenoic acid-hydroxy hexadecadienoic acid	FAHFA(20:1-O-16:2)	C36H64O4
154	EDAHHDDA	Eicosadienoic acid-hydroxy hexadecadienoic acid	FAHFA(20:2-O-16:2)	C36H62O4
155	ETAHHDDA	Eicosatrienoic acid-hydroxy hexadecadienoic acid	FAHFA(20:3-O-16:2)	C36H60O4
156	ARAHHDDA	Arachidonic acid-hydroxy hexadecadienoic acid	FAHFA(20:4-O-16:2)	C36H58O4
157	EPAHHDDA	Eicosapentaenoic acid-hydroxy hexadecadienoic acid	FAHFA(20:5-O-16:2)	C36H56O4
158	BAHHDDA	Behenic acid-hydroxy hexadecadienoic acid	FAHFA(22:0-O-16:2)	C38H70O4
159	DEAHHDDA	Docosenoic acid-hydroxy hexadecadienoic acid	FAHFA(22:1-O-16:2)	C38H68O4
160	DDAHHDDA	Docosadienoate-hydroxy hexadecadienoic acid	FAHFA(22:2-O-16:2)	C38H66O4
161	DHAHHDDA	Docosahexaenoic acid-hydroxy hexadecadienoic acid	FAHFA(22:6-O-16:2)	C38H58O4
162	MAHHTA	Myristic acid-hydroxy hexadecatrienoic acid	FAHFA(14:0-O-16:3)	C30H52O4
163	MOHHTA	Myristoleic acid-hydroxy hexadecatrienoic acid	FAHFA(14:1-O-16:3)	C30H50O4
164	PDAHHTA	Pentadecanoic acid-hydroxy hexadecatrienoic acid	FAHFA(15:0-O-16:3)	C31H54O4
165	PDEAHHTA	Pentadecenoic acid-hydroxy hexadecatrienoic acid	FAHFA(15:1-O-16:3)	C31H52O4
166	POHHTA	Palmitoleic acid-hydroxy hexadecatrienoic acid	FAHFA(16:1-O-16:3)	C32H54O4
167	PAHHTA	Palmitic acid-hydroxy hexadecatrienoic acid	FAHFA(16:0-O-16:3)	C32H56O4
168	HDAHHTA	Heptadecanoic acid-hydroxy hexadecatrienoic acid	FAHFA(17:0-O-16:3)	C33H58O4
169	HDEAHHTA	Heptadecenoic acid-hydroxy hexadecatrienoic acid	FAHFA(17:1-O-16:3)	C33H56O4
170	SAHHTA	Stearic acid-hydroxy hexadecatrienoic acid	FAHFA(18:0-O-16:3)	C34H60O4
171	OAHHTA	Oleic acid-hydroxy hexadecatrienoic acid	FAHFA(18:1-O-16:3)	C34H58O4
172	LAHHTA	Linoleic acid-hydroxy hexadecatrienoic acid	FAHFA(18:2-O-16:3)	C34H56O4
173	ALAHHTA	α-Linolenic acid-hydroxy hexadecatrienoic acid	FAHFA(18:3-O-16:3)	C34H54O4
174	SDAHHTA	Stearidonic acid-hydroxy hexadecatrienoic acid	FAHFA(18:4-O-16:3)	C34H52O4
175	AAHHTA	Arachidic acid-hydroxy hexadecatrienoic acid	FAHFA(20:0-O-16:3)	C36H64O4
176	EAHHTA	Eicosenoic acid-hydroxy hexadecatrienoic acid	FAHFA(20:1-O-16:3)	C36H62O4
177	EDAHHTA	Eicosadienoic acid-hydroxy hexadecatrienoic acid	FAHFA(20:2-O-16:3)	C36H60O4
178	ETAHHTA	Eicosatrienoic acid-hydroxy hexadecatrienoic acid	FAHFA(20:3-O-16:3)	C36H58O4
179	ARAHHTA	Arachidonic acid-hydroxy hexadecatrienoic acid	FAHFA(20:4-O-16:3)	C36H56O4
180	EPAHHTA	Eicosapentaenoic acid-hydroxy hexadecatrienoic acid	FAHFA(20:5-O-16:3)	C36H54O4
181	BAHHTA	Behenic acid-hydroxy hexadecatrienoic acid	FAHFA(22:0-O-16:3)	C38H68O4
182	DEAHHTA	Docosenoic acid-hydroxy hexadecatrienoic acid	FAHFA(22:1-O-16:3)	C38H66O4
183	DDAHHTA	Docosadienoate-hydroxy hexadecatrienoic acid	FAHFA(22:2-O-16:3)	C38H64O4
184	DHAHHTA	Docosahexaenoic acid-hydroxy hexadecatrienoic acid	FAHFA(22:6-O-16:3)	C38H56O4
185	MAHOA	Myristic acid-hydroxy oleic acid	FAHFA(14:0-O-18:1)	C32H60O4
186	MOHOA	Myristoleic acid-hydroxy oleic acid	FAHFA(14:1-O-18:1)	C32H58O4
187	PDAHOA	Pentadecanoic acid-hydroxy oleic acid	FAHFA(15:0-O-18:1)	C33H62O4
188	PDEAHOA	Pentadecenoic acid-hydroxy oleic acid	FAHFA(15:1-O-18:1)	C33H60O4
189	POHOA	Palmitoleic acid-hydroxy oleic acid	FAHFA(16:1-O-18:1)	C34H62O4
190	PAHOA	Palmitic acid-hydroxy oleic acid	FAHFA(16:0-O-18:1)	C34H64O4
191	HDAHOA	Heptadecanoic acid-hydroxy oleic acid	FAHFA(17:0-O-18:1)	C35H66O4
192	HDEAHOA	Heptadecenoic acid-hydroxy oleic acid	FAHFA(17:1-O-18:1)	C35H64O4
193	SAHOA	Stearic acid-hydroxy oleic acid	FAHFA(18:0-O-18:1)	C36H68O4
194	OAHOA	Oleic acid-hydroxy oleic acid	FAHFA(18:1-O-18:1)	C36H66O4
195	LAHOA	Linoleic acid-hydroxy oleic acid	FAHFA(18:2-O-18:1)	C36H64O4
196	ALAHOA	α-Linolenic acid-hydroxy oleic acid	FAHFA(18:3-O-18:1)	C36H62O4
197	SDAHOA	Stearidonic acid-hydroxy oleic acid	FAHFA(18:4-O-18:1)	C36H60O4
198	AAHOA	Arachidic acid-hydroxy oleic acid	FAHFA(20:0-O-18:1)	C38H72O4
199	EAHOA	Eicosenoic acid-hydroxy oleic acid	FAHFA(20:1-O-18:1)	C38H70O4
200	EDAHOA	Eicosadienoic acid-hydroxy oleic acid	FAHFA(20:2-O-18:1)	C38H68O4
201	ETAHOA	Eicosatrienoic acid-hydroxy oleic acid	FAHFA(20:3-O-18:1)	C38H66O4
202	ARAHOA	Arachidonic acid-hydroxy oleic acid	FAHFA(20:4-O-18:1)	C38H64O4
203	EPAHOA	Eicosapentaenoic acid-hydroxy oleic acid	FAHFA(20:5-O-18:1)	C38H62O4
204	BAHOA	Behenic acid-hydroxy oleic acid	FAHFA(22:0-O-18:1)	C40H76O4
205	DEAHOA	Docosenoic acid-hydroxy oleic acid	FAHFA(22:1-O-18:1)	C40H74O4
206	DDAHOA	Docosadienoate-hydroxy oleic acid	FAHFA(22:2-O-18:1)	C40H72O4
207	DHAHOA	Docosahexaenoic acid-hydroxy oleic acid	FAHFA(22:6-O-18:1)	C40H64O4
208	MAHLA	Myristic acid-hydroxy linoleic acid	FAHFA(14:0-O-18:2)	C32H58O4
209	MOHLA	Myristoleic acid-hydroxy linoleic acid	FAHFA(14:1-O-18:2)	C32H56O4
210	PDAHLA	Pentadecanoic acid-hydroxy linoleic acid	FAHFA(15:0-O-18:2)	C33H60O4
211	PDEAHLA	Pentadecenoic acid-hydroxy linoleic acid	FAHFA(15:1-O-18:2)	C33H58O4
212	POHLA	Palmitoleic acid-hydroxy linoleic acid	FAHFA(16:1-O-18:2)	C34H60O4
213	PAHLA	Palmitic acid-hydroxy linoleic acid	FAHFA(16:0-O-18:2)	C34H62O4
214	HDAHLA	Heptadecanoic acid-hydroxy linoleic acid	FAHFA(17:0-O-18:2)	C35H64O4
215	HDEAHLA	Heptadecenoic acid-hydroxy linoleic acid	FAHFA(17:1-O-18:2)	C35H62O4
216	SAHLA	Stearic acid-hydroxy linoleic acid	FAHFA(18:0-O-18:2)	C36H66O4
217	OAHLA	Oleic acid-hydroxy linoleic acid	FAHFA(18:1-O-18:2)	C36H64O4
218	LAHLA	Linoleic acid-hydroxy linoleic acid	FAHFA(18:2-O-18:2)	C36H62O4
219	ALAHLA	α-Linolenic acid-hydroxy linoleic acid	FAHFA(18:3-O-18:2)	C36H60O4
220	SDAHLA	Stearidonic acid-hydroxy linoleic acid	FAHFA(18:4-O-18:2)	C36H58O4

221	AAHLA	Arachidic acid-hydroxy linoleic acid	FAHFA(20:0-O-18:2)	C38H70O4
222	EAHLA	Eicosenoic acid-hydroxy linoleic acid	FAHFA(20:1-O-18:2)	C38H68O4
223	EDAHLA	Eicosadienoic acid-hydroxy linoleic acid	FAHFA(20:2-O-18:2)	C38H66O4
224	ETAHLA	Eicosatrienoic acid-hydroxy linoleic acid	FAHFA(20:3-O-18:2)	C38H64O4
225	ARAHLA	Arachidonic acid-hydroxy linoleic acid	FAHFA(20:4-O-18:2)	C38H62O4
226	EPAHLA	Eicosapentaenoic acid-hydroxy linoleic acid	FAHFA(20:5-O-18:2)	C38H60O4
227	BAHLA	Behenic acid-hydroxy linoleic acid	FAHFA(22:0-O-18:2)	C40H74O4
228	DEAHLA	Docosenoic acid-hydroxy linoleic acid	FAHFA(22:1-O-18:2)	C40H72O4
229	DDAHLA	Docosadienoate-hydroxy linoleic acid	FAHFA(22:2-O-18:2)	C40H70O4
230	DHAHLA	Docosahexaenoic acid-hydroxy linoleic acid	FAHFA(22:6-O-18:2)	C40H62O4
231	MAHALA	Myristic acid-hydroxy α-Linolenic acid	FAHFA(14:0-O-18:3)	C32H56O4
232	MOHALA	Myristoleic acid-hydroxy α-Linolenic acid	FAHFA(14:1-O-18:3)	C32H54O4
233	PDAHALA	Pentadecanoic acid-hydroxy α-Linolenic acid	FAHFA(15:0-O-18:3)	C33H58O4
234	PDEAHALA	Pentadecenoic acid-hydroxy α-Linolenic acid	FAHFA(15:1-O-18:3)	C33H56O4
235	POHALA	Palmitoleic acid-hydroxy α-Linolenic acid	FAHFA(16:1-O-18:3)	C34H58O4
236	PAHALA	Palmitic acid-hydroxy α-Linolenic acid	FAHFA(16:0-O-18:3)	C34H60O4
237	HDAHALA	Heptadecanoic acid-hydroxy α-Linolenic acid	FAHFA(17:0-O-18:3)	C35H62O4
238	HDEAHALA	Heptadecenoic acid-hydroxy α-Linolenic acid	FAHFA(17:1-O-18:3)	C35H60O4
239	SAHALA	Stearic acid-hydroxy α-Linolenic acid	FAHFA(18:0-O-18:3)	C36H64O4
240	OAHALA	Oleic acid-hydroxy α-Linolenic acid	FAHFA(18:1-O-18:3)	C36H62O4
241	LAHALA	Linoleic acid-hydroxy α-Linolenic acid	FAHFA(18:2-O-18:3)	C36H60O4
242	ALAHALA	α-Linolenic acid-hydroxy α-Linolenic acid	FAHFA(18:3-O-18:3)	C36H58O4
243	SDAHALA	Stearidonic acid-hydroxy α-Linolenic acid	FAHFA(18:4-O-18:3)	C36H56O4
244	AAHALA	Arachidic acid-hydroxy α-Linolenic acid	FAHFA(20:0-O-18:3)	C38H68O4
245	EAHALA	Eicosenoic acid-hydroxy α-Linolenic acid	FAHFA(20:1-O-18:3)	C38H66O4
246	EDAHALA	Eicosadienoic acid-hydroxy α-Linolenic acid	FAHFA(20:2-O-18:3)	C38H64O4
247	ETAHALA	Eicosatrienoic acid-hydroxy α-Linolenic acid	FAHFA(20:3-O-18:3)	C38H62O4
248	ARAHALA	Arachidonic acid-hydroxy α-Linolenic acid	FAHFA(20:4-O-18:3)	C38H60O4
249	EPAHALA	Eicosapentaenoic acid-hydroxy α-Linolenic acid	FAHFA(20:5-O-18:3)	C38H58O4
250	BAHALA	Behenic acid-hydroxy α-Linolenic acid	FAHFA(22:0-O-18:3)	C40H72O4
251	DEAHALA	Docosenoic acid-hydroxy α-Linolenic acid	FAHFA(22:1-O-18:3)	C40H70O4
252	DDAHALA	Docosadienoate-hydroxy α-Linolenic acid	FAHFA(22:2-O-18:3)	C40H68O4
253	DHAHALA	Docosahexaenoic acid-hydroxy α-Linolenic acid	FAHFA(22:6-O-18:3)	C40H60O4
254	MAHEA	Myristic acid-hydroxy eicosenoic acid	FAHFA(14:0-O-20:1)	C34H64O4
255	MOHEA	Myristoleic acid-hydroxy eicosenoic acid	FAHFA(14:1-O-20:1)	C34H62O4
256	PDAHEA	Pentadecanoic acid-hydroxy eicosenoic acid	FAHFA(15:0-O-20:1)	C35H66O4
257	PDEAHEA	Pentadecenoic acid-hydroxy eicosenoic acid	FAHFA(15:1-O-20:1)	C35H64O4
258	POHEA	Palmitoleic acid-hydroxy eicosenoic acid	FAHFA(16:1-O-20:1)	C36H66O4
259	PAHEA	Palmitic acid-hydroxy eicosenoic acid	FAHFA(16:0-O-20:1)	C36H68O4
260	HDAHEA	Heptadecanoic acid-hydroxy eicosenoic acid	FAHFA(17:0-O-20:1)	C37H70O4
261	HDEAHEA	Heptadecenoic acid-hydroxy eicosenoic acid	FAHFA(17:1-O-20:1)	C37H68O4
262	SAHEA	Stearic acid-hydroxy eicosenoic acid	FAHFA(18:0-O-20:1)	C38H72O4
263	OAHEA	Oleic acid-hydroxy eicosenoic acid	FAHFA(18:1-O-20:1)	C38H70O4
264	LAHEA	Linoleic acid-hydroxy eicosenoic acid	FAHFA(18:2-O-20:1)	C38H68O4
265	ALAHEA	α-Linolenic acid-hydroxy eicosenoic acid	FAHFA(18:3-O-20:1)	C38H66O4
266	SDAHEA	Stearidonic acid-hydroxy eicosenoic acid	FAHFA(18:4-O-20:1)	C38H64O4
267	AAHEA	Arachidic acid-hydroxy eicosenoic acid	FAHFA(20:0-O-20:1)	C40H76O4
268	EAHEA	Eicosenoic acid-hydroxy eicosenoic acid	FAHFA(20:1-O-20:1)	C40H74O4
269	EDAHEA	Eicosadienoic acid-hydroxy eicosenoic acid	FAHFA(20:2-O-20:1)	C40H72O4
270	ETAHEA	Eicosatrienoic acid-hydroxy eicosenoic acid	FAHFA(20:3-O-20:1)	C40H70O4
271	ARAHEA	Arachidonic acid-hydroxy eicosenoic acid	FAHFA(20:4-O-20:1)	C40H68O4
272	EPAHEA	Eicosapentaenoic acid-hydroxy eicosenoic acid	FAHFA(20:5-O-20:1)	C40H66O4
273	BAHEA	Behenic acid-hydroxy eicosenoic acid	FAHFA(22:0-O-20:1)	C42H80O4
274	DEAHEA	Docosenoic acid-hydroxy eicosenoic acid	FAHFA(22:1-O-20:1)	C42H78O4
275	DDAHEA	Docosadienoate-hydroxy eicosenoic acid	FAHFA(22:2-O-20:1)	C40H76O4
276	DHAHEA	Docosahexaenoic acid-hydroxy eicosenoic acid	FAHFA(22:6-O-20:1)	C42H68O4
277	MAHEDA	Myristic acid-hydroxy eicosadienoic acid	FAHFA(14:0-O-20:2)	C34H62O4
278	MOHEDA	Myristoleic acid-hydroxy eicosadienoic acid	FAHFA(14:1-O-20:2)	C34H60O4
279	PDAHEDA	Pentadecanoic acid-hydroxy eicosadienoic acid	FAHFA(15:0-O-20:2)	C35H64O4
280	PDEAHEDA	Pentadecenoic acid-hydroxy eicosadienoic acid	FAHFA(15:1-O-20:2)	C35H62O4
281	POHEDA	Palmitoleic acid-hydroxy eicosadienoic acid	FAHFA(16:1-O-20:2)	C36H64O4
282	PAHEDA	Palmitic acid-hydroxy eicosadienoic acid	FAHFA(16:0-O-20:2)	C36H66O4
283	HDAHEDA	Heptadecanoic acid-hydroxy eicosadienoic acid	FAHFA(17:0-O-20:2)	C37H68O4
284	HDEAHEDA	Heptadecenoic acid-hydroxy eicosadienoic acid	FAHFA(17:1-O-20:2)	C37H66O4
285	SAHEDA	Stearic acid-hydroxy eicosadienoic acid	FAHFA(18:0-O-20:2)	C38H70O4
286	OAHEDA	Oleic acid-hydroxy eicosadienoic acid	FAHFA(18:1-O-20:2)	C38H68O4
287	LAHEDA	Linoleic acid-hydroxy eicosadienoic acid	FAHFA(18:2-O-20:2)	C38H66O4
288	ALAHEDA	α-Linolenic acid-hydroxy eicosadienoic acid	FAHFA(18:3-O-20:2)	C38H64O4
289	SDAHEDA	Stearidonic acid-hydroxy eicosadienoic acid	FAHFA(18:4-O-20:2)	C38H62O4
290	AAHEDA	Arachidic acid-hydroxy eicosadienoic acid	FAHFA(20:0-O-20:2)	C40H74O4
291	EAHEDA	Eicosenoic acid-hydroxy eicosadienoic acid	FAHFA(20:1-O-20:2)	C40H72O4
292	EDAHEDA	Eicosadienoic acid-hydroxy eicosadienoic acid	FAHFA(20:2-O-20:2)	C40H70O4
293	ETAHEDA	Eicosatrienoic acid-hydroxy eicosadienoic acid	FAHFA(20:3-O-20:2)	C40H68O4
294	ARAHEDA	Arachidonic acid-hydroxy eicosadienoic acid	FAHFA(20:4-O-20:2)	C40H66O4
295	EPAHEDA	Eicosapentaenoic acid-hydroxy eicosadienoic acid	FAHFA(20:5-O-20:2)	C40H64O4
296	BAHEDA	Behenic acid-hydroxy eicosadienoic acid	FAHFA(22:0-O-20:2)	C42H78O4
297	DEAHEDA	Docosenoic acid-hydroxy eicosadienoic acid	FAHFA(22:1-O-20:2)	C42H76O4

298	DDAHEDA	Docosadienoate-hydroxy eicosadienoic acid	FAHFA(22:2-O-20:2)	C40H74O4
299	DHAHEDA	Docosahexaenoic acid-hydroxy eicosadienoic acid	FAHFA(22:6-O-20:2)	C42H66O4
300	MAHETA	Myristic acid-hydroxy eicosatrienoic acid	FAHFA(14:0-O-20:3)	C34H60O4
301	MOHETA	Myristoleic acid-hydroxy eicosatrienoic acid	FAHFA(14:1-O-20:3)	C34H58O4
302	PDAHETA	Pentadecanoic acid-hydroxy eicosatrienoic acid	FAHFA(15:0-O-20:3)	C35H62O4
303	PDEAHETA	Pentadecenoic acid-hydroxy eicosatrienoic acid	FAHFA(15:1-O-20:3)	C35H60O4
304	POHETA	Palmitoleic acid-hydroxy eicosatrienoic acid	FAHFA(16:1-O-20:3)	C36H62O4
305	PAHETA	Palmitic acid-hydroxy eicosatrienoic acid	FAHFA(16:0-O-20:3)	C36H64O4
306	HDAHETA	Heptadecanoic acid-hydroxy eicosatrienoic acid	FAHFA(17:0-O-20:3)	C37H66O4
307	HDEAHETA	Heptadecenoic acid-hydroxy eicosatrienoic acid	FAHFA(17:1-O-20:3)	C37H64O4
308	SAHETA	Stearic acid-hydroxy eicosatrienoic acid	FAHFA(18:0-O-20:3)	C38H68O4
309	OAHETA	Oleic acid-hydroxy eicosatrienoic acid	FAHFA(18:1-O-20:3)	C38H66O4
310	LAHETA	Linoleic acid-hydroxy eicosatrienoic acid	FAHFA(18:2-O-20:3)	C38H64O4
311	ALAHETA	α-Linolenic acid-hydroxy eicosatrienoic acid	FAHFA(18:3-O-20:3)	C38H62O4
312	SDAHETA	Stearidonic acid-hydroxy eicosatrienoic acid	FAHFA(18:4-O-20:3)	C38H60O4
313	AAHETA	Arachidic acid-hydroxy eicosatrienoic acid	FAHFA(20:0-O-20:3)	C40H72O4
314	EAHETA	Eicosenoic acid-hydroxy eicosatrienoic acid	FAHFA(20:1-O-20:3)	C40H70O4
315	EDAHETA	Eicosadienoic acid-hydroxy eicosatrienoic acid	FAHFA(20:2-O-20:3)	C40H68O4
316	ETAHETA	Eicosatrienoic acid-hydroxy eicosatrienoic acid	FAHFA(20:3-O-20:3)	C40H66O4
317	ARAHETA	Arachidonic acid-hydroxy eicosatrienoic acid	FAHFA(20:4-O-20:3)	C40H64O4
318	EPAHETA	Eicosapentaenoic acid-hydroxy eicosatrienoic acid	FAHFA(20:5-O-20:3)	C40H62O4
319	BAHETA	Behenic acid-hydroxy eicosatrienoic acid	FAHFA(22:0-O-20:3)	C42H76O4
320	DEAHETA	Docosenoic acid-hydroxy eicosatrienoic acid	FAHFA(22:1-O-20:3)	C42H74O4
321	DDAHETA	Docosadienoate-hydroxy eicosatrienoic acid	FAHFA(22:2-O-20:3)	C40H72O4
322	DHAHETA	Docosahexaenoic acid-hydroxy eicosatrienoic acid	FAHFA(22:6-O-20:3)	C42H64O4
323	MAHDHA	Myristic acid-hydroxy docosahexaenoic acid	FAHFA(14:0-O-22:6)	C36H58O4
324	MOHDHA	Myristoleic acid-hydroxy docosahexaenoic acid	FAHFA(14:1-O-22:6)	C36H56O4
325	PDAHDHA	Pentadecanoic acid-hydroxy docosahexaenoic acid	FAHFA(15:0-O-22:6)	C37H60O4
326	PDEAHDHA	Pentadecenoic acid-hydroxy docosahexaenoic acid	FAHFA(15:1-O-22:6)	C37H58O4
327	POHDHA	Palmitoleic acid-hydroxy docosahexaenoic acid	FAHFA(16:1-O-22:6)	C38H60O4
328	PAHDHA	Palmitic acid-hydroxy docosahexaenoic acid	FAHFA(16:0-O-22:6)	C38H62O4
329	HDAHDHA	Heptadecanoic acid-hydroxy docosahexaenoic acid	FAHFA(17:0-O-22:6)	C39H64O4
330	HDEAHDHA	Heptadecenoic acid-hydroxy docosahexaenoic acid	FAHFA(17:1-O-22:6)	C39H62O4
331	SAHDHA	Stearic acid-hydroxy docosahexaenoic acid	FAHFA(18:0-O-22:6)	C40H66O4
332	OAHDHA	Oleic acid-hydroxy docosahexaenoic acid	FAHFA(18:1-O-22:6)	C40H64O4
333	LAHDHA	Linoleic acid-hydroxy docosahexaenoic acid	FAHFA(18:2-O-22:6)	C40H62O4
334	ALAHDHA	α-Linolenic acid-hydroxy docosahexaenoic acid	FAHFA(18:3-O-22:6)	C40H60O4
335	SDAHDHA	Stearidonic acid-hydroxy docosahexaenoic acid	FAHFA(18:4-O-22:6)	C40H58O4
336	AAHDHA	Arachidic acid-hydroxy docosahexaenoic acid	FAHFA(20:0-O-22:6)	C42H70O4
337	EAHDHA	Eicosenoic acid-hydroxy docosahexaenoic acid	FAHFA(20:1-O-22:6)	C42H68O4
338	EDAHDHA	Eicosadienoic acid-hydroxy docosahexaenoic acid	FAHFA(20:2-O-22:6)	C42H66O4
339	ETAHDHA	Eicosatrienoic acid-hydroxy docosahexaenoic acid	FAHFA(20:3-O-22:6)	C42H64O4
340	ARAHDHA	Arachidonic acid-hydroxy docosahexaenoic acid	FAHFA(20:4-O-22:6)	C42H62O4
341	EPAHDHA	Eicosapentaenoic acid-hydroxy docosahexaenoic acid	FAHFA(20:5-O-22:6)	C42H60O4
342	BAHDHA	Behenic acid-hydroxy docosahexaenoic acid	FAHFA(22:0-O-22:6)	C44H74O4
343	DEAHDHA	Docosenoic acid-hydroxy docosahexaenoic acid	FAHFA(22:1-O-22:6)	C44H72O4
344	DDAHDHA	Docosadienoate-hydroxy docosahexaenoic acid	FAHFA(22:2-O-22:6)	C44H70O4
345	DHAHDHA	Docosahexaenoic acid-hydroxy docosahexaenoic acid	FAHFA(22:6-O-22:6)	C44H62O4
346	MAHARA	Myristic acid-hydroxy arachidonic acid	FAHFA(14:0-O-20:4)	C34H58O4
347	MOHARA	Myristoleic acid-hydroxy arachidonic acid	FAHFA(14:1-O-20:4)	C34H56O4
348	PDAHARA	Pentadecanoic acid-hydroxy arachidonic acid	FAHFA(15:0-O-20:4)	C35H60O4
349	PDEAHARA	Pentadecenoic acid-hydroxy arachidonic acid	FAHFA(15:1-O-20:4)	C35H58O4
350	POHARA	Palmitoleic acid-hydroxy arachidonic acid	FAHFA(16:1-O-20:4)	C36H60O4
351	PAHARA	Palmitic acid-hydroxy arachidonic acid	FAHFA(16:0-O-20:4)	C36H62O4
352	HDAHARA	Heptadecanoic acid-hydroxy arachidonic acid	FAHFA(17:0-O-20:4)	C37H64O4
353	HDEAHARA	Heptadecenoic acid-hydroxy arachidonic acid	FAHFA(17:1-O-20:4)	C37H62O4
354	SAHARA	Stearic acid-hydroxy arachidonic acid	FAHFA(18:0-O-20:4)	C38H66O4
355	OAHARA	Oleic acid-hydroxy arachidonic acid	FAHFA(18:1-O-20:4)	C38H64O4
356	LAHARA	Linoleic acid-hydroxy arachidonic acid	FAHFA(18:2-O-20:4)	C38H62O4
357	ALAHARA	α-Linolenic acid-hydroxy arachidonic acid	FAHFA(18:3-O-20:4)	C38H60O4
358	SDAHARA	Stearidonic acid-hydroxy arachidonic acid	FAHFA(18:4-O-20:4)	C38H58O4
359	AAHARA	Arachidic acid-hydroxy arachidonic acid	FAHFA(20:0-O-20:4)	C40H70O4
360	EAHARA	Eicosenoic acid-hydroxy arachidonic acid	FAHFA(20:1-O-20:4)	C40H68O4
361	EDAHARA	Eicosadienoic acid-hydroxy arachidonic acid	FAHFA(20:2-O-20:4)	C40H66O4
362	ETAHARA	Eicosatrienoic acid-hydroxy arachidonic acid	FAHFA(20:3-O-20:4)	C40H64O4
363	ARAHARA	Arachidonic acid-hydroxy arachidonic acid	FAHFA(20:4-O-20:4)	C40H62O4
364	EPAHARA	Eicosapentaenoic acid-hydroxy arachidonic acid	FAHFA(20:5-O-20:4)	C40H60O4
365	BAHARA	Behenic acid-hydroxy arachidonic acid	FAHFA(22:0-O-20:4)	C42H74O4
366	DEAHARA	Docosenoic acid-hydroxy arachidonic acid	FAHFA(22:1-O-20:4)	C42H72O4
367	DDAHARA	Docosadienoate-hydroxy arachidonic acid	FAHFA(22:2-O-20:4)	C40H70O4
368	DHAHARA	Docosahexaenoic acid-hydroxy arachidonic acid	FAHFA(22:6-O-20:4)	C42H62O4
369	MAHDA	Myristic acid-hydroxy dodecanoic acid	FAHFA(14:0-O-12:0)	C26H50O4
370	MOHDA	Myristoleic acid-hydroxy dodecanoic acid	FAHFA(14:1-O-12:0)	C22H48O4
371	PDAHDA	Pentadecanoic acid-hydroxy dodecanoic acid	FAHFA(15:0-O-12:0)	C27H52O4
372	PDEAHDA	Pentadecenoic acid-hydroxy dodecanoic acid	FAHFA(15:1-O-12:0)	C27H50O4
373	POHDA	Palmitoleic acid-hydroxy dodecanoic acid	FAHFA(16:1-O-12:0)	C28H52O4
374	PAHDA	Palmitic acid-hydroxy dodecanoic acid	FAHFA(16:0-O-12:0)	C28H54O4

375	HDAHDA	Heptadecanoic acid-hydroxy dodecanoic acid	FAHFA(17:0-O-12:0)	C29H56O4
376	HDEAHDA	Heptadecenoic acid-hydroxy dodecanoic acid	FAHFA(17:1-O-12:0)	C29H54O4
377	SAHDA	Stearic acid-hydroxy dodecanoic acid	FAHFA(18:0-O-12:0)	C30H58O4
378	OAHDA	Oleic acid-hydroxy dodecanoic acid	FAHFA(18:1-O-12:0)	C30H56O4
379	LAHDA	Linoleic acid-hydroxy dodecanoic acid	FAHFA(18:2-O-12:0)	C30H54O4
380	ALAHDA	α-Linolenic acid-hydroxy dodecanoic acid	FAHFA(18:3-O-12:0)	C30H52O4
381	SDAHDA	Stearidonic acid-hydroxy dodecanoic acid	FAHFA(18:4-O-12:0)	C30H50O4
382	AAHDA	Arachidic acid-hydroxy dodecanoic acid	FAHFA(20:0-O-12:0)	C32H62O4
383	EAHDA	Eicosenoic acid-hydroxy dodecanoic acid	FAHFA(20:1-O-12:0)	C32H60O4
384	EDAHDA	Eicosadienoic acid-hydroxy dodecanoic acid	FAHFA(20:2-O-12:0)	C32H58O4
385	ETAHDA	Eicosatrienoic acid-hydroxy dodecanoic acid	FAHFA(20:3-O-12:0)	C32H56O4
386	ARAHDA	Arachidonic acid-hydroxy dodecanoic acid	FAHFA(20:4-O-12:0)	C32H54O4
387	EPAHDA	Eicosapentaenoic acid-hydroxy dodecanoic acid	FAHFA(20:5-O-12:0)	C32H52O4
388	BAHDA	Behenic acid-hydroxy dodecanoic acid	FAHFA(22:0-O-12:0)	C34H66O4
389	DEAHDA	Docosenoic acid-hydroxy dodecanoic acid	FAHFA(22:1-O-12:0)	C34H64O4
390	DDAHDA	Docosadienoate-hydroxy dodecanoic acid	FAHFA(22:2-O-12:0)	C34H62O4
391	DHAHDA	Docosahexaenoic acid-hydroxy dodecanoic acid	FAHFA(22:6-O-12:0)	C34H54O4
392	MAHMA	Myristic acid-hydroxy myristic acid	FAHFA(14:0-O-14:0)	C28H54O4
393	MOHMA	Myristoleic acid-hydroxy myristic acid	FAHFA(14:1-O-14:0)	C28H52O4
394	PDAHMA	Pentadecanoic acid-hydroxy myristic acid	FAHFA(15:0-O-14:0)	C29H56O4
395	PDEAHMA	Pentadecenoic acid-hydroxy myristic acid	FAHFA(15:1-O-14:0)	C29H54O4
396	POHMA	Palmitoleic acid-hydroxy myristic acid	FAHFA(16:1-O-14:0)	C30H56O4
397	PAHMA	Palmitic acid-hydroxy myristic acid	FAHFA(16:0-O-14:0)	C30H58O4
398	HDAHMA	Heptadecanoic acid-hydroxy myristic acid	FAHFA(17:0-O-14:0)	C31H60O4
399	HDEAHMA	Heptadecenoic acid-hydroxy myristic acid	FAHFA(17:1-O-14:0)	C31H58O4
400	SAHMA	Stearic acid-hydroxy myristic acid	FAHFA(18:0-O-14:0)	C32H62O4
401	OAHMA	Oleic acid-hydroxy myristic acid	FAHFA(18:1-O-14:0)	C32H60O4
402	LAHMA	Linoleic acid-hydroxy myristic acid	FAHFA(18:2-O-14:0)	C32H58O4
403	ALAHMA	α-Linolenic acid-hydroxy myristic acid	FAHFA(18:3-O-14:0)	C32H56O4
404	SDAHMA	Stearidonic acid-hydroxy myristic acid	FAHFA(18:4-O-14:0)	C32H54O4
405	AAHMA	Arachidic acid-hydroxy myristic acid	FAHFA(20:0-O-14:0)	C34H66O4
406	EAHMA	Eicosenoic acid-hydroxy myristic acid	FAHFA(20:1-O-14:0)	C34H64O4
407	EDAHMA	Eicosadienoic acid-hydroxy myristic acid	FAHFA(20:2-O-14:0)	C34H62O4
408	ETAHMA	Eicosatrienoic acid-hydroxy myristic acid	FAHFA(20:3-O-14:0)	C34H60O4
409	ARAHMA	Arachidonic acid-hydroxy myristic acid	FAHFA(20:4-O-14:0)	C34H58O4
410	EPAHMA	Eicosapentaenoic acid-hydroxy myristic acid	FAHFA(20:5-O-14:0)	C34H56O4
411	BAHMA	Behenic acid-hydroxy myristic acid	FAHFA(22:0-O-14:0)	C36H70O4
412	DEAHMA	Docosenoic acid-hydroxy myristic acid	FAHFA(22:1-O-14:0)	C36H68O4
413	DDAHMA	Docosadienoate-hydroxy myristic acid	FAHFA(22:2-O-14:0)	C36H66O4
414	DHAHMA	Docosahexaenoic acid-hydroxy myristic acid	FAHFA(22:6-O-14:0)	C36H58O4
415	MAHPDA	Myristic acid-hydroxy pentadecanoic acid	FAHFA(14:0-O-15:0)	C29H56O4
416	MOHPDA	Myristoleic acid-hydroxy pentadecanoic acid	FAHFA(14:1-O-15:0)	C29H54O4
417	PDAHPDA	Pentadecanoic acid-hydroxy pentadecanoic acid	FAHFA(15:0-O-15:0)	C30H58O4
418	PDEAHPDA	Pentadecenoic acid-hydroxy pentadecanoic acid	FAHFA(15:1-O-15:0)	C30H56O4
419	POHPDA	Palmitoleic acid-hydroxy pentadecanoic acid	FAHFA(16:1-O-15:0)	C31H58O4
420	PAHPDA	Palmitic acid-hydroxy pentadecanoic acid	FAHFA(16:0-O-15:0)	C31H60O4
421	HDAHPDA	Heptadecanoic acid-hydroxy pentadecanoic acid	FAHFA(17:0-O-15:0)	C32H62O4
422	HDEAHPDA	Heptadecenoic acid-hydroxy pentadecanoic acid	FAHFA(17:1-O-15:0)	C32H60O4
423	SAHPDA	Stearic acid-hydroxy pentadecanoic acid	FAHFA(18:0-O-15:0)	C33H64O4
424	OAHPDA	Oleic acid-hydroxy pentadecanoic acid	FAHFA(18:1-O-15:0)	C33H62O4
425	LAHPDA	Linoleic acid-hydroxy pentadecanoic acid	FAHFA(18:2-O-15:0)	C33H60O4
426	ALAHPDA	α-Linolenic acid-hydroxy pentadecanoic acid	FAHFA(18:3-O-15:0)	C33H58O4
427	SDAHPDA	Stearidonic acid-hydroxy pentadecanoic acid	FAHFA(18:4-O-15:0)	C33H56O4
428	AAHPDA	Arachidic acid-hydroxy pentadecanoic acid	FAHFA(20:0-O-15:0)	C35H68O4
429	EAHPDA	Eicosenoic acid-hydroxy pentadecanoic acid	FAHFA(20:1-O-15:0)	C35H66O4
430	EDAHPDA	Eicosadienoic acid-hydroxy pentadecanoic acid	FAHFA(20:2-O-15:0)	C35H64O4
431	ETAHPDA	Eicosatrienoic acid-hydroxy pentadecanoic acid	FAHFA(20:3-O-15:0)	C35H62O4
432	ARAHPDA	Arachidonic acid-hydroxy pentadecanoic acid	FAHFA(20:4-O-15:0)	C35H60O4
433	EPAHPDA	Eicosapentaenoic acid-hydroxy pentadecanoic acid	FAHFA(20:5-O-15:0)	C35H58O4
434	BAHPDA	Behenic acid-hydroxy pentadecanoic acid	FAHFA(22:0-O-15:0)	C37H72O4
435	DEAHPDA	Docosenoic acid-hydroxy pentadecanoic acid	FAHFA(22:1-O-15:0)	C37H70O4
436	DDAHPDA	Docosadienoate-hydroxy pentadecanoic acid	FAHFA(22:2-O-15:0)	C37H68O4
437	DHAHPDA	Docosahexaenoic acid-hydroxy pentadecanoic acid	FAHFA(22:6-O-15:0)	C37H60O4
438	MAHPA	Myristic acid-hydroxy palmitic acid	FAHFA(14:0-O-16:0)	C30H58O4
439	MOHPA	Myristoleic acid-hydroxy palmitic acid	FAHFA(14:1-O-16:0)	C30H56O4
440	PDAHPA	Pentadecanoic acid-hydroxy palmitic acid	FAHFA(15:0-O-16:0)	C31H60O4
441	PDEAHPA	Pentadecenoic acid-hydroxy palmitic acid	FAHFA(15:1-O-16:0)	C31H58O4
442	POHPA	Palmitoleic acid-hydroxy palmitic acid	FAHFA(16:1-O-16:0)	C32H60O4
443	PAHPA	Palmitic acid-hydroxy palmitic acid	FAHFA(16:0-O-16:0)	C32H62O4
444	HDAHPA	Heptadecanoic acid-hydroxy palmitic acid	FAHFA(17:0-O-16:0)	C33H64O4
445	HDEAHPA	Heptadecenoic acid-hydroxy palmitic acid	FAHFA(17:1-O-16:0)	C33H62O4
446	SAHPA	Stearic acid-hydroxy palmitic acid	FAHFA(18:0-O-16:0)	C34H66O4
447	OAHPA	Oleic acid-hydroxy palmitic acid	FAHFA(18:1-O-16:0)	C34H64O4
448	LAHPA	Linoleic acid-hydroxy palmitic acid	FAHFA(18:2-O-16:0)	C34H62O4
449	ALAHPA	α-Linolenic acid-hydroxy palmitic acid	FAHFA(18:3-O-16:0)	C34H60O4
450	SDAHPA	Stearidonic acid-hydroxy palmitic acid	FAHFA(18:4-O-16:0)	C34H58O4
451	AAHPA	Arachidic acid-hydroxy palmitic acid	FAHFA(20:0-O-16:0)	C36H70O4

452	EAHPA	Eicosenoic acid-hydroxy palmitic acid	FAHFA(20:1-O-16:0)	C36H68O4
453	EDAHPA	Eicosadienoic acid-hydroxy palmitic acid	FAHFA(20:2-O-16:0)	C36H66O4
454	ETAHPA	Eicosatrienoic acid-hydroxy palmitic acid	FAHFA(20:3-O-16:0)	C36H64O4
455	ARAHPA	Arachidonic acid-hydroxy palmitic acid	FAHFA(20:4-O-16:0)	C36H62O4
456	EPAHPA	Eicosapentaenoic acid-hydroxy palmitic acid	FAHFA(20:5-O-16:0)	C36H60O4
457	BAHPA	Behenic acid-hydroxy palmitic acid	FAHFA(22:0-O-16:0)	C38H74O4
458	DEAHPA	Docosenoic acid-hydroxy palmitic acid	FAHFA(22:1-O-16:0)	C38H72O4
459	DDAHPA	Docosadienoate-hydroxy palmitic acid	FAHFA(22:2-O-16:0)	C38H70O4
460	DHAHPA	Docosahexaenoic acid-hydroxy palmitic acid	FAHFA(22:6-O-16:0)	C38H62O4
461	MAHHDA	Myristic acid-hydroxy heptadecanoic acid	FAHFA(14:0-O-17:0)	C31H60O4
462	MOHHDA	Myristoleic acid-hydroxy heptadecanoic acid	FAHFA(14:1-O-17:0)	C31H58O4
463	PDAHHDA	Pentadecanoic acid-hydroxy heptadecanoic acid	FAHFA(15:0-O-17:0)	C32H62O4
464	PDEAHHDA	Pentadecenoic acid-hydroxy heptadecanoic acid	FAHFA(15:1-O-17:0)	C32H60O4
465	POHHDA	Palmitoleic acid-hydroxy heptadecanoic acid	FAHFA(16:1-O-17:0)	C33H62O4
466	PAHHDA	Palmitic acid-hydroxy heptadecanoic acid	FAHFA(16:0-O-17:0)	C33H64O4
467	HDAHHDA	Heptadecanoic acid-hydroxy heptadecanoic acid	FAHFA(17:0-O-17:0)	C34H66O4
468	HDEAHHDA	Heptadecenoic acid-hydroxy heptadecanoic acid	FAHFA(17:1-O-17:0)	C34H64O4
469	SAHHDA	Stearic acid-hydroxy heptadecanoic acid	FAHFA(18:0-O-17:0)	C35H68O4
470	OAHHDA	Oleic acid-hydroxy heptadecanoic acid	FAHFA(18:1-O-17:0)	C35H66O4
471	LAHHDA	Linoleic acid-hydroxy heptadecanoic acid	FAHFA(18:2-O-17:0)	C35H64O4
472	ALAHHDA	α-Linolenic acid-hydroxy heptadecanoic acid	FAHFA(18:3-O-17:0)	C35H62O4
473	SDAHHDA	Stearidonic acid-hydroxy heptadecanoic acid	FAHFA(18:4-O-17:0)	C35H60O4
474	AAHHDA	Arachidic acid-hydroxy heptadecanoic acid	FAHFA(20:0-O-17:0)	C37H72O4
475	EAHHDA	Eicosenoic acid-hydroxy heptadecanoic acid	FAHFA(20:1-O-17:0)	C37H70O4
476	EDAHHDA	Eicosadienoic acid-hydroxy heptadecanoic acid	FAHFA(20:2-O-17:0)	C37H68O4
477	ETAHHDA	Eicosatrienoic acid-hydroxy heptadecanoic acid	FAHFA(20:3-O-17:0)	C37H66O4
478	ARAHHDA	Arachidonic acid-hydroxy heptadecanoic acid	FAHFA(20:4-O-17:0)	C37H64O4
479	EPAHHDA	Eicosapentaenoic acid-hydroxy heptadecanoic acid	FAHFA(20:5-O-17:0)	C37H62O4
480	BAHHDA	Behenic acid-hydroxy heptadecanoic acid	FAHFA(22:0-O-17:0)	C39H76O4
481	DEAHHDA	Docosenoic acid-hydroxy heptadecanoic acid	FAHFA(22:1-O-17:0)	C39H74O4
482	DDAHHDA	Docosadienoate-hydroxy heptadecanoic acid	FAHFA(22:2-O-17:0)	C39H72O4
483	DHAHHDA	Docosahexaenoic acid-hydroxy heptadecanoic acid	FAHFA(22:6-O-17:0)	C39H64O4
484	MAHSA	Myristic acid-hydroxy stearic acid	FAHFA(14:0-O-18:0)	C32H62O4
485	MOHSA	Myristoleic acid-hydroxy stearic acid	FAHFA(14:1-O-18:0)	C32H60O4
486	PDAHSA	Pentadecanoic acid-hydroxy stearic acid	FAHFA(15:0-O-18:0)	C33H64O4
487	PDEAHSA	Pentadecenoic acid-hydroxy stearic acid	FAHFA(15:1-O-18:0)	C33H62O4
488	POHSA	Palmitoleic acid-hydroxy stearic acid	FAHFA(16:1-O-18:0)	C34H64O4
489	PAHSA	Palmitic acid-hydroxy stearic acid	FAHFA(16:0-O-18:0)	C34H66O4
490	HDAHSA	Heptadecanoic acid-hydroxy stearic acid	FAHFA(17:0-O-18:0)	C35H68O4
491	HDEAHSA	Heptadecenoic acid-hydroxy stearic acid	FAHFA(17:1-O-18:0)	C35H66O4
492	SAHSA	Stearic acid-hydroxy stearic acid	FAHFA(18:0-O-18:0)	C36H70O4
493	OAHSA	Oleic acid-hydroxy stearic acid	FAHFA(18:1-O-18:0)	C36H68O4
494	LAHSA	Linoleic acid-hydroxy stearic acid	FAHFA(18:2-O-18:0)	C36H66O4
495	ALAHSA	α-Linolenic acid-hydroxy stearic acid	FAHFA(18:3-O-18:0)	C36H64O4
496	SDAHSA	Stearidonic acid-hydroxy stearic acid	FAHFA(18:4-O-18:0)	C36H62O4
497	AAHSA	Arachidic acid-hydroxy stearic acid	FAHFA(20:0-O-18:0)	C38H74O4
498	EAHSA	Eicosenoic acid-hydroxy stearic acid	FAHFA(20:1-O-18:0)	C38H72O4
499	EDAHSA	Eicosadienoic acid-hydroxy stearic acid	FAHFA(20:2-O-18:0)	C38H70O4
500	ETAHSA	Eicosatrienoic acid-hydroxy stearic acid	FAHFA(20:3-O-18:0)	C38H68O4
501	ARAHSA	Arachidonic acid-hydroxy stearic acid	FAHFA(20:4-O-18:0)	C38H66O4
502	EPAHSA	Eicosapentaenoic acid-hydroxy stearic acid	FAHFA(20:5-O-18:0)	C38H64O4
503	BAHSA	Behenic acid-hydroxy stearic acid	FAHFA(22:0-O-18:0)	C40H78O4
504	DEAHSA	Docosenoic acid-hydroxy stearic acid	FAHFA(22:1-O-18:0)	C40H76O4
505	DDAHSA	Docosadienoate-hydroxy stearic acid	FAHFA(22:2-O-18:0)	C40H74O4
506	DHAHSA	Docosahexaenoic acid-hydroxy stearic acid	FAHFA(22:6-O-18:0)	C40H66O4
507	MAHNDA	Myristic acid-hydroxy nonadecanoic acid	FAHFA(14:0-O-19:0)	C33H64O4
508	MOHNDA	Myristoleic acid-hydroxy nonadecanoic acid	FAHFA(14:1-O-19:0)	C33H62O4
509	PDAHNDA	Pentadecanoic acid-hydroxy nonadecanoic acid	FAHFA(15:0-O-19:0)	C34H66O4
510	PDEAHNDA	Pentadecenoic acid-hydroxy nonadecanoic acid	FAHFA(15:1-O-19:0)	C34H64O4
511	POHNDA	Palmitoleic acid-hydroxy nonadecanoic acid	FAHFA(16:1-O-19:0)	C35H66O4
512	PAHNDA	Palmitic acid-hydroxy nonadecanoic acid	FAHFA(16:0-O-19:0)	C35H68O4
513	HDAHNDA	Heptadecanoic acid-hydroxy nonadecanoic acid	FAHFA(17:0-O-19:0)	C36H70O4
514	HDEAHNDA	Heptadecenoic acid-hydroxy nonadecanoic acid	FAHFA(17:1-O-19:0)	C36H68O4
515	SAHNDA	Stearic acid-hydroxy nonadecanoic acid	FAHFA(18:0-O-19:0)	C37H72O4
516	OAHNDA	Oleic acid-hydroxy nonadecanoic acid	FAHFA(18:1-O-19:0)	C37H70O4
517	LAHNDA	Linoleic acid-hydroxy nonadecanoic acid	FAHFA(18:2-O-19:0)	C37H68O4
518	ALAHNDA	α-Linolenic acid-hydroxy nonadecanoic acid	FAHFA(18:3-O-19:0)	C37H66O4
519	SDAHNDA	Stearidonic acid-hydroxy nonadecanoic acid	FAHFA(18:4-O-19:0)	C37H64O4
520	AAHNDA	Arachidic acid-hydroxy nonadecanoic acid	FAHFA(20:0-O-19:0)	C39H76O4
521	EAHNDA	Eicosenoic acid-hydroxy nonadecanoic acid	FAHFA(20:1-O-19:0)	C39H74O4
522	EDAHNDA	Eicosadienoic acid-hydroxy nonadecanoic acid	FAHFA(20:2-O-19:0)	C39H72O4
523	ETAHNDA	Eicosatrienoic acid-hydroxy nonadecanoic acid	FAHFA(20:3-O-19:0)	C39H70O4
524	ARAHNDA	Arachidonic acid-hydroxy nonadecanoic acid	FAHFA(20:4-O-19:0)	C39H68O4
525	EPAHNDA	Eicosapentaenoic acid-hydroxy nonadecanoic acid	FAHFA(20:5-O-19:0)	C39H66O4
526	BAHNDA	Behenic acid-hydroxy nonadecanoic acid	FAHFA(22:0-O-19:0)	C41H80O4
527	DEAHNDA	Docosenoic acid-hydroxy nonadecanoic acid	FAHFA(22:1-O-19:0)	C41H78O4
528	DDAHNDA	Docosadienoate-hydroxy nonadecanoic acid	FAHFA(22:2-O-19:0)	C41H76O4

529	DHAHNDA	Docosahexaenoic acid-hydroxy nonadecanoic acid	FAHFA(22:6-O-19:0)	C41H68O4
530	MAHAA	Myristic acid-hydroxy arachidic acid	FAHFA(14:0-O-20:0)	C34H66O4
531	MOHAA	Myristoleic acid-hydroxy arachidic acid	FAHFA(14:1-O-20:0)	C34H64O4
532	PDAHAA	Pentadecanoic acid-hydroxy arachidic acid	FAHFA(15:0-O-20:0)	C35H68O4
533	PDEAHAA	Pentadecenoic acid-hydroxy arachidic acid	FAHFA(15:1-O-20:0)	C35H66O4
534	POHAA	Palmitoleic acid-hydroxy arachidic acid	FAHFA(16:1-O-20:0)	C36H68O4
535	PAHAA	Palmitic acid-hydroxy arachidic acid	FAHFA(16:0-O-20:0)	C36H70O4
536	HDAHAA	Heptadecanoic acid-hydroxy arachidic acid	FAHFA(17:0-O-20:0)	C37H72O4
537	HDEAHAA	Heptadecenoic acid-hydroxy arachidic acid	FAHFA(17:1-O-20:0)	C37H70O4
538	SAHAA	Stearic acid-hydroxy arachidic acid	FAHFA(18:0-O-20:0)	C38H74O4
539	OAHAA	Oleic acid-hydroxy arachidic acid	FAHFA(18:1-O-20:0)	C38H72O4
540	LAHAA	Linoleic acid-hydroxy arachidic acid	FAHFA(18:2-O-20:0)	C38H70O4
541	ALAHAA	α-Linolenic acid-hydroxy arachidic acid	FAHFA(18:3-O-20:0)	C38H68O4
542	SDAHAA	Stearidonic acid-hydroxy arachidic acid	FAHFA(18:4-O-20:0)	C38H66O4
543	AAHAA	Arachidic acid-hydroxy arachidic acid	FAHFA(20:0-O-20:0)	C40H78O4
544	EAHAA	Eicosenoic acid-hydroxy arachidic acid	FAHFA(20:1-O-20:0)	C40H76O4
545	EDAHAA	Eicosadienoic acid-hydroxy arachidic acid	FAHFA(20:2-O-20:0)	C40H74O4
546	ETAHAA	Eicosatrienoic acid-hydroxy arachidic acid	FAHFA(20:3-O-20:0)	C40H72O4
547	ARAHAA	Arachidonic acid-hydroxy arachidic acid	FAHFA(20:4-O-20:0)	C40H70O4
548	EPAHAA	Eicosapentaenoic acid-hydroxy arachidic acid	FAHFA(20:5-O-20:0)	C40H68O4
549	BAHAA	Behenic acid-hydroxy arachidic acid	FAHFA(22:0-O-20:0)	C42H82O4
550	DEAHAA	Docosenoic acid-hydroxy arachidic acid	FAHFA(22:1-O-20:0)	C42H80O4
551	DDAHAA	Docosadienoate-hydroxy arachidic acid	FAHFA(22:2-O-20:0)	C42H78O4
552	DHAHAA	Docosahexaenoic acid-hydroxy arachidic acid	FAHFA(22:6-O-20:0)	C42H70O4
553	MAHHEA	Myristic acid-hydroxy heneicosanoic acid	FAHFA(14:0-O-21:0)	C35H68O4
554	MOHHEA	Myristoleic acid-hydroxy heneicosanoic acid	FAHFA(14:1-O-21:0)	C35H66O4
555	PDAHHEA	Pentadecanoic acid-hydroxy heneicosanoic acid	FAHFA(15:0-O-21:0)	C36H70O4
556	PDEAHHEA	Pentadecenoic acid-hydroxy heneicosanoic acid	FAHFA(15:1-O-21:0)	C36H68O4
557	POHHEA	Palmitoleic acid-hydroxy heneicosanoic acid	FAHFA(16:1-O-21:0)	C37H70O4
558	PAHHEA	Palmitic acid-hydroxy heneicosanoic acid	FAHFA(16:0-O-21:0)	C37H72O4
559	HDAHHEA	Heptadecanoic acid-hydroxy heneicosanoic acid	FAHFA(17:0-O-21:0)	C38H74O4
560	HDEAHHEA	Heptadecenoic acid-hydroxy heneicosanoic acid	FAHFA(17:1-O-21:0)	C38H72O4
561	SAHHEA	Stearic acid-hydroxy heneicosanoic acid	FAHFA(18:0-O-21:0)	C39H76O4
562	OAHHEA	Oleic acid-hydroxy heneicosanoic acid	FAHFA(18:1-O-21:0)	C39H74O4
563	LAHHEA	Linoleic acid-hydroxy heneicosanoic acid	FAHFA(18:2-O-21:0)	C39H72O4
564	ALAHHEA	α-Linolenic acid-hydroxy heneicosanoic acid	FAHFA(18:3-O-21:0)	C39H70O4
565	SDAHHEA	Stearidonic acid-hydroxy heneicosanoic acid	FAHFA(18:4-O-21:0)	C39H68O4
566	AAHHEA	Arachidic acid-hydroxy heneicosanoic acid	FAHFA(21:0-O-21:0)	C41H80O4
567	EAHHEA	Eicosenoic acid-hydroxy heneicosanoic acid	FAHFA(20:1-O-21:0)	C41H78O4
568	EDAHHEA	Eicosadienoic acid-hydroxy heneicosanoic acid	FAHFA(20:2-O-21:0)	C41H76O4
569	ETAHHEA	Eicosatrienoic acid-hydroxy heneicosanoic acid	FAHFA(20:3-O-21:0)	C41H74O4
570	ARAHHEA	Arachidonic acid-hydroxy heneicosanoic acid	FAHFA(20:4-O-21:0)	C41H72O4
571	EPAHHEA	Eicosapentaenoic acid-hydroxy heneicosanoic acid	FAHFA(20:5-O-21:0)	C41H70O4
572	BAHHEA	Behenic acid-hydroxy heneicosanoic acid	FAHFA(22:0-O-21:0)	C43H84O4
573	DEAHHEA	Docosenoic acid-hydroxy heneicosanoic acid	FAHFA(22:1-O-21:0)	C43H82O4
574	DDAHHEA	Docosadienoate-hydroxy heneicosanoic acid	FAHFA(22:2-O-21:0)	C43H80O4
575	DHAHHEA	Docosahexaenoic acid-hydroxy heneicosanoic acid	FAHFA(22:6-O-21:0)	C43H72O4
