# Supplementary material for: A dataset of branched fatty acid esters of hydroxy fatty acids diversity in foods
Source: Sci Data. 2023 Nov 10;10:790. doi: 10.1038/s41597-023-02712-z (PMC10638281; doi:10.1038/s41597-023-02712-z)
Supplement: Supplementary file 2 — Supplementary information-3 Table S3. MRM transitions list [file 41597_2023_2712_MOESM2_ESM.pdf]

Supplementary Table S3. MRM transitions of predicted FAHFAs in positive ion mode

Event	*m/z* (presursor ion)	*m/z* (product ion)	Ionization mode	Collision energy	Predicted FAHFA structure			
					FAHFA ID	Full name	Normal name	Molecular formula
1	523.5	250.2	(+)	35	MAHMO	Myristic acid-hydroxy myristoleic acid	FAHFA(14:0-O-14:1)	C28H52O4
2	521.5	250.2	(+)	35	MOHMO	Myristoleic acid-hydroxy myristoleic acid	FAHFA(14:1-O-14:1)	C28H50O4
3	537.5	250.2	(+)	35	PDAHMO	Pentadecanoic acid-hydroxy myristoleic acid	FAHFA(15:0-O-14:1)	C29H54O4
4	535.5	250.2	(+)	35	PDEAHMO	Pentadecenoic acid-hydroxy myristoleic acid	FAHFA(15:1-O-14:1)	C29H52O4
5	549.5	250.2	(+)	35	POHMO	Palmitoleic acid-hydroxy myristoleic acid	FAHFA(16:1-O-14:1)	C30H54O4
6	551.5	250.2	(+)	35	PAHMO	Palmitic acid-hydroxy myristoleic acid	FAHFA(16:0-O-14:1)	C30H56O4
7	565.5	250.2	(+)	35	HDAHMO	Heptadecanoic acid-hydroxy myristoleic acid	FAHFA(17:0-O-14:1)	C31H58O4
8	563.5	250.2	(+)	35	HDEAHMO	Heptadecenoic acid-hydroxy myristoleic acid	FAHFA(17:1-O-14:1)	C31H56O4
9	579.5	250.2	(+)	35	SAHMO	Stearic acid-hydroxy myristoleic acid	FAHFA(18:0-O-14:1)	C32H60O4
10	577.5	250.2	(+)	35	OAHMO	Oleic acid-hydroxy myristoleic acid	FAHFA(18:1-O-14:1)	C32H58O4
11	575.5	250.2	(+)	35	LAHMO	Linoleic acid-hydroxy myristoleic acid	FAHFA(18:2-O-14:1)	C32H56O4
12	573.5	250.2	(+)	35	ALAHMO	α-Linolenic acid-hydroxy myristoleic acid	FAHFA(18:3-O-14:1)	C32H54O4
13	571.5	250.2	(+)	35	SDAHMO	Stearidonic acid-hydroxy myristoleic acid	FAHFA(18:4-O-14:1)	C32H52O4
14	607.5	250.2	(+)	35	AAHMO	Arachidic acid-hydroxy myristoleic acid	FAHFA(20:0-O-14:1)	C34H64O4
15	605.5	250.2	(+)	35	EAHMO	Eicosenoic acid-hydroxy myristoleic acid	FAHFA(20:1-O-14:1)	C34H62O4
16	603.5	250.2	(+)	35	EDAHMO	Eicosadienoic acid-hydroxy myristoleic acid	FAHFA(20:2-O-14:1)	C34H60O4
17	601.5	250.2	(+)	35	ETAHMO	Eicosatrienoic acid-hydroxy myristoleic acid	FAHFA(20:3-O-14:1)	C34H58O4
18	599.5	250.2	(+)	35	ARAHMO	Arachidonic acid-hydroxy myristoleic acid	FAHFA(20:4-O-14:1)	C34H56O4
19	597.5	250.2	(+)	35	EPAHMO	Eicosapentaenoic acid-hydroxy myristoleic acid	FAHFA(20:5-O-14:1)	C34H54O4
20	635.5	250.2	(+)	35	BAHMO	Behenic acid-hydroxy myristoleic acid	FAHFA(22:0-O-14:1)	C36H68O4
21	633.5	250.2	(+)	35	DEAHMO	Docosenoic acid-hydroxy myristoleic acid	FAHFA(22:1-O-14:1)	C36H66O4
22	631.5	250.2	(+)	35	DDAHMO	Docosadienoate-hydroxy myristoleic acid	FAHFA(22:2-O-14:1)	C36H64O4
23	623.5	250.2	(+)	35	DHAHMO	Docosahexaenoic acid-hydroxy myristoleic acid	FAHFA(22:6-O-14:1)	C36H56O4
24	521.5	248.2	(+)	35	MAHTDA	Myristic acid-hydroxy tetradecadienoic acid	FAHFA(14:0-O-14:2)	C28H50O4
25	519.5	248.2	(+)	35	MOHTDA	Myristoleic acid-hydroxy tetradecadienoic acid	FAHFA(14:1-O-14:2)	C28H48O4
26	535.5	248.2	(+)	35	PDAHTDA	Pentadecanoic acid-hydroxy tetradecadienoic acid	FAHFA(15:0-O-14:2)	C29H52O4
27	533.5	248.2	(+)	35	PDEAHTDA	Pentadecenoic acid-hydroxy tetradecadienoic acid	FAHFA(15:1-O-14:2)	C29H50O4
28	547.5	248.2	(+)	35	POHTDA	Palmitoleic acid-hydroxy tetradecadienoic acid	FAHFA(16:1-O-14:2)	C30H52O4
29	549.5	248.2	(+)	35	PAHTDA	Palmitic acid-hydroxy tetradecadienoic acid	FAHFA(16:0-O-14:2)	C30H54O4
30	563.5	248.2	(+)	35	HDAHTDA	Heptadecanoic acid-hydroxy tetradecadienoic acid	FAHFA(17:0-O-14:2)	C31H56O4
31	561.5	248.2	(+)	35	HDEAHTDA	Heptadecenoic acid-hydroxy tetradecadienoic acid	FAHFA(17:1-O-14:2)	C31H54O4
32	577.5	248.2	(+)	35	SAHTDA	Stearic acid-hydroxy tetradecadienoic acid	FAHFA(18:0-O-14:2)	C32H58O4
33	575.5	248.2	(+)	35	OAHTDA	Oleic acid-hydroxy tetradecadienoic acid	FAHFA(18:1-O-14:2)	C32H56O4
34	573.5	248.2	(+)	35	LAHTDA	Linoleic acid-hydroxy tetradecadienoic acid	FAHFA(18:2-O-14:2)	C32H54O4
35	571.5	248.2	(+)	35	ALAHTDA	α-Linolenic acid-hydroxy tetradecadienoic acid	FAHFA(18:3-O-14:2)	C32H52O4
36	569.5	248.2	(+)	35	SDAHTDA	Stearidonic acid-hydroxy tetradecadienoic acid	FAHFA(18:4-O-14:2)	C32H50O4
37	605.5	248.2	(+)	35	AAHTDA	Arachidic acid-hydroxy tetradecadienoic acid	FAHFA(20:0-O-14:2)	C34H62O4
38	603.5	248.2	(+)	35	EAHTDA	Eicosenoic acid-hydroxy tetradecadienoic acid	FAHFA(20:1-O-14:2)	C34H60O4
39	601.5	248.2	(+)	35	EDAHTDA	Eicosadienoic acid-hydroxy tetradecadienoic acid	FAHFA(20:2-O-14:2)	C34H58O4
40	599.5	248.2	(+)	35	ETAHTDA	Eicosatrienoic acid-hydroxy tetradecadienoic acid	FAHFA(20:3-O-14:2)	C34H56O4
41	597.5	248.2	(+)	35	ARAHTDA	Arachidonic acid-hydroxy tetradecadienoic acid	FAHFA(20:4-O-14:2)	C34H54O4
42	595.5	248.2	(+)	35	EPAHTDA	Eicosapentaenoic acid-hydroxy tetradecadienoic acid	FAHFA(20:5-O-14:2)	C34H52O4
43	633.5	248.2	(+)	35	BAHTDA	Behenic acid-hydroxy tetradecadienoic acid	FAHFA(22:0-O-14:2)	C36H66O4
44	631.5	248.2	(+)	35	DEAHTDA	Docosenoic acid-hydroxy tetradecadienoic acid	FAHFA(22:1-O-14:2)	C36H64O4
45	629.5	248.2	(+)	35	DDAHTDA	Docosadienoate-hydroxy tetradecadienoic acid	FAHFA(22:2-O-14:2)	C36H62O4
46	621.5	248.2	(+)	35	DHAHTDA	Docosahexaenoic acid-hydroxy tetradecadienoic acid	FAHFA(22:6-O-14:2)	C36H54O4
47	519.5	246.2	(+)	35	MAHTTA	Myristic acid-hydroxy tetradecatrienoic acid	FAHFA(14:0-O-14:3)	C28H48O4
48	517.5	246.2	(+)	35	MOHTTA	Myristoleic acid-hydroxy tetradecatrienoic acid	FAHFA(14:1-O-14:3)	C28H46O4
49	533.5	246.2	(+)	35	PDAHTTA	Pentadecanoic acid-hydroxy tetradecatrienoic acid	FAHFA(15:0-O-14:3)	C29H50O4
50	531.5	246.2	(+)	35	PDEAHTTA	Pentadecenoic acid-hydroxy tetradecatrienoic acid	FAHFA(15:1-O-14:3)	C29H48O4
51	545.5	246.2	(+)	35	POHTTA	Palmitoleic acid-hydroxy tetradecatrienoic acid	FAHFA(16:1-O-14:3)	C30H50O4
52	547.5	246.2	(+)	35	PAHTTA	Palmitic acid-hydroxy tetradecatrienoic acid	FAHFA(16:0-O-14:3)	C30H52O4
53	561.5	246.2	(+)	35	HDAHTTA	Heptadecanoic acid-hydroxy tetradecatrienoic acid	FAHFA(17:0-O-14:3)	C31H54O4
54	559.5	246.2	(+)	35	HDEAHTTA	Heptadecenoic acid-hydroxy tetradecatrienoic acid	FAHFA(17:1-O-14:3)	C31H52O4
55	575.5	246.2	(+)	35	SAHTTA	Stearic acid-hydroxy tetradecatrienoic acid	FAHFA(18:0-O-14:3)	C32H56O4
56	573.5	246.2	(+)	35	OAHTTA	Oleic acid-hydroxy tetradecatrienoic acid	FAHFA(18:1-O-14:3)	C32H54O4
57	571.5	246.2	(+)	35	LAHTTA	Linoleic acid-hydroxy tetradecatrienoic acid	FAHFA(18:2-O-14:3)	C32H52O4
58	569.5	246.2	(+)	35	ALAHTTA	α-Linolenic acid-hydroxy tetradecatrienoic acid	FAHFA(18:3-O-14:3)	C32H50O4
59	567.5	246.2	(+)	35	SDAHTTA	Stearidonic acid-hydroxy tetradecatrienoic acid	FAHFA(18:4-O-14:3)	C32H48O4
60	603.5	246.2	(+)	35	AAHTTA	Arachidic acid-hydroxy tetradecatrienoic acid	FAHFA(20:0-O-14:3)	C34H60O4
61	601.5	246.2	(+)	35	EAHTTA	Eicosenoic acid-hydroxy tetradecatrienoic acid	FAHFA(20:1-O-14:3)	C34H58O4
62	599.5	246.2	(+)	35	EDAHTTA	Eicosadienoic acid-hydroxy tetradecatrienoic acid	FAHFA(20:2-O-14:3)	C34H56O4
63	597.5	246.2	(+)	35	ETAHTTA	Eicosatrienoic acid-hydroxy tetradecatrienoic acid	FAHFA(20:3-O-14:3)	C34H54O4
64	595.5	246.2	(+)	35	ARAHTTA	Arachidonic acid-hydroxy tetradecatrienoic acid	FAHFA(20:4-O-14:3)	C34H52O4
65	593.5	246.2	(+)	35	EPAHTTA	Eicosapentaenoic acid-hydroxy tetradecatrienoic acid	FAHFA(20:5-O-14:3)	C34H50O4
66	631.5	246.2	(+)	35	BAHTTA	Behenic acid-hydroxy tetradecatrienoic acid	FAHFA(22:0-O-14:3)	C36H64O4
67	629.5	246.2	(+)	35	DEAHTTA	Docosenoic acid-hydroxy tetradecatrienoic acid	FAHFA(22:1-O-14:3)	C36H62O4
68	627.5	246.2	(+)	35	DDAHTTA	Docosadienoate-hydroxy tetradecatrienoic acid	FAHFA(22:2-O-14:3)	C36H60O4
69	619.5	246.2	(+)	35	DHAHTTA	Docosahexaenoic acid-hydroxy tetradecatrienoic acid	FAHFA(22:6-O-14:3)	C36H52O4
70	551.5	278.2	(+)	35	MAHPO	Myristic acid-hydroxy palmitoleic acid	FAHFA(14:0-O-16:1)	C30H56O4
71	549.5	278.2	(+)	35	MOHPO	Myristoleic acid-hydroxy palmitoleic acid	FAHFA(14:1-O-16:1)	C30H54O4
72	565.5	278.2	(+)	35	PDAHPO	Pentadecanoic acid-hydroxy palmitoleic acid	FAHFA(15:0-O-16:1)	C31H58O4
73	563.5	278.2	(+)	35	PDEAHPO	Pentadecenoic acid-hydroxy palmitoleic acid	FAHFA(15:1-O-16:1)	C31H56O4
74	577.5	278.2	(+)	35	POHPO	Palmitoleic acid-hydroxy palmitoleic acid	FAHFA(16:1-O-16:1)	C32H58O4
75	579.5	278.2	(+)	35	PAHPO	Palmitic acid-hydroxy palmitoleic acid	FAHFA(16:0-O-16:1)	C32H60O4
76	593.5	278.2	(+)	35	HDAHPO	Heptadecanoic acid-hydroxy palmitoleic acid	FAHFA(17:0-O-16:1)	C33H62O4
77	591.5	278.2	(+)	35	HDEAHPO	Heptadecenoic acid-hydroxy palmitoleic acid	FAHFA(17:1-O-16:1)	C33H60O4
78	607.5	278.2	(+)	35	SAHPO	Stearic acid-hydroxy palmitoleic acid	FAHFA(18:0-O-16:1)	C34H64O4
79	605.5	278.2	(+)	35	OAHPO	Oleic acid-hydroxy palmitoleic acid	FAHFA(18:1-O-16:1)	C34H62O4
80	603.5	278.2	(+)	35	LAHPO	Linoleic acid-hydroxy palmitoleic acid	FAHFA(18:2-O-16:1)	C34H60O4
81	601.5	278.2	(+)	35	ALAHPO	α-Linolenic acid-hydroxy palmitoleic acid	FAHFA(18:3-O-16:1)	C34H58O4
82	599.5	278.2	(+)	35	SDAHPO	Stearidonic acid-hydroxy palmitoleic acid	FAHFA(18:4-O-16:1)	C34H56O4
83	635.5	278.2	(+)	35	AAHPO	Arachidic acid-hydroxy palmitoleic acid	FAHFA(20:0-O-16:1)	C36H68O4
84	633.5	278.2	(+)	35	EAHPO	Eicosenoic acid-hydroxy palmitoleic acid	FAHFA(20:1-O-16:1)	C36H66O4
85	631.5	278.2	(+)	35	EDAHPO	Eicosadienoic acid-hydroxy palmitoleic acid	FAHFA(20:2-O-16:1)	C36H64O4
86	629.5	278.2	(+)	35	ETAHPO	Eicosatrienoic acid-hydroxy palmitoleic acid	FAHFA(20:3-O-16:1)	C36H62O4
87	627.5	278.2	(+)	35	ARAHPO	Arachidonic acid-hydroxy palmitoleic acid	FAHFA(20:4-O-16:1)	C36H60O4
88	625.5	278.2	(+)	35	EPAHPO	Eicosapentaenoic acid-hydroxy palmitoleic acid	FAHFA(20:5-O-16:1)	C36H58O4
89	663.5	278.2	(+)	35	BAHPO	Behenic acid-hydroxy palmitoleic acid	FAHFA(22:0-O-16:1)	C38H72O4
90	661.5	278.2	(+)	35	DEAHPO	Docosenoic acid-hydroxy palmitoleic acid	FAHFA(22:1-O-16:1)	C38H70O4
91	659.5	278.2	(+)	35	DDAHPO	Docosadienoate-hydroxy palmitoleic acid	FAHFA(22:2-O-16:1)	C38H68O4
92	651.4	278.2	(+)	35	DHAHPO	Docosahexaenoic acid-hydroxy palmitoleic acid	FAHFA(22:6-O-16:1)	C38H60O4
93	549.5	276.2	(+)	35	MAHHDDA	Myristic acid-hydroxy hexadecadienoic acid	FAHFA(14:0-O-16:2)	C30H54O4
94	547.5	276.2	(+)	35	MOHHDDA	Myristoleic acid-hydroxy hexadecadienoic acid	FAHFA(14:1-O-16:2)	C30H52O4
95	563.5	276.2	(+)	35	PDAHHDDA	Pentadecanoic acid-hydroxy hexadecadienoic acid	FAHFA(15:0-O-16:2)	C31H56O4
96	561.5	276.2	(+)	35	PDEAHHDDA	Pentadecenoic acid-hydroxy hexadecadienoic acid	FAHFA(15:1-O-16:2)	C31H54O4
97	575.5	276.2	(+)	35	POHHDDA	Palmitoleic acid-hydroxy hexadecadienoic acid	FAHFA(16:1-O-16:2)	C32H56O4
98	577.5	276.2	(+)	35	PAHHDDA	Palmitic acid-hydroxy hexadecadienoic acid	FAHFA(16:0-O-16:2)	C32H58O4
99	591.5	276.2	(+)	35	HDAHHDDA	Heptadecanoic acid-hydroxy hexadecadienoic acid	FAHFA(17:0-O-16:2)	C33H60O4

100	589.5	276.2	(+)	35	HDEAHHDDA	Heptadecenoic acid-hydroxy hexadecadienoic acid	FAHFA(17:1-O-16:2)	C33H58O4
101	605.5	276.2	(+)	35	SAHHDDA	Stearic acid-hydroxy hexadecadienoic acid	FAHFA(18:0-O-16:2)	C34H62O4
102	603.5	276.2	(+)	35	OAHHDDA	Oleic acid-hydroxy hexadecadienoic acid	FAHFA(18:1-O-16:2)	C34H60O4
103	601.5	276.2	(+)	35	LAHHDDA	Linoleic acid-hydroxy hexadecadienoic acid	FAHFA(18:2-O-16:2)	C34H58O4
104	599.5	276.2	(+)	35	ALAHHDDA	α-Linolenic acid-hydroxy hexadecadienoic acid	FAHFA(18:3-O-16:2)	C34H56O4
105	597.5	276.2	(+)	35	SDAHHDDA	Stearidonic acid-hydroxy hexadecadienoic acid	FAHFA(18:4-O-16:2)	C34H54O4
106	633.5	276.2	(+)	35	AAHHDDA	Arachidic acid-hydroxy hexadecadienoic acid	FAHFA(20:0-O-16:2)	C36H66O4
107	631.5	276.2	(+)	35	EAHHDDA	Eicosenoic acid-hydroxy hexadecadienoic acid	FAHFA(20:1-O-16:2)	C36H64O4
108	629.5	276.2	(+)	35	EDAHHDDA	Eicosadienoic acid-hydroxy hexadecadienoic acid	FAHFA(20:2-O-16:2)	C36H62O4
109	627.5	276.2	(+)	35	ETAHHDDA	Eicosatrienoic acid-hydroxy hexadecadienoic acid	FAHFA(20:3-O-16:2)	C36H60O4
110	625.5	276.2	(+)	35	ARAHHDDA	Arachidonic acid-hydroxy hexadecadienoic acid	FAHFA(20:4-O-16:2)	C36H58O4
111	623.5	276.2	(+)	35	EPAHHDDA	Eicosapentaenoic acid-hydroxy hexadecadienoic acid	FAHFA(20:5-O-16:2)	C36H56O4
112	661.5	276.2	(+)	35	BAHHDDA	Behenic acid-hydroxy hexadecadienoic acid	FAHFA(22:0-O-16:2)	C38H70O4
113	659.5	276.2	(+)	35	DEAHHDDA	Docosenoic acid-hydroxy hexadecadienoic acid	FAHFA(22:1-O-16:2)	C38H68O4
114	657.5	276.2	(+)	35	DDAHHDDA	Docosadienoate-hydroxy hexadecadienoic acid	FAHFA(22:2-O-16:2)	C38H66O4
115	649.5	276.2	(+)	35	DHAHHDDA	Docosahexaenoic acid-hydroxy hexadecadienoic acid	FAHFA(22:6-O-16:2)	C38H58O4
116	547.5	274.2	(+)	35	MAHHTA	Myristic acid-hydroxy hexadecatrienoic acid	FAHFA(14:0-O-16:3)	C30H52O4
117	545.5	274.2	(+)	35	MOHHTA	Myristoleic acid-hydroxy hexadecatrienoic acid	FAHFA(14:1-O-16:3)	C30H50O4
118	561.5	274.2	(+)	35	PDAHHTA	Pentadecanoic acid-hydroxy hexadecatrienoic acid	FAHFA(15:0-O-16:3)	C31H54O4
119	559.5	274.2	(+)	35	PDEAHHTA	Pentadecenoic acid-hydroxy hexadecatrienoic acid	FAHFA(15:1-O-16:3)	C31H52O4
120	573.5	274.2	(+)	35	POHHTA	Palmitoleic acid-hydroxy hexadecatrienoic acid	FAHFA(16:1-O-16:3)	C32H54O4
121	575.5	274.2	(+)	35	PAHHTA	Palmitic acid-hydroxy hexadecatrienoic acid	FAHFA(16:0-O-16:3)	C32H56O4
122	589.5	274.2	(+)	35	HDAHHTA	Heptadecanoic acid-hydroxy hexadecatrienoic acid	FAHFA(17:0-O-16:3)	C33H58O4
123	587.5	274.2	(+)	35	HDEAHHTA	Heptadecenoic acid-hydroxy hexadecatrienoic acid	FAHFA(17:1-O-16:3)	C33H56O4
124	603.5	274.2	(+)	35	SAHHTA	Stearic acid-hydroxy hexadecatrienoic acid	FAHFA(18:0-O-16:3)	C34H60O4
125	601.5	274.2	(+)	35	OAHHTA	Oleic acid-hydroxy hexadecatrienoic acid	FAHFA(18:1-O-16:3)	C34H58O4
126	599.5	274.2	(+)	35	LAHHTA	Linoleic acid-hydroxy hexadecatrienoic acid	FAHFA(18:2-O-16:3)	C34H56O4
127	597.5	274.2	(+)	35	ALAHHTA	α-Linolenic acid-hydroxy hexadecatrienoic acid	FAHFA(18:3-O-16:3)	C34H54O4
128	595.5	274.2	(+)	35	SDAHHTA	Stearidonic acid-hydroxy hexadecatrienoic acid	FAHFA(18:4-O-16:3)	C34H52O4
129	631.5	274.2	(+)	35	AAHHTA	Arachidic acid-hydroxy hexadecatrienoic acid	FAHFA(20:0-O-16:3)	C36H64O4
130	629.5	274.2	(+)	35	EAHHTA	Eicosenoic acid-hydroxy hexadecatrienoic acid	FAHFA(20:1-O-16:3)	C36H62O4
131	627.5	274.2	(+)	35	EDAHHTA	Eicosadienoic acid-hydroxy hexadecatrienoic acid	FAHFA(20:2-O-16:3)	C36H60O4
132	625.5	274.2	(+)	35	ETAHHTA	Eicosatrienoic acid-hydroxy hexadecatrienoic acid	FAHFA(20:3-O-16:3)	C36H58O4
133	623.5	274.2	(+)	35	ARAHHTA	Arachidonic acid-hydroxy hexadecatrienoic acid	FAHFA(20:4-O-16:3)	C36H56O4
134	621.5	274.2	(+)	35	EPAHHTA	Eicosapentaenoic acid-hydroxy hexadecatrienoic acid	FAHFA(20:5-O-16:3)	C36H54O4
135	659.5	274.2	(+)	35	BAHHTA	Behenic acid-hydroxy hexadecatrienoic acid	FAHFA(22:0-O-16:3)	C38H68O4
136	657.5	274.2	(+)	35	DEAHHTA	Docosenoic acid-hydroxy hexadecatrienoic acid	FAHFA(22:1-O-16:3)	C38H66O4
137	655.5	274.2	(+)	35	DDAHHTA	Docosadienoate-hydroxy hexadecatrienoic acid	FAHFA(22:2-O-16:3)	C38H64O4
138	647.5	274.2	(+)	35	DHAHHTA	Docosahexaenoic acid-hydroxy hexadecatrienoic acid	FAHFA(22:6-O-16:3)	C38H56O4
139	579.5	306.3	(+)	35	MAHOA	Myristic acid-hydroxy oleic acid	FAHFA(14:0-O-18:1)	C32H60O4
140	577.5	306.3	(+)	35	MOHOA	Myristoleic acid-hydroxy oleic acid	FAHFA(14:1-O-18:1)	C32H58O4
141	593.5	306.3	(+)	35	PDAHOA	Pentadecanoic acid-hydroxy oleic acid	FAHFA(15:0-O-18:1)	C33H62O4
142	591.5	306.3	(+)	35	PDEAHOA	Pentadecenoic acid-hydroxy oleic acid	FAHFA(15:1-O-18:1)	C33H60O4
143	605.5	306.3	(+)	35	POHOA	Palmitoleic acid-hydroxy oleic acid	FAHFA(16:1-O-18:1)	C34H62O4
144	607.5	306.3	(+)	35	PAHOA	Palmitic acid-hydroxy oleic acid	FAHFA(16:0-O-18:1)	C34H64O4
145	621.5	306.3	(+)	35	HDAHOA	Heptadecanoic acid-hydroxy oleic acid	FAHFA(17:0-O-18:1)	C35H66O4
146	619.5	306.3	(+)	35	HDEAHOA	Heptadecenoic acid-hydroxy oleic acid	FAHFA(17:1-O-18:1)	C35H64O4
147	635.5	306.3	(+)	35	SAHOA	Stearic acid-hydroxy oleic acid	FAHFA(18:0-O-18:1)	C36H68O4
148	633.5	306.3	(+)	35	OAHOA	Oleic acid-hydroxy oleic acid	FAHFA(18:1-O-18:1)	C36H66O4
149	631.5	306.3	(+)	35	LAHOA	Linoleic acid-hydroxy oleic acid	FAHFA(18:2-O-18:1)	C36H64O4
150	629.5	306.3	(+)	35	ALAHOA	α-Linolenic acid-hydroxy oleic acid	FAHFA(18:3-O-18:1)	C36H62O4
151	627.5	306.3	(+)	35	SDAHOA	Stearidonic acid-hydroxy oleic acid	FAHFA(18:4-O-18:1)	C36H60O4
152	663.5	306.3	(+)	35	AAHOA	Arachidic acid-hydroxy oleic acid	FAHFA(20:0-O-18:1)	C38H72O4
153	661.5	306.3	(+)	35	EAHOA	Eicosenoic acid-hydroxy oleic acid	FAHFA(20:1-O-18:1)	C38H70O4
154	659.5	306.3	(+)	35	EDAHOA	Eicosadienoic acid-hydroxy oleic acid	FAHFA(20:2-O-18:1)	C38H68O4
155	657.5	306.3	(+)	35	ETAHOA	Eicosatrienoic acid-hydroxy oleic acid	FAHFA(20:3-O-18:1)	C38H66O4
156	655.5	306.3	(+)	35	ARAHOA	Arachidonic acid-hydroxy oleic acid	FAHFA(20:4-O-18:1)	C38H64O4
157	653.5	306.3	(+)	35	EPAHOA	Eicosapentaenoic acid-hydroxy oleic acid	FAHFA(20:5-O-18:1)	C38H62O4
158	691.5	306.3	(+)	35	BAHOA	Behenic acid-hydroxy oleic acid	FAHFA(22:0-O-18:1)	C40H76O4
159	689.5	306.3	(+)	35	DEAHOA	Docosenoic acid-hydroxy oleic acid	FAHFA(22:1-O-18:1)	C40H74O4
160	687.5	306.3	(+)	35	DDAHOA	Docosadienoate-hydroxy oleic acid	FAHFA(22:2-O-18:1)	C40H72O4
161	679.5	306.3	(+)	35	DHAHOA	Docosahexaenoic acid-hydroxy oleic acid	FAHFA(22:6-O-18:1)	C40H64O4
162	577.5	304.3	(+)	35	MAHLA	Myristic acid-hydroxy linoleic acid	FAHFA(14:0-O-18:2)	C32H58O4
163	575.5	304.3	(+)	35	MOHLA	Myristoleic acid-hydroxy linoleic acid	FAHFA(14:1-O-18:2)	C32H56O4
164	591.5	304.3	(+)	35	PDAHLA	Pentadecanoic acid-hydroxy linoleic acid	FAHFA(15:0-O-18:2)	C33H60O4
165	589.5	304.3	(+)	35	PDEAHLA	Pentadecenoic acid-hydroxy linoleic acid	FAHFA(15:1-O-18:2)	C33H58O4
166	603.5	304.3	(+)	35	POHLA	Palmitoleic acid-hydroxy linoleic acid	FAHFA(16:1-O-18:2)	C34H60O4
167	605.5	304.3	(+)	35	PAHLA	Palmitic acid-hydroxy linoleic acid	FAHFA(16:0-O-18:2)	C34H62O4
168	619.5	304.3	(+)	35	HDAHLA	Heptadecanoic acid-hydroxy linoleic acid	FAHFA(17:0-O-18:2)	C35H64O4
169	617.5	304.3	(+)	35	HDEAHLA	Heptadecenoic acid-hydroxy linoleic acid	FAHFA(17:1-O-18:2)	C35H62O4
170	633.5	304.3	(+)	35	SAHLA	Stearic acid-hydroxy linoleic acid	FAHFA(18:0-O-18:2)	C36H66O4
171	631.5	304.3	(+)	35	OAHLA	Oleic acid-hydroxy linoleic acid	FAHFA(18:1-O-18:2)	C36H64O4
172	629.5	304.3	(+)	35	LAHLA	Linoleic acid-hydroxy linoleic acid	FAHFA(18:2-O-18:2)	C36H62O4
173	627.5	304.3	(+)	35	ALAHLA	α-Linolenic acid-hydroxy linoleic acid	FAHFA(18:3-O-18:2)	C36H60O4
174	625.5	304.3	(+)	35	SDAHLA	Stearidonic acid-hydroxy linoleic acid	FAHFA(18:4-O-18:2)	C36H58O4
175	661.5	304.3	(+)	35	AAHLA	Arachidic acid-hydroxy linoleic acid	FAHFA(20:0-O-18:2)	C38H70O4
176	659.5	304.3	(+)	35	EAHLA	Eicosenoic acid-hydroxy linoleic acid	FAHFA(20:1-O-18:2)	C38H68O4
177	657.5	304.3	(+)	35	EDAHLA	Eicosadienoic acid-hydroxy linoleic acid	FAHFA(20:2-O-18:2)	C38H66O4
178	655.5	304.3	(+)	35	ETAHLA	Eicosatrienoic acid-hydroxy linoleic acid	FAHFA(20:3-O-18:2)	C38H64O4
179	653.5	304.3	(+)	35	ARAHLA	Arachidonic acid-hydroxy linoleic acid	FAHFA(20:4-O-18:2)	C38H62O4
180	651.5	304.3	(+)	35	EPAHLA	Eicosapentaenoic acid-hydroxy linoleic acid	FAHFA(20:5-O-18:2)	C38H60O4
181	689.5	304.3	(+)	35	BAHLA	Behenic acid-hydroxy linoleic acid	FAHFA(22:0-O-18:2)	C40H74O4
182	687.5	304.3	(+)	35	DEAHLA	Docosenoic acid-hydroxy linoleic acid	FAHFA(22:1-O-18:2)	C40H72O4
183	685.5	304.3	(+)	35	DDAHLA	Docosadienoate-hydroxy linoleic acid	FAHFA(22:2-O-18:2)	C40H70O4
184	677.5	304.3	(+)	35	DHAHLA	Docosahexaenoic acid-hydroxy linoleic acid	FAHFA(22:6-O-18:2)	C40H62O4
185	575.5	302.2	(+)	35	MAHALA	Myristic acid-hydroxy α-Linolenic acid	FAHFA(14:0-O-18:3)	C32H56O4
186	573.5	302.2	(+)	35	MOHALA	Myristoleic acid-hydroxy α-Linolenic acid	FAHFA(14:1-O-18:3)	C32H54O4
187	589.5	302.2	(+)	35	PDAHALA	Pentadecanoic acid-hydroxy α-Linolenic acid	FAHFA(15:0-O-18:3)	C33H58O4
188	587.5	302.2	(+)	35	PDEAHALA	Pentadecenoic acid-hydroxy α-Linolenic acid	FAHFA(15:1-O-18:3)	C33H56O4
189	601.5	302.2	(+)	35	POHALA	Palmitoleic acid-hydroxy α-Linolenic acid	FAHFA(16:1-O-18:3)	C34H58O4
190	603.5	302.2	(+)	35	PAHALA	Palmitic acid-hydroxy α-Linolenic acid	FAHFA(16:0-O-18:3)	C34H60O4
191	617.5	302.2	(+)	35	HDAHALA	Heptadecanoic acid-hydroxy α-Linolenic acid	FAHFA(17:0-O-18:3)	C35H62O4
192	615.5	302.2	(+)	35	HDEAHALA	Heptadecenoic acid-hydroxy α-Linolenic acid	FAHFA(17:1-O-18:3)	C35H60O4
193	631.5	302.2	(+)	35	SAHALA	Stearic acid-hydroxy α-Linolenic acid	FAHFA(18:0-O-18:3)	C36H64O4
194	629.5	302.2	(+)	35	OAHALA	Oleic acid-hydroxy α-Linolenic acid	FAHFA(18:1-O-18:3)	C36H62O4
195	627.5	302.2	(+)	35	LAHALA	Linoleic acid-hydroxy α-Linolenic acid	FAHFA(18:2-O-18:3)	C36H60O4
196	625.5	302.2	(+)	35	ALAHALA	α-Linolenic acid-hydroxy α-Linolenic acid	FAHFA(18:3-O-18:3)	C36H58O4
197	623.5	302.2	(+)	35	SDAHALA	Stearidonic acid-hydroxy α-Linolenic acid	FAHFA(18:4-O-18:3)	C36H56O4
198	659.5	302.2	(+)	35	AAHALA	Arachidic acid-hydroxy α-Linolenic acid	FAHFA(20:0-O-18:3)	C38H68O4
199	657.5	302.2	(+)	35	EAHALA	Eicosenoic acid-hydroxy α-Linolenic acid	FAHFA(20:1-O-18:3)	C38H66O4
200	655.5	302.2	(+)	35	EDAHALA	Eicosadienoic acid-hydroxy α-Linolenic acid	FAHFA(20:2-O-18:3)	C38H64O4
201	653.5	302.2	(+)	35	ETAHALA	Eicosatrienoic acid-hydroxy α-Linolenic acid	FAHFA(20:3-O-18:3)	C38H62O4
202	651.5	302.2	(+)	35	ARAHALA	Arachidonic acid-hydroxy α-Linolenic acid	FAHFA(20:4-O-18:3)	C38H60O4
203	649.5	302.2	(+)	35	EPAHALA	Eicosapentaenoic acid-hydroxy α-Linolenic acid	FAHFA(20:5-O-18:3)	C38H58O4
204	687.5	302.2	(+)	35	BAHALA	Behenic acid-hydroxy α-Linolenic acid	FAHFA(22:0-O-18:3)	C40H72O4

205	685.5	302.2	(+)	35	DEAHALA	Docosenoic acid-hydroxy α-Linolenic acid	FAHFA(22:1-O-18:3)	C40H70O4
206	683.5	302.2	(+)	35	DDAHALA	Docosadienoate-hydroxy α-Linolenic acid	FAHFA(22:2-O-18:3)	C40H68O4
207	675.5	302.2	(+)	35	DHAHALA	Docosahexaenoic acid-hydroxy α-Linolenic acid	FAHFA(22:6-O-18:3)	C40H60O4
208	607.5	334.3	(+)	35	MAHEA	Myristic acid-hydroxy eicosenoic acid	FAHFA(14:0-O-20:1)	C34H64O4
209	605.5	334.3	(+)	35	MOHEA	Myristoleic acid-hydroxy eicosenoic acid	FAHFA(14:1-O-20:1)	C34H62O4
210	621.5	334.3	(+)	35	PDAHEA	Pentadecanoic acid-hydroxy eicosenoic acid	FAHFA(15:0-O-20:1)	C35H66O4
211	619.5	334.3	(+)	35	PDEAHEA	Pentadecenoic acid-hydroxy eicosenoic acid	FAHFA(15:1-O-20:1)	C35H64O4
212	633.5	334.3	(+)	35	POHEA	Palmitoleic acid-hydroxy eicosenoic acid	FAHFA(16:1-O-20:1)	C36H66O4
213	635.5	334.3	(+)	35	PAHEA	Palmitic acid-hydroxy eicosenoic acid	FAHFA(16:0-O-20:1)	C36H68O4
214	649.5	334.3	(+)	35	HDAHEA	Heptadecanoic acid-hydroxy eicosenoic acid	FAHFA(17:0-O-20:1)	C37H70O4
215	647.5	334.3	(+)	35	HDEAHEA	Heptadecenoic acid-hydroxy eicosenoic acid	FAHFA(17:1-O-20:1)	C37H68O4
216	663.5	334.3	(+)	35	SAHEA	Stearic acid-hydroxy eicosenoic acid	FAHFA(18:0-O-20:1)	C38H72O4
217	661.5	334.3	(+)	35	OAHEA	Oleic acid-hydroxy eicosenoic acid	FAHFA(18:1-O-20:1)	C38H70O4
218	659.5	334.3	(+)	35	LAHEA	Linoleic acid-hydroxy eicosenoic acid	FAHFA(18:2-O-20:1)	C38H68O4
219	657.5	334.3	(+)	35	ALAHEA	α-Linolenic acid-hydroxy eicosenoic acid	FAHFA(18:3-O-20:1)	C38H66O4
220	655.5	334.3	(+)	35	SDAHEA	Stearidonic acid-hydroxy eicosenoic acid	FAHFA(18:4-O-20:1)	C38H64O4
221	691.5	334.3	(+)	35	AAHEA	Arachidic acid-hydroxy eicosenoic acid	FAHFA(20:0-O-20:1)	C40H76O4
222	689.5	334.3	(+)	35	EAHEA	Eicosenoic acid-hydroxy eicosenoic acid	FAHFA(20:1-O-20:1)	C40H74O4
223	687.5	334.3	(+)	35	EDAHEA	Eicosadienoic acid-hydroxy eicosenoic acid	FAHFA(20:2-O-20:1)	C40H72O4
224	685.5	334.3	(+)	35	ETAHEA	Eicosatrienoic acid-hydroxy eicosenoic acid	FAHFA(20:3-O-20:1)	C40H70O4
225	683.5	334.3	(+)	35	ARAHEA	Arachidonic acid-hydroxy eicosenoic acid	FAHFA(20:4-O-20:1)	C40H68O4
226	681.5	334.3	(+)	35	EPAHEA	Eicosapentaenoic acid-hydroxy eicosenoic acid	FAHFA(20:5-O-20:1)	C40H66O4
227	719.5	334.3	(+)	35	BAHEA	Behenic acid-hydroxy eicosenoic acid	FAHFA(22:0-O-20:1)	C42H80O4
228	717.5	334.3	(+)	35	DEAHEA	Docosenoic acid-hydroxy eicosenoic acid	FAHFA(22:1-O-20:1)	C42H78O4
229	715.5	334.3	(+)	35	DDAHEA	Docosadienoate-hydroxy eicosenoic acid	FAHFA(22:2-O-20:1)	C40H76O4
230	707.5	334.3	(+)	35	DHAHEA	Docosahexaenoic acid-hydroxy eicosenoic acid	FAHFA(22:6-O-20:1)	C42H68O4
231	605.5	332.3	(+)	35	MAHEDA	Myristic acid-hydroxy eicosadienoic acid	FAHFA(14:0-O-20:2)	C34H62O4
232	603.5	332.3	(+)	35	MOHEDA	Myristoleic acid-hydroxy eicosadienoic acid	FAHFA(14:1-O-20:2)	C34H60O4
233	619.5	332.3	(+)	35	PDAHEDA	Pentadecanoic acid-hydroxy eicosadienoic acid	FAHFA(15:0-O-20:2)	C35H64O4
234	617.5	332.3	(+)	35	PDEAHEDA	Pentadecenoic acid-hydroxy eicosadienoic acid	FAHFA(15:1-O-20:2)	C35H62O4
235	631.5	332.3	(+)	35	POHEDA	Palmitoleic acid-hydroxy eicosadienoic acid	FAHFA(16:1-O-20:2)	C36H64O4
236	633.5	332.3	(+)	35	PAHEDA	Palmitic acid-hydroxy eicosadienoic acid	FAHFA(16:0-O-20:2)	C36H66O4
237	647.5	332.3	(+)	35	HDAHEDA	Heptadecanoic acid-hydroxy eicosadienoic acid	FAHFA(17:0-O-20:2)	C37H68O4
238	645.5	332.3	(+)	35	HDEAHEDA	Heptadecenoic acid-hydroxy eicosadienoic acid	FAHFA(17:1-O-20:2)	C37H66O4
239	661.5	332.3	(+)	35	SAHEDA	Stearic acid-hydroxy eicosadienoic acid	FAHFA(18:0-O-20:2)	C38H70O4
240	659.5	332.3	(+)	35	OAHEDA	Oleic acid-hydroxy eicosadienoic acid	FAHFA(18:1-O-20:2)	C38H68O4
241	657.5	332.3	(+)	35	LAHEDA	Linoleic acid-hydroxy eicosadienoic acid	FAHFA(18:2-O-20:2)	C38H66O4
242	655.5	332.3	(+)	35	ALAHEDA	α-Linolenic acid-hydroxy eicosadienoic acid	FAHFA(18:3-O-20:2)	C38H64O4
243	653.5	332.3	(+)	35	SDAHEDA	Stearidonic acid-hydroxy eicosadienoic acid	FAHFA(18:4-O-20:2)	C38H62O4
244	689.5	332.3	(+)	35	AAHEDA	Arachidic acid-hydroxy eicosadienoic acid	FAHFA(20:0-O-20:2)	C40H74O4
245	687.5	332.3	(+)	35	EAHEDA	Eicosenoic acid-hydroxy eicosadienoic acid	FAHFA(20:1-O-20:2)	C40H72O4
246	685.5	332.3	(+)	35	EDAHEDA	Eicosadienoic acid-hydroxy eicosadienoic acid	FAHFA(20:2-O-20:2)	C40H70O4
247	683.5	332.3	(+)	35	ETAHEDA	Eicosatrienoic acid-hydroxy eicosadienoic acid	FAHFA(20:3-O-20:2)	C40H68O4
248	681.5	332.3	(+)	35	ARAHEDA	Arachidonic acid-hydroxy eicosadienoic acid	FAHFA(20:4-O-20:2)	C40H66O4
249	679.5	332.3	(+)	35	EPAHEDA	Eicosapentaenoic acid-hydroxy eicosadienoic acid	FAHFA(20:5-O-20:2)	C40H64O4
250	717.5	332.3	(+)	35	BAHEDA	Behenic acid-hydroxy eicosadienoic acid	FAHFA(22:0-O-20:2)	C42H78O4
251	715.5	332.3	(+)	35	DEAHEDA	Docosenoic acid-hydroxy eicosadienoic acid	FAHFA(22:1-O-20:2)	C42H76O4
252	713.5	332.3	(+)	35	DDAHEDA	Docosadienoate-hydroxy eicosadienoic acid	FAHFA(22:2-O-20:2)	C40H74O4
253	705.5	332.3	(+)	35	DHAHEDA	Docosahexaenoic acid-hydroxy eicosadienoic acid	FAHFA(22:6-O-20:2)	C42H66O4
254	603.5	330.3	(+)	35	MAHETA	Myristic acid-hydroxy eicosatrienoic acid	FAHFA(14:0-O-20:3)	C34H60O4
255	601.5	330.3	(+)	35	MOHETA	Myristoleic acid-hydroxy eicosatrienoic acid	FAHFA(14:1-O-20:3)	C34H58O4
256	617.5	330.3	(+)	35	PDAHETA	Pentadecanoic acid-hydroxy eicosatrienoic acid	FAHFA(15:0-O-20:3)	C35H62O4
257	615.5	330.3	(+)	35	PDEAHETA	Pentadecenoic acid-hydroxy eicosatrienoic acid	FAHFA(15:1-O-20:3)	C35H60O4
258	629.5	330.3	(+)	35	POHETA	Palmitoleic acid-hydroxy eicosatrienoic acid	FAHFA(16:1-O-20:3)	C36H62O4
259	631.5	330.3	(+)	35	PAHETA	Palmitic acid-hydroxy eicosatrienoic acid	FAHFA(16:0-O-20:3)	C36H64O4
260	645.5	330.3	(+)	35	HDAHETA	Heptadecanoic acid-hydroxy eicosatrienoic acid	FAHFA(17:0-O-20:3)	C37H66O4
261	643.5	330.3	(+)	35	HDEAHETA	Heptadecenoic acid-hydroxy eicosatrienoic acid	FAHFA(17:1-O-20:3)	C37H64O4
262	659.5	330.3	(+)	35	SAHETA	Stearic acid-hydroxy eicosatrienoic acid	FAHFA(18:0-O-20:3)	C38H68O4
263	657.5	330.3	(+)	35	OAHETA	Oleic acid-hydroxy eicosatrienoic acid	FAHFA(18:1-O-20:3)	C38H66O4
264	655.5	330.3	(+)	35	LAHETA	Linoleic acid-hydroxy eicosatrienoic acid	FAHFA(18:2-O-20:3)	C38H64O4
265	653.5	330.3	(+)	35	ALAHETA	α-Linolenic acid-hydroxy eicosatrienoic acid	FAHFA(18:3-O-20:3)	C38H62O4
266	651.5	330.3	(+)	35	SDAHETA	Stearidonic acid-hydroxy eicosatrienoic acid	FAHFA(18:4-O-20:3)	C38H60O4
267	687.5	330.3	(+)	35	AAHETA	Arachidic acid-hydroxy eicosatrienoic acid	FAHFA(20:0-O-20:3)	C40H72O4
268	685.5	330.3	(+)	35	EAHETA	Eicosenoic acid-hydroxy eicosatrienoic acid	FAHFA(20:1-O-20:3)	C40H70O4
269	683.5	330.3	(+)	35	EDAHETA	Eicosadienoic acid-hydroxy eicosatrienoic acid	FAHFA(20:2-O-20:3)	C40H68O4
270	681.5	330.3	(+)	35	ETAHETA	Eicosatrienoic acid-hydroxy eicosatrienoic acid	FAHFA(20:3-O-20:3)	C40H66O4
271	679.5	330.3	(+)	35	ARAHETA	Arachidonic acid-hydroxy eicosatrienoic acid	FAHFA(20:4-O-20:3)	C40H64O4
272	677.5	330.3	(+)	35	EPAHETA	Eicosapentaenoic acid-hydroxy eicosatrienoic acid	FAHFA(20:5-O-20:3)	C40H62O4
273	715.5	330.3	(+)	35	BAHETA	Behenic acid-hydroxy eicosatrienoic acid	FAHFA(22:0-O-20:3)	C42H76O4
274	713.5	330.3	(+)	35	DEAHETA	Docosenoic acid-hydroxy eicosatrienoic acid	FAHFA(22:1-O-20:3)	C42H74O4
275	711.5	330.3	(+)	35	DDAHETA	Docosadienoate-hydroxy eicosatrienoic acid	FAHFA(22:2-O-20:3)	C40H72O4
276	703.5	330.3	(+)	35	DHAHETA	Docosahexaenoic acid-hydroxy eicosatrienoic acid	FAHFA(22:6-O-20:3)	C42H64O4
277	625.5	352.3	(+)	35	MAHDHA	Myristic acid-hydroxy docosahexaenoic acid	FAHFA(14:0-O-22:6)	C36H58O4
278	623.5	352.3	(+)	35	MOHDHA	Myristoleic acid-hydroxy docosahexaenoic acid	FAHFA(14:1-O-22:6)	C36H56O4
279	639.5	352.3	(+)	35	PDAHDHA	Pentadecanoic acid-hydroxy docosahexaenoic acid	FAHFA(15:0-O-22:6)	C37H60O4
280	637.5	352.3	(+)	35	PDEAHDHA	Pentadecenoic acid-hydroxy docosahexaenoic acid	FAHFA(15:1-O-22:6)	C37H58O4
281	651.5	352.3	(+)	35	POHDHA	Palmitoleic acid-hydroxy docosahexaenoic acid	FAHFA(16:1-O-22:6)	C38H60O4
282	653.5	352.3	(+)	35	PAHDHA	Palmitic acid-hydroxy docosahexaenoic acid	FAHFA(16:0-O-22:6)	C38H62O4
283	667.5	352.3	(+)	35	HDAHDHA	Heptadecanoic acid-hydroxy docosahexaenoic acid	FAHFA(17:0-O-22:6)	C39H64O4
284	665.5	352.3	(+)	35	HDEAHDHA	Heptadecenoic acid-hydroxy docosahexaenoic acid	FAHFA(17:1-O-22:6)	C39H62O4
285	681.5	352.3	(+)	35	SAHDHA	Stearic acid-hydroxy docosahexaenoic acid	FAHFA(18:0-O-22:6)	C40H66O4
286	679.5	352.3	(+)	35	OAHDHA	Oleic acid-hydroxy docosahexaenoic acid	FAHFA(18:1-O-22:6)	C40H64O4
287	677.5	352.3	(+)	35	LAHDHA	Linoleic acid-hydroxy docosahexaenoic acid	FAHFA(18:2-O-22:6)	C40H62O4
288	675.5	352.3	(+)	35	ALAHDHA	α-Linolenic acid-hydroxy docosahexaenoic acid	FAHFA(18:3-O-22:6)	C40H60O4
289	673.5	352.3	(+)	35	SDAHDHA	Stearidonic acid-hydroxy docosahexaenoic acid	FAHFA(18:4-O-22:6)	C40H58O4
290	709.5	352.3	(+)	35	AAHDHA	Arachidic acid-hydroxy docosahexaenoic acid	FAHFA(20:0-O-22:6)	C42H70O4
291	707.5	352.3	(+)	35	EAHDHA	Eicosenoic acid-hydroxy docosahexaenoic acid	FAHFA(20:1-O-22:6)	C42H68O4
292	705.5	352.3	(+)	35	EDAHDHA	Eicosadienoic acid-hydroxy docosahexaenoic acid	FAHFA(20:2-O-22:6)	C42H66O4
293	703.5	352.3	(+)	35	ETAHDHA	Eicosatrienoic acid-hydroxy docosahexaenoic acid	FAHFA(20:3-O-22:6)	C42H64O4
294	701.5	352.3	(+)	35	ARAHDHA	Arachidonic acid-hydroxy docosahexaenoic acid	FAHFA(20:4-O-22:6)	C42H62O4
295	699.5	352.3	(+)	35	EPAHDHA	Eicosapentaenoic acid-hydroxy docosahexaenoic acid	FAHFA(20:5-O-22:6)	C42H60O4
296	737.5	352.3	(+)	35	BAHDHA	Behenic acid-hydroxy docosahexaenoic acid	FAHFA(22:0-O-22:6)	C44H74O4
297	735.5	352.3	(+)	35	DEAHDHA	Docosenoic acid-hydroxy docosahexaenoic acid	FAHFA(22:1-O-22:6)	C44H72O4
298	733.5	352.3	(+)	35	DDAHDHA	Docosadienoate-hydroxy docosahexaenoic acid	FAHFA(22:2-O-22:6)	C44H70O4
299	725.5	352.3	(+)	35	DHAHDHA	Docosahexaenoic acid-hydroxy docosahexaenoic acid	FAHFA(22:6-O-22:6)	C44H62O4
300	601.5	356.3	(+)	35	MAHARA	Myristic acid-hydroxy arachidonic acid	FAHFA(14:0-O-20:4)	C34H58O4
301	599.5	356.3	(+)	35	MOHARA	Myristoleic acid-hydroxy arachidonic acid	FAHFA(14:1-O-20:4)	C34H56O4
302	615.5	356.3	(+)	35	PDAHARA	Pentadecanoic acid-hydroxy arachidonic acid	FAHFA(15:0-O-20:4)	C35H60O4
303	613.5	356.3	(+)	35	PDEAHARA	Pentadecenoic acid-hydroxy arachidonic acid	FAHFA(15:1-O-20:4)	C35H58O4
304	627.5	356.3	(+)	35	POHARA	Palmitoleic acid-hydroxy arachidonic acid	FAHFA(16:1-O-20:4)	C36H60O4
305	629.5	356.3	(+)	35	PAHARA	Palmitic acid-hydroxy arachidonic acid	FAHFA(16:0-O-20:4)	C36H62O4
306	643.5	356.3	(+)	35	HDAHARA	Heptadecanoic acid-hydroxy arachidonic acid	FAHFA(17:0-O-20:4)	C37H64O4
307	641.5	356.3	(+)	35	HDEAHARA	Heptadecenoic acid-hydroxy arachidonic acid	FAHFA(17:1-O-20:4)	C37H62O4
308	657.5	356.3	(+)	35	SAHARA	Stearic acid-hydroxy arachidonic acid	FAHFA(18:0-O-20:4)	C38H66O4
309	655.5	356.3	(+)	35	OAHARA	Oleic acid-hydroxy arachidonic acid	FAHFA(18:1-O-20:4)	C38H64O4

310	653.5	356.3	(+)	35	LAHARA	Linoleic acid-hydroxy arachidonic acid	FAHFA(18:2-O-20:4)	$C_{38}H_{62}O_4$
311	651.5	356.3	(+)	35	ALAHARA	α-Linolenic acid-hydroxy arachidonic acid	FAHFA(18:3-O-20:4)	$C_{38}H_{60}O_4$
312	649.5	356.3	(+)	35	SDAHARA	Stearidonic acid-hydroxy arachidonic acid	FAHFA(18:4-O-20:4)	$C_{38}H_{58}O_4$
313	685.5	356.3	(+)	35	AAHARA	Arachidic acid-hydroxy arachidonic acid	FAHFA(20:0-O-20:4)	$C_{40}H_{70}O_4$
314	683.5	356.3	(+)	35	EAHARA	Eicosenoic acid-hydroxy arachidonic acid	FAHFA(20:1-O-20:4)	$C_{40}H_{68}O_4$
315	681.5	356.3	(+)	35	EDAHARA	Eicosadienoic acid-hydroxy arachidonic acid	FAHFA(20:2-O-20:4)	$C_{40}H_{66}O_4$
316	679.5	356.3	(+)	35	ETAHARA	Eicosatrienoic acid-hydroxy arachidonic acid	FAHFA(20:3-O-20:4)	$C_{40}H_{64}O_4$
317	677.5	356.3	(+)	35	ARAHARA	Arachidonic acid-hydroxy arachidonic acid	FAHFA(20:4-O-20:4)	$C_{40}H_{62}O_4$
318	675.5	356.3	(+)	35	EPAHARA	Eicosapentaenoic acid-hydroxy arachidonic acid	FAHFA(20:5-O-20:4)	$C_{40}H_{60}O_4$
319	713.5	356.3	(+)	35	BAHARA	Behenic acid-hydroxy arachidonic acid	FAHFA(22:0-O-20:4)	$C_{42}H_{74}O_4$
320	711.5	356.3	(+)	35	DEAHARA	Docosenoic acid-hydroxy arachidonic acid	FAHFA(22:1-O-20:4)	$C_{42}H_{72}O_4$
321	709.5	356.3	(+)	35	DDAHARA	Docosadienoate-hydroxy arachidonic acid	FAHFA(22:2-O-20:4)	$C_{40}H_{70}O_4$
322	701.5	356.3	(+)	35	DHAHARA	Docosahexaenoic acid-hydroxy arachidonic acid	FAHFA(22:6-O-20:4)	$C_{42}H_{62}O_4$
323	497.5	224.2	(+)	35	MAHDA	Myristic acid-hydroxy dodecanoic acid	FAHFA(14:0-O-12:0)	$C_{26}H_{50}O_4$
324	495.5	224.2	(+)	35	MOHDA	Myristoleic acid-hydroxy dodecanoic acid	FAHFA(14:1-O-12:0)	$C_{22}H_{48}O_4$
325	511.5	224.2	(+)	35	PDAHDA	Pentadecanoic acid-hydroxy dodecanoic acid	FAHFA(15:0-O-12:0)	$C_{27}H_{52}O_4$
326	509.5	224.2	(+)	35	PDEAHDA	Pentadecenoic acid-hydroxy dodecanoic acid	FAHFA(15:1-O-12:0)	$C_{27}H_{50}O_4$
327	523.5	224.2	(+)	35	POHDA	Palmitoleic acid-hydroxy dodecanoic acid	FAHFA(16:1-O-12:0)	$C_{28}H_{52}O_4$
328	525.5	224.2	(+)	35	PAHDA	Palmitic acid-hydroxy dodecanoic acid	FAHFA(16:0-O-12:0)	$C_{28}H_{54}O_4$
329	539.5	224.2	(+)	35	HDAHDA	Heptadecanoic acid-hydroxy dodecanoic acid	FAHFA(17:0-O-12:0)	$C_{29}H_{56}O_4$
330	537.5	224.2	(+)	35	HDEAHDA	Heptadecenoic acid-hydroxy dodecanoic acid	FAHFA(17:1-O-12:0)	$C_{29}H_{54}O_4$
331	553.5	224.2	(+)	35	SAHDA	Stearic acid-hydroxy dodecanoic acid	FAHFA(18:0-O-12:0)	$C_{30}H_{58}O_4$
332	551.5	224.2	(+)	35	OAHDA	Oleic acid-hydroxy dodecanoic acid	FAHFA(18:1-O-12:0)	$C_{30}H_{56}O_4$
333	549.5	224.2	(+)	35	LAHDA	Linoleic acid-hydroxy dodecanoic acid	FAHFA(18:2-O-12:0)	$C_{30}H_{54}O_4$
334	547.5	224.2	(+)	35	ALAHDA	α-Linolenic acid-hydroxy dodecanoic acid	FAHFA(18:3-O-12:0)	$C_{30}H_{52}O_4$
335	545.5	224.2	(+)	35	SDAHDA	Stearidonic acid-hydroxy dodecanoic acid	FAHFA(18:4-O-12:0)	$C_{30}H_{50}O_4$
336	581.5	224.2	(+)	35	AAHDA	Arachidic acid-hydroxy dodecanoic acid	FAHFA(20:0-O-12:0)	$C_{32}H_{62}O_4$
337	579.5	224.2	(+)	35	EAHDA	Eicosenoic acid-hydroxy dodecanoic acid	FAHFA(20:1-O-12:0)	$C_{32}H_{60}O_4$
338	577.5	224.2	(+)	35	EDAHDA	Eicosadienoic acid-hydroxy dodecanoic acid	FAHFA(20:2-O-12:0)	$C_{32}H_{58}O_4$
339	575.5	224.2	(+)	35	ETAHDA	Eicosatrienoic acid-hydroxy dodecanoic acid	FAHFA(20:3-O-12:0)	$C_{32}H_{56}O_4$
340	573.5	224.2	(+)	35	ARAHDA	Arachidonic acid-hydroxy dodecanoic acid	FAHFA(20:4-O-12:0)	$C_{32}H_{54}O_4$
341	571.5	224.2	(+)	35	EPAHDA	Eicosapentaenoic acid-hydroxy dodecanoic acid	FAHFA(20:5-O-12:0)	$C_{32}H_{52}O_4$
342	609.5	224.2	(+)	35	BAHDA	Behenic acid-hydroxy dodecanoic acid	FAHFA(22:0-O-12:0)	$C_{34}H_{66}O_4$
343	607.5	224.2	(+)	35	DEAHDA	Docosenoic acid-hydroxy dodecanoic acid	FAHFA(22:1-O-12:0)	$C_{34}H_{64}O_4$
344	605.5	224.2	(+)	35	DDAHDA	Docosadienoate-hydroxy dodecanoic acid	FAHFA(22:2-O-12:0)	$C_{34}H_{62}O_4$
345	597.5	224.2	(+)	35	DHAHDA	Docosahexaenoic acid-hydroxy dodecanoic acid	FAHFA(22:6-O-12:0)	$C_{34}H_{54}O_4$
346	525.5	252.2	(+)	35	MAHMA	Myristic acid-hydroxy myristic acid	FAHFA(14:0-O-14:0)	$C_{28}H_{54}O_4$
347	523.5	252.2	(+)	35	MOHMA	Myristoleic acid-hydroxy myristic acid	FAHFA(14:1-O-14:0)	$C_{28}H_{52}O_4$
348	539.5	252.2	(+)	35	PDAHMA	Pentadecanoic acid-hydroxy myristic acid	FAHFA(15:0-O-14:0)	$C_{29}H_{56}O_4$
349	537.5	252.2	(+)	35	PDEAHMA	Pentadecenoic acid-hydroxy myristic acid	FAHFA(15:1-O-14:0)	$C_{29}H_{54}O_4$
350	551.5	252.2	(+)	35	POHMA	Palmitoleic acid-hydroxy myristic acid	FAHFA(16:1-O-14:0)	$C_{30}H_{56}O_4$
351	553.5	252.2	(+)	35	PAHMA	Palmitic acid-hydroxy myristic acid	FAHFA(16:0-O-14:0)	$C_{30}H_{58}O_4$
352	567.5	252.2	(+)	35	HDAHMA	Heptadecanoic acid-hydroxy myristic acid	FAHFA(17:0-O-14:0)	$C_{31}H_{60}O_4$
353	565.5	252.2	(+)	35	HDEAHMA	Heptadecenoic acid-hydroxy myristic acid	FAHFA(17:1-O-14:0)	$C_{31}H_{58}O_4$
354	581.5	252.2	(+)	35	SAHMA	Stearic acid-hydroxy myristic acid	FAHFA(18:0-O-14:0)	$C_{32}H_{62}O_4$
355	579.5	252.2	(+)	35	OAHMA	Oleic acid-hydroxy myristic acid	FAHFA(18:1-O-14:0)	$C_{32}H_{60}O_4$
356	577.5	252.2	(+)	35	LAHMA	Linoleic acid-hydroxy myristic acid	FAHFA(18:2-O-14:0)	$C_{32}H_{58}O_4$
357	575.5	252.2	(+)	35	ALAHMA	α-Linolenic acid-hydroxy myristic acid	FAHFA(18:3-O-14:0)	$C_{32}H_{56}O_4$
358	573.5	252.2	(+)	35	SDAHMA	Stearidonic acid-hydroxy myristic acid	FAHFA(18:4-O-14:0)	$C_{32}H_{54}O_4$
359	609.5	252.2	(+)	35	AAHMA	Arachidic acid-hydroxy myristic acid	FAHFA(20:0-O-14:0)	$C_{34}H_{66}O_4$
360	607.5	252.2	(+)	35	EAHMA	Eicosenoic acid-hydroxy myristic acid	FAHFA(20:1-O-14:0)	$C_{34}H_{64}O_4$
361	605.5	252.2	(+)	35	EDAHMA	Eicosadienoic acid-hydroxy myristic acid	FAHFA(20:2-O-14:0)	$C_{34}H_{62}O_4$
362	603.5	252.2	(+)	35	ETAHMA	Eicosatrienoic acid-hydroxy myristic acid	FAHFA(20:3-O-14:0)	$C_{34}H_{60}O_4$
363	601.5	252.2	(+)	35	ARAHMA	Arachidonic acid-hydroxy myristic acid	FAHFA(20:4-O-14:0)	$C_{34}H_{58}O_4$
364	599.5	252.2	(+)	35	EPAHMA	Eicosapentaenoic acid-hydroxy myristic acid	FAHFA(20:5-O-14:0)	$C_{34}H_{56}O_4$
365	637.5	252.2	(+)	35	BAHMA	Behenic acid-hydroxy myristic acid	FAHFA(22:0-O-14:0)	$C_{36}H_{70}O_4$
366	635.5	252.2	(+)	35	DEAHMA	Docosenoic acid-hydroxy myristic acid	FAHFA(22:1-O-14:0)	$C_{36}H_{68}O_4$
367	633.5	252.2	(+)	35	DDAHMA	Docosadienoate-hydroxy myristic acid	FAHFA(22:2-O-14:0)	$C_{36}H_{66}O_4$
368	625.5	252.2	(+)	35	DHAHMA	Docosahexaenoic acid-hydroxy myristic acid	FAHFA(22:6-O-14:0)	$C_{36}H_{58}O_4$
369	539.5	266.2	(+)	35	MAHPDA	Myristic acid-hydroxy pentadecanoic acid	FAHFA(14:0-O-15:0)	$C_{29}H_{56}O_4$
370	537.5	266.2	(+)	35	MOHPDA	Myristoleic acid-hydroxy pentadecanoic acid	FAHFA(14:1-O-15:0)	$C_{29}H_{54}O_4$
371	553.5	266.2	(+)	35	PDAHPDA	Pentadecanoic acid-hydroxy pentadecanoic acid	FAHFA(15:0-O-15:0)	$C_{30}H_{58}O_4$
372	551.5	266.2	(+)	35	PDEAHPDA	Pentadecenoic acid-hydroxy pentadecanoic acid	FAHFA(15:1-O-15:0)	$C_{30}H_{56}O_4$
373	565.5	266.2	(+)	35	POHPDA	Palmitoleic acid-hydroxy pentadecanoic acid	FAHFA(16:1-O-15:0)	$C_{31}H_{58}O_4$
374	567.5	266.2	(+)	35	PAHPDA	Palmitic acid-hydroxy pentadecanoic acid	FAHFA(16:0-O-15:0)	$C_{31}H_{60}O_4$
375	581.5	266.2	(+)	35	HDAHPDA	Heptadecanoic acid-hydroxy pentadecanoic acid	FAHFA(17:0-O-15:0)	$C_{32}H_{62}O_4$
376	579.5	266.2	(+)	35	HDEAHPDA	Heptadecenoic acid-hydroxy pentadecanoic acid	FAHFA(17:1-O-15:0)	$C_{32}H_{60}O_4$
377	595.5	266.2	(+)	35	SAHPDA	Stearic acid-hydroxy pentadecanoic acid	FAHFA(18:0-O-15:0)	$C_{33}H_{64}O_4$
378	593.5	266.2	(+)	35	OAHPDA	Oleic acid-hydroxy pentadecanoic acid	FAHFA(18:1-O-15:0)	$C_{33}H_{62}O_4$
379	591.5	266.2	(+)	35	LAHPDA	Linoleic acid-hydroxy pentadecanoic acid	FAHFA(18:2-O-15:0)	$C_{33}H_{60}O_4$
380	589.5	266.2	(+)	35	ALAHPDA	α-Linolenic acid-hydroxy pentadecanoic acid	FAHFA(18:3-O-15:0)	$C_{33}H_{58}O_4$
381	587.5	266.2	(+)	35	SDAHPDA	Stearidonic acid-hydroxy pentadecanoic acid	FAHFA(18:4-O-15:0)	$C_{33}H_{56}O_4$
382	623.5	266.2	(+)	35	AAHPDA	Arachidic acid-hydroxy pentadecanoic acid	FAHFA(20:0-O-15:0)	$C_{35}H_{68}O_4$
383	621.5	266.2	(+)	35	EAHPDA	Eicosenoic acid-hydroxy pentadecanoic acid	FAHFA(20:1-O-15:0)	$C_{35}H_{66}O_4$
384	619.5	266.2	(+)	35	EDAHPDA	Eicosadienoic acid-hydroxy pentadecanoic acid	FAHFA(20:2-O-15:0)	$C_{35}H_{64}O_4$
385	617.5	266.2	(+)	35	ETAHPDA	Eicosatrienoic acid-hydroxy pentadecanoic acid	FAHFA(20:3-O-15:0)	$C_{35}H_{62}O_4$
386	615.5	266.2	(+)	35	ARAHPDA	Arachidonic acid-hydroxy pentadecanoic acid	FAHFA(20:4-O-15:0)	$C_{35}H_{60}O_4$
387	613.5	266.2	(+)	35	EPAHPDA	Eicosapentaenoic acid-hydroxy pentadecanoic acid	FAHFA(20:5-O-15:0)	$C_{35}H_{58}O_4$
388	651.5	266.2	(+)	35	BAHPDA	Behenic acid-hydroxy pentadecanoic acid	FAHFA(22:0-O-15:0)	$C_{37}H_{72}O_4$
389	649.5	266.2	(+)	35	DEAHPDA	Docosenoic acid-hydroxy pentadecanoic acid	FAHFA(22:1-O-15:0)	$C_{37}H_{70}O_4$
390	647.5	266.2	(+)	35	DDAHPDA	Docosadienoate-hydroxy pentadecanoic acid	FAHFA(22:2-O-15:0)	$C_{37}H_{68}O_4$
391	639.5	266.2	(+)	35	DHAHPDA	Docosahexaenoic acid-hydroxy pentadecanoic acid	FAHFA(22:6-O-15:0)	$C_{37}H_{60}O_4$
392	553.6	280.3	(+)	35	MAHPA	Myristic acid-hydroxy palmitic acid	FAHFA(14:0-O-16:0)	$C_{30}H_{58}O_4$
393	551.6	280.3	(+)	35	MOHPA	Myristoleic acid-hydroxy palmitic acid	FAHFA(14:1-O-16:0)	$C_{30}H_{56}O_4$
394	567.6	280.3	(+)	35	PDAHPA	Pentadecanoic acid-hydroxy palmitic acid	FAHFA(15:0-O-16:0)	$C_{31}H_{60}O_4$
395	565.6	280.3	(+)	35	PDEAHPA	Pentadecenoic acid-hydroxy palmitic acid	FAHFA(15:1-O-16:0)	$C_{31}H_{58}O_4$
396	579.6	280.3	(+)	35	POHPA	Palmitoleic acid-hydroxy palmitic acid	FAHFA(16:1-O-16:0)	$C_{32}H_{60}O_4$
397	581.6	280.3	(+)	35	PAHPA	Palmitic acid-hydroxy palmitic acid	FAHFA(16:0-O-16:0)	$C_{32}H_{62}O_4$
398	595.6	280.3	(+)	35	HDAHPA	Heptadecanoic acid-hydroxy palmitic acid	FAHFA(17:0-O-16:0)	$C_{33}H_{64}O_4$
399	593.6	280.3	(+)	35	HDEAHPA	Heptadecenoic acid-hydroxy palmitic acid	FAHFA(17:1-O-16:0)	$C_{33}H_{62}O_4$
400	609.6	280.3	(+)	35	SAHPA	Stearic acid-hydroxy palmitic acid	FAHFA(18:0-O-16:0)	$C_{34}H_{66}O_4$
401	607.6	280.3	(+)	35	OAHPA	Oleic acid-hydroxy palmitic acid	FAHFA(18:1-O-16:0)	$C_{34}H_{64}O_4$
402	605.6	280.3	(+)	35	LAHPA	Linoleic acid-hydroxy palmitic acid	FAHFA(18:2-O-16:0)	$C_{34}H_{62}O_4$
403	603.6	280.3	(+)	35	ALAHPA	α-Linolenic acid-hydroxy palmitic acid	FAHFA(18:3-O-16:0)	$C_{34}H_{60}O_4$
404	601.6	280.3	(+)	35	SDAHPA	Stearidonic acid-hydroxy palmitic acid	FAHFA(18:4-O-16:0)	$C_{34}H_{58}O_4$
405	637.6	280.3	(+)	35	AAHPA	Arachidic acid-hydroxy palmitic acid	FAHFA(20:0-O-16:0)	$C_{36}H_{70}O_4$
406	635.6	280.3	(+)	35	EAHPA	Eicosenoic acid-hydroxy palmitic acid	FAHFA(20:1-O-16:0)	$C_{36}H_{68}O_4$
407	633.6	280.3	(+)	35	EDAHPA	Eicosadienoic acid-hydroxy palmitic acid	FAHFA(20:2-O-16:0)	$C_{36}H_{66}O_4$
408	631.6	280.3	(+)	35	ETAHPA	Eicosatrienoic acid-hydroxy palmitic acid	FAHFA(20:3-O-16:0)	$C_{36}H_{64}O_4$
409	629.6	280.3	(+)	35	ARAHPA	Arachidonic acid-hydroxy palmitic acid	FAHFA(20:4-O-16:0)	$C_{36}H_{62}O_4$
410	627.6	280.3	(+)	35	EPAHPA	Eicosapentaenoic acid-hydroxy palmitic acid	FAHFA(20:5-O-16:0)	$C_{36}H_{60}O_4$
411	665.6	280.3	(+)	35	BAHPA	Behenic acid-hydroxy palmitic acid	FAHFA(22:0-O-16:0)	$C_{38}H_{74}O_4$
412	663.6	280.3	(+)	35	DEAHPA	Docosenoic acid-hydroxy palmitic acid	FAHFA(22:1-O-16:0)	$C_{38}H_{72}O_4$
413	661.6	280.3	(+)	35	DDAHPA	Docosadienoate-hydroxy palmitic acid	FAHFA(22:2-O-16:0)	$C_{38}H_{70}O_4$
414	653.6	280.3	(+)	35	DHAHPA	Docosahexaenoic acid-hydroxy palmitic acid	FAHFA(22:6-O-16:0)	$C_{38}H_{62}O_4$

415	567.6	294.3	(+)	35	MAHHDA	Myristic acid-hydroxy heptadecanoic acid	FAHFA(14:0-O-17:0)	C31H60O4
416	565.6	294.3	(+)	35	MOHHDA	Myristoleic acid-hydroxy heptadecanoic acid	FAHFA(14:1-O-17:0)	C31H58O4
417	581.6	294.3	(+)	35	PDAHHDA	Pentadecanoic acid-hydroxy heptadecanoic acid	FAHFA(15:0-O-17:0)	C32H62O4
418	579.6	294.3	(+)	35	PDEAHHDA	Pentadecenoic acid-hydroxy heptadecanoic acid	FAHFA(15:1-O-17:0)	C32H60O4
419	593.6	294.3	(+)	35	POHHDA	Palmitoleic acid-hydroxy heptadecanoic acid	FAHFA(16:1-O-17:0)	C33H62O4
420	595.6	294.3	(+)	35	PAHHDA	Palmitic acid-hydroxy heptadecanoic acid	FAHFA(16:0-O-17:0)	C33H64O4
421	609.6	294.3	(+)	35	HDAHHDA	Heptadecanoic acid-hydroxy heptadecanoic acid	FAHFA(17:0-O-17:0)	C34H66O4
422	607.6	294.3	(+)	35	HDEAHHDA	Heptadecenoic acid-hydroxy heptadecanoic acid	FAHFA(17:1-O-17:0)	C34H64O4
423	623.6	294.3	(+)	35	SAHHDA	Stearic acid-hydroxy heptadecanoic acid	FAHFA(18:0-O-17:0)	C35H68O4
424	621.6	294.3	(+)	35	OAHHDA	Oleic acid-hydroxy heptadecanoic acid	FAHFA(18:1-O-17:0)	C35H66O4
425	619.6	294.3	(+)	35	LAHHDA	Linoleic acid-hydroxy heptadecanoic acid	FAHFA(18:2-O-17:0)	C35H64O4
426	617.6	294.3	(+)	35	ALAHHDA	α-Linolenic acid-hydroxy heptadecanoic acid	FAHFA(18:3-O-17:0)	C35H62O4
427	615.6	294.3	(+)	35	SDAHHDA	Stearidonic acid-hydroxy heptadecanoic acid	FAHFA(18:4-O-17:0)	C35H60O4
428	651.6	294.3	(+)	35	AAHHDA	Arachidic acid-hydroxy heptadecanoic acid	FAHFA(20:0-O-17:0)	C37H72O4
429	649.6	294.3	(+)	35	EAHHDA	Eicosenoic acid-hydroxy heptadecanoic acid	FAHFA(20:1-O-17:0)	C37H70O4
430	647.6	294.3	(+)	35	EDAHHDA	Eicosadienoic acid-hydroxy heptadecanoic acid	FAHFA(20:2-O-17:0)	C37H68O4
431	645.6	294.3	(+)	35	ETAHHDA	Eicosatrienoic acid-hydroxy heptadecanoic acid	FAHFA(20:3-O-17:0)	C37H66O4
432	643.6	294.3	(+)	35	ARAHHDA	Arachidonic acid-hydroxy heptadecanoic acid	FAHFA(20:4-O-17:0)	C37H64O4
433	641.6	294.3	(+)	35	EPAHHDA	Eicosapentaenoic acid-hydroxy heptadecanoic acid	FAHFA(20:5-O-17:0)	C37H62O4
434	679.6	294.3	(+)	35	BAHHDA	Behenic acid-hydroxy heptadecanoic acid	FAHFA(22:0-O-17:0)	C39H76O4
435	677.6	294.3	(+)	35	DEAHHDA	Docosenoic acid-hydroxy heptadecanoic acid	FAHFA(22:1-O-17:0)	C39H74O4
436	675.6	294.3	(+)	35	DDAHHDA	Docosadienoate-hydroxy heptadecanoic acid	FAHFA(22:2-O-17:0)	C39H72O4
437	667.6	294.3	(+)	35	DHAHHDA	Docosahexaenoic acid-hydroxy heptadecanoic acid	FAHFA(22:6-O-17:0)	C39H64O4
438	581.8	308.3	(+)	35	MAHSA	Myristic acid-hydroxy stearic acid	FAHFA(14:0-O-18:0)	C32H62O4
439	579.8	308.3	(+)	35	MOHSA	Myristoleic acid-hydroxy stearic acid	FAHFA(14:1-O-18:0)	C32H60O4
440	595.8	308.3	(+)	35	PDAHSA	Pentadecanoic acid-hydroxy stearic acid	FAHFA(15:0-O-18:0)	C33H64O4
441	593.8	308.3	(+)	35	PDEAHSA	Pentadecenoic acid-hydroxy stearic acid	FAHFA(15:1-O-18:0)	C33H62O4
442	607.8	308.3	(+)	35	POHSA	Palmitoleic acid-hydroxy stearic acid	FAHFA(16:1-O-18:0)	C34H64O4
443	609.8	308.3	(+)	35	PAHSA	Palmitic acid-hydroxy stearic acid	FAHFA(16:0-O-18:0)	C34H66O4
444	623.8	308.3	(+)	35	HDAHSA	Heptadecanoic acid-hydroxy stearic acid	FAHFA(17:0-O-18:0)	C35H68O4
445	621.8	308.3	(+)	35	HDEAHSA	Heptadecenoic acid-hydroxy stearic acid	FAHFA(17:1-O-18:0)	C35H66O4
446	637.8	308.3	(+)	35	SAHSA	Stearic acid-hydroxy stearic acid	FAHFA(18:0-O-18:0)	C36H70O4
447	635.8	308.3	(+)	35	OAHSA	Oleic acid-hydroxy stearic acid	FAHFA(18:1-O-18:0)	C36H68O4
448	633.8	308.3	(+)	35	LAHSA	Linoleic acid-hydroxy stearic acid	FAHFA(18:2-O-18:0)	C36H66O4
449	631.8	308.3	(+)	35	ALAHSA	α-Linolenic acid-hydroxy stearic acid	FAHFA(18:3-O-18:0)	C36H64O4
450	629.8	308.3	(+)	35	SDAHSA	Stearidonic acid-hydroxy stearic acid	FAHFA(18:4-O-18:0)	C36H62O4
451	665.8	308.3	(+)	35	AAHSA	Arachidic acid-hydroxy stearic acid	FAHFA(20:0-O-18:0)	C38H74O4
452	663.8	308.3	(+)	35	EAHSA	Eicosenoic acid-hydroxy stearic acid	FAHFA(20:1-O-18:0)	C38H72O4
453	661.8	308.3	(+)	35	EDAHSA	Eicosadienoic acid-hydroxy stearic acid	FAHFA(20:2-O-18:0)	C38H70O4
454	659.8	308.3	(+)	35	ETAHSA	Eicosatrienoic acid-hydroxy stearic acid	FAHFA(20:3-O-18:0)	C38H68O4
455	657.8	308.3	(+)	35	ARAHSA	Arachidonic acid-hydroxy stearic acid	FAHFA(20:4-O-18:0)	C38H66O4
456	655.8	308.3	(+)	35	EPAHSA	Eicosapentaenoic acid-hydroxy stearic acid	FAHFA(20:5-O-18:0)	C38H64O4
457	693.8	308.3	(+)	35	BAHSA	Behenic acid-hydroxy stearic acid	FAHFA(22:0-O-18:0)	C40H78O4
458	691.8	308.3	(+)	35	DEAHSA	Docosenoic acid-hydroxy stearic acid	FAHFA(22:1-O-18:0)	C40H76O4
459	689.8	308.3	(+)	35	DDAHSA	Docosadienoate-hydroxy stearic acid	FAHFA(22:2-O-18:0)	C40H74O4
460	681.8	308.3	(+)	35	DHAHSA	Docosahexaenoic acid-hydroxy stearic acid	FAHFA(22:6-O-18:0)	C40H66O4
461	595.8	322.3	(+)	35	MAHNDA	Myristic acid-hydroxy nonadecanoic acid	FAHFA(14:0-O-19:0)	C33H64O4
462	593.8	322.3	(+)	35	MOHNDA	Myristoleic acid-hydroxy nonadecanoic acid	FAHFA(14:1-O-19:0)	C33H62O4
463	609.8	322.3	(+)	35	PDAHNDA	Pentadecanoic acid-hydroxy nonadecanoic acid	FAHFA(15:0-O-19:0)	C34H66O4
464	607.8	322.3	(+)	35	PDEAHNDA	Pentadecenoic acid-hydroxy nonadecanoic acid	FAHFA(15:1-O-19:0)	C34H64O4
465	621.8	322.3	(+)	35	POHNDA	Palmitoleic acid-hydroxy nonadecanoic acid	FAHFA(16:1-O-19:0)	C35H66O4
466	623.8	322.3	(+)	35	PAHNDA	Palmitic acid-hydroxy nonadecanoic acid	FAHFA(16:0-O-19:0)	C35H68O4
467	637.8	322.3	(+)	35	HDAHNDA	Heptadecanoic acid-hydroxy nonadecanoic acid	FAHFA(17:0-O-19:0)	C36H70O4
468	635.8	322.3	(+)	35	HDEAHNDA	Heptadecenoic acid-hydroxy nonadecanoic acid	FAHFA(17:1-O-19:0)	C36H68O4
469	651.8	322.3	(+)	35	SAHNDA	Stearic acid-hydroxy nonadecanoic acid	FAHFA(18:0-O-19:0)	C37H72O4
470	649.8	322.3	(+)	35	OAHNDA	Oleic acid-hydroxy nonadecanoic acid	FAHFA(18:1-O-19:0)	C37H70O4
471	647.8	322.3	(+)	35	LAHNDA	Linoleic acid-hydroxy nonadecanoic acid	FAHFA(18:2-O-19:0)	C37H68O4
472	645.8	322.3	(+)	35	ALAHNDA	α-Linolenic acid-hydroxy nonadecanoic acid	FAHFA(18:3-O-19:0)	C37H66O4
473	643.8	322.3	(+)	35	SDAHNDA	Stearidonic acid-hydroxy nonadecanoic acid	FAHFA(18:4-O-19:0)	C37H64O4
474	679.8	322.3	(+)	35	AAHNDA	Arachidic acid-hydroxy nonadecanoic acid	FAHFA(20:0-O-19:0)	C39H76O4
475	677.8	322.3	(+)	35	EAHNDA	Eicosenoic acid-hydroxy nonadecanoic acid	FAHFA(20:1-O-19:0)	C39H74O4
476	675.8	322.3	(+)	35	EDAHNDA	Eicosadienoic acid-hydroxy nonadecanoic acid	FAHFA(20:2-O-19:0)	C39H72O4
477	673.8	322.3	(+)	35	ETAHNDA	Eicosatrienoic acid-hydroxy nonadecanoic acid	FAHFA(20:3-O-19:0)	C39H70O4
478	671.8	322.3	(+)	35	ARAHNDA	Arachidonic acid-hydroxy nonadecanoic acid	FAHFA(20:4-O-19:0)	C39H68O4
479	669.8	322.3	(+)	35	EPAHNDA	Eicosapentaenoic acid-hydroxy nonadecanoic acid	FAHFA(20:5-O-19:0)	C39H66O4
480	707.8	322.3	(+)	35	BAHNDA	Behenic acid-hydroxy nonadecanoic acid	FAHFA(22:0-O-19:0)	C41H80O4
481	705.8	322.3	(+)	35	DEAHNDA	Docosenoic acid-hydroxy nonadecanoic acid	FAHFA(22:1-O-19:0)	C41H78O4
482	703.8	322.3	(+)	35	DDAHNDA	Docosadienoate-hydroxy nonadecanoic acid	FAHFA(22:2-O-19:0)	C41H76O4
483	695.8	322.3	(+)	35	DHAHNDA	Docosahexaenoic acid-hydroxy nonadecanoic acid	FAHFA(22:6-O-19:0)	C41H68O4
484	609.8	336.3	(+)	35	MAHAA	Myristic acid-hydroxy arachidic acid	FAHFA(14:0-O-20:0)	C34H66O4
485	607.8	336.3	(+)	35	MOHAA	Myristoleic acid-hydroxy arachidic acid	FAHFA(14:1-O-20:0)	C34H64O4
486	623.8	336.3	(+)	35	PDAHAA	Pentadecanoic acid-hydroxy arachidic acid	FAHFA(15:0-O-20:0)	C35H68O4
487	621.8	336.3	(+)	35	PDEAHAA	Pentadecenoic acid-hydroxy arachidic acid	FAHFA(15:1-O-20:0)	C35H66O4
488	635.8	336.3	(+)	35	POHAA	Palmitoleic acid-hydroxy arachidic acid	FAHFA(16:1-O-20:0)	C36H68O4
489	637.8	336.3	(+)	35	PAHAA	Palmitic acid-hydroxy arachidic acid	FAHFA(16:0-O-20:0)	C36H70O4
490	651.8	336.3	(+)	35	HDAHAA	Heptadecanoic acid-hydroxy arachidic acid	FAHFA(17:0-O-20:0)	C37H72O4
491	649.8	336.3	(+)	35	HDEAHAA	Heptadecenoic acid-hydroxy arachidic acid	FAHFA(17:1-O-20:0)	C37H70O4
492	665.8	336.3	(+)	35	SAHAA	Stearic acid-hydroxy arachidic acid	FAHFA(18:0-O-20:0)	C38H74O4
493	663.8	336.3	(+)	35	OAHAA	Oleic acid-hydroxy arachidic acid	FAHFA(18:1-O-20:0)	C38H72O4
494	661.8	336.3	(+)	35	LAHAA	Linoleic acid-hydroxy arachidic acid	FAHFA(18:2-O-20:0)	C38H70O4
495	659.8	336.3	(+)	35	ALAHAA	α-Linolenic acid-hydroxy arachidic acid	FAHFA(18:3-O-20:0)	C38H68O4
496	657.8	336.3	(+)	35	SDAHAA	Stearidonic acid-hydroxy arachidic acid	FAHFA(18:4-O-20:0)	C38H66O4
497	693.8	336.3	(+)	35	AAHAA	Arachidic acid-hydroxy arachidic acid	FAHFA(20:0-O-20:0)	C40H78O4
498	691.8	336.3	(+)	35	EAHAA	Eicosenoic acid-hydroxy arachidic acid	FAHFA(20:1-O-20:0)	C40H76O4
499	689.8	336.3	(+)	35	EDAHAA	Eicosadienoic acid-hydroxy arachidic acid	FAHFA(20:2-O-20:0)	C40H74O4
500	687.8	336.3	(+)	35	ETAHAA	Eicosatrienoic acid-hydroxy arachidic acid	FAHFA(20:3-O-20:0)	C40H72O4
501	685.8	336.3	(+)	35	ARAHAA	Arachidonic acid-hydroxy arachidic acid	FAHFA(20:4-O-20:0)	C40H70O4
502	683.8	336.3	(+)	35	EPAHAA	Eicosapentaenoic acid-hydroxy arachidic acid	FAHFA(20:5-O-20:0)	C40H68O4
503	721.8	336.3	(+)	35	BAHAA	Behenic acid-hydroxy arachidic acid	FAHFA(22:0-O-20:0)	C42H82O4
504	719.8	336.3	(+)	35	DEAHAA	Docosenoic acid-hydroxy arachidic acid	FAHFA(22:1-O-20:0)	C42H80O4
505	717.8	336.3	(+)	35	DDAHAA	Docosadienoate-hydroxy arachidic acid	FAHFA(22:2-O-20:0)	C42H78O4
506	709.8	336.3	(+)	35	DHAHAA	Docosahexaenoic acid-hydroxy arachidic acid	FAHFA(22:6-O-20:0)	C42H70O4
507	623.8	350.3	(+)	35	MAHHEA	Myristic acid-hydroxy heneicosanoic acid	FAHFA(14:0-O-21:0)	C35H68O4
508	621.8	350.3	(+)	35	MOHHEA	Myristoleic acid-hydroxy heneicosanoic acid	FAHFA(14:1-O-21:0)	C35H66O4
509	637.8	350.3	(+)	35	PDAHHEA	Pentadecanoic acid-hydroxy heneicosanoic acid	FAHFA(15:0-O-21:0)	C36H70O4
510	635.8	350.3	(+)	35	PDEAHHEA	Pentadecenoic acid-hydroxy heneicosanoic acid	FAHFA(15:1-O-21:0)	C36H68O4
511	649.8	350.3	(+)	35	POHHEA	Palmitoleic acid-hydroxy heneicosanoic acid	FAHFA(16:1-O-21:0)	C37H70O4
512	651.8	350.3	(+)	35	PAHHEA	Palmitic acid-hydroxy heneicosanoic acid	FAHFA(16:0-O-21:0)	C37H72O4
513	665.8	350.3	(+)	35	HDAHHEA	Heptadecanoic acid-hydroxy heneicosanoic acid	FAHFA(17:0-O-21:0)	C38H74O4
514	663.8	350.3	(+)	35	HDEAHHEA	Heptadecenoic acid-hydroxy heneicosanoic acid	FAHFA(17:1-O-21:0)	C38H72O4
515	679.8	350.3	(+)	35	SAHHEA	Stearic acid-hydroxy heneicosanoic acid	FAHFA(18:0-O-21:0)	C39H76O4
516	677.8	350.3	(+)	35	OAHHEA	Oleic acid-hydroxy heneicosanoic acid	FAHFA(18:1-O-21:0)	C39H74O4
517	675.8	350.3	(+)	35	LAHHEA	Linoleic acid-hydroxy heneicosanoic acid	FAHFA(18:2-O-21:0)	C39H72O4
518	673.8	350.3	(+)	35	ALAHHEA	α-Linolenic acid-hydroxy heneicosanoic acid	FAHFA(18:3-O-21:0)	C39H70O4
519	671.8	350.3	(+)	35	SDAHHEA	Stearidonic acid-hydroxy heneicosanoic acid	FAHFA(18:4-O-21:0)	C39H68O4

520	707.8	350.3	(+)	35	AAHHEA	Arachidic acid-hydroxy heneicosanoic acid	FAHFA(21:0-O-21:0)	C41H80O4
521	705.8	350.3	(+)	35	EAHHEA	Eicosenoic acid-hydroxy heneicosanoic acid	FAHFA(20:1-O-21:0)	C41H78O4
522	703.8	350.3	(+)	35	EDAHHEA	Eicosadienoic acid-hydroxy heneicosanoic acid	FAHFA(20:2-O-21:0)	C41H76O4
523	701.8	350.3	(+)	35	ETAHHEA	Eicosatrienoic acid-hydroxy heneicosanoic acid	FAHFA(20:3-O-21:0)	C41H74O4
524	699.8	350.3	(+)	35	ARAHHEA	Arachidonic acid-hydroxy heneicosanoic acid	FAHFA(20:4-O-21:0)	C41H72O4
525	697.8	350.3	(+)	35	EPAHHEA	Eicosapentaenoic acid-hydroxy heneicosanoic acid	FAHFA(20:5-O-21:0)	C41H70O4
526	735.8	350.3	(+)	35	BAHHEA	Behenic acid-hydroxy heneicosanoic acid	FAHFA(22:0-O-21:0)	C43H84O4
527	733.8	350.3	(+)	35	DEAHHEA	Docosenoic acid-hydroxy heneicosanoic acid	FAHFA(22:1-O-21:0)	C43H82O4
528	731.8	350.3	(+)	35	DDAHHEA	Docosadienoate-hydroxy heneicosanoic acid	FAHFA(22:2-O-21:0)	C43H80O4
529	723.8	350.3	(+)	35	DHAHHEA	Docosahexaenoic acid-hydroxy heneicosanoic acid	FAHFA(22:6-O-21:0)	C43H72O4
530	527.5	250.2	(+)	35	MAHMO	Myristic acid-hydroxy myristoleic acid	FAHFA(14:0-O-14:1)	C28H52O4
531	525.5	250.2	(+)	35	MOHMO	Myristoleic acid-hydroxy myristoleic acid	FAHFA(14:1-O-14:1)	C28H50O4
532	541.5	250.2	(+)	35	PDAHMO	Pentadecanoic acid-hydroxy myristoleic acid	FAHFA(15:0-O-14:1)	C29H54O4
533	539.5	250.2	(+)	35	PDEAHMO	Pentadecenoic acid-hydroxy myristoleic acid	FAHFA(15:1-O-14:1)	C29H52O4
534	553.5	250.2	(+)	35	POHMO	Palmitoleic acid-hydroxy myristoleic acid	FAHFA(16:1-O-14:1)	C30H54O4
535	555.5	250.2	(+)	35	PAHMO	Palmitic acid-hydroxy myristoleic acid	FAHFA(16:0-O-14:1)	C30H56O4
536	569.5	250.2	(+)	35	HDAHMO	Heptadecanoic acid-hydroxy myristoleic acid	FAHFA(17:0-O-14:1)	C31H58O4
537	567.5	250.2	(+)	35	HDEAHMO	Heptadecenoic acid-hydroxy myristoleic acid	FAHFA(17:1-O-14:1)	C31H56O4
538	583.5	250.2	(+)	35	SAHMO	Stearic acid-hydroxy myristoleic acid	FAHFA(18:0-O-14:1)	C32H60O4
539	581.5	250.2	(+)	35	OAHMO	Oleic acid-hydroxy myristoleic acid	FAHFA(18:1-O-14:1)	C32H58O4
540	579.5	250.2	(+)	35	LAHMO	Linoleic acid-hydroxy myristoleic acid	FAHFA(18:2-O-14:1)	C32H56O4
541	577.5	250.2	(+)	35	ALAHMO	α-Linolenic acid-hydroxy myristoleic acid	FAHFA(18:3-O-14:1)	C32H54O4
542	575.5	250.2	(+)	35	SDAHMO	Stearidonic acid-hydroxy myristoleic acid	FAHFA(18:4-O-14:1)	C32H52O4
543	611.5	250.2	(+)	35	AAHMO	Arachidic acid-hydroxy myristoleic acid	FAHFA(20:0-O-14:1)	C34H64O4
544	609.5	250.2	(+)	35	EAHMO	Eicosenoic acid-hydroxy myristoleic acid	FAHFA(20:1-O-14:1)	C34H62O4
545	607.5	250.2	(+)	35	EDAHMO	Eicosadienoic acid-hydroxy myristoleic acid	FAHFA(20:2-O-14:1)	C34H60O4
546	605.5	250.2	(+)	35	ETAHMO	Eicosatrienoic acid-hydroxy myristoleic acid	FAHFA(20:3-O-14:1)	C34H58O4
547	603.5	250.2	(+)	35	ARAHMO	Arachidonic acid-hydroxy myristoleic acid	FAHFA(20:4-O-14:1)	C34H56O4
548	601.5	250.2	(+)	35	EPAHMO	Eicosapentaenoic acid-hydroxy myristoleic acid	FAHFA(20:5-O-14:1)	C34H54O4
549	639.5	250.2	(+)	35	BAHMO	Behenic acid-hydroxy myristoleic acid	FAHFA(22:0-O-14:1)	C36H68O4
550	637.5	250.2	(+)	35	DEAHMO	Docosenoic acid-hydroxy myristoleic acid	FAHFA(22:1-O-14:1)	C36H66O4
551	635.5	250.2	(+)	35	DDAHMO	Docosadienoate-hydroxy myristoleic acid	FAHFA(22:2-O-14:1)	C36H64O4
552	627.5	250.2	(+)	35	DHAHMO	Docosahexaenoic acid-hydroxy myristoleic acid	FAHFA(22:6-O-14:1)	C36H56O4
553	525.5	248.2	(+)	35	MAHTDA	Myristic acid-hydroxy tetradecanoic acid	FAHFA(14:0-O-14:2)	C28H50O4
554	523.5	248.2	(+)	35	MOHTDA	Myristoleic acid-hydroxy tetradecanoic acid	FAHFA(14:1-O-14:2)	C28H48O4
555	539.5	248.2	(+)	35	PDAHTDA	Pentadecanoic acid-hydroxy tetradecanoic acid	FAHFA(15:0-O-14:2)	C29H52O4
556	537.5	248.2	(+)	35	PDEAHTDA	Pentadecenoic acid-hydroxy tetradecanoic acid	FAHFA(15:1-O-14:2)	C29H50O4
557	551.5	248.2	(+)	35	POHTDA	Palmitoleic acid-hydroxy tetradecanoic acid	FAHFA(16:1-O-14:2)	C30H52O4
558	553.5	248.2	(+)	35	PAHTDA	Palmitic acid-hydroxy tetradecanoic acid	FAHFA(16:0-O-14:2)	C30H54O4
559	567.5	248.2	(+)	35	HDAHTDA	Heptadecanoic acid-hydroxy tetradecanoic acid	FAHFA(17:0-O-14:2)	C31H56O4
560	565.5	248.2	(+)	35	HDEAHTDA	Heptadecenoic acid-hydroxy tetradecanoic acid	FAHFA(17:1-O-14:2)	C31H54O4
561	581.5	248.2	(+)	35	SAHTDA	Stearic acid-hydroxy tetradecanoic acid	FAHFA(18:0-O-14:2)	C32H58O4
562	579.5	248.2	(+)	35	OAHTDA	Oleic acid-hydroxy tetradecanoic acid	FAHFA(18:1-O-14:2)	C32H56O4
563	577.5	248.2	(+)	35	LAHTDA	Linoleic acid-hydroxy tetradecanoic acid	FAHFA(18:2-O-14:2)	C32H54O4
564	575.5	248.2	(+)	35	ALAHTDA	α-Linolenic acid-hydroxy tetradecanoic acid	FAHFA(18:3-O-14:2)	C32H52O4
565	573.5	248.2	(+)	35	SDAHTDA	Stearidonic acid-hydroxy tetradecanoic acid	FAHFA(18:4-O-14:2)	C32H50O4
566	609.5	248.2	(+)	35	AAHTDA	Arachidic acid-hydroxy tetradecanoic acid	FAHFA(20:0-O-14:2)	C34H62O4
567	607.5	248.2	(+)	35	EAHTDA	Eicosenoic acid-hydroxy tetradecanoic acid	FAHFA(20:1-O-14:2)	C34H60O4
568	605.5	248.2	(+)	35	EDAHTDA	Eicosadienoic acid-hydroxy tetradecanoic acid	FAHFA(20:2-O-14:2)	C34H58O4
569	603.5	248.2	(+)	35	ETAHTDA	Eicosatrienoic acid-hydroxy tetradecanoic acid	FAHFA(20:3-O-14:2)	C34H56O4
570	601.5	248.2	(+)	35	ARAHTDA	Arachidonic acid-hydroxy tetradecanoic acid	FAHFA(20:4-O-14:2)	C34H54O4
571	599.5	248.2	(+)	35	EPAHTDA	Eicosapentaenoic acid-hydroxy tetradecanoic acid	FAHFA(20:5-O-14:2)	C34H52O4
572	637.5	248.2	(+)	35	BAHTDA	Behenic acid-hydroxy tetradecanoic acid	FAHFA(22:0-O-14:2)	C36H66O4
573	635.5	248.2	(+)	35	DEAHTDA	Docosenoic acid-hydroxy tetradecanoic acid	FAHFA(22:1-O-14:2)	C36H64O4
574	633.5	248.2	(+)	35	DDAHTDA	Docosadienoate-hydroxy tetradecanoic acid	FAHFA(22:2-O-14:2)	C36H62O4
575	625.5	248.2	(+)	35	DHAHTDA	Docosahexaenoic acid-hydroxy tetradecanoic acid	FAHFA(22:6-O-14:2)	C36H54O4
576	523.5	246.2	(+)	35	MAHTTA	Myristic acid-hydroxy tetradecatrienoic acid	FAHFA(14:0-O-14:3)	C28H48O4
577	521.5	246.2	(+)	35	MOHTTA	Myristoleic acid-hydroxy tetradecatrienoic acid	FAHFA(14:1-O-14:3)	C28H46O4
578	537.5	246.2	(+)	35	PDAHTTA	Pentadecanoic acid-hydroxy tetradecatrienoic acid	FAHFA(15:0-O-14:3)	C29H50O4
579	535.5	246.2	(+)	35	PDEAHTTA	Pentadecenoic acid-hydroxy tetradecatrienoic acid	FAHFA(15:1-O-14:3)	C29H48O4
580	549.5	246.2	(+)	35	POHTTA	Palmitoleic acid-hydroxy tetradecatrienoic acid	FAHFA(16:1-O-14:3)	C30H50O4
581	551.5	246.2	(+)	35	PAHTTA	Palmitic acid-hydroxy tetradecatrienoic acid	FAHFA(16:0-O-14:3)	C30H52O4
582	565.5	246.2	(+)	35	HDAHTTA	Heptadecanoic acid-hydroxy tetradecatrienoic acid	FAHFA(17:0-O-14:3)	C31H54O4
583	563.5	246.2	(+)	35	HDEAHTTA	Heptadecenoic acid-hydroxy tetradecatrienoic acid	FAHFA(17:1-O-14:3)	C31H52O4
584	579.5	246.2	(+)	35	SAHTTA	Stearic acid-hydroxy tetradecatrienoic acid	FAHFA(18:0-O-14:3)	C32H56O4
585	577.5	246.2	(+)	35	OAHTTA	Oleic acid-hydroxy tetradecatrienoic acid	FAHFA(18:1-O-14:3)	C32H54O4
586	575.5	246.2	(+)	35	LAHTTA	Linoleic acid-hydroxy tetradecatrienoic acid	FAHFA(18:2-O-14:3)	C32H52O4
587	573.5	246.2	(+)	35	ALAHTTA	α-Linolenic acid-hydroxy tetradecatrienoic acid	FAHFA(18:3-O-14:3)	C32H50O4
588	571.5	246.2	(+)	35	SDAHTTA	Stearidonic acid-hydroxy tetradecatrienoic acid	FAHFA(18:4-O-14:3)	C32H48O4
589	607.5	246.2	(+)	35	AAHTTA	Arachidic acid-hydroxy tetradecatrienoic acid	FAHFA(20:0-O-14:3)	C34H60O4
590	605.5	246.2	(+)	35	EAHTTA	Eicosenoic acid-hydroxy tetradecatrienoic acid	FAHFA(20:1-O-14:3)	C34H58O4
591	603.5	246.2	(+)	35	EDAHTTA	Eicosadienoic acid-hydroxy tetradecatrienoic acid	FAHFA(20:2-O-14:3)	C34H56O4
592	601.5	246.2	(+)	35	ETAHTTA	Eicosatrienoic acid-hydroxy tetradecatrienoic acid	FAHFA(20:3-O-14:3)	C34H54O4
593	599.5	246.2	(+)	35	ARAHTTA	Arachidonic acid-hydroxy tetradecatrienoic acid	FAHFA(20:4-O-14:3)	C34H52O4
594	597.5	246.2	(+)	35	EPAHTTA	Eicosapentaenoic acid-hydroxy tetradecatrienoic acid	FAHFA(20:5-O-14:3)	C34H50O4
595	635.5	246.2	(+)	35	BAHTTA	Behenic acid-hydroxy tetradecatrienoic acid	FAHFA(22:0-O-14:3)	C36H64O4
596	633.5	246.2	(+)	35	DEAHTTA	Docosenoic acid-hydroxy tetradecatrienoic acid	FAHFA(22:1-O-14:3)	C36H62O4
597	631.5	246.2	(+)	35	DDAHTTA	Docosadienoate-hydroxy tetradecatrienoic acid	FAHFA(22:2-O-14:3)	C36H60O4
598	623.5	246.2	(+)	35	DHAHTTA	Docosahexaenoic acid-hydroxy tetradecatrienoic acid	FAHFA(22:6-O-14:3)	C36H52O4
599	555.5	278.2	(+)	35	MAHPO	Myristic acid-hydroxy palmitoleic acid	FAHFA(14:0-O-16:1)	C30H56O4
600	553.5	278.2	(+)	35	MOHPO	Myristoleic acid-hydroxy palmitoleic acid	FAHFA(14:1-O-16:1)	C30H54O4
601	569.5	278.2	(+)	35	PDAHPO	Pentadecanoic acid-hydroxy palmitoleic acid	FAHFA(15:0-O-16:1)	C31H58O4
602	567.5	278.2	(+)	35	PDEAHPO	Pentadecenoic acid-hydroxy palmitoleic acid	FAHFA(15:1-O-16:1)	C31H56O4
603	581.5	278.2	(+)	35	POHPO	Palmitoleic acid-hydroxy palmitoleic acid	FAHFA(16:1-O-16:1)	C32H58O4
604	583.5	278.2	(+)	35	PAHPO	Palmitic acid-hydroxy palmitoleic acid	FAHFA(16:0-O-16:1)	C32H60O4
605	597.5	278.2	(+)	35	HDAHPO	Heptadecanoic acid-hydroxy palmitoleic acid	FAHFA(17:0-O-16:1)	C33H62O4
606	595.5	278.2	(+)	35	HDEAHPO	Heptadecenoic acid-hydroxy palmitoleic acid	FAHFA(17:1-O-16:1)	C33H60O4
607	611.5	278.2	(+)	35	SAHPO	Stearic acid-hydroxy palmitoleic acid	FAHFA(18:0-O-16:1)	C34H64O4
608	609.5	278.2	(+)	35	OAHPO	Oleic acid-hydroxy palmitoleic acid	FAHFA(18:1-O-16:1)	C34H62O4
609	607.5	278.2	(+)	35	LAHPO	Linoleic acid-hydroxy palmitoleic acid	FAHFA(18:2-O-16:1)	C34H60O4
610	605.5	278.2	(+)	35	ALAHPO	α-Linolenic acid-hydroxy palmitoleic acid	FAHFA(18:3-O-16:1)	C34H58O4
611	603.5	278.2	(+)	35	SDAHPO	Stearidonic acid-hydroxy palmitoleic acid	FAHFA(18:4-O-16:1)	C34H56O4
612	639.5	278.2	(+)	35	AAHPO	Arachidic acid-hydroxy palmitoleic acid	FAHFA(20:0-O-16:1)	C36H68O4
613	637.5	278.2	(+)	35	EAHPO	Eicosenoic acid-hydroxy palmitoleic acid	FAHFA(20:1-O-16:1)	C36H66O4
614	635.5	278.2	(+)	35	EDAHPO	Eicosadienoic acid-hydroxy palmitoleic acid	FAHFA(20:2-O-16:1)	C36H64O4
615	633.5	278.2	(+)	35	ETAHPO	Eicosatrienoic acid-hydroxy palmitoleic acid	FAHFA(20:3-O-16:1)	C36H62O4
616	631.5	278.2	(+)	35	ARAHPO	Arachidonic acid-hydroxy palmitoleic acid	FAHFA(20:4-O-16:1)	C36H60O4
617	629.5	278.2	(+)	35	EPAHPO	Eicosapentaenoic acid-hydroxy palmitoleic acid	FAHFA(20:5-O-16:1)	C36H58O4
618	667.5	278.2	(+)	35	BAHPO	Behenic acid-hydroxy palmitoleic acid	FAHFA(22:0-O-16:1)	C38H72O4
619	665.5	278.2	(+)	35	DEAHPO	Docosenoic acid-hydroxy palmitoleic acid	FAHFA(22:1-O-16:1)	C38H70O4
620	663.5	278.2	(+)	35	DDAHPO	Docosadienoate-hydroxy palmitoleic acid	FAHFA(22:2-O-16:1)	C38H68O4
621	655.4	278.2	(+)	35	DHAHPO	Docosahexaenoic acid-hydroxy palmitoleic acid	FAHFA(22:6-O-16:1)	C38H60O4
622	553.5	276.2	(+)	35	MAHHDA	Myristic acid-hydroxy hexadecadienoic acid	FAHFA(14:0-O-16:2)	C30H54O4
623	551.5	276.2	(+)	35	MOHHDA	Myristoleic acid-hydroxy hexadecadienoic acid	FAHFA(14:1-O-16:2)	C30H52O4
624	567.5	276.2	(+)	35	PDAHHDA	Pentadecanoic acid-hydroxy hexadecadienoic acid	FAHFA(15:0-O-16:2)	C31H56O4

625	565.5	276.2	(+)	35	PDEAHHDA	Pentadecenoic acid-hydroxy hexadecadienoic acid	FAHFA(15:1-O-16:2)	C31H54O4
626	579.5	276.2	(+)	35	POHHDA	Palmitoleic acid-hydroxy hexadecadienoic acid	FAHFA(16:1-O-16:2)	C32H56O4
627	581.5	276.2	(+)	35	PAHHDA	Palmitic acid-hydroxy hexadecadienoic acid	FAHFA(16:0-O-16:2)	C32H58O4
628	595.5	276.2	(+)	35	HDAHHDA	Heptadecanoic acid-hydroxy hexadecadienoic acid	FAHFA(17:0-O-16:2)	C33H60O4
629	593.5	276.2	(+)	35	HDEAHHDA	Heptadecenoic acid-hydroxy hexadecadienoic acid	FAHFA(17:1-O-16:2)	C33H58O4
630	609.5	276.2	(+)	35	SAHHDA	Stearic acid-hydroxy hexadecadienoic acid	FAHFA(18:0-O-16:2)	C34H62O4
631	607.5	276.2	(+)	35	OAHHDA	Oleic acid-hydroxy hexadecadienoic acid	FAHFA(18:1-O-16:2)	C34H60O4
632	605.5	276.2	(+)	35	LAHHDA	Linoleic acid-hydroxy hexadecadienoic acid	FAHFA(18:2-O-16:2)	C34H58O4
633	603.5	276.2	(+)	35	ALAHHDA	α-Linolenic acid-hydroxy hexadecadienoic acid	FAHFA(18:3-O-16:2)	C34H56O4
634	601.5	276.2	(+)	35	SDAHHDA	Stearidonic acid-hydroxy hexadecadienoic acid	FAHFA(18:4-O-16:2)	C34H54O4
635	637.5	276.2	(+)	35	AAHHDA	Arachidic acid-hydroxy hexadecadienoic acid	FAHFA(20:0-O-16:2)	C36H66O4
636	635.5	276.2	(+)	35	EAHHDA	Eicosenoic acid-hydroxy hexadecadienoic acid	FAHFA(20:1-O-16:2)	C36H64O4
637	633.5	276.2	(+)	35	EDAHHDA	Eicosadienoic acid-hydroxy hexadecadienoic acid	FAHFA(20:2-O-16:2)	C36H62O4
638	631.5	276.2	(+)	35	ETAHHDA	Eicosatrienoic acid-hydroxy hexadecadienoic acid	FAHFA(20:3-O-16:2)	C36H60O4
639	629.5	276.2	(+)	35	ARAHHDA	Arachidonic acid-hydroxy hexadecadienoic acid	FAHFA(20:4-O-16:2)	C36H58O4
640	627.5	276.2	(+)	35	EPAHHDA	Eicosapentaenoic acid-hydroxy hexadecadienoic acid	FAHFA(20:5-O-16:2)	C36H56O4
641	665.5	276.2	(+)	35	BAHHDA	Behenic acid-hydroxy hexadecadienoic acid	FAHFA(22:0-O-16:2)	C38H70O4
642	663.5	276.2	(+)	35	DEAHHDA	Docosenoic acid-hydroxy hexadecadienoic acid	FAHFA(22:1-O-16:2)	C38H68O4
643	661.5	276.2	(+)	35	DDAHHDA	Docosadienoate-hydroxy hexadecadienoic acid	FAHFA(22:2-O-16:2)	C38H66O4
644	653.5	276.2	(+)	35	DHAHHDA	Docosahexaenoic acid-hydroxy hexadecadienoic acid	FAHFA(22:6-O-16:2)	C38H58O4
645	551.5	274.2	(+)	35	MAHHTA	Myristic acid-hydroxy hexadecatrienoic acid	FAHFA(14:0-O-16:3)	C30H52O4
646	549.5	274.2	(+)	35	MOHHTA	Myristoleic acid-hydroxy hexadecatrienoic acid	FAHFA(14:1-O-16:3)	C30H50O4
647	565.5	274.2	(+)	35	PDAHHTA	Pentadecanoic acid-hydroxy hexadecatrienoic acid	FAHFA(15:0-O-16:3)	C31H54O4
648	563.5	274.2	(+)	35	PDEAHHTA	Pentadecenoic acid-hydroxy hexadecatrienoic acid	FAHFA(15:1-O-16:3)	C31H52O4
649	577.5	274.2	(+)	35	POHHTA	Palmitoleic acid-hydroxy hexadecatrienoic acid	FAHFA(16:1-O-16:3)	C32H54O4
650	579.5	274.2	(+)	35	PAHHTA	Palmitic acid-hydroxy hexadecatrienoic acid	FAHFA(16:0-O-16:3)	C32H56O4
651	593.5	274.2	(+)	35	HDAHHTA	Heptadecanoic acid-hydroxy hexadecatrienoic acid	FAHFA(17:0-O-16:3)	C33H58O4
652	591.5	274.2	(+)	35	HDEAHHTA	Heptadecenoic acid-hydroxy hexadecatrienoic acid	FAHFA(17:1-O-16:3)	C33H56O4
653	607.5	274.2	(+)	35	SAHHTA	Stearic acid-hydroxy hexadecatrienoic acid	FAHFA(18:0-O-16:3)	C34H60O4
654	605.5	274.2	(+)	35	OAHHTA	Oleic acid-hydroxy hexadecatrienoic acid	FAHFA(18:1-O-16:3)	C34H58O4
655	603.5	274.2	(+)	35	LAHHTA	Linoleic acid-hydroxy hexadecatrienoic acid	FAHFA(18:2-O-16:3)	C34H56O4
656	601.5	274.2	(+)	35	ALAHHTA	α-Linolenic acid-hydroxy hexadecatrienoic acid	FAHFA(18:3-O-16:3)	C34H54O4
657	599.5	274.2	(+)	35	SDAHHTA	Stearidonic acid-hydroxy hexadecatrienoic acid	FAHFA(18:4-O-16:3)	C34H52O4
658	635.5	274.2	(+)	35	AAHHTA	Arachidic acid-hydroxy hexadecatrienoic acid	FAHFA(20:0-O-16:3)	C36H64O4
659	633.5	274.2	(+)	35	EAHHTA	Eicosenoic acid-hydroxy hexadecatrienoic acid	FAHFA(20:1-O-16:3)	C36H62O4
660	631.5	274.2	(+)	35	EDAHHTA	Eicosadienoic acid-hydroxy hexadecatrienoic acid	FAHFA(20:2-O-16:3)	C36H60O4
661	629.5	274.2	(+)	35	ETAHHTA	Eicosatrienoic acid-hydroxy hexadecatrienoic acid	FAHFA(20:3-O-16:3)	C36H58O4
662	627.5	274.2	(+)	35	ARAHHTA	Arachidonic acid-hydroxy hexadecatrienoic acid	FAHFA(20:4-O-16:3)	C36H56O4
663	625.5	274.2	(+)	35	EPAHHTA	Eicosapentaenoic acid-hydroxy hexadecatrienoic acid	FAHFA(20:5-O-16:3)	C36H54O4
664	663.5	274.2	(+)	35	BAHHTA	Behenic acid-hydroxy hexadecatrienoic acid	FAHFA(22:0-O-16:3)	C38H68O4
665	661.5	274.2	(+)	35	DEAHHTA	Docosenoic acid-hydroxy hexadecatrienoic acid	FAHFA(22:1-O-16:3)	C38H66O4
666	659.5	274.2	(+)	35	DDAHHTA	Docosadienoate-hydroxy hexadecatrienoic acid	FAHFA(22:2-O-16:3)	C38H64O4
667	651.5	274.2	(+)	35	DHAHHTA	Docosahexaenoic acid-hydroxy hexadecatrienoic acid	FAHFA(22:6-O-16:3)	C38H56O4
668	583.5	306.3	(+)	35	MAHOA	Myristic acid-hydroxy oleic acid	FAHFA(14:0-O-18:1)	C32H60O4
669	581.5	306.3	(+)	35	MOHOA	Myristoleic acid-hydroxy oleic acid	FAHFA(14:1-O-18:1)	C32H58O4
670	597.5	306.3	(+)	35	PDAHOA	Pentadecanoic acid-hydroxy oleic acid	FAHFA(15:0-O-18:1)	C33H62O4
671	595.5	306.3	(+)	35	PDEAHOA	Pentadecenoic acid-hydroxy oleic acid	FAHFA(15:1-O-18:1)	C33H60O4
672	609.5	306.3	(+)	35	POHOA	Palmitoleic acid-hydroxy oleic acid	FAHFA(16:1-O-18:1)	C34H62O4
673	611.5	306.3	(+)	35	PAHOA	Palmitic acid-hydroxy oleic acid	FAHFA(16:0-O-18:1)	C34H64O4
674	625.5	306.3	(+)	35	HDAHOA	Heptadecanoic acid-hydroxy oleic acid	FAHFA(17:0-O-18:1)	C35H66O4
675	623.5	306.3	(+)	35	HDEAHOA	Heptadecenoic acid-hydroxy oleic acid	FAHFA(17:1-O-18:1)	C35H64O4
676	639.5	306.3	(+)	35	SAHOA	Stearic acid-hydroxy oleic acid	FAHFA(18:0-O-18:1)	C36H68O4
677	637.5	306.3	(+)	35	OAHOA	Oleic acid-hydroxy oleic acid	FAHFA(18:1-O-18:1)	C36H66O4
678	635.5	306.3	(+)	35	LAHOA	Linoleic acid-hydroxy oleic acid	FAHFA(18:2-O-18:1)	C36H64O4
679	633.5	306.3	(+)	35	ALAHOA	α-Linolenic acid-hydroxy oleic acid	FAHFA(18:3-O-18:1)	C36H62O4
680	631.5	306.3	(+)	35	SDAHOA	Stearidonic acid-hydroxy oleic acid	FAHFA(18:4-O-18:1)	C36H60O4
681	667.5	306.3	(+)	35	AAHOA	Arachidic acid-hydroxy oleic acid	FAHFA(20:0-O-18:1)	C38H72O4
682	665.5	306.3	(+)	35	EAHOA	Eicosenoic acid-hydroxy oleic acid	FAHFA(20:1-O-18:1)	C38H70O4
683	663.5	306.3	(+)	35	EDAHOA	Eicosadienoic acid-hydroxy oleic acid	FAHFA(20:2-O-18:1)	C38H68O4
684	661.5	306.3	(+)	35	ETAHOA	Eicosatrienoic acid-hydroxy oleic acid	FAHFA(20:3-O-18:1)	C38H66O4
685	659.5	306.3	(+)	35	ARAHOA	Arachidonic acid-hydroxy oleic acid	FAHFA(20:4-O-18:1)	C38H64O4
686	657.5	306.3	(+)	35	EPAHOA	Eicosapentaenoic acid-hydroxy oleic acid	FAHFA(20:5-O-18:1)	C38H62O4
687	695.5	306.3	(+)	35	BAHOA	Behenic acid-hydroxy oleic acid	FAHFA(22:0-O-18:1)	C40H76O4
688	693.5	306.3	(+)	35	DEAHOA	Docosenoic acid-hydroxy oleic acid	FAHFA(22:1-O-18:1)	C40H74O4
689	691.5	306.3	(+)	35	DDAHOA	Docosadienoate-hydroxy oleic acid	FAHFA(22:2-O-18:1)	C40H72O4
690	683.5	306.3	(+)	35	DHAHOA	Docosahexaenoic acid-hydroxy oleic acid	FAHFA(22:6-O-18:1)	C40H64O4
691	581.5	304.3	(+)	35	MAHLA	Myristic acid-hydroxy linoleic acid	FAHFA(14:0-O-18:2)	C32H58O4
692	579.5	304.3	(+)	35	MOHLA	Myristoleic acid-hydroxy linoleic acid	FAHFA(14:1-O-18:2)	C32H56O4
693	595.5	304.3	(+)	35	PDAHLA	Pentadecanoic acid-hydroxy linoleic acid	FAHFA(15:0-O-18:2)	C33H60O4
694	593.5	304.3	(+)	35	PDEAHLA	Pentadecenoic acid-hydroxy linoleic acid	FAHFA(15:1-O-18:2)	C33H58O4
695	607.5	304.3	(+)	35	POHLA	Palmitoleic acid-hydroxy linoleic acid	FAHFA(16:1-O-18:2)	C34H60O4
696	609.5	304.3	(+)	35	PAHLA	Palmitic acid-hydroxy linoleic acid	FAHFA(16:0-O-18:2)	C34H62O4
697	623.5	304.3	(+)	35	HDAHLA	Heptadecanoic acid-hydroxy linoleic acid	FAHFA(17:0-O-18:2)	C35H64O4
698	621.5	304.3	(+)	35	HDEAHLA	Heptadecenoic acid-hydroxy linoleic acid	FAHFA(17:1-O-18:2)	C35H62O4
699	637.5	304.3	(+)	35	SAHLA	Stearic acid-hydroxy linoleic acid	FAHFA(18:0-O-18:2)	C36H66O4
700	635.5	304.3	(+)	35	OAHLA	Oleic acid-hydroxy linoleic acid	FAHFA(18:1-O-18:2)	C36H64O4
701	633.5	304.3	(+)	35	LAHLA	Linoleic acid-hydroxy linoleic acid	FAHFA(18:2-O-18:2)	C36H62O4
702	631.5	304.3	(+)	35	ALAHLA	α-Linolenic acid-hydroxy linoleic acid	FAHFA(18:3-O-18:2)	C36H60O4
703	629.5	304.3	(+)	35	SDAHLA	Stearidonic acid-hydroxy linoleic acid	FAHFA(18:4-O-18:2)	C36H58O4
704	665.5	304.3	(+)	35	AAHLA	Arachidic acid-hydroxy linoleic acid	FAHFA(20:0-O-18:2)	C38H70O4
705	663.5	304.3	(+)	35	EAHLA	Eicosenoic acid-hydroxy linoleic acid	FAHFA(20:1-O-18:2)	C38H68O4
706	661.5	304.3	(+)	35	EDAHLA	Eicosadienoic acid-hydroxy linoleic acid	FAHFA(20:2-O-18:2)	C38H66O4
707	659.5	304.3	(+)	35	ETAHLA	Eicosatrienoic acid-hydroxy linoleic acid	FAHFA(20:3-O-18:2)	C38H64O4
708	657.5	304.3	(+)	35	ARAHLA	Arachidonic acid-hydroxy linoleic acid	FAHFA(20:4-O-18:2)	C38H62O4
709	655.5	304.3	(+)	35	EPAHLA	Eicosapentaenoic acid-hydroxy linoleic acid	FAHFA(20:5-O-18:2)	C38H60O4
710	693.5	304.3	(+)	35	BAHLA	Behenic acid-hydroxy linoleic acid	FAHFA(22:0-O-18:2)	C40H74O4
711	691.5	304.3	(+)	35	DEAHLA	Docosenoic acid-hydroxy linoleic acid	FAHFA(22:1-O-18:2)	C40H72O4
712	689.5	304.3	(+)	35	DDAHLA	Docosadienoate-hydroxy linoleic acid	FAHFA(22:2-O-18:2)	C40H70O4
713	681.5	304.3	(+)	35	DHAHLA	Docosahexaenoic acid-hydroxy linoleic acid	FAHFA(22:6-O-18:2)	C40H62O4
714	579.5	302.2	(+)	35	MAHALA	Myristic acid-hydroxy α-Linolenic acid	FAHFA(14:0-O-18:3)	C32H56O4
715	577.5	302.2	(+)	35	MOHALA	Myristoleic acid-hydroxy α-Linolenic acid	FAHFA(14:1-O-18:3)	C32H54O4
716	593.5	302.2	(+)	35	PDAHALA	Pentadecanoic acid-hydroxy α-Linolenic acid	FAHFA(15:0-O-18:3)	C33H58O4
717	591.5	302.2	(+)	35	PDEAHALA	Pentadecenoic acid-hydroxy α-Linolenic acid	FAHFA(15:1-O-18:3)	C33H56O4
718	605.5	302.2	(+)	35	POHALA	Palmitoleic acid-hydroxy α-Linolenic acid	FAHFA(16:1-O-18:3)	C34H58O4
719	607.5	302.2	(+)	35	PAHALA	Palmitic acid-hydroxy α-Linolenic acid	FAHFA(16:0-O-18:3)	C34H60O4
720	621.5	302.2	(+)	35	HDAHALA	Heptadecanoic acid-hydroxy α-Linolenic acid	FAHFA(17:0-O-18:3)	C35H62O4
721	619.5	302.2	(+)	35	HDEAHALA	Heptadecenoic acid-hydroxy α-Linolenic acid	FAHFA(17:1-O-18:3)	C35H60O4
722	635.5	302.2	(+)	35	SAHALA	Stearic acid-hydroxy α-Linolenic acid	FAHFA(18:0-O-18:3)	C36H64O4
723	633.5	302.2	(+)	35	OAHALA	Oleic acid-hydroxy α-Linolenic acid	FAHFA(18:1-O-18:3)	C36H62O4
724	631.5	302.2	(+)	35	LAHALA	Linoleic acid-hydroxy α-Linolenic acid	FAHFA(18:2-O-18:3)	C36H60O4
725	629.5	302.2	(+)	35	ALAHALA	α-Linolenic acid-hydroxy α-Linolenic acid	FAHFA(18:3-O-18:3)	C36H58O4
726	627.5	302.2	(+)	35	SDAHALA	Stearidonic acid-hydroxy α-Linolenic acid	FAHFA(18:4-O-18:3)	C36H56O4
727	663.5	302.2	(+)	35	AAHALA	Arachidic acid-hydroxy α-Linolenic acid	FAHFA(20:0-O-18:3)	C38H68O4
728	661.5	302.2	(+)	35	EAHALA	Eicosenoic acid-hydroxy α-Linolenic acid	FAHFA(20:1-O-18:3)	C38H66O4
729	659.5	302.2	(+)	35	EDAHALA	Eicosadienoic acid-hydroxy α-Linolenic acid	FAHFA(20:2-O-18:3)	C38H64O4

730	657.5	302.2	(+)	35	ETAHALA	Eicosatrienoic acid-hydroxy α-Linolenic acid	FAHFA(20:3-O-18:3)	C38H62O4
731	655.5	302.2	(+)	35	ARAHALA	Arachidonic acid-hydroxy α-Linolenic acid	FAHFA(20:4-O-18:3)	C38H60O4
732	653.5	302.2	(+)	35	EPAHALA	Eicosapentaenoic acid-hydroxy α-Linolenic acid	FAHFA(20:5-O-18:3)	C38H58O4
733	691.5	302.2	(+)	35	BAHALA	Behenic acid-hydroxy α-Linolenic acid	FAHFA(22:0-O-18:3)	C40H72O4
734	689.5	302.2	(+)	35	DEAHALA	Docosenoic acid-hydroxy α-Linolenic acid	FAHFA(22:1-O-18:3)	C40H70O4
735	687.5	302.2	(+)	35	DDAHALA	Docosadienoate-hydroxy α-Linolenic acid	FAHFA(22:2-O-18:3)	C40H68O4
736	679.5	302.2	(+)	35	DHAHALA	Docosahexaenoic acid-hydroxy α-Linolenic acid	FAHFA(22:6-O-18:3)	C40H60O4
737	611.5	334.3	(+)	35	MAHEA	Myristic acid-hydroxy eicosenoic acid	FAHFA(14:0-O-20:1)	C34H64O4
738	609.5	334.3	(+)	35	MOHEA	Myristoleic acid-hydroxy eicosenoic acid	FAHFA(14:1-O-20:1)	C34H62O4
739	625.5	334.3	(+)	35	PDAHEA	Pentadecanoic acid-hydroxy eicosenoic acid	FAHFA(15:0-O-20:1)	C35H66O4
740	623.5	334.3	(+)	35	PDEAHEA	Pentadecenoic acid-hydroxy eicosenoic acid	FAHFA(15:1-O-20:1)	C35H64O4
741	637.5	334.3	(+)	35	POHEA	Palmitoleic acid-hydroxy eicosenoic acid	FAHFA(16:1-O-20:1)	C36H66O4
742	639.5	334.3	(+)	35	PAHEA	Palmitic acid-hydroxy eicosenoic acid	FAHFA(16:0-O-20:1)	C36H68O4
743	653.5	334.3	(+)	35	HDAHEA	Heptadecanoic acid-hydroxy eicosenoic acid	FAHFA(17:0-O-20:1)	C37H70O4
744	651.5	334.3	(+)	35	HDEAHEA	Heptadecenoic acid-hydroxy eicosenoic acid	FAHFA(17:1-O-20:1)	C37H68O4
745	667.5	334.3	(+)	35	SAHEA	Stearic acid-hydroxy eicosenoic acid	FAHFA(18:0-O-20:1)	C38H72O4
746	665.5	334.3	(+)	35	OAHEA	Oleic acid-hydroxy eicosenoic acid	FAHFA(18:1-O-20:1)	C38H70O4
747	663.5	334.3	(+)	35	LAHEA	Linoleic acid-hydroxy eicosenoic acid	FAHFA(18:2-O-20:1)	C38H68O4
748	661.5	334.3	(+)	35	ALAHEA	α-Linolenic acid-hydroxy eicosenoic acid	FAHFA(18:3-O-20:1)	C38H66O4
749	659.5	334.3	(+)	35	SDAHEA	Stearidonic acid-hydroxy eicosenoic acid	FAHFA(18:4-O-20:1)	C38H64O4
750	695.5	334.3	(+)	35	AAHEA	Arachidic acid-hydroxy eicosenoic acid	FAHFA(20:0-O-20:1)	C40H76O4
751	693.5	334.3	(+)	35	EAHEA	Eicosenoic acid-hydroxy eicosenoic acid	FAHFA(20:1-O-20:1)	C40H74O4
752	691.5	334.3	(+)	35	EDAHEA	Eicosadienoic acid-hydroxy eicosenoic acid	FAHFA(20:2-O-20:1)	C40H72O4
753	689.5	334.3	(+)	35	ETAHEA	Eicosatrienoic acid-hydroxy eicosenoic acid	FAHFA(20:3-O-20:1)	C40H70O4
754	687.5	334.3	(+)	35	ARAHEA	Arachidonic acid-hydroxy eicosenoic acid	FAHFA(20:4-O-20:1)	C40H68O4
755	685.5	334.3	(+)	35	EPAHEA	Eicosapentaenoic acid-hydroxy eicosenoic acid	FAHFA(20:5-O-20:1)	C40H66O4
756	723.5	334.3	(+)	35	BAHEA	Behenic acid-hydroxy eicosenoic acid	FAHFA(22:0-O-20:1)	C42H80O4
757	721.5	334.3	(+)	35	DEAHEA	Docosenoic acid-hydroxy eicosenoic acid	FAHFA(22:1-O-20:1)	C42H78O4
758	719.5	334.3	(+)	35	DDAHEA	Docosadienoate-hydroxy eicosenoic acid	FAHFA(22:2-O-20:1)	C40H76O4
759	711.5	334.3	(+)	35	DHAHEA	Docosahexaenoic acid-hydroxy eicosenoic acid	FAHFA(22:6-O-20:1)	C42H68O4
760	609.5	332.3	(+)	35	MAHEDA	Myristic acid-hydroxy eicosadienoic acid	FAHFA(14:0-O-20:2)	C34H62O4
761	607.5	332.3	(+)	35	MOHEDA	Myristoleic acid-hydroxy eicosadienoic acid	FAHFA(14:1-O-20:2)	C34H60O4
762	623.5	332.3	(+)	35	PDAHEDA	Pentadecanoic acid-hydroxy eicosadienoic acid	FAHFA(15:0-O-20:2)	C35H64O4
763	621.5	332.3	(+)	35	PDEAHEDA	Pentadecenoic acid-hydroxy eicosadienoic acid	FAHFA(15:1-O-20:2)	C35H62O4
764	635.5	332.3	(+)	35	POHEDA	Palmitoleic acid-hydroxy eicosadienoic acid	FAHFA(16:1-O-20:2)	C36H64O4
765	637.5	332.3	(+)	35	PAHEDA	Palmitic acid-hydroxy eicosadienoic acid	FAHFA(16:0-O-20:2)	C36H66O4
766	651.5	332.3	(+)	35	HDAHEDA	Heptadecanoic acid-hydroxy eicosadienoic acid	FAHFA(17:0-O-20:2)	C37H68O4
767	649.5	332.3	(+)	35	HDEAHEDA	Heptadecenoic acid-hydroxy eicosadienoic acid	FAHFA(17:1-O-20:2)	C37H66O4
768	665.5	332.3	(+)	35	SAHEDA	Stearic acid-hydroxy eicosadienoic acid	FAHFA(18:0-O-20:2)	C38H70O4
769	663.5	332.3	(+)	35	OAHEDA	Oleic acid-hydroxy eicosadienoic acid	FAHFA(18:1-O-20:2)	C38H68O4
770	661.5	332.3	(+)	35	LAHEDA	Linoleic acid-hydroxy eicosadienoic acid	FAHFA(18:2-O-20:2)	C38H66O4
771	659.5	332.3	(+)	35	ALAHEDA	α-Linolenic acid-hydroxy eicosadienoic acid	FAHFA(18:3-O-20:2)	C38H64O4
772	657.5	332.3	(+)	35	SDAHEDA	Stearidonic acid-hydroxy eicosadienoic acid	FAHFA(18:4-O-20:2)	C38H62O4
773	693.5	332.3	(+)	35	AAHEDA	Arachidic acid-hydroxy eicosadienoic acid	FAHFA(20:0-O-20:2)	C40H74O4
774	691.5	332.3	(+)	35	EAHEDA	Eicosenoic acid-hydroxy eicosadienoic acid	FAHFA(20:1-O-20:2)	C40H72O4
775	689.5	332.3	(+)	35	EDAHEDA	Eicosadienoic acid-hydroxy eicosadienoic acid	FAHFA(20:2-O-20:2)	C40H70O4
776	687.5	332.3	(+)	35	ETAHEDA	Eicosatrienoic acid-hydroxy eicosadienoic acid	FAHFA(20:3-O-20:2)	C40H68O4
777	685.5	332.3	(+)	35	ARAHEDA	Arachidonic acid-hydroxy eicosadienoic acid	FAHFA(20:4-O-20:2)	C40H66O4
778	683.5	332.3	(+)	35	EPAHEDA	Eicosapentaenoic acid-hydroxy eicosadienoic acid	FAHFA(20:5-O-20:2)	C40H64O4
779	721.5	332.3	(+)	35	BAHEDA	Behenic acid-hydroxy eicosadienoic acid	FAHFA(22:0-O-20:2)	C42H78O4
780	719.5	332.3	(+)	35	DEAHEDA	Docosenoic acid-hydroxy eicosadienoic acid	FAHFA(22:1-O-20:2)	C42H76O4
781	717.5	332.3	(+)	35	DDAHEDA	Docosadienoate-hydroxy eicosadienoic acid	FAHFA(22:2-O-20:2)	C40H74O4
782	709.5	332.3	(+)	35	DHAHEDA	Docosahexaenoic acid-hydroxy eicosadienoic acid	FAHFA(22:6-O-20:2)	C42H66O4
783	607.5	330.3	(+)	35	MAHETA	Myristic acid-hydroxy eicosatrienoic acid	FAHFA(14:0-O-20:3)	C34H60O4
784	605.5	330.3	(+)	35	MOHETA	Myristoleic acid-hydroxy eicosatrienoic acid	FAHFA(14:1-O-20:3)	C34H58O4
785	621.5	330.3	(+)	35	PDAHETA	Pentadecanoic acid-hydroxy eicosatrienoic acid	FAHFA(15:0-O-20:3)	C35H62O4
786	619.5	330.3	(+)	35	PDEAHETA	Pentadecenoic acid-hydroxy eicosatrienoic acid	FAHFA(15:1-O-20:3)	C35H60O4
787	633.5	330.3	(+)	35	POHETA	Palmitoleic acid-hydroxy eicosatrienoic acid	FAHFA(16:1-O-20:3)	C36H62O4
788	635.5	330.3	(+)	35	PAHETA	Palmitic acid-hydroxy eicosatrienoic acid	FAHFA(16:0-O-20:3)	C36H64O4
789	649.5	330.3	(+)	35	HDAHETA	Heptadecanoic acid-hydroxy eicosatrienoic acid	FAHFA(17:0-O-20:3)	C37H66O4
790	647.5	330.3	(+)	35	HDEAHETA	Heptadecenoic acid-hydroxy eicosatrienoic acid	FAHFA(17:1-O-20:3)	C37H64O4
791	663.5	330.3	(+)	35	SAHETA	Stearic acid-hydroxy eicosatrienoic acid	FAHFA(18:0-O-20:3)	C38H68O4
792	661.5	330.3	(+)	35	OAHETA	Oleic acid-hydroxy eicosatrienoic acid	FAHFA(18:1-O-20:3)	C38H66O4
793	659.5	330.3	(+)	35	LAHETA	Linoleic acid-hydroxy eicosatrienoic acid	FAHFA(18:2-O-20:3)	C38H64O4
794	657.5	330.3	(+)	35	ALAHETA	α-Linolenic acid-hydroxy eicosatrienoic acid	FAHFA(18:3-O-20:3)	C38H62O4
795	655.5	330.3	(+)	35	SDAHETA	Stearidonic acid-hydroxy eicosatrienoic acid	FAHFA(18:4-O-20:3)	C38H60O4
796	691.5	330.3	(+)	35	AAHETA	Arachidic acid-hydroxy eicosatrienoic acid	FAHFA(20:0-O-20:3)	C40H72O4
797	689.5	330.3	(+)	35	EAHETA	Eicosenoic acid-hydroxy eicosatrienoic acid	FAHFA(20:1-O-20:3)	C40H70O4
798	687.5	330.3	(+)	35	EDAHETA	Eicosadienoic acid-hydroxy eicosatrienoic acid	FAHFA(20:2-O-20:3)	C40H68O4
799	685.5	330.3	(+)	35	ETAHETA	Eicosatrienoic acid-hydroxy eicosatrienoic acid	FAHFA(20:3-O-20:3)	C40H66O4
800	683.5	330.3	(+)	35	ARAHETA	Arachidonic acid-hydroxy eicosatrienoic acid	FAHFA(20:4-O-20:3)	C40H64O4
801	681.5	330.3	(+)	35	EPAHETA	Eicosapentaenoic acid-hydroxy eicosatrienoic acid	FAHFA(20:5-O-20:3)	C40H62O4
802	719.5	330.3	(+)	35	BAHETA	Behenic acid-hydroxy eicosatrienoic acid	FAHFA(22:0-O-20:3)	C42H76O4
803	717.5	330.3	(+)	35	DEAHETA	Docosenoic acid-hydroxy eicosatrienoic acid	FAHFA(22:1-O-20:3)	C42H74O4
804	715.5	330.3	(+)	35	DDAHETA	Docosadienoate-hydroxy eicosatrienoic acid	FAHFA(22:2-O-20:3)	C40H72O4
805	707.5	330.3	(+)	35	DHAHETA	Docosahexaenoic acid-hydroxy eicosatrienoic acid	FAHFA(22:6-O-20:3)	C42H64O4
806	629.5	352.3	(+)	35	MAHDHA	Myristic acid-hydroxy docosahexaenoic acid	FAHFA(14:0-O-22:6)	C36H58O4
807	627.5	352.3	(+)	35	MOHDHA	Myristoleic acid-hydroxy docosahexaenoic acid	FAHFA(14:1-O-22:6)	C36H56O4
808	643.5	352.3	(+)	35	PDAHDHA	Pentadecanoic acid-hydroxy docosahexaenoic acid	FAHFA(15:0-O-22:6)	C37H60O4
809	641.5	352.3	(+)	35	PDEAHDHA	Pentadecenoic acid-hydroxy docosahexaenoic acid	FAHFA(15:1-O-22:6)	C37H58O4
810	655.5	352.3	(+)	35	POHDHA	Palmitoleic acid-hydroxy docosahexaenoic acid	FAHFA(16:1-O-22:6)	C38H60O4
811	657.5	352.3	(+)	35	PAHDHA	Palmitic acid-hydroxy docosahexaenoic acid	FAHFA(16:0-O-22:6)	C38H62O4
812	671.5	352.3	(+)	35	HDAHDHA	Heptadecanoic acid-hydroxy docosahexaenoic acid	FAHFA(17:0-O-22:6)	C39H64O4
813	669.5	352.3	(+)	35	HDEAHDHA	Heptadecenoic acid-hydroxy docosahexaenoic acid	FAHFA(17:1-O-22:6)	C39H62O4
814	685.5	352.3	(+)	35	SAHDHA	Stearic acid-hydroxy docosahexaenoic acid	FAHFA(18:0-O-22:6)	C40H66O4
815	683.5	352.3	(+)	35	OAHDHA	Oleic acid-hydroxy docosahexaenoic acid	FAHFA(18:1-O-22:6)	C40H64O4
816	681.5	352.3	(+)	35	LAHDHA	Linoleic acid-hydroxy docosahexaenoic acid	FAHFA(18:2-O-22:6)	C40H62O4
817	679.5	352.3	(+)	35	ALAHDHA	α-Linolenic acid-hydroxy docosahexaenoic acid	FAHFA(18:3-O-22:6)	C40H60O4
818	677.5	352.3	(+)	35	SDAHDHA	Stearidonic acid-hydroxy docosahexaenoic acid	FAHFA(18:4-O-22:6)	C40H58O4
819	713.5	352.3	(+)	35	AAHDHA	Arachidic acid-hydroxy docosahexaenoic acid	FAHFA(20:0-O-22:6)	C42H70O4
820	711.5	352.3	(+)	35	EAHDHA	Eicosenoic acid-hydroxy docosahexaenoic acid	FAHFA(20:1-O-22:6)	C42H68O4
821	709.5	352.3	(+)	35	EDAHDHA	Eicosadienoic acid-hydroxy docosahexaenoic acid	FAHFA(20:2-O-22:6)	C42H66O4
822	707.5	352.3	(+)	35	ETAHDHA	Eicosatrienoic acid-hydroxy docosahexaenoic acid	FAHFA(20:3-O-22:6)	C42H64O4
823	705.5	352.3	(+)	35	ARAHDHA	Arachidonic acid-hydroxy docosahexaenoic acid	FAHFA(20:4-O-22:6)	C42H62O4
824	703.5	352.3	(+)	35	EPAHDHA	Eicosapentaenoic acid-hydroxy docosahexaenoic acid	FAHFA(20:5-O-22:6)	C42H60O4
825	741.5	352.3	(+)	35	BAHDHA	Behenic acid-hydroxy docosahexaenoic acid	FAHFA(22:0-O-22:6)	C44H74O4
826	739.5	352.3	(+)	35	DEAHDHA	Docosenoic acid-hydroxy docosahexaenoic acid	FAHFA(22:1-O-22:6)	C44H72O4
827	737.5	352.3	(+)	35	DDAHDHA	Docosadienoate-hydroxy docosahexaenoic acid	FAHFA(22:2-O-22:6)	C44H70O4
828	729.5	352.3	(+)	35	DHAHDHA	Docosahexaenoic acid-hydroxy docosahexaenoic acid	FAHFA(22:6-O-22:6)	C44H62O4
829	605.5	356.3	(+)	35	MAHARA	Myristic acid-hydroxy arachidonic acid	FAHFA(14:0-O-20:4)	C34H58O4
830	603.5	356.3	(+)	35	MOHARA	Myristoleic acid-hydroxy arachidonic acid	FAHFA(14:1-O-20:4)	C34H56O4
831	619.5	356.3	(+)	35	PDAHARA	Pentadecanoic acid-hydroxy arachidonic acid	FAHFA(15:0-O-20:4)	C35H60O4
832	617.5	356.3	(+)	35	PDEAHARA	Pentadecenoic acid-hydroxy arachidonic acid	FAHFA(15:1-O-20:4)	C35H58O4
833	631.5	356.3	(+)	35	POHARA	Palmitoleic acid-hydroxy arachidonic acid	FAHFA(16:1-O-20:4)	C36H60O4
834	633.5	356.3	(+)	35	PAHARA	Palmitic acid-hydroxy arachidonic acid	FAHFA(16:0-O-20:4)	C36H62O4

835	647.5	356.3	(+)	35	HDAHARA	Heptadecanoic acid-hydroxy arachidonic acid	FAHFA(17:0-O-20:4)	C37H64O4
836	645.5	356.3	(+)	35	HDEAHARA	Heptadecenoic acid-hydroxy arachidonic acid	FAHFA(17:1-O-20:4)	C37H62O4
837	661.5	356.3	(+)	35	SAHARA	Stearic acid-hydroxy arachidonic acid	FAHFA(18:0-O-20:4)	C38H66O4
838	659.5	356.3	(+)	35	OAHARA	Oleic acid-hydroxy arachidonic acid	FAHFA(18:1-O-20:4)	C38H64O4
839	657.5	356.3	(+)	35	LAHARA	Linoleic acid-hydroxy arachidonic acid	FAHFA(18:2-O-20:4)	C38H62O4
840	655.5	356.3	(+)	35	ALAHARA	α-Linolenic acid-hydroxy arachidonic acid	FAHFA(18:3-O-20:4)	C38H60O4
841	653.5	356.3	(+)	35	SDAHARA	Stearidonic acid-hydroxy arachidonic acid	FAHFA(18:4-O-20:4)	C38H58O4
842	689.5	356.3	(+)	35	AAHARA	Arachidic acid-hydroxy arachidonic acid	FAHFA(20:0-O-20:4)	C40H70O4
843	687.5	356.3	(+)	35	EAHARA	Eicosenoic acid-hydroxy arachidonic acid	FAHFA(20:1-O-20:4)	C40H68O4
844	685.5	356.3	(+)	35	EDAHARA	Eicosadienoic acid-hydroxy arachidonic acid	FAHFA(20:2-O-20:4)	C40H66O4
845	683.5	356.3	(+)	35	ETAHARA	Eicosatrienoic acid-hydroxy arachidonic acid	FAHFA(20:3-O-20:4)	C40H64O4
846	681.5	356.3	(+)	35	ARAHARA	Arachidonic acid-hydroxy arachidonic acid	FAHFA(20:4-O-20:4)	C40H62O4
847	679.5	356.3	(+)	35	EPAHARA	Eicosapentaenoic acid-hydroxy arachidonic acid	FAHFA(20:5-O-20:4)	C40H60O4
848	717.5	356.3	(+)	35	BAHARA	Behenic acid-hydroxy arachidonic acid	FAHFA(22:0-O-20:4)	C42H74O4
849	715.5	356.3	(+)	35	DEAHARA	Docosenoic acid-hydroxy arachidonic acid	FAHFA(22:1-O-20:4)	C42H72O4
850	713.5	356.3	(+)	35	DDAHARA	Docosadienoate-hydroxy arachidonic acid	FAHFA(22:2-O-20:4)	C40H70O4
851	705.5	356.3	(+)	35	DHAHARA	Docosahexaenoic acid-hydroxy arachidonic acid	FAHFA(22:6-O-20:4)	C42H62O4
852	501.5	224.2	(+)	35	MAHDA	Myristic acid-hydroxy dodecanoic acid	FAHFA(14:0-O-12:0)	C26H50O4
853	499.5	224.2	(+)	35	MOHDA	Myristoleic acid-hydroxy dodecanoic acid	FAHFA(14:1-O-12:0)	C22H48O4
854	515.5	224.2	(+)	35	PDAHDA	Pentadecanoic acid-hydroxy dodecanoic acid	FAHFA(15:0-O-12:0)	C27H52O4
855	513.5	224.2	(+)	35	PDEAHDA	Pentadecenoic acid-hydroxy dodecanoic acid	FAHFA(15:1-O-12:0)	C27H50O4
856	527.5	224.2	(+)	35	POHDA	Palmitoleic acid-hydroxy dodecanoic acid	FAHFA(16:1-O-12:0)	C28H52O4
857	529.5	224.2	(+)	35	PAHDA	Palmitic acid-hydroxy dodecanoic acid	FAHFA(16:0-O-12:0)	C28H54O4
858	543.5	224.2	(+)	35	HDAHDA	Heptadecanoic acid-hydroxy dodecanoic acid	FAHFA(17:0-O-12:0)	C29H56O4
859	541.5	224.2	(+)	35	HDEAHDA	Heptadecenoic acid-hydroxy dodecanoic acid	FAHFA(17:1-O-12:0)	C29H54O4
860	557.5	224.2	(+)	35	SAHDA	Stearic acid-hydroxy dodecanoic acid	FAHFA(18:0-O-12:0)	C30H58O4
861	555.5	224.2	(+)	35	OAHDA	Oleic acid-hydroxy dodecanoic acid	FAHFA(18:1-O-12:0)	C30H56O4
862	553.5	224.2	(+)	35	LAHDA	Linoleic acid-hydroxy dodecanoic acid	FAHFA(18:2-O-12:0)	C30H54O4
863	551.5	224.2	(+)	35	ALAHDA	α-Linolenic acid-hydroxy dodecanoic acid	FAHFA(18:3-O-12:0)	C30H52O4
864	549.5	224.2	(+)	35	SDAHDA	Stearidonic acid-hydroxy dodecanoic acid	FAHFA(18:4-O-12:0)	C30H50O4
865	585.5	224.2	(+)	35	AAHDA	Arachidic acid-hydroxy dodecanoic acid	FAHFA(20:0-O-12:0)	C32H62O4
866	583.5	224.2	(+)	35	EAHDA	Eicosenoic acid-hydroxy dodecanoic acid	FAHFA(20:1-O-12:0)	C32H60O4
867	581.5	224.2	(+)	35	EDAHDA	Eicosadienoic acid-hydroxy dodecanoic acid	FAHFA(20:2-O-12:0)	C32H58O4
868	579.5	224.2	(+)	35	ETAHDA	Eicosatrienoic acid-hydroxy dodecanoic acid	FAHFA(20:3-O-12:0)	C32H56O4
869	577.5	224.2	(+)	35	ARAHDA	Arachidonic acid-hydroxy dodecanoic acid	FAHFA(20:4-O-12:0)	C32H54O4
870	575.5	224.2	(+)	35	EPAHDA	Eicosapentaenoic acid-hydroxy dodecanoic acid	FAHFA(20:5-O-12:0)	C32H52O4
871	613.5	224.2	(+)	35	BAHDA	Behenic acid-hydroxy dodecanoic acid	FAHFA(22:0-O-12:0)	C34H66O4
872	611.5	224.2	(+)	35	DEAHDA	Docosenoic acid-hydroxy dodecanoic acid	FAHFA(22:1-O-12:0)	C34H64O4
873	609.5	224.2	(+)	35	DDAHDA	Docosadienoate-hydroxy dodecanoic acid	FAHFA(22:2-O-12:0)	C34H62O4
874	601.5	224.2	(+)	35	DHAHDA	Docosahexaenoic acid-hydroxy dodecanoic acid	FAHFA(22:6-O-12:0)	C34H54O4
875	529.5	252.2	(+)	35	MAHMA	Myristic acid-hydroxy myristic acid	FAHFA(14:0-O-14:0)	C28H54O4
876	527.5	252.2	(+)	35	MOHMA	Myristoleic acid-hydroxy myristic acid	FAHFA(14:1-O-14:0)	C28H52O4
877	543.5	252.2	(+)	35	PDAHMA	Pentadecanoic acid-hydroxy myristic acid	FAHFA(15:0-O-14:0)	C29H56O4
878	541.5	252.2	(+)	35	PDEAHMA	Pentadecenoic acid-hydroxy myristic acid	FAHFA(15:1-O-14:0)	C29H54O4
879	555.5	252.2	(+)	35	POHMA	Palmitoleic acid-hydroxy myristic acid	FAHFA(16:1-O-14:0)	C30H56O4
880	557.5	252.2	(+)	35	PAHMA	Palmitic acid-hydroxy myristic acid	FAHFA(16:0-O-14:0)	C30H58O4
881	571.5	252.2	(+)	35	HDAHMA	Heptadecanoic acid-hydroxy myristic acid	FAHFA(17:0-O-14:0)	C31H60O4
882	569.5	252.2	(+)	35	HDEAHMA	Heptadecenoic acid-hydroxy myristic acid	FAHFA(17:1-O-14:0)	C31H58O4
883	585.5	252.2	(+)	35	SAHMA	Stearic acid-hydroxy myristic acid	FAHFA(18:0-O-14:0)	C32H62O4
884	583.5	252.2	(+)	35	OAHMA	Oleic acid-hydroxy myristic acid	FAHFA(18:1-O-14:0)	C32H60O4
885	581.5	252.2	(+)	35	LAHMA	Linoleic acid-hydroxy myristic acid	FAHFA(18:2-O-14:0)	C32H58O4
886	579.5	252.2	(+)	35	ALAHMA	α-Linolenic acid-hydroxy myristic acid	FAHFA(18:3-O-14:0)	C32H56O4
887	577.5	252.2	(+)	35	SDAHMA	Stearidonic acid-hydroxy myristic acid	FAHFA(18:4-O-14:0)	C32H54O4
888	613.5	252.2	(+)	35	AAHMA	Arachidic acid-hydroxy myristic acid	FAHFA(20:0-O-14:0)	C34H66O4
889	611.5	252.2	(+)	35	EAHMA	Eicosenoic acid-hydroxy myristic acid	FAHFA(20:1-O-14:0)	C34H64O4
890	609.5	252.2	(+)	35	EDAHMA	Eicosadienoic acid-hydroxy myristic acid	FAHFA(20:2-O-14:0)	C34H62O4
891	607.5	252.2	(+)	35	ETAHMA	Eicosatrienoic acid-hydroxy myristic acid	FAHFA(20:3-O-14:0)	C34H60O4
892	605.5	252.2	(+)	35	ARAHMA	Arachidonic acid-hydroxy myristic acid	FAHFA(20:4-O-14:0)	C34H58O4
893	603.5	252.2	(+)	35	EPAHMA	Eicosapentaenoic acid-hydroxy myristic acid	FAHFA(20:5-O-14:0)	C34H56O4
894	641.5	252.2	(+)	35	BAHMA	Behenic acid-hydroxy myristic acid	FAHFA(22:0-O-14:0)	C36H70O4
895	639.5	252.2	(+)	35	DEAHMA	Docosenoic acid-hydroxy myristic acid	FAHFA(22:1-O-14:0)	C36H68O4
896	637.5	252.2	(+)	35	DDAHMA	Docosadienoate-hydroxy myristic acid	FAHFA(22:2-O-14:0)	C36H66O4
897	629.5	252.2	(+)	35	DHAHMA	Docosahexaenoic acid-hydroxy myristic acid	FAHFA(22:6-O-14:0)	C36H58O4
898	543.5	266.2	(+)	35	MAHPDA	Myristic acid-hydroxy pentadecanoic acid	FAHFA(14:0-O-15:0)	C29H56O4
899	541.5	266.2	(+)	35	MOHPDA	Myristoleic acid-hydroxy pentadecanoic acid	FAHFA(14:1-O-15:0)	C29H54O4
900	557.5	266.2	(+)	35	PDAHPDA	Pentadecanoic acid-hydroxy pentadecanoic acid	FAHFA(15:0-O-15:0)	C30H58O4
901	555.5	266.2	(+)	35	PDEAHPDA	Pentadecenoic acid-hydroxy pentadecanoic acid	FAHFA(15:1-O-15:0)	C30H56O4
902	569.5	266.2	(+)	35	POHPDA	Palmitoleic acid-hydroxy pentadecanoic acid	FAHFA(16:1-O-15:0)	C31H58O4
903	571.5	266.2	(+)	35	PAHPDA	Palmitic acid-hydroxy pentadecanoic acid	FAHFA(16:0-O-15:0)	C31H60O4
904	585.5	266.2	(+)	35	HDAHPDA	Heptadecanoic acid-hydroxy pentadecanoic acid	FAHFA(17:0-O-15:0)	C32H62O4
905	583.5	266.2	(+)	35	HDEAHPDA	Heptadecenoic acid-hydroxy pentadecanoic acid	FAHFA(17:1-O-15:0)	C32H60O4
906	599.5	266.2	(+)	35	SAHPDA	Stearic acid-hydroxy pentadecanoic acid	FAHFA(18:0-O-15:0)	C33H64O4
907	597.5	266.2	(+)	35	OAHPDA	Oleic acid-hydroxy pentadecanoic acid	FAHFA(18:1-O-15:0)	C33H62O4
908	595.5	266.2	(+)	35	LAHPDA	Linoleic acid-hydroxy pentadecanoic acid	FAHFA(18:2-O-15:0)	C33H60O4
909	593.5	266.2	(+)	35	ALAHPDA	α-Linolenic acid-hydroxy pentadecanoic acid	FAHFA(18:3-O-15:0)	C33H58O4
910	591.5	266.2	(+)	35	SDAHPDA	Stearidonic acid-hydroxy pentadecanoic acid	FAHFA(18:4-O-15:0)	C33H56O4
911	627.5	266.2	(+)	35	AAHPDA	Arachidic acid-hydroxy pentadecanoic acid	FAHFA(20:0-O-15:0)	C35H68O4
912	625.5	266.2	(+)	35	EAHPDA	Eicosenoic acid-hydroxy pentadecanoic acid	FAHFA(20:1-O-15:0)	C35H66O4
913	623.5	266.2	(+)	35	EDAHPDA	Eicosadienoic acid-hydroxy pentadecanoic acid	FAHFA(20:2-O-15:0)	C35H64O4
914	621.5	266.2	(+)	35	ETAHPDA	Eicosatrienoic acid-hydroxy pentadecanoic acid	FAHFA(20:3-O-15:0)	C35H62O4
915	619.5	266.2	(+)	35	ARAHPDA	Arachidonic acid-hydroxy pentadecanoic acid	FAHFA(20:4-O-15:0)	C35H60O4
916	617.5	266.2	(+)	35	EPAHPDA	Eicosapentaenoic acid-hydroxy pentadecanoic acid	FAHFA(20:5-O-15:0)	C35H58O4
917	655.5	266.2	(+)	35	BAHPDA	Behenic acid-hydroxy pentadecanoic acid	FAHFA(22:0-O-15:0)	C37H72O4
918	653.5	266.2	(+)	35	DEAHPDA	Docosenoic acid-hydroxy pentadecanoic acid	FAHFA(22:1-O-15:0)	C37H70O4
919	651.5	266.2	(+)	35	DDAHPDA	Docosadienoate-hydroxy pentadecanoic acid	FAHFA(22:2-O-15:0)	C37H68O4
920	643.5	266.2	(+)	35	DHAHPDA	Docosahexaenoic acid-hydroxy pentadecanoic acid	FAHFA(22:6-O-15:0)	C37H60O4
921	557.6	280.3	(+)	35	MAHPA	Myristic acid-hydroxy palmitic acid	FAHFA(14:0-O-16:0)	C30H58O4
922	555.6	280.3	(+)	35	MOHPA	Myristoleic acid-hydroxy palmitic acid	FAHFA(14:1-O-16:0)	C30H56O4
923	571.6	280.3	(+)	35	PDAHPA	Pentadecanoic acid-hydroxy palmitic acid	FAHFA(15:0-O-16:0)	C31H60O4
924	569.6	280.3	(+)	35	PDEAHPA	Pentadecenoic acid-hydroxy palmitic acid	FAHFA(15:1-O-16:0)	C31H58O4
925	583.6	280.3	(+)	35	POHPA	Palmitoleic acid-hydroxy palmitic acid	FAHFA(16:1-O-16:0)	C32H60O4
926	585.6	280.3	(+)	35	PAHPA	Palmitic acid-hydroxy palmitic acid	FAHFA(16:0-O-16:0)	C32H62O4
927	599.6	280.3	(+)	35	HDAHPA	Heptadecanoic acid-hydroxy palmitic acid	FAHFA(17:0-O-16:0)	C33H64O4
928	597.6	280.3	(+)	35	HDEAHPA	Heptadecenoic acid-hydroxy palmitic acid	FAHFA(17:1-O-16:0)	C33H62O4
929	613.6	280.3	(+)	35	SAHPA	Stearic acid-hydroxy palmitic acid	FAHFA(18:0-O-16:0)	C34H66O4
930	611.6	280.3	(+)	35	OAHPA	Oleic acid-hydroxy palmitic acid	FAHFA(18:1-O-16:0)	C34H64O4
931	609.6	280.3	(+)	35	LAHPA	Linoleic acid-hydroxy palmitic acid	FAHFA(18:2-O-16:0)	C34H62O4
932	607.6	280.3	(+)	35	ALAHPA	α-Linolenic acid-hydroxy palmitic acid	FAHFA(18:3-O-16:0)	C34H60O4
933	605.6	280.3	(+)	35	SDAHPA	Stearidonic acid-hydroxy palmitic acid	FAHFA(18:4-O-16:0)	C34H58O4
934	641.6	280.3	(+)	35	AAHPA	Arachidic acid-hydroxy palmitic acid	FAHFA(20:0-O-16:0)	C36H70O4
935	639.6	280.3	(+)	35	EAHPA	Eicosenoic acid-hydroxy palmitic acid	FAHFA(20:1-O-16:0)	C36H68O4
936	637.6	280.3	(+)	35	EDAHPA	Eicosadienoic acid-hydroxy palmitic acid	FAHFA(20:2-O-16:0)	C36H66O4
937	635.6	280.3	(+)	35	ETAHPA	Eicosatrienoic acid-hydroxy palmitic acid	FAHFA(20:3-O-16:0)	C36H64O4
938	633.6	280.3	(+)	35	ARAHPA	Arachidonic acid-hydroxy palmitic acid	FAHFA(20:4-O-16:0)	C36H62O4
939	631.6	280.3	(+)	35	EPAHPA	Eicosapentaenoic acid-hydroxy palmitic acid	FAHFA(20:5-O-16:0)	C36H60O4

940	669.6	280.3	(+)	35	BAHPA	Behenic acid-hydroxy palmitic acid	FAHFA(22:0-O-16:0)	C38H74O4
941	667.6	280.3	(+)	35	DEAHPA	Docosenoic acid-hydroxy palmitic acid	FAHFA(22:1-O-16:0)	C38H72O4
942	665.6	280.3	(+)	35	DDAHPA	Docosadienoate-hydroxy palmitic acid	FAHFA(22:2-O-16:0)	C38H70O4
943	657.6	280.3	(+)	35	DHAHPA	Docosahexaenoic acid-hydroxy palmitic acid	FAHFA(22:6-O-16:0)	C38H62O4
944	571.6	294.3	(+)	35	MAHHDA	Myristic acid-hydroxy heptadecanoic acid	FAHFA(14:0-O-17:0)	C31H60O4
945	569.6	294.3	(+)	35	MOHHDA	Myristoleic acid-hydroxy heptadecanoic acid	FAHFA(14:1-O-17:0)	C31H58O4
946	585.6	294.3	(+)	35	PDAHHDA	Pentadecanoic acid-hydroxy heptadecanoic acid	FAHFA(15:0-O-17:0)	C32H62O4
947	583.6	294.3	(+)	35	PDEAHHDA	Pentadecenoic acid-hydroxy heptadecanoic acid	FAHFA(15:1-O-17:0)	C32H60O4
948	597.6	294.3	(+)	35	POHHDA	Palmitoleic acid-hydroxy heptadecanoic acid	FAHFA(16:1-O-17:0)	C33H62O4
949	599.6	294.3	(+)	35	PAHHDA	Palmitic acid-hydroxy heptadecanoic acid	FAHFA(16:0-O-17:0)	C33H64O4
950	613.6	294.3	(+)	35	HDAHHDA	Heptadecanoic acid-hydroxy heptadecanoic acid	FAHFA(17:0-O-17:0)	C34H66O4
951	611.6	294.3	(+)	35	HDEAHHDA	Heptadecenoic acid-hydroxy heptadecanoic acid	FAHFA(17:1-O-17:0)	C34H64O4
952	627.6	294.3	(+)	35	SAHHDA	Stearic acid-hydroxy heptadecanoic acid	FAHFA(18:0-O-17:0)	C35H68O4
953	625.6	294.3	(+)	35	OAHHDA	Oleic acid-hydroxy heptadecanoic acid	FAHFA(18:1-O-17:0)	C35H66O4
954	623.6	294.3	(+)	35	LAHHDA	Linoleic acid-hydroxy heptadecanoic acid	FAHFA(18:2-O-17:0)	C35H64O4
955	621.6	294.3	(+)	35	ALAHHDA	α-Linolenic acid-hydroxy heptadecanoic acid	FAHFA(18:3-O-17:0)	C35H62O4
956	619.6	294.3	(+)	35	SDAHHDA	Stearidonic acid-hydroxy heptadecanoic acid	FAHFA(18:4-O-17:0)	C35H60O4
957	655.6	294.3	(+)	35	AAHHDA	Arachidic acid-hydroxy heptadecanoic acid	FAHFA(20:0-O-17:0)	C37H72O4
958	653.6	294.3	(+)	35	EAHHDA	Eicosenoic acid-hydroxy heptadecanoic acid	FAHFA(20:1-O-17:0)	C37H70O4
959	651.6	294.3	(+)	35	EDAHHDA	Eicosadienoic acid-hydroxy heptadecanoic acid	FAHFA(20:2-O-17:0)	C37H68O4
960	649.6	294.3	(+)	35	ETAHHDA	Eicosatrienoic acid-hydroxy heptadecanoic acid	FAHFA(20:3-O-17:0)	C37H66O4
961	647.6	294.3	(+)	35	ARAHHDA	Arachidonic acid-hydroxy heptadecanoic acid	FAHFA(20:4-O-17:0)	C37H64O4
962	645.6	294.3	(+)	35	EPAHHDA	Eicosapentaenoic acid-hydroxy heptadecanoic acid	FAHFA(20:5-O-17:0)	C37H62O4
963	683.6	294.3	(+)	35	BAHHDA	Behenic acid-hydroxy heptadecanoic acid	FAHFA(22:0-O-17:0)	C39H76O4
964	681.6	294.3	(+)	35	DEAHHDA	Docosenoic acid-hydroxy heptadecanoic acid	FAHFA(22:1-O-17:0)	C39H74O4
965	679.6	294.3	(+)	35	DDAHHDA	Docosadienoate-hydroxy heptadecanoic acid	FAHFA(22:2-O-17:0)	C39H72O4
966	671.6	294.3	(+)	35	DHAHHDA	Docosahexaenoic acid-hydroxy heptadecanoic acid	FAHFA(22:6-O-17:0)	C39H64O4
967	585.8	308.3	(+)	35	MAHSA	Myristic acid-hydroxy stearic acid	FAHFA(14:0-O-18:0)	C32H62O4
968	583.8	308.3	(+)	35	MOHSA	Myristoleic acid-hydroxy stearic acid	FAHFA(14:1-O-18:0)	C32H60O4
969	599.8	308.3	(+)	35	PDAHSA	Pentadecanoic acid-hydroxy stearic acid	FAHFA(15:0-O-18:0)	C33H64O4
970	597.8	308.3	(+)	35	PDEAHSA	Pentadecenoic acid-hydroxy stearic acid	FAHFA(15:1-O-18:0)	C33H62O4
971	611.8	308.3	(+)	35	POHSA	Palmitoleic acid-hydroxy stearic acid	FAHFA(16:1-O-18:0)	C34H64O4
972	613.8	308.3	(+)	35	PAHSA	Palmitic acid-hydroxy stearic acid	FAHFA(16:0-O-18:0)	C34H66O4
973	627.8	308.3	(+)	35	HDAHSA	Heptadecanoic acid-hydroxy stearic acid	FAHFA(17:0-O-18:0)	C35H68O4
974	625.8	308.3	(+)	35	HDEAHSA	Heptadecenoic acid-hydroxy stearic acid	FAHFA(17:1-O-18:0)	C35H66O4
975	641.8	308.3	(+)	35	SAHSA	Stearic acid-hydroxy stearic acid	FAHFA(18:0-O-18:0)	C36H70O4
976	639.8	308.3	(+)	35	OAHSA	Oleic acid-hydroxy stearic acid	FAHFA(18:1-O-18:0)	C36H68O4
977	637.8	308.3	(+)	35	LAHSA	Linoleic acid-hydroxy stearic acid	FAHFA(18:2-O-18:0)	C36H66O4
978	635.8	308.3	(+)	35	ALAHSA	α-Linolenic acid-hydroxy stearic acid	FAHFA(18:3-O-18:0)	C36H64O4
979	633.8	308.3	(+)	35	SDAHSA	Stearidonic acid-hydroxy stearic acid	FAHFA(18:4-O-18:0)	C36H62O4
980	669.8	308.3	(+)	35	AAHSA	Arachidic acid-hydroxy stearic acid	FAHFA(20:0-O-18:0)	C38H74O4
981	667.8	308.3	(+)	35	EAHSA	Eicosenoic acid-hydroxy stearic acid	FAHFA(20:1-O-18:0)	C38H72O4
982	665.8	308.3	(+)	35	EDAHSA	Eicosadienoic acid-hydroxy stearic acid	FAHFA(20:2-O-18:0)	C38H70O4
983	663.8	308.3	(+)	35	ETAHSA	Eicosatrienoic acid-hydroxy stearic acid	FAHFA(20:3-O-18:0)	C38H68O4
984	661.8	308.3	(+)	35	ARAHSA	Arachidonic acid-hydroxy stearic acid	FAHFA(20:4-O-18:0)	C38H66O4
985	659.8	308.3	(+)	35	EPAHSA	Eicosapentaenoic acid-hydroxy stearic acid	FAHFA(20:5-O-18:0)	C38H64O4
986	697.8	308.3	(+)	35	BAHSA	Behenic acid-hydroxy stearic acid	FAHFA(22:0-O-18:0)	C40H78O4
987	695.8	308.3	(+)	35	DEAHSA	Docosenoic acid-hydroxy stearic acid	FAHFA(22:1-O-18:0)	C40H76O4
988	693.8	308.3	(+)	35	DDAHSA	Docosadienoate-hydroxy stearic acid	FAHFA(22:2-O-18:0)	C40H74O4
989	685.8	308.3	(+)	35	DHAHSA	Docosahexaenoic acid-hydroxy stearic acid	FAHFA(22:6-O-18:0)	C40H66O4
990	599.8	322.3	(+)	35	MAHNDA	Myristic acid-hydroxy nonadecanoic acid	FAHFA(14:0-O-19:0)	C33H64O4
991	597.8	322.3	(+)	35	MOHNDA	Myristoleic acid-hydroxy nonadecanoic acid	FAHFA(14:1-O-19:0)	C33H62O4
992	613.8	322.3	(+)	35	PDAHNDA	Pentadecanoic acid-hydroxy nonadecanoic acid	FAHFA(15:0-O-19:0)	C34H66O4
993	611.8	322.3	(+)	35	PDEAHNDA	Pentadecenoic acid-hydroxy nonadecanoic acid	FAHFA(15:1-O-19:0)	C34H64O4
994	625.8	322.3	(+)	35	POHNDA	Palmitoleic acid-hydroxy nonadecanoic acid	FAHFA(16:1-O-19:0)	C35H66O4
995	627.8	322.3	(+)	35	PAHNDA	Palmitic acid-hydroxy nonadecanoic acid	FAHFA(16:0-O-19:0)	C35H68O4
996	641.8	322.3	(+)	35	HDAHNDA	Heptadecanoic acid-hydroxy nonadecanoic acid	FAHFA(17:0-O-19:0)	C36H70O4
997	639.8	322.3	(+)	35	HDEAHNDA	Heptadecenoic acid-hydroxy nonadecanoic acid	FAHFA(17:1-O-19:0)	C36H68O4
998	655.8	322.3	(+)	35	SAHNDA	Stearic acid-hydroxy nonadecanoic acid	FAHFA(18:0-O-19:0)	C37H72O4
999	653.8	322.3	(+)	35	OAHNDA	Oleic acid-hydroxy nonadecanoic acid	FAHFA(18:1-O-19:0)	C37H70O4
1000	651.8	322.3	(+)	35	LAHNDA	Linoleic acid-hydroxy nonadecanoic acid	FAHFA(18:2-O-19:0)	C37H68O4
1001	649.8	322.3	(+)	35	ALAHNDA	α-Linolenic acid-hydroxy nonadecanoic acid	FAHFA(18:3-O-19:0)	C37H66O4
1002	647.8	322.3	(+)	35	SDAHNDA	Stearidonic acid-hydroxy nonadecanoic acid	FAHFA(18:4-O-19:0)	C37H64O4
1003	683.8	322.3	(+)	35	AAHNDA	Arachidic acid-hydroxy nonadecanoic acid	FAHFA(20:0-O-19:0)	C39H76O4
1004	681.8	322.3	(+)	35	EAHNDA	Eicosenoic acid-hydroxy nonadecanoic acid	FAHFA(20:1-O-19:0)	C39H74O4
1005	679.8	322.3	(+)	35	EDAHNDA	Eicosadienoic acid-hydroxy nonadecanoic acid	FAHFA(20:2-O-19:0)	C39H72O4
1006	677.8	322.3	(+)	35	ETAHNDA	Eicosatrienoic acid-hydroxy nonadecanoic acid	FAHFA(20:3-O-19:0)	C39H70O4
1007	675.8	322.3	(+)	35	ARAHNDA	Arachidonic acid-hydroxy nonadecanoic acid	FAHFA(20:4-O-19:0)	C39H68O4
1008	673.8	322.3	(+)	35	EPAHNDA	Eicosapentaenoic acid-hydroxy nonadecanoic acid	FAHFA(20:5-O-19:0)	C39H66O4
1009	711.8	322.3	(+)	35	BAHNDA	Behenic acid-hydroxy nonadecanoic acid	FAHFA(22:0-O-19:0)	C41H80O4
1010	709.8	322.3	(+)	35	DEAHNDA	Docosenoic acid-hydroxy nonadecanoic acid	FAHFA(22:1-O-19:0)	C41H78O4
1011	707.8	322.3	(+)	35	DDAHNDA	Docosadienoate-hydroxy nonadecanoic acid	FAHFA(22:2-O-19:0)	C41H76O4
1012	699.8	322.3	(+)	35	DHAHNDA	Docosahexaenoic acid-hydroxy nonadecanoic acid	FAHFA(22:6-O-19:0)	C41H68O4
1013	613.8	336.3	(+)	35	MAHAA	Myristic acid-hydroxy arachidic acid	FAHFA(14:0-O-20:0)	C34H66O4
1014	611.8	336.3	(+)	35	MOHAA	Myristoleic acid-hydroxy arachidic acid	FAHFA(14:1-O-20:0)	C34H64O4
1015	627.8	336.3	(+)	35	PDAHAA	Pentadecanoic acid-hydroxy arachidic acid	FAHFA(15:0-O-20:0)	C35H68O4
1016	625.8	336.3	(+)	35	PDEAHAA	Pentadecenoic acid-hydroxy arachidic acid	FAHFA(15:1-O-20:0)	C35H66O4
1017	639.8	336.3	(+)	35	POHAA	Palmitoleic acid-hydroxy arachidic acid	FAHFA(16:1-O-20:0)	C36H68O4
1018	641.8	336.3	(+)	35	PAHAA	Palmitic acid-hydroxy arachidic acid	FAHFA(16:0-O-20:0)	C36H70O4
1019	655.8	336.3	(+)	35	HDAHAA	Heptadecanoic acid-hydroxy arachidic acid	FAHFA(17:0-O-20:0)	C37H72O4
1020	653.8	336.3	(+)	35	HDEAHAA	Heptadecenoic acid-hydroxy arachidic acid	FAHFA(17:1-O-20:0)	C37H70O4
1021	669.8	336.3	(+)	35	SAHAA	Stearic acid-hydroxy arachidic acid	FAHFA(18:0-O-20:0)	C38H74O4
1022	667.8	336.3	(+)	35	OAHAA	Oleic acid-hydroxy arachidic acid	FAHFA(18:1-O-20:0)	C38H72O4
1023	665.8	336.3	(+)	35	LAHAA	Linoleic acid-hydroxy arachidic acid	FAHFA(18:2-O-20:0)	C38H70O4
1024	663.8	336.3	(+)	35	ALAHAA	α-Linolenic acid-hydroxy arachidic acid	FAHFA(18:3-O-20:0)	C38H68O4
1025	661.8	336.3	(+)	35	SDAHAA	Stearidonic acid-hydroxy arachidic acid	FAHFA(18:4-O-20:0)	C38H66O4
1026	697.8	336.3	(+)	35	AAHAA	Arachidic acid-hydroxy arachidic acid	FAHFA(20:0-O-20:0)	C40H78O4
1027	695.8	336.3	(+)	35	EAHAA	Eicosenoic acid-hydroxy arachidic acid	FAHFA(20:1-O-20:0)	C40H76O4
1028	693.8	336.3	(+)	35	EDAHAA	Eicosadienoic acid-hydroxy arachidic acid	FAHFA(20:2-O-20:0)	C40H74O4
1029	691.8	336.3	(+)	35	ETAHAA	Eicosatrienoic acid-hydroxy arachidic acid	FAHFA(20:3-O-20:0)	C40H72O4
1030	689.8	336.3	(+)	35	ARAHAA	Arachidonic acid-hydroxy arachidic acid	FAHFA(20:4-O-20:0)	C40H70O4
1031	687.8	336.3	(+)	35	EPAHAA	Eicosapentaenoic acid-hydroxy arachidic acid	FAHFA(20:5-O-20:0)	C40H68O4
1032	725.8	336.3	(+)	35	BAHAA	Behenic acid-hydroxy arachidic acid	FAHFA(22:0-O-20:0)	C42H82O4
1033	723.8	336.3	(+)	35	DEAHAA	Docosenoic acid-hydroxy arachidic acid	FAHFA(22:1-O-20:0)	C42H80O4
1034	721.8	336.3	(+)	35	DDAHAA	Docosadienoate-hydroxy arachidic acid	FAHFA(22:2-O-20:0)	C42H78O4
1035	713.8	336.3	(+)	35	DHAHAA	Docosahexaenoic acid-hydroxy arachidic acid	FAHFA(22:6-O-20:0)	C42H70O4
1036	627.8	350.3	(+)	35	MAHHEA	Myristic acid-hydroxy heneicosanoic acid	FAHFA(14:0-O-21:0)	C35H68O4
1037	625.8	350.3	(+)	35	MOHHEA	Myristoleic acid-hydroxy heneicosanoic acid	FAHFA(14:1-O-21:0)	C35H66O4
1038	641.8	350.3	(+)	35	PDAHHEA	Pentadecanoic acid-hydroxy heneicosanoic acid	FAHFA(15:0-O-21:0)	C36H70O4
1039	639.8	350.3	(+)	35	PDEAHHEA	Pentadecenoic acid-hydroxy heneicosanoic acid	FAHFA(15:1-O-21:0)	C36H68O4
1040	653.8	350.3	(+)	35	POHHEA	Palmitoleic acid-hydroxy heneicosanoic acid	FAHFA(16:1-O-21:0)	C37H70O4
1041	655.8	350.3	(+)	35	PAHHEA	Palmitic acid-hydroxy heneicosanoic acid	FAHFA(16:0-O-21:0)	C37H72O4
1042	669.8	350.3	(+)	35	HDAHHEA	Heptadecanoic acid-hydroxy heneicosanoic acid	FAHFA(17:0-O-21:0)	C38H74O4
1043	667.8	350.3	(+)	35	HDEAHHEA	Heptadecenoic acid-hydroxy heneicosanoic acid	FAHFA(17:1-O-21:0)	C38H72O4
1044	683.8	350.3	(+)	35	SAHHEA	Stearic acid-hydroxy heneicosanoic acid	FAHFA(18:0-O-21:0)	C39H76O4

1045	681.8	350.3	(+)	35	OAHHEA	Oleic acid-hydroxy heneicosanoic acid	FAHFA(18:1-O-21:0)	$C_{39}H_{74}O_4$
1046	679.8	350.3	(+)	35	LAHHEA	Linoleic acid-hydroxy heneicosanoic acid	FAHFA(18:2-O-21:0)	$C_{39}H_{72}O_4$
1047	677.8	350.3	(+)	35	ALAHHEA	α-Linolenic acid-hydroxy heneicosanoic acid	FAHFA(18:3-O-21:0)	$C_{39}H_{70}O_4$
1048	675.8	350.3	(+)	35	SDAHHEA	Stearidonic acid-hydroxy heneicosanoic acid	FAHFA(18:4-O-21:0)	$C_{39}H_{68}O_4$
1049	711.8	350.3	(+)	35	AAHHEA	Arachidic acid-hydroxy heneicosanoic acid	FAHFA(21:0-O-21:0)	$C_{41}H_{80}O_4$
1050	709.8	350.3	(+)	35	EAHHEA	Eicosenoic acid-hydroxy heneicosanoic acid	FAHFA(20:1-O-21:0)	$C_{41}H_{78}O_4$
1051	707.8	350.3	(+)	35	EDAHHEA	Eicosadienoic acid-hydroxy heneicosanoic acid	FAHFA(20:2-O-21:0)	$C_{41}H_{76}O_4$
1052	705.8	350.3	(+)	35	ETAHHEA	Eicosatrienoic acid-hydroxy heneicosanoic acid	FAHFA(20:3-O-21:0)	$C_{41}H_{74}O_4$
1053	703.8	350.3	(+)	35	ARAHHEA	Arachidonic acid-hydroxy heneicosanoic acid	FAHFA(20:4-O-21:0)	$C_{41}H_{72}O_4$
1054	701.8	350.3	(+)	35	EPAHHEA	Eicosapentaenoic acid-hydroxy heneicosanoic acid	FAHFA(20:5-O-21:0)	$C_{41}H_{70}O_4$
1055	739.8	350.3	(+)	35	BAHHEA	Behenic acid-hydroxy heneicosanoic acid	FAHFA(22:0-O-21:0)	$C_{43}H_{84}O_4$
1056	737.8	350.3	(+)	35	DEAHHEA	Docosenoic acid-hydroxy heneicosanoic acid	FAHFA(22:1-O-21:0)	$C_{43}H_{82}O_4$
1057	735.8	350.3	(+)	35	DDAHHEA	Docosadienoate-hydroxy heneicosanoic acid	FAHFA(22:2-O-21:0)	$C_{43}H_{80}O_4$
1058	727.8	350.3	(+)	35	DHAHHEA	Docosahexaenoic acid-hydroxy heneicosanoic acid	FAHFA(22:6-O-21:0)	$C_{43}H_{72}O_4$
