# Supplementary material for: A dataset of branched fatty acid esters of hydroxy fatty acids diversity in foods
Source: Sci Data. 2023 Nov 10;10:790. doi: 10.1038/s41597-023-02712-z (PMC10638281; doi:10.1038/s41597-023-02712-z)
Supplement: Supplementary file 3 — Supplementary information-4 Table S4. Distribution of FAHFAs [file 41597_2023_2712_MOESM3_ESM.pdf]

Supplementary Table S4. Distribution of FAHFAs (family and regioisomer) detected in 12 food samples and 4 medicinal food samples

NO.	FAHFA name		Edible algaes			Edible fungus	Medicinal foods				Edible plant foods						Edible animal foods	
			Spirulina	*Nostoc commune Vauch*	Kelp (*Laminaria japonica*)	*Lentinus edodes*	Lotus Plumule (*Plumula Nelumbinis*)	Chinese yam (*Rhizoma Disocoreae*)	*Lycium chinense*	*Coptis chinensis*	Tomato (*Lycopersicon esculentum*)	Apple (*Malus pumila*)	Peanut (*Arachis hypogaea)*	Black Sesame (*Sesamum indicum*)	Wheat (*Triticum aestivum*)	Rice (*Oryza sativa*)	Egg	Fish (*Carassius auratus*)
	**Total number of family**		**64**	**51**	**76**	**84**	**70**	**85**	**133**	**97**	**49**	**56**	**18**	**16**	**112**	**30**	**26**	**25**
	**Total number of regioisomers**		**156**	**150**	**214**	**215**	**211**	**276**	**450**	**227**	**119**	**142**	**32**	**40**	**376**	**85**	**61**	**45**
1	FAHFA(14:1-O-14:1)	MOHMO			2													
2	FAHFA(15:0-O-14:1)	PDAHMO							1									
3	FAHFA(15:1-O-14:1)	PDEAHMO							1									
4	FAHFA(16:1-O-14:1)	POHMO			2													
5	FAHFA(16:0-O-14:1)	PAHMO					6		5								1	
6	FAHFA(18:0-O-14:1)	SAHMO															2	
7	FAHFA(18:1-O-14:1)	OAHMO					4		1								2	
8	FAHFA(18:2-O-14:1)	LAHMO			2	3	5	3	5						3		1	
9	FAHFA(18:3-O-14:1)	ALAHMO			2		3		1									1
10	FAHFA(20:0-O-14:1)	AAHMO													1			
11	FAHFA(20:1-O-14:1)	EAHMO													1			
12	FAHFA(20:2-O-14:1)	EDAHMO							1									
13	FAHFA(20:3-O-14:1)	ETAHMO						2										
14	FAHFA(22:2-O-14:1)	DDAHMO						1							2			
15	FAHFA(14:1-O-14:2)	MOHTDA							1									
16	FAHFA(16:0-O-14:2)	PAHTDA					1										1	
17	FAHFA(17:0-O-14:2)	HDAHTDA								1							1	
18	FAHFA(18:0-O-14:2)	SAHTDA															3	
19	FAHFA(18:1-O-14:2)	OAHTDA					1		2	1							1	
20	FAHFA(18:2-O-14:2)	LAHTDA				3	1	4							1			
21	FAHFA(18:3-O-14:2)	ALAHTDA																1
22	FAHFA(20:0-O-14:2)	AAHTDA				2		2	3	1					2		2	
23	FAHFA(20:1-O-14:2)	EAHTDA						1	1								1	
24	FAHFA(22:1-O-14:2)	DEAHTDA							3						3	1		
25	FAHFA(22:2-O-14:2)	DDAHTDA				3	2	3	3						4	1		
26	FAHFA(22:6-O-14:2)	DHAHTDA					1								1		1	
27	FAHFA(14:0-O-14:3)	MAHTTA							1									
28	FAHFA(16:0-O-14:3)	PAHTTA							5									
29	FAHFA(17:0-O-14:3)	HDAHTTA								2								
30	FAHFA(18:0-O-14:3)	SAHTTA							1									
31	FAHFA(18:2-O-14:3)	LAHTTA					1											
32	FAHFA(22:1-O-14:3)	DEAHTTA													2			
33	FAHFA(22:2-O-14:3)	DDAHTTA							1						2			
34	FAHFA(14:0-O-16:1)	MAHPO							1		2							
35	FAHFA(15:0-O-16:1)	PDAHPO	2															
36	FAHFA(16:1-O-16:1)	POHPO		2						2	2	2						
37	FAHFA(16:0-O-16:1)	PAHPO		4			2		8	2					4			
38	FAHFA(17:0-O-16:1)	HDAHPO						1			1							
39	FAHFA(18:0-O-16:1)	SAHPO		2					1			1						
40	FAHFA(18:1-O-16:1)	OAHPO			2		1		8	2					3			
41	FAHFA(18:2-O-16:1)	LAHPO		2		6	4	11	9	8				1	5			
42	FAHFA(18:3-O-16:1)	ALAHPO		2			1		1									
43	FAHFA(20:0-O-16:1)	AAHPO														1		
44	FAHFA(20:2-O-16:1)	EDAHPO				1												
45	FAHFA(20:4-O-16:1)	ARAHPO								1								
46	FAHFA(20:5-O-16:1)	EPAHPO							5									
47	FAHFA(16:1-O-16:2)	POHHDDA			2						1							
48	FAHFA(16:0-O-16:2)	PAHHDDA			2		2		2						2			
49	FAHFA(18:0-O-16:2)	SAHHDDA							1						2			
50	FAHFA(18:1-O-16:2)	OAHHDDA					3		2						2			
51	FAHFA(18:2-O-16:2)	LAHHDDA			2	7	5		3						2			
52	FAHFA(20:1-O-16:2)	EAHHDDA													1			
53	FAHFA(20:2-O-16:2)	EDAHHDDA				3		3	1						2			

54	FAHFA(20:5-O-16:2)	EPAHHDDA				2						4					
55	FAHFA(14:0-O-16:3)	MAHHTA							1								
56	FAHFA(16:0-O-16:3)	PAHHTA						1	2		1						
57	FAHFA(18:0-O-16:3)	SAHHTA							1						2		
58	FAHFA(18:2-O-16:3)	LAHHTA						1									
59	FAHFA(20:1-O-16:3)	EAHHTA							2						2		
60	FAHFA(20:2-O-16:3)	EDAHHTA							1						2		
61	FAHFA(20:5-O-16:3)	EPAHHTA										2			2		
62	FAHFA(22:6-O-16:3)	DHAHHTA				2									1		
63	FAHFA(14:0-O-18:1)	MAHOA	2		5				7						2	3	
64	FAHFA(14:1-O-18:1)	MOHOA				2						1					
65	FAHFA(15:0-O-18:1)	PDAHOA	1		1												
66	FAHFA(15:1-O-18:1)	PDEAHOA				2											
67	FAHFA(16:1-O-18:1)	POHOA				2	5	4	8	6			1		4		
68	FAHFA(16:0-O-18:1)	PAHOA		4	3	6	5	12	7	9	1	5			5	3	
69	FAHFA(17:0-O-18:1)	HDAHOA			1												
70	FAHFA(18:0-O-18:1)	SAHOA		3	2			4	5		2	1					
71	FAHFA(18:1-O-18:1)	OAHOA	1	1	5	3	1	9	7	6	3	5	1	4	5	4	
72	FAHFA(18:2-O-18:1)	LAHOA			1	6	7	9	6	4	2	3	1	4	5	5	
73	FAHFA(18:4-O-18:1)	SDAHOA					2	3									
74	FAHFA(20:0-O-18:1)	AAHOA	2						2		1						
75	FAHFA(20:1-O-18:1)	EAHOA						1	2		1	1			3		
76	FAHFA(20:3-O-18:1)	ETAHOA			1	1											
77	FAHFA(20:5-O-18:1)	EPAHOA							4								
78	FAHFA(22:0-O-18:1)	BAHOA							1								
79	FAHFA(22:1-O-18:1)	DEAHOA							1	1							
80	FAHFA(22:2-O-18:1)	DDAHOA						1	1			1					
81	FAHFA(22:6-O-18:1)	DHAHOA									1	1			6		
82	FAHFA(14:0-O-18:2)	MAHLA			6	3	2	5	5	1	2				3	2	
83	FAHFA(14:1-O-18:2)	MOHLA									1						
84	FAHFA(15:0-O-18:2)	PDAHLA				3		5	3		1				3		
85	FAHFA(15:1-O-18:2)	PDEAHLA							3						2		
86	FAHFA(16:1-O-18:2)	POHLA				3	1		1	4					1		
87	FAHFA(16:0-O-18:2)	PAHLA		6	6	4	7	6	7	3	3	5	3	2	2	3	
88	FAHFA(17:0-O-18:2)	HDAHLA						2	1								
89	FAHFA(17:1-O-18:2)	HDEAHLA						2	2	2					1		
90	FAHFA(18:0-O-18:2)	SAHLA			6			3	4	2	2	2					
91	FAHFA(18:1-O-18:2)	OAHLA			4	6	5	6	6	3		4	2		5	5	
92	FAHFA(18:2-O-18:2)	LAHLA			4	5	7	4	4	2	2	4	2	2	3	3	1
93	FAHFA(20:0-O-18:2)	AAHLA				2		2	4						2		
94	FAHFA(20:1-O-18:2)	EAHLA						2	1						2		
95	FAHFA(20:2-O-18:2)	EDAHLA				1		3	2						1		
96	FAHFA(20:3-O-18:2)	ETAHLA						2									
97	FAHFA(20:4-O-18:2)	ARAHLA			2												
98	FAHFA(20:5-O-18:2)	EPAHLA			2				4								
99	FAHFA(22:0-O-18:2)	BAHLA						2	3	1					1		
100	FAHFA(22:1-O-18:2)	DEAHLA								1					2		
101	FAHFA(22:2-O-18:2)	DDAHLA						4		1							
102	FAHFA(22:6-O-18:2)	DHAHLA					6								6		
103	FAHFA(14:0-O-18:3)	MAHALA			2				2		1				2		
104	FAHFA(15:0-O-18:3)	PDAHALA							2								
105	FAHFA(16:1-O-18:3)	POHALA		1					2	3							
106	FAHFA(16:0-O-18:3)	PAHALA		5	2		3	5	4	3	1				3	2	
107	FAHFA(17:0-O-18:3)	HDAHALA							2								
108	FAHFA(18:0-O-18:3)	SAHALA		4	1				4								
109	FAHFA(18:1-O-18:3)	OAHALA			1	2	3	3	6	4					4	2	
110	FAHFA(18:2-O-18:3)	LAHALA		2	2		4	5	6	3	2	1	2		2	2	
111	FAHFA(20:0-O-18:3)	AAHALA			1				2								
112	FAHFA(20:1-O-18:3)	EAHALA													1		
113	FAHFA(20:5-O-18:3)	EPAHALA						4									
114	FAHFA(22:0-O-18:3)	BAHALA							3								
115	FAHFA(22:2-O-18:3)	DDAHALA							1								
116	FAHFA(22:6-O-18:3)	DHAHALA													3		
117	FAHFA(14:0-O-20:1)	MAHEA			1												
118	FAHFA(14:1-O-20:1)	MOHEA			2	1											

119	FAHFA(15:1-O-20:1)	PDEAHEA																1
120	FAHFA(16:1-O-20:1)	POHEA			2													
121	FAHFA(16:0-O-20:1)	PAHEA			1				1									
122	FAHFA(17:1-O-20:1)	HDEAHEA																1
123	FAHFA(18:1-O-20:1)	OAHEA	1	1														
124	FAHFA(18:2-O-20:1)	LAHEA			3	2									5			
125	FAHFA(18:3-O-20:1)	ALAHEA	3															
126	FAHFA(20:0-O-20:1)	AAHEA	2	1														
127	FAHFA(20:1-O-20:1)	EAHEA																1
128	FAHFA(20:3-O-20:1)	ETAHEA						1										
129	FAHFA(20:5-O-20:1)	EPAHEA			2													
130	FAHFA(22:1-O-20:1)	DEAHEA																2
131	FAHFA(22:2-O-20:1)	DDAHEA																1
132	FAHFA(14:0-O-20:2)	MAHEDA			6													
133	FAHFA(15:1-O-20:2)	PDEAHEDA																1
134	FAHFA(16:0-O-20:2)	PAHEDA				1	2		1						3			
135	FAHFA(17:1-O-20:2)	HDEAHEDA																1
136	FAHFA(18:1-O-20:2)	OAHEDA			4		4								1			
137	FAHFA(18:2-O-20:2)	LAHEDA				1	3	3						2	1			2
138	FAHFA(18:4-O-20:2)	SDAHEDA																1
139	FAHFA(20:0-O-20:2)	AAHEDA	1															3
140	FAHFA(20:1-O-20:2)	EAHEDA																2
141	FAHFA(20:3-O-20:2)	ETAHEDA				1												
142	FAHFA(20:4-O-20:2)	ARAHEDA			4													
143	FAHFA(20:5-O-20:2)	EPAHEDA			3													
144	FAHFA(22:1-O-20:2)	DEAHEDA																3
145	FAHFA(22:2-O-20:2)	DDAHEDA																1
146	FAHFA(15:0-O-22:6)	PDAHDHA	3							1								
147	FAHFA(15:1-O-22:6)	PDEAHDHA								1								
148	FAHFA(16:0-O-22:6)	PAHDHA					2		2						3			
149	FAHFA(17:1-O-22:6)	HDEAHDHA								1								
150	FAHFA(18:1-O-22:6)	OAHDHA							4						6	2		
151	FAHFA(18:3-O-22:6)	ALAHDHA	1												2			
152	FAHFA(14:0-O-20:4)	MAHARA			1													
153	FAHFA(15:0-O-20:4)	PDAHARA			1													
154	FAHFA(16:1-O-20:4)	POHARA			1													
155	FAHFA(16:0-O-20:4)	PAHARA			5		1											
156	FAHFA(18:1-O-20:4)	OAHARA			1		1											
157	FAHFA(18:2-O-20:4)	LAHARA			1		1											
158	FAHFA(18:4-O-20:4)	SDAHARA			1													
159	FAHFA(20:5-O-20:4)	EPAHARA			1													
160	FAHFA(22:0-O-20:4)	BAHARA												1				
161	FAHFA(14:0-O-12:0)	MAHDA	3					1	2	1	2	2						
162	FAHFA(15:0-O-12:0)	PDAHDA									2	2						
163	FAHFA(15:1-O-12:0)	PDEAHDA						1										
164	FAHFA(16:1-O-12:0)	POHDA	2	3				1	1		1	2						
165	FAHFA(16:0-O-12:0)	PAHDA	2	4		2	1	3	6	3	3	3			2		1	1
166	FAHFA(17:0-O-12:0)	HDAHDA	2							1								
167	FAHFA(17:1-O-12:0)	HDEAHDA						1		1								
168	FAHFA(18:0-O-12:0)	SAHDA	4	4				3	4	2	3	3						
169	FAHFA(18:1-O-12:0)	OAHDA	2				2	2	7									
170	FAHFA(18:2-O-12:0)	LAHDA			1	3	2	3	4	3					5			
171	FAHFA(18:3-O-12:0)	ALAHDA					1	2	2									
172	FAHFA(20:0-O-12:0)	AAHDA			1													
173	FAHFA(20:2-O-12:0)	EDAHDA				1												
174	FAHFA(20:5-O-12:0)	EPAHDA			1													
175	FAHFA(22:0-O-12:0)	BAHDA				2												
176	FAHFA(22:2-O-12:0)	DDAHDA						4							1			
177	FAHFA(22:6-O-12:0)	DHAHDA																1
178	FAHFA(14:0-O-14:0)	MAHMA	4	3	6	2		3	2	1	2	3			1	1		
179	FAHFA(14:1-O-14:0)	MOHMA							1	2								
180	FAHFA(15:0-O-14:0)	PDAHMA	2	2		1						3						
181	FAHFA(15:1-O-14:0)	PDEAHMA		1						1								
182	FAHFA(16:1-O-14:0)	POHMA	2	3		1			1	1	2	1						
183	FAHFA(16:0-O-14:0)	PAHMA	5	6	8	3	1	8	4	2	3	7			9			1

184	FAHFA(18:0-O-14:0)	SAHMA	3					3	2	1	3	3			1			
185	FAHFA(18:1-O-14:0)	OAHMA	1	2	6	2	1	3	4	2				1	7			
186	FAHFA(18:2-O-14:0)	LAHMA	1		1	1	1	2	4	2				1	8			
187	FAHFA(18:3-O-14:0)	ALAHMA		2	1	1	1	1	2	1					3			
188	FAHFA(18:4-O-14:0)	SDAHMA			1													
189	FAHFA(20:0-O-14:0)	AAHMA								1								
190	FAHFA(20:2-O-14:0)	EDAHMA				1		1										
191	FAHFA(20:5-O-14:0)	EPAHMA			1													
192	FAHFA(22:6-O-14:0)	DHAHMA																1
193	FAHFA(14:0-O-15:0)	MAHPDA	3	2		1												
194	FAHFA(15:0-O-15:0)	PDAHPDA	2	1						1								
195	FAHFA(15:1-O-15:0)	PDEAHPDA	3							1							1	
196	FAHFA(16:1-O-15:0)	POHPDA	1			1												
197	FAHFA(16:0-O-15:0)	PAHPDA	5	5		1		4	3	2	3				5			
198	FAHFA(17:0-O-15:0)	HDAHPDA	2															
199	FAHFA(17:1-O-15:0)	HDEAHPDA	1														1	
200	FAHFA(18:0-O-15:0)	SAHPDA		4				1		1	3							
201	FAHFA(18:1-O-15:0)	OAHPDA	1			2		1		2					5			
202	FAHFA(18:2-O-15:0)	LAHPDA				1	5	6	2	3					6			
203	FAHFA(18:3-O-15:0)	ALAHPDA				3		1		1								
204	FAHFA(20:5-O-15:0)	EPAHPDA			1													
205	FAHFA(22:1-O-15:0)	DEAHPDA															1	
206	FAHFA(14:0-O-16:0)	MAHPA	3	2	9	2			10						5	1		
207	FAHFA(15:0-O-16:0)	PDAHPA	2	3	1	2		1	11	1					3			
208	FAHFA(15:1-O-16:0)	PDEAHPA	4						3			1			1			
209	FAHFA(16:1-O-16:0)	POHPA	1	3		1			4	4	1				3			
210	FAHFA(16:0-O-16:0)	PAHPA	3	12	10	2		9	10	6	11	10	1		3	2	10	11
211	FAHFA(17:0-O-16:0)	HDAHPA	5							1					3			
212	FAHFA(17:1-O-16:0)	HDEAHPA	2									1			1			
213	FAHFA(18:0-O-16:0)	SAHPA	9	10	9			6		1	11	11					5	2
214	FAHFA(18:1-O-16:0)	OAHPA	2	1	8	4		4	8	10		2		2	8	2	3	1
215	FAHFA(18:2-O-16:0)	LAHPA	1		9	1	8	2	9	5	1	1	1		6	7		
216	FAHFA(18:3-O-16:0)	ALAHPA	1	4	1	6		1	2	2	2				1	2		
217	FAHFA(18:4-O-16:0)	SDAHPA							1									
218	FAHFA(20:0-O-16:0)	AAHPA		1					1									
219	FAHFA(20:1-O-16:0)	EAHPA							1	1					1			
220	FAHFA(20:2-O-16:0)	EDAHPA							1						1			
221	FAHFA(20:3-O-16:0)	ETAHPA							1	2		1			1			
222	FAHFA(20:4-O-16:0)	ARAHPA			1					1								
223	FAHFA(20:5-O-16:0)	EPAHPA			1					2								
224	FAHFA(22:0-O-16:0)	BAHPA							1									
225	FAHFA(22:2-O-16:0)	DDAHPA							1									
226	FAHFA(22:6-O-16:0)	DHAHPA		1							2							2
227	FAHFA(14:0-O-17:0)	MAHHDA	2	1														
228	FAHFA(15:0-O-17:0)	PDAHHDA	2	1						1								
229	FAHFA(15:1-O-17:0)	PDEAHHDA	2							1								
230	FAHFA(16:0-O-17:0)	PAHHDA	2	1						1								
231	FAHFA(17:0-O-17:0)	HDAHHDA	3			2												
232	FAHFA(18:0-O-17:0)	SAHHDA				2	4					3			2			
233	FAHFA(18:1-O-17:0)	OAHHDA	1						2			1		1				
234	FAHFA(18:2-O-17:0)	LAHHDA				9				3		1		1	8			
235	FAHFA(18:3-O-17:0)	ALAHHDA	1															
236	FAHFA(20:0-O-17:0)	AAHHDA	1							1								
237	FAHFA(20:1-O-17:0)	EAHHDA				4	2								4			
238	FAHFA(20:2-O-17:0)	EDAHHDA				2	4								4	2		
239	FAHFA(22:0-O-17:0)	BAHHDA							3									
240	FAHFA(14:0-O-18:0)	MAHSA	2		1			5	2	2								
241	FAHFA(15:0-O-18:0)	PDAHSA	2							1								
242	FAHFA(15:1-O-18:0)	PDEAHSA				2						1						
243	FAHFA(16:1-O-18:0)	POHSA				2	1	1	4	5	1		1		1	1		
244	FAHFA(16:0-O-18:0)	PAHSA	12	9	6			11	9	5	10	11			8		8	
245	FAHFA(17:0-O-18:0)	HDAHSA								2								
246	FAHFA(17:1-O-18:0)	HDEAHSA										1						
247	FAHFA(18:0-O-18:0)	SAHSA	11	9	8			5	4	1	9	5					5	
248	FAHFA(18:1-O-18:0)	OAHSA	1	2	2			4	6	4			1		12		4	1

249	FAHFA(18:2-O-18:0)	LAHSA			2	11			8	3			1	1	14	5		
250	FAHFA(18:3-O-18:0)	ALAHSA				2	2	2	3	3			1					
251	FAHFA(20:0-O-18:0)	AAHSA	1															
252	FAHFA(20:1-O-18:0)	EAHSA										1						
253	FAHFA(20:2-O-18:0)	EDAHSA										1						
254	FAHFA(20:5-O-18:0)	EPAHSA			1					1								
255	FAHFA(22:1-O-18:0)	DEAHSA							1			1						
256	FAHFA(22:2-O-18:0)	DDAHSA										1						
257	FAHFA(22:6-O-18:0)	DHAHSA						1		4	1							2
258	FAHFA(14:0-O-19:0)	MAHNDA				2	4		4						4	4		
259	FAHFA(15:0-O-19:0)	PDAHNDA	1			2	4								4			
260	FAHFA(15:1-O-19:0)	PDEAHNDA				1		1	2						2			
261	FAHFA(16:1-O-19:0)	POHNDA		1		2	4		5	4					4	2		
262	FAHFA(16:0-O-19:0)	PAHNDA		1		2	4	5	7				4	3	4	5		
263	FAHFA(17:0-O-19:0)	HDAHNDA				2	2			1					4			
264	FAHFA(17:1-O-19:0)	HDEAHNDA				2	3								3			
265	FAHFA(18:0-O-19:0)	SAHNDA	1	1					7	2								
266	FAHFA(18:1-O-19:0)	OAHNDA		1		4	8	5	8	4			4	9	6	5	2	
267	FAHFA(18:2-O-19:0)	LAHNDA				4	4	5	5	4	2	4	4	5	4	5		
268	FAHFA(18:3-O-19:0)	ALAHNDA				2	4			5					4	2		
269	FAHFA(20:0-O-19:0)	AAHNDA	1			4	4		6						6			
270	FAHFA(20:1-O-19:0)	EAHNDA				2	4								6			
271	FAHFA(20:2-O-19:0)	EDAHNDA				4	5								6			
272	FAHFA(20:3-O-19:0)	ETAHNDA				1		2										
273	FAHFA(22:0-O-19:0)	BAHNDA				2	4		7			1			7			
274	FAHFA(22:1-O-19:0)	DEAHNDA							1			1			6			
275	FAHFA(22:2-O-19:0)	DDAHNDA					1											
276	FAHFA(22:6-O-19:0)	DHAHNDA							1						1			
277	FAHFA(14:0-O-20:0)	MAHAA		1														
278	FAHFA(14:1-O-20:0)	MOHAA										1						
279	FAHFA(15:0-O-20:0)	PDAHAA	2															
280	FAHFA(16:1-O-20:0)	POHAA				1			1	1		1	1					
281	FAHFA(16:0-O-20:0)	PAHAA			1					1	2		1					
282	FAHFA(18:0-O-20:0)	SAHAA						1			2							
283	FAHFA(18:1-O-20:0)	OAHAA		2	1			1		1	1	1					1	
284	FAHFA(18:2-O-20:0)	LAHAA				2			6	3					3			
285	FAHFA(18:3-O-20:0)	ALAHAA								2								
286	FAHFA(20:0-O-20:0)	AAHAA	2															
287	FAHFA(20:1-O-20:0)	EAHAA						1			1	1					1	
288	FAHFA(20:2-O-20:0)	EDAHAA						1				1					1	
289	FAHFA(20:3-O-20:0)	ETAHAA						2				1						
290	FAHFA(22:1-O-20:0)	DEAHAA										1						
291	FAHFA(15:0-O-21:0)	PDAHHEA	2															
292	FAHFA(16:1-O-21:0)	POHHEA								4								
293	FAHFA(16:0-O-21:0)	PAHHEA		1	1	2	1				1	3			1			
294	FAHFA(18:0-O-21:0)	SAHHEA									1							
295	FAHFA(18:1-O-21:0)	OAHHEA					1		4	1					1			
296	FAHFA(18:2-O-21:0)	LAHHEA				2	3		1	1					3			
297	FAHFA(18:3-O-21:0)	ALAHHEA								1								
298	FAHFA(21:0-O-21:0)	AAHHEA	1															

Highlighted in orange were first reported
