# Supplementary material for: A dataset of branched fatty acid esters of hydroxy fatty acids diversity in foods
Source: Sci Data. 2023 Nov 10;10:790. doi: 10.1038/s41597-023-02712-z (PMC10638281; doi:10.1038/s41597-023-02712-z)
Supplement: Supplementary file 4 — Supplementary information-5 Table S5. Detected FAHFA lists [file 41597_2023_2712_MOESM4_ESM.pdf]

Supplementary Table S5. Detected FAHFA lists in 12 food samples and 4 medicinal food samples

Spirulina

NO.	Normal name	FAHFA ID	*m/z* (DMED labeling)	*m/z* (d_4-DMED labeling)	Product ion	Total number	*t1*	*t2*	*t3*	*t4*	*t5*	*t6*	*t7*	*t8*	*t9*	*t10*	*t11*	*t12*
1	FAHFA(15:0-O-16:1)	PDAHPO	565.5	569.5	278.2	**2**	2507	2534										
2	FAHFA(14:0-O-18:1)	MAHOA	579.5	583.5	306.2	**2**	2584	2610										
3	FAHFA(15:0-O-18:1)	PDAHOA	593.5	597.5	306.2	**1**	2672											
4	FAHFA(18:1-O-18:1)	OAHOA	633.5	637.5	306.2	**1**	2797											
5	FAHFA(20:0-O-18:1)	AAHOA	663.5	667.5	306.2	**2**	2764	2813										
6	FAHFA(18:1-O-20:1)	OAHEA	661.5	666.5	334.2	**1**	2950											
7	FAHFA(18:3-O-20:1)	ALAHEA	637.5	641.5	334.2	**3**	2634	2662	2678									
8	FAHFA(20:0-O-20:1)	AAHEA	691.5	695.5	334.2	**2**	2922	2978										
9	FAHFA(20:0-O-20:2)	AAHEDA	689.5	693.5	332.2	**1**	2827											
10	FAHFA(15:0-O-22:6)	PDAHDHA	639.5	643.5	352.2	**3**	2686	2711	2772									
11	FAHFA(18:3-O-22:6)	ALAHDHA	675.5	679.5	352.2	**1**	3058											
12	FAHFA(14:0-O-12:0)	MAHDA	497.5	501.5	224.2	**3**	2000	2025	2204									
13	FAHFA(16:1-O-12:0)	POHDA	523.5	527.5	224.2	**2**	2052	2223										
14	FAHFA(16:0-O-12:0)	PAHDA	525.5	529.5	224.2	**2**	2204	2407										
15	FAHFA(17:0-O-12:0)	HDAHDA	539.5	543.5	224.2	**2**	2368	2506										
16	FAHFA(18:0-O-12:0)	SAHDA	553.5	557.5	224.2	**4**	1985	2398	2412	2599								
17	FAHFA(18:1-O-12:0)	OAHDA	551.5	556.5	224.2	**2**	2413	2447										
18	FAHFA(14:0-O-14:0)	MAHMA	525.5	529.5	252.2	**4**	2147	2171	2236	2409								
19	FAHFA(15:0-O-14:0)	PDAHMA	539.5	543.5	252.2	**2**	2456	2483										
20	FAHFA(16:1-O-14:0)	POHMA	551.5	555.5	252.2	**2**	2201	2429										
21	FAHFA(16:0-O-14:0)	PAHMA	553.5	557.5	252.2	**5**	2358	2437	2555	2581	2609							
22	FAHFA(18:0-O-14:0)	SAHMA	581.5	585.5	252.2	**3**	2730	2763	2789									
23	FAHFA(18:1-O-14:0)	OAHMA	579.5	583.5	252.2	**1**	2611											
24	FAHFA(18:2-O-14:0)	LAHMA	577.5	581.5	252.2	**1**	2108											
25	FAHFA(14:0-O-15:0)	MAHPDA	539.5	543.5	266.2	**3**	2455	2484	2513									
26	FAHFA(15:0-O-15:0)	PDAHPDA	553.5	557.5	266.2	**2**	2551	2581										
27	FAHFA(15:1-O-15:0)	PDEAHPDA	551.5	555.5	266.2	**3**	2385	2442	2471									
28	FAHFA(16:1-O-15:0)	POHPDA	565.5	569.5	266.2	**1**	2499											
29	FAHFA(16:0-O-15:0)	PAHPDA	567.5	571.5	266.2	**5**	2282	2311	2645	2672	2694							
30	FAHFA(17:0-O-15:0)	HDAHPDA	581.5	585.5	266.2	**2**	2331	2723										
31	FAHFA(17:1-O-15:0)	HDEAHPDA	579.5	583.5	266.2	**1**	2562											
32	FAHFA(18:1-O-15:0)	OAHPDA	593.5	597.5	266.2	**1**	2673											
33	FAHFA(14:0-O-16:0)	MAHPA	553.6	557.6	280.3	**3**	2552	2580	2609									
34	FAHFA(15:0-O-16:0)	PDAHPA	567.6	571.6	280.3	**2**	2643	2671										
35	FAHFA(15:1-O-16:0)	PDEAHPA	565.6	569.6	280.3	**4**	2487	2516	2540	2568								
36	FAHFA(16:1-O-16:0)	POHPA	579.6	583.6	280.3	**1**	2593											
37	FAHFA(16:0-O-16:0)	PAHPA	581.6	585.6	280.3	**3**	2726	2765	2794									
38	FAHFA(17:0-O-16:0)	HDAHPA	595.6	599.6	280.3	**5**	2405	2430	2461	2823	2852							
39	FAHFA(17:1-O-16:0)	HDEAHPA	593.6	597.6	280.3	**2**	2653	2680										
40	FAHFA(18:0-O-16:0)	SAHPA	609.6	613.6	280.3	**9**	2494	2688	2752	2802	2827	2852	2916	2892	3011			
41	FAHFA(18:1-O-16:0)	OAHPA	607.6	611.6	280.3	**2**	2763	2793										
42	FAHFA(18:2-O-16:0)	LAHPA	605.6	609.6	280.3	**1**	2655											
43	FAHFA(18:3-O-16:0)	ALAHPA	603.6	607.6	280.3	**1**	2550											
44	FAHFA(14:0-O-17:0)	MAHHDA	567.6	571.6	294.3	**2**	2642	2670										
45	FAHFA(15:0-O-17:0)	PDAHHDA	581.6	585.6	294.3	**2**	2726	2765										
46	FAHFA(15:1-O-17:0)	PDEAHHDA	579.6	583.6	294.3	**2**	2579	2632										
47	FAHFA(16:0-O-17:0)	PAHHDA	581.6	585.6	294.3	**2**	2726	2764										
48	FAHFA(17:0-O-17:0)	HDAHHDA	609.6	613.6	294.3	**3**	2520	2910	2937									
49	FAHFA(18:1-O-17:0)	OAHHDA	621.6	625.6	294.3	**1**	2852											
50	FAHFA(18:3-O-17:0)	ALAHHDA	617.6	621.6	294.3	**1**	2614											
51	FAHFA(20:0-O-17:0)	AAHHDA	651.6	655.6	294.3	**1**	2725											
52	FAHFA(14:0-O-18:0)	MAHSA	581.8	585.8	308.3	**2**	2763	2793										
53	FAHFA(15:0-O-18:0)	PDAHSA	595.8	599.8	308.3	**2**	2826	2854										
54	FAHFA(16:0-O-18:0)	PAHSA	609.8	613.8	308.3	**12**	2663	2678	2698	2715	2743	2767	2796	2826	2912	2939	2965	3013
55	FAHFA(18:0-O-18:0)	SAHSA	637.8	641.8	308.3	**11**	2840	2856	2878	2894	2916	2937	2962	2989	3014	3043	3117	
56	FAHFA(18:1-O-18:0)	OAHSA	635.8	639.8	308.3	**1**	2961											
57	FAHFA(20:0-O-18:0)	AAHSA	665.8	669.8	308.3	**1**	2931											
58	FAHFA(15:0-O-19:0)	PDAHNDA	609.8	613.8	322.3	**1**	2903											
59	FAHFA(18:0-O-19:0)	SAHNDA	651.8	655.8	322.3	**1**	2721											
60	FAHFA(20:0-O-19:0)	AAHNDA	679.8	683.8	322.3	**1**	3010											
61	FAHFA(15:0-O-20:0)	PDAHAA	623.8	627.8	336.3	**2**	2717	3016										
62	FAHFA(20:0-O-20:0)	AAHAA	693.8	697.8	336.3	**2**	2925	3085										
63	FAHFA(15:0-O-21:0)	PDAHHEA	637.8	641.8	350.3	**2**	2637	2664										
64	FAHFA(21:0-O-21:0)	AAHHEA	707.8	713.8	350.3	**1**	3034											

t , retention index

Nostoc commune Vauch

NO.	Normal name	FAHFA ID	*m/z* (DMED labeling)	*m/z* (d_4-DMED labeling)	Product ion	Total number	*t1*	*t2*	*t3*	*t4*	*t5*	*t6*	*t7*	*t8*	*t9*	*t10*	*t11*	*t12*
1	FAHFA(16:1-O-16:1)	POHPO	577.5	581.5	278.2	**2**	2334	2350										
2	FAHFA(16:0-O-16:1)	PAHPO	579.5	583.5	278.2	**4**	2485	2511	2532	2627								
3	FAHFA(18:0-O-16:1)	SAHPO	607.5	611.5	278.2	**2**	2670	2693										
4	FAHFA(18:2-O-16:1)	LAHPO	603.5	607.5	278.2	**2**	2368	2385										
5	FAHFA(18:3-O-16:1)	ALAHPO	601.5	605.5	278.2	**2**	2241	2256										
6	FAHFA(16:0-O-18:1)	PAHOA	607.5	611.5	306.2	**4**	2604	2639	2785	2799								
7	FAHFA(18:0-O-18:1)	SAHOA	635.5	639.5	306.2	**3**	2785	2814	2963									
8	FAHFA(18:1-O-18:1)	OAHOA	633.5	637.5	306.2	**1**	2785											
9	FAHFA(16:0-O-18:2)	PAHLA	605.5	609.5	304.2	**6**	2478	2520	2560	2577	2606	2671						
10	FAHFA(16:1-O-18:3)	POHALA	601.5	605.5	302.2	**1**	2284											
11	FAHFA(16:0-O-18:3)	PAHALA	603.5	607.5	302.2	**5**	2412	2438	2465	2547	2589							
12	FAHFA(18:0-O-18:3)	SAHALA	631.5	635.5	302.2	**4**	2600	2624	2651	2728								
13	FAHFA(18:2-O-18:3)	LAHALA	627.5	631.5	302.2	**2**	2196	2320										
14	FAHFA(18:1-O-20:1)	OAHEA	661.5	665.5	334.2	**1**	2939											
15	FAHFA(20:0-O-20:1)	AAHEA	691.5	695.5	334.2	**1**	2911											
16	FAHFA(16:1-O-12:0)	POHDA	523.5	527.5	224.2	**3**	2028	2049	2164									
17	FAHFA(16:0-O-12:0)	PAHDA	525.5	529.5	224.2	**4**	2201	2341	2400	2460								
18	FAHFA(18:0-O-12:0)	SAHDA	553.5	557.5	224.2	**4**	1982	2010	2393	2537								
19	FAHFA(14:0-O-14:0)	MAHMA	525.5	529.5	252.2	**3**	2163	2226	2399									
20	FAHFA(15:0-O-14:0)	PDAHMA	539.5	543.5	252.2	**2**	2481	2500										
21	FAHFA(15:1-O-14:0)	PDEAHMA	537.5	541.5	252.2	**1**	2327											
22	FAHFA(16:1-O-14:0)	POHMA	551.5	555.5	252.2	**3**	2190	2331	2417									
23	FAHFA(16:0-O-14:0)	PAHMA	553.5	557.5	252.2	**6**	2352	2423	2506	2543	2597	2654						
24	FAHFA(18:1-O-14:0)	OAHMA	579.5	583.5	252.2	**2**	2520	2599										
25	FAHFA(18:3-O-14:0)	ALAHMA	575.5	579.5	252.2	**2**	2243	2323										
26	FAHFA(14:0-O-15:0)	MAHPDA	539.5	543.5	266.2	**2**	2481	2500										
27	FAHFA(15:0-O-15:0)	PDAHPDA	553.5	557.5	266.2	**1**	2540											
28	FAHFA(16:0-O-15:0)	PAHPDA	567.5	571.5	266.2	**5**	1875	1891	1908	2567		2692						
29	FAHFA(18:0-O-15:0)	SAHPDA	595.5	599.5	266.2	**4**	2027	2049	2748	2868								
30	FAHFA(14:0-O-16:0)	MAHPA	553.6	557.6	280.3	**2**	2417	2542										
31	FAHFA(15:0-O-16:0)	PDAHPA	567.6	571.6	280.3	**3**	2514	2635	2666									
32	FAHFA(16:1-O-16:0)	POHPA	579.6	583.6	280.3	**3**	1940	2339	2612									
33	FAHFA(16:0-O-16:0)	PAHPA	581.6	585.6	280.3	**12**	2079	2505	2528	2560	2582	2610	2634	2667	2697	2729	2781	2833
34	FAHFA(18:0-O-16:0)	SAHPA	609.6	613.6	280.3	**10**	2259	2685	2742	2763	2785	2812	2840	2870	2903	2950		
35	FAHFA(18:1-O-16:0)	OAHPA	607.6	611.6	280.3	**1**	2779											
36	FAHFA(18:3-O-16:0)	ALAHPA	603.6	607.6	280.3	**4**	1878	2244	2517	2576								
37	FAHFA(20:0-O-16:0)	AAHPA	637.6	641.6	280.3	**1**	2750											
38	FAHFA(22:6-O-16:0)	DHAHPA	653.6	657.6	280.3	**1**	2785											
39	FAHFA(14:0-O-17:0)	MAHHDA	567.6	571.6	294.3	**1**	2637											
40	FAHFA(15:0-O-17:0)	PDAHHDA	581.6	585.6	294.3	**1**	2727											
41	FAHFA(16:0-O-17:0)	PAHHDA	581.6	585.6	294.3	**1**	2726											
42	FAHFA(16:0-O-18:0)	PAHSA	609.8	613.8	308.3	**9**	2661	2675	2699	2717	2736	2759	2784	2812	2953			
43	FAHFA(18:0-O-18:0)	SAHSA	637.8	641.8	308.3	**9**	2827	2843	2866	2883	2904	2926	2950	2977	3106			
44	FAHFA(18:1-O-18:0)	OAHSA	635.8	639.8	308.3	**2**	2788	2950										
45	FAHFA(16:1-O-19:0)	POHNDA	621.8	625.8	322.3	**1**	2076											
46	FAHFA(16:0-O-19:0)	PAHNDA	623.8	627.8	322.3	**1**	2232											
47	FAHFA(18:0-O-19:0)	SAHNDA	651.8	655.8	322.3	**1**	2416											
48	FAHFA(18:1-O-19:0)	OAHNDA	649.8	653.8	322.3	**1**	2899											
49	FAHFA(14:0-O-20:0)	MAHAA	609.8	613.8	336.3	**1**	2810											
50	FAHFA(18:1-O-20:0)	OAHAA	663.8	667.8	336.3	**2**	2771	2945										
51	FAHFA(16:0-O-21:0)	PAHHEA	651.8	655.8	350.3	**1**	2961											

t , retention index

Kelp (*Laminaria japonica*)

NO.	Normal name	FAHFA ID	*m/z* (DMED labeling)	*m/z* (d_4-DMED labeling)	Product ion	Total number	*t1*	*t2*	*t3*	*t4*	*t5*	*t6*	*t7*	*t8*	*t9*	*t10*
1	FAHFA(14:1-O-14:1)	MOHMO	521.5	525.5	250.2	**2**	2274	2296								
2	FAHFA(16:1-O-14:1)	POHMO	549.5	553.5	250.2	**2**	2468	2491								
3	FAHFA(18:2-O-14:1)	LAHMO	575.5	579.5	250.2	**2**	2474	2500								
4	FAHFA(18:3-O-14:1)	ALAHMO	573.5	577.5	250.2	**2**	2253	2355								
5	FAHFA(18:1-O-16:1)	OAHPO	605.5	609.5	278.2	**2**	2516	2630								
6	FAHFA(16:1-O-16:2)	POHHDA	575.5	579.5	276.2	**2**	1845	1878								
7	FAHFA(16:0-O-16:2)	PAHHDA	577.5	581.5	276.2	**2**	2471	2509								
8	FAHFA(18:2-O-16:2)	LAHHDA	601.5	605.5	276.2	**2**	1863	1893								
9	FAHFA(14:0-O-18:1)	MAHOA	579.5	583.5	306.2	**5**	2362	2411	2494	2509	2616					
10	FAHFA(15:0-O-18:1)	PDAHOA	593.5	597.5	306.2	**1**	2505									
11	FAHFA(16:0-O-18:1)	PAHOA	607.5	611.5	306.2	**3**	2594	2675	2689							
12	FAHFA(17:0-O-18:1)	HDAHOA	621.5	625.5	306.2	**1**	2686									
13	FAHFA(18:0-O-18:1)	SAHOA	635.5	639.5	306.2	**2**	2760	2852								
14	FAHFA(18:1-O-18:1)	OAHOA	633.5	637.5	306.2	**5**	2549	2593	2630	2674	2688					
15	FAHFA(18:2-O-18:1)	LAHOA	631.5	635.5	306.2	**1**	2453									
16	FAHFA(20:3-O-18:1)	ETAHOA	657.5	661.5	306.2	**1**	2490									
17	FAHFA(14:0-O-18:2)	MAHLA	577.5	581.5	304.2	**6**	2279	2321	2361	2371	2409	2476				
18	FAHFA(16:0-O-18:2)	PAHLA	605.5	609.5	304.2	**6**	2470	2483	2512	2551	2594	2662				
19	FAHFA(18:0-O-18:2)	SAHLA	633.5	637.5	304.2	**6**	2648	2664	2691	2727	2745	2764				
20	FAHFA(18:1-O-18:2)	OAHLA	631.5	635.5	304.2	**4**	2480	2515	2554	2596						
21	FAHFA(18:2-O-18:2)	LAHLA	629.5	633.5	304.2	**4**	2329	2367	2408	2451						
22	FAHFA(20:4-O-18:2)	ARAHLA	653.5	657.5	304.2	**2**	2341	2385								
23	FAHFA(20:5-O-18:2)	EPAHLA	651.5	655.5	304.2	**2**	2222	2265								
24	FAHFA(14:0-O-18:3)	MAHALA	575.5	579.5	304.2	**2**	2211	2282								
25	FAHFA(16:0-O-18:3)	PAHALA	603.5	607.5	302.2	**2**	2401	2479								
26	FAHFA(18:0-O-18:3)	SAHALA	631.5	635.5	302.2	**1**	2662									
27	FAHFA(18:1-O-18:3)	OAHALA	629.5	633.5	302.2	**1**	2482									
28	FAHFA(18:2-O-18:3)	LAHALA	627.5	631.5	302.2	**2**	2263	2332								
29	FAHFA(20:0-O-18:3)	AAHALA	659.5	663.5	302.2	**1**	2823									
30	FAHFA(14:0-O-20:1)	MAHEA	607.5	611.5	334.2	**1**	2448									
31	FAHFA(14:1-O-20:1)	MOHEA	605.5	609.5	334.2	**2**	2803	2827								
32	FAHFA(16:1-O-20:1)	POHEA	633.5	637.5	334.2	**2**	2968	2984								
33	FAHFA(16:0-O-20:1)	PAHEA	635.5	639.5	334.2	**1**	2624									
34	FAHFA(18:2-O-20:1)	LAHEA	659.5	663.5	334.2	**3**	2489	2966	2986							
35	FAHFA(20:5-O-20:1)	EPAHEA	681.5	685.5	334.2	**2**	2800	2823								
36	FAHFA(14:0-O-20:2)	MAHEDA	605.5	609.5	332.2	**6**	1971	2000	2042	2085	2096	2431				
37	FAHFA(18:1-O-20:2)	OAHEDA	659.5	663.5	332.2	**4**	2156	2189	2231	2283						
38	FAHFA(20:4-O-20:2)	ARAHEDA	681.5	685.5	332.2	**4**	1971	2006	2046	2091						
39	FAHFA(20:5-O-20:2)	EPAHEDA	679.5	683.5	332.2	**3**	1879	1906	1939							
40	FAHFA(14:0-O-20:4)	MAHARA	601.5	605.5	328.2	**1**	2424									
41	FAHFA(15:0-O-20:4)	PDAHARA	615.3	619.3	328.2	**1**	2520									
42	FAHFA(16:1-O-20:4)	POHARA	627.5	631.5	328.2	**1**	2436									
43	FAHFA(16:0-O-20:4)	PAHARA	629.5	633.5	328.2	**5**	2501	2527	2554	2611	2632					
44	FAHFA(18:1-O-20:4)	OAHARA	655.5	659.5	328.2	**1**	2616									
45	FAHFA(18:2-O-20:4)	LAHARA	653.5	657.5	328.2	**1**	2472									
46	FAHFA(18:4-O-20:4)	SDAHARA	649.5	653.5	328.2	**1**	2233									
47	FAHFA(20:5-O-20:4)	EPAHARA	675.5	679.5	328.2	**1**	2298									
48	FAHFA(18:2-O-12:0)	LAHDA	549.5	553.5	224.2	**1**	1780									
49	FAHFA(20:0-O-12:0)	AAHDA	581.5	585.5	224.2	**1**	1980									
50	FAHFA(20:5-O-12:0)	EPAHDA	571.5	575.5	224.2	**1**	1743									
51	FAHFA(14:0-O-14:0)	MAHMA	525.5	529.5	252.2	**6**	2154	2194	2223	2248	2342	2396				
52	FAHFA(16:0-O-14:0)	PAHMA	553.5	557.5	252.2	**8**	2353	2393	2423	2449	2477	2505	2541	2594		
53	FAHFA(18:1-O-14:0)	OAHMA	579.5	583.5	252.2	**6**	2397	2425	2476	2511	2543	2599				
54	FAHFA(18:2-O-14:0)	LAHMA	577.5	581.5	252.2	**1**	2452									
55	FAHFA(18:3-O-14:0)	ALAHMA	575.5	579.5	252.2	**1**	2316									
56	FAHFA(18:4-O-14:0)	SDAHMA	573.5	577.5	252.2	**1**	2210									
57	FAHFA(20:5-O-14:0)	EPAHMA	599.5	603.5	252.2	**1**	2280									
58	FAHFA(20:5-O-15:0)	EPAHPDA	613.5	617.5	266.2	**1**	2104									
59	FAHFA(14:0-O-16:0)	MAHPA	553.6	557.6	280.3	**9**	2339	2360	2383	2410	2441	2474	2504	2539	2594	
60	FAHFA(15:0-O-16:0)	PDAHPA	567.6	571.6	280.3	**1**	2686									
61	FAHFA(16:0-O-16:0)	PAHPA	581.6	585.6	280.3	**10**	2503	2533	2551	2575	2600	2629	2659	2688	2722	2764
62	FAHFA(18:0-O-16:0)	SAHPA	609.6	613.6	280.3	**9**	2676	2709	2730	2747	2766	2796	2825	2853	2887	
63	FAHFA(18:1-O-16:0)	OAHPA	607.6	611.6	280.3	**8**	2533	2551	2574	2598	2628	2654	2723	2766		
64	FAHFA(18:2-O-16:0)	LAHPA	605.6	609.6	280.3	**9**	2321	2357	2385	2406	2426	2454	2483	2509	2635	
65	FAHFA(18:3-O-16:0)	ALAHPA	603.6	607.6	280.3	**1**	2509									
66	FAHFA(20:4-O-16:0)	ARAHPA	629.6	633.6	280.3	**1**	2593									
67	FAHFA(20:5-O-16:0)	EPAHPA	627.6	631.6	280.3	**1**	2472									
68	FAHFA(14:0-O-18:0)	MAHSA	581.8	585.8	308.3	**1**	2596									
69	FAHFA(16:0-O-18:0)	PAHSA	609.8	613.8	308.3	**6**	2596	2684	2704	2722	2743	2764				
70	FAHFA(18:0-O-18:0)	SAHSA	637.8	641.8	308.3	**8**	2760	2807	2824	2846	2862	2884	2905	2930		
71	FAHFA(18:1-O-18:0)	OAHSA	635.8	639.8	308.3	**2**	2593	2761								
72	FAHFA(18:2-O-18:0)	LAHSA	633.8	637.8	308.3	**2**	2491	2631								
73	FAHFA(20:5-O-18:0)	EPAHSA	655.8	659.8	308.3	**1**	2386									
74	FAHFA(16:0-O-20:0)	PAHAA	637.8	641.8	336.3	**1**	1905									
75	FAHFA(18:1-O-20:0)	OAHAA	663.8	667.8	336.3	**1**	1919									
76	FAHFA(16:0-O-21:0)	PAHHEA	651.8	655.8	350.3	**1**	2985									

t, retention index

Lentinus edodes

NO.	Normal name	FAHFA ID	*m/z* (DMED labeling)	*m/z* (d_4-DMED labeling)	Product ion	Total number	*t1*	*t2*	*t3*	*t4*	*t5*	*t6*	*t7*	*t8*	*t9*	*t10*	*t11*
1	FAHFA(18:2-O-14:1)	LAHMO	575.5	579.5	250.2	**3**	2132	2311	2362								
2	FAHFA(18:2-O-14:2)	LAHTDA	573.5	577.5	248.2	**3**	1861	1875	2243								
3	FAHFA(20:0-O-14:2)	AAHTDA	605.5	609.5	248.2	**2**	2559	2596									
4	FAHFA(22:2-O-14:2)	DDAHTDA	629.5	633.5	248.2	**3**	2327	2428	2467								
5	FAHFA(18:2-O-16:1)	LAHPO	603.5	607.5	278.2	**6**	2272	2327	2364	2413	2492	2541					
6	FAHFA(20:2-O-16:1)	EDAHPO	631.5	635.5	278.2	**1**	2664										
7	FAHFA(18:2-O-16:2)	LAHHDA	601.5	605.5	276.2	**7**	2183	2258	2286	2333	2383	2421	2448				
8	FAHFA(20:2-O-16:2)	EDAHHDA	629.5	633.5	276.2	**3**	2427	2467	2565								
9	FAHFA(20:5-O-16:2)	EPAHHDA	623.5	627.5	276.2	**2**	2292	2339									
10	FAHFA(22:6-O-16:3)	DHAHHTA	647.5	651.5	274.2	**2**	2152	2196									
11	FAHFA(14:1-O-18:1)	MOHOA	577.5	581.5	306.2	**2**	2207	2516									
12	FAHFA(15:1-O-18:1)	PDEAHOA	591.5	595.5	306.2	**2**	2302	2601									
13	FAHFA(16:1-O-18:1)	POHOA	605.5	609.5	306.2	**2**	2398	2654									
14	FAHFA(16:0-O-18:1)	PAHOA	607.5	611.5	306.2	**6**	2398	2559	2599	2654	2731	2747					
15	FAHFA(18:1-O-18:1)	OAHOA	633.5	637.5	306.2	**3**	2563	2597	2663								
16	FAHFA(18:2-O-18:1)	LAHOA	631.5	635.5	306.2	**6**	2423	2441	2465	2502	2527	2664					
17	FAHFA(20:3-O-18:1)	ETAHOA	689.5	693.5	306.2	**1**	2878										
18	FAHFA(14:0-O-18:2)	MAHLA	577.5	581.5	304.2	**3**	2379	2424	2529								
19	FAHFA(15:0-O-18:2)	PDAHLA	591.5	595.5	304.2	**3**	2473	2516	2615								
20	FAHFA(16:1-O-18:2)	POHLA	603.5	607.5	304.2	**3**	2389	2436	2519								
21	FAHFA(16:0-O-18:2)	PAHLA	605.5	609.5	304.2	**4**	2464	2561	2597	2659							
22	FAHFA(18:1-O-18:2)	OAHLA	631.5	635.5	304.2	**6**	2427	2468	2534	2562	2598	2673					
23	FAHFA(18:2-O-18:2)	LAHLA	629.5	633.5	304.2	**5**	2327	2428	2469	2536	2565						
24	FAHFA(20:0-O-18:2)	AAHLA	661.5	665.5	304.2	**2**	1861	1874									
25	FAHFA(20:2-O-18:2)	EDAHLA	657.5	661.5	304.2	**1**	2127										
26	FAHFA(18:1-O-18:3)	OAHALA	629.5	633.5	302.2	**2**	2424	2464									
27	FAHFA(14:1-O-20:1)	MOHEA	605.5	609.5	334.2	**1**	2655										
28	FAHFA(18:2-O-20:1)	LAHEA	659.5	663.5	334.2	**2**	2057	2416									
29	FAHFA(16:0-O-20:2)	PAHEDA	633.5	637.5	332.2	**1**	2644										
30	FAHFA(18:2-O-20:2)	LAHEDA	657.5	661.5	332.2	**1**	2537										
31	FAHFA(20:3-O-20:2)	ETAHEDA	683.5	687.5	332.2	**1**	1966										
32	FAHFA(16:0-O-12:0)	PAHDA	525.5	529.5	224.2	**2**	2190	2467									
33	FAHFA(18:2-O-12:0)	LAHDA	549.5	553.5	224.2	**3**	2078	2268	2325								
34	FAHFA(20:2-O-12:0)	EDAHDA	577.5	581.5	224.2	**1**	2515										
35	FAHFA(22:0-O-12:0)	BAHDA	609.5	613.5	224.2	**2**	2249	2286									
36	FAHFA(14:0-O-14:0)	MAHMA	525.5	529.5	252.2	**2**	2405	2464									
37	FAHFA(15:0-O-14:0)	PDAHMA	539.5	543.5	252.2	**1**	2556										
38	FAHFA(16:1-O-14:0)	POHMA	551.5	555.5	252.2	**1**	2477										
39	FAHFA(16:0-O-14:0)	PAHMA	553.5	557.5	252.2	**3**	2349	2623	2632								
40	FAHFA(18:1-O-14:0)	OAHMA	579.5	583.5	252.2	**2**	2514	2634									
41	FAHFA(18:2-O-14:0)	LAHMA	577.5	581.5	252.2	**1**	2515										
42	FAHFA(18:3-O-14:0)	ALAHMA	575.5	579.5	252.2	**1**	2409										
43	FAHFA(20:2-O-14:0)	EDAHMA	605.5	609.5	252.2	**1**	2651										
44	FAHFA(14:0-O-15:0)	MAHPDA	539.5	543.5	266.2	**1**	2559										
45	FAHFA(16:1-O-15:0)	POHPDA	565.5	569.5	266.2	**1**	2563										
46	FAHFA(16:0-O-15:0)	PAHPDA	567.5	571.5	266.2	**1**	2711										
47	FAHFA(18:1-O-15:0)	OAHPDA	593.5	597.5	266.2	**2**	2601	2714									
48	FAHFA(18:2-O-15:0)	LAHPDA	591.5	595.5	266.2	**1**	2600										
49	FAHFA(18:3-O-15:0)	ALAHPDA	589.5	593.5	266.2	**3**	2276	2312	2502								
50	FAHFA(14:0-O-16:0)	MAHPA	553.6	557.6	280.3	**2**	2319	2629									
51	FAHFA(15:0-O-16:0)	PDAHPA	567.6	571.6	280.3	**2**	2413	2711									
52	FAHFA(16:1-O-16:0)	POHPA	579.6	583.6	280.3	**1**	2635										
53	FAHFA(16:0-O-16:0)	PAHPA	581.6	585.6	280.3	**2**	2503	2724									
54	FAHFA(18:1-O-16:0)	OAHPA	607.6	611.6	280.3	**4**	2364	2508	2652	2725							
55	FAHFA(18:2-O-16:0)	LAHPA	605.6	609.6	280.3	**1**	2651										
56	FAHFA(18:3-O-16:0)	ALAHPA	603.6	607.6	280.3	**6**	2223	2249	2367	2405	2562	2593					
57	FAHFA(17:0-O-17:0)	HDAHHDA	609.6	613.6	294.3	**2**	2183	2248									
58	FAHFA(18:0-O-17:0)	SAHHDA	623.6	627.6	294.3	**2**	2289	2336									
59	FAHFA(18:2-O-17:0)	LAHHDA	619.6	623.6	294.3	**9**	1976	2011	2053	2096	2136	2190	2401	2447	2722		
60	FAHFA(20:1-O-17:0)	EAHHDA	649.6	653.6	294.3	**4**	2152	2193	2304	2350							
61	FAHFA(20:2-O-17:0)	EDAHHDA	647.6	651.6	294.3	**2**	2152	2194									
62	FAHFA(15:1-O-18:0)	PDEAHSA	593.8	597.8	308.3	**2**	2304	2444									
63	FAHFA(16:1-O-18:0)	POHSA	607.8	611.8	308.3	**2**	2398	2534									
64	FAHFA(18:2-O-18:0)	LAHSA	633.8	637.8	308.3	**11**	2085	2125	2166	2443	2465	2523	2560	2590	2622	2660	2761
65	FAHFA(18:3-O-18:0)	ALAHSA	631.8	635.8	308.3	**2**	2310	2450									
66	FAHFA(14:0-O-19:0)	MAHNDA	595.8	599.8	322.3	**2**	2108	2140									
67	FAHFA(15:0-O-19:0)	PDAHNDA	609.8	613.8	322.3	**2**	2188	2248									
68	FAHFA(15:1-O-19:0)	PDEAHNDA	607.8	611.8	322.3	**1**	2656										
69	FAHFA(16:1-O-19:0)	POHNDA	621.8	625.8	322.3	**2**	2123	2162									
70	FAHFA(16:0-O-19:0)	PAHNDA	623.8	627.8	322.3	**2**	2290	2338									
71	FAHFA(17:0-O-19:0)	HDAHNDA	637.8	641.8	322.3	**2**	2384	2438									
72	FAHFA(17:1-O-19:0)	HDEAHNDA	635.8	639.8	322.3	**2**	2211	2264									
73	FAHFA(18:1-O-19:0)	OAHNDA	649.8	653.8	322.3	**4**	2148	2196	2302	2350							
74	FAHFA(18:2-O-19:0)	LAHNDA	647.8	651.8	322.3	**4**	2096	2112	2153	2196							
75	FAHFA(18:3-O-19:0)	ALAHNDA	645.8	649.8	322.3	**2**	2059	2103									
76	FAHFA(20:0-O-19:0)	AAHNDA	679.8	683.8	322.3	**4**	2072	2112	2625	2653							
77	FAHFA(20:1-O-19:0)	EAHNDA	677.8	681.8	322.3	**2**	2479	2528									
78	FAHFA(20:2-O-19:0)	EDAHNDA	675.8	679.8	322.3	**4**	2251	2273	2330	2382							
79	FAHFA(20:3-O-19:0)	ETAHNDA	673.8	677.8	322.3	**1**	2254										
80	FAHFA(22:0-O-19:0)	BAHNDA	707.8	711.8	322.3	**2**	2724	2756									
81	FAHFA(16:1-O-20:0)	POHAA	635.8	639.8	336.3	**1**	2792										
82	FAHFA(18:2-O-20:0)	LAHAA	661.8	665.8	336.3	**2**	2918	2983									
83	FAHFA(16:0-O-21:0)	PAHHEA	651.8	655.8	350.3	**2**	2380	2422									
84	FAHFA(18:2-O-21:0)	LAHHEA	675.8	679.8	350.3	**2**	2249	2286									

t , retention index

Lotus Plumule (*Plumula Nelumbinis*)

NO.	Normal name	FAHFA ID	*m/z* (DMED labeling)	*m/z* (d_4-DMED labeling)	Product ion	Total number	*t1*	*t2*	*t3*	*t4*	*t5*	*t6*	*t7*	*t8*
1	FAHFA(16:0-O-14:1)	PAHMO	551.5	555.5	250.2	**6**	1795	1814	1879	2374	2468	2498		
2	FAHFA(18:1-O-14:1)	OAHMO	577.5	581.5	250.2	**4**	1891	2226	2378	2510				
3	FAHFA(18:2-O-14:1)	LAHMO	575.5	579.5	250.2	**5**	1796	2225	2308	2328	2357			
4	FAHFA(18:3-O-14:1)	ALAHMO	573.5	577.5	250.2	**3**	2094	2224	2372					
5	FAHFA(16:0-O-14:2)	PAHTDA	549.5	553.5	248.2	**1**	2380							
6	FAHFA(18:1-O-14:2)	OAHTDA	575.5	579.5	248.2	**1**	2393							
7	FAHFA(18:2-O-14:2)	LAHTDA	573.5	577.5	248.2	**1**	2238							
8	FAHFA(22:2-O-14:2)	DDAHTDA	629.5	633.5	248.2	**2**	2418	2465						
9	FAHFA(22:6-O-14:2)	DHAHTDA	621.5	625.5	248.2	**1**	2115							
10	FAHFA(18:2-O-14:3)	LAHTTA	571.5	575.5	246.2	**1**	2127							
11	FAHFA(16:0-O-16:1)	PAHPO	579.5	583.5	278.2	**2**	1934	2571						
12	FAHFA(18:1-O-16:1)	OAHPO	605.5	609.5	278.2	**1**	1942							
13	FAHFA(18:2-O-16:1)	LAHPO	603.5	607.5	278.2	**4**	1829	1858	2423	2602				
14	FAHFA(18:3-O-16:1)	ALAHPO	601.5	605.5	278.2	**1**	1801							
15	FAHFA(16:0-O-16:2)	PAHHDA	577.5	581.5	276.2	**2**	1846	1909						
16	FAHFA(18:1-O-16:2)	OAHHDA	603.5	607.5	276.2	**3**	1814	1859	1919					
17	FAHFA(18:2-O-16:2)	LAHHDA	601.5	605.5	276.2	**5**	1813	2251	2302	2380	2469			
18	FAHFA(16:1-O-18:1)	POHOA	605.5	609.5	306.2	**5**	1880	1924	1961	2377	2562			
19	FAHFA(16:0-O-18:1)	PAHOA	607.5	611.5	306.2	**5**	2020	2567	2605	2637	2692			
20	FAHFA(18:1-O-18:1)	OAHOA	633.5	637.5	306.2	**1**	2029							
21	FAHFA(18:2-O-18:1)	LAHOA	631.5	635.5	306.2	**7**	1910	2417	2463	2498	2540	2570	2712	
22	FAHFA(18:4-O-18:1)	SDAHOA	627.5	631.5	306.2	**2**	2170	2291						
23	FAHFA(14:0-O-18:2)	MAHLA	577.5	581.5	304.2	**2**	2372	2419						
24	FAHFA(16:1-O-18:2)	POHLA	603.5	607.5	304.2	**1**	2386							
25	FAHFA(16:0-O-18:2)	PAHLA	605.5	609.5	304.2	**7**	1975	2237	2463	2524	2567	2607	2711	
26	FAHFA(18:1-O-18:2)	OAHLA	631.5	635.5	304.2	**5**	2418	2465	2568	2608	2715			
27	FAHFA(18:2-O-18:2)	LAHLA	629.5	633.5	304.2	**7**	1877	2327	2378	2418	2464	2537	2579	
28	FAHFA(22:6-O-18:2)	DHAHLA	677.5	681.5	304.2	**6**	2312	2332	2405	2424	2477	2530		
29	FAHFA(16:0-O-18:3)	PAHALA	603.5	607.5	302.2	**3**	2414	2458	2589					
30	FAHFA(18:1-O-18:3)	OAHALA	629.5	633.5	302.2	**3**	2274	2419	2463					
31	FAHFA(18:2-O-18:3)	LAHALA	627.5	631.5	302.2	**4**	2274	2311	2411	2457				
32	FAHFA(16:0-O-20:2)	PAHEDA	633.5	637.5	332.2	**2**	2151	2197						
33	FAHFA(18:1-O-20:2)	OAHEDA	659.5	663.5	332.2	**4**	2034	2066	2162	2202				
34	FAHFA(18:2-O-20:2)	LAHEDA	657.5	661.5	332.2	**3**	1962	2033	2066					
35	FAHFA(16:0-O-22:6)	PAHDHA	653.5	657.5	352.2	**2**	2486	2525						
36	FAHFA(16:0-O-20:4)	PAHARA	629.5	633.5	328.2	**1**	2108							
37	FAHFA(18:1-O-20:4)	OAHARA	655.5	659.5	328.2	**1**	2119							
38	FAHFA(18:2-O-20:4)	LAHARA	653.5	657.5	328.2	**1**	2023							
39	FAHFA(16:0-O-12:0)	PAHDA	525.5	529.5	224.2	**1**	2409							
40	FAHFA(18:1-O-12:0)	OAHDA	551.5	555.5	224.2	**2**	2267	2422						
41	FAHFA(18:2-O-12:0)	LAHDA	549.5	553.5	224.2	**2**	1790	2267						
42	FAHFA(18:3-O-12:0)	ALAHDA	547.5	551.5	224.2	**1**	2134							
43	FAHFA(16:0-O-14:0)	PAHMA	553.5	557.5	252.2	**1**	2598							
44	FAHFA(18:1-O-14:0)	OAHMA	579.5	583.5	252.2	**1**	2604							
45	FAHFA(18:2-O-14:0)	LAHMA	577.5	581.5	252.2	**1**	2460							
46	FAHFA(18:3-O-14:0)	ALAHMA	575.5	579.5	252.2	**1**	2328							
47	FAHFA(18:2-O-15:0)	LAHPDA	591.5	599.5	266.2	**5**	1942	1965	2030	2067	2113			
48	FAHFA(18:2-O-16:0)	LAHPA	605.6	609.6	280.3	**8**	2365	2412	2461	2489	2519	2583	2644	2696
49	FAHFA(18:0-O-17:0)	SAHHDA	623.6	627.6	294.3	**4**	2207	2226	2284	2336				
50	FAHFA(20:1-O-17:0)	EAHHDA	649.6	653.6	294.3	**2**	2218	2239						
51	FAHFA(20:2-O-17:0)	EDAHHDA	647.6	651.6	294.3	**4**	2077	2093	2146	2197				
52	FAHFA(16:1-O-18:0)	POHSA	607.8	611.8	308.3	**1**	2510							
53	FAHFA(18:3-O-18:0)	ALAHSA	631.8	635.8	308.3	**2**	2281	2418						
54	FAHFA(14:0-O-19:0)	MAHNDA	595.8	599.8	322.3	**4**	2020	2038	2087	2134				
55	FAHFA(15:0-O-19:0)	PDAHNDA	609.8	613.8	322.3	**4**	2111	2128	2184	2230				
56	FAHFA(16:1-O-19:0)	POHNDA	621.8	625.8	322.3	**4**	2041	2057	2108	2152				
57	FAHFA(16:0-O-19:0)	PAHNDA	623.8	627.8	322.3	**4**	2204	2223	2282	2332				
58	FAHFA(17:0-O-19:0)	HDAHNDA	637.8	641.8	322.3	**2**	2299	2321						
59	FAHFA(17:1-O-19:0)	HDEAHNDA	635.8	639.8	322.3	**3**	2038	2126	2140					
60	FAHFA(18:1-O-19:0)	OAHNDA	649.8	653.8	322.3	**8**	2073	2091	2143	2193	2216	2236	2292	2343
61	FAHFA(18:2-O-19:0)	LAHNDA	647.8	651.8	322.3	**4**	2072	2091	2143	2193				
62	FAHFA(18:3-O-19:0)	ALAHNDA	645.8	679.8	322.3	**4**	1960	1970	2027	2067				
63	FAHFA(20:0-O-19:0)	AAHNDA	679.8	683.8	322.3	**4**	2572	2591	2646	2695				
64	FAHFA(20:1-O-19:0)	EAHNDA	677.8	681.8	322.3	**4**	2397	2414	2472	2522				
65	FAHFA(20:2-O-19:0)	EDAHNDA	675.8	679.8	322.3	**5**	2157	2177	2251	2268	2326			
66	FAHFA(22:0-O-19:0)	BAHNDA	707.8	711.8	322.3	**4**	2742	2759	2816	2859				
67	FAHFA(22:2-O-19:0)	DDAHNDA	703.8	707.8	322.3	**1**	2372							
68	FAHFA(16:0-O-21:0)	PAHHEA	651.8	655.8	350.3	**1**	2326							
69	FAHFA(18:1-O-21:0)	OAHHEA	677.8	681.8	350.3	**1**	2329							
70	FAHFA(18:2-O-21:0)	LAHHEA	675.8	679.8	350.3	**3**	2191	2231	2274					

t , retention index

Chinese yam (*Rhizoma Disocoreae*)

NO.	Normal name	FAHFA ID	*m/z* (DMED labeling)	*m/z* (d_4-DMED labeling)	Product ion	Total number	*t1*	*t2*	*t3*	*t4*	*t5*	*t6*	*t7*	*t8*	*t9*	*t10*	*t11*	*t12*
1	FAHFA(18:2-O-14:1)	LAHMO	575.5	579.5	250.2	**3**	2262	2314	2350									
2	FAHFA(20:3-O-14:1)	ETAHMO	601.5	605.5	250.2	**2**	1947	1979										
3	FAHFA(22:2-O-14:1)	DDAHMO	631.5	635.5	250.2	**1**	2584											
4	FAHFA(18:2-O-14:2)	LAHTDA	573.5	577.5	248.2	**4**	2069	2092	2216	2241								
5	FAHFA(20:0-O-14:2)	AAHTDA	605.5	609.5	248.2	**2**	2569	2609										
6	FAHFA(20:1-O-14:2)	EAHTDA	603.5	607.5	248.2	**1**	2435											
7	FAHFA(22:2-O-14:2)	DDAHTDA	629.5	633.5	248.2	**3**	2370	2421	2464									
8	FAHFA(17:0-O-16:1)	HDAHPO	593.5	597.5	278.2	**1**	2244											
9	FAHFA(18:2-O-16:1)	LAHPO	603.5	607.5	278.2	**11**	2322	2344	2362	2388	2426	2458	2491	2522	2549	2577	2606	
10	FAHFA(20:2-O-16:2)	EDAHHDA	629.5	633.5	276.2	**3**	2420	2462	2581									
11	FAHFA(16:0-O-16:3)	PAHHTA	575.5	579.5	276.2	**1**	2206											
12	FAHFA(18:2-O-16:3)	LAHHTA	599.5	603.5	276.2	**1**	2074											
13	FAHFA(16:1-O-18:1)	POHOA	605.5	609.5	306.2	**4**	2386	2568	2612	2705								
14	FAHFA(16:0-O-18:1)	PAHOA	607.5	611.5	306.2	**12**	2301	2336	2387	2523	2573	2609	2645	2671	2696	2718	2807	2848
15	FAHFA(18:0-O-18:1)	SAHOA	635.5	639.5	306.2	**4**	2748	2784	2964	3002								
16	FAHFA(18:1-O-18:1)	OAHOA	633.5	637.5	306.2	**9**	2575	2611	2648	2670	2695	2719	2747	2812	2852			
17	FAHFA(18:2-O-18:1)	LAHOA	631.5	635.5	306.2	**9**	2370	2424	2462	2527	2549	2578	2611	2672	2719			
18	FAHFA(18:4-O-18:1)	SDAHOA	627.5	631.5	306.2	**3**	2179	2296	2333									
19	FAHFA(20:1-O-18:1)	EAHOA	661.5	665.5	306.2	**1**	2778											
20	FAHFA(22:2-O-18:1)	DDAHOA	687.5	691.5	306.2	**1**	2777											
21	FAHFA(14:0-O-18:2)	MAHLA	577.5	581.5	304.2	**5**	2287	2331	2371	2422	2532							
22	FAHFA(15:0-O-18:2)	PDAHLA	591.5	595.5	304.2	**5**	2428	2474	2521	2579	2621							
23	FAHFA(16:0-O-18:2)	PAHLA	605.5	609.5	304.2	**6**	2479	2525	2570	2611	2673	2716						
24	FAHFA(17:0-O-18:2)	HDAHLA	619.5	623.5	304.2	**2**	2663	2699										
25	FAHFA(17:1-O-18:2)	HDEAHLA	617.5	621.5	304.2	**2**	2480	2528										
26	FAHFA(18:0-O-18:2)	SAHLA	633.5	637.5	304.2	**3**	2747	2784	2882									
27	FAHFA(18:1-O-18:2)	OAHLA	631.5	635.5	304.2	**6**	2422	2461	2527	2574	2612	2722						
28	FAHFA(18:2-O-18:2)	LAHLA	629.5	632.5	304.2	**4**	2369	2422	2465	2583								
29	FAHFA(20:0-O-18:2)	AAHLA	661.5	665.5	304.2	**2**	2906	2938										
30	FAHFA(20:1-O-18:2)	EAHLA	659.5	663.5	304.2	**2**	2741	2779										
31	FAHFA(20:2-O-18:2)	EDAHLA	657.5	661.5	304.2	**3**	2125	2596	2639									
32	FAHFA(20:3-O-18:2)	ETAHLA	655.5	659.5	304.2	**2**	2067	2100										
33	FAHFA(22:0-O-18:2)	BAHLA	689.5	693.5	304.2	**2**	3052	3083										
34	FAHFA(22:2-O-18:2)	DDAHLA	685.5	689.5	304.2	**4**	2056	2105	2227	2267								
35	FAHFA(16:0-O-18:3)	PAHALA	603.5	607.5	302.2	**5**	2412	2430	2458	2480	2593							
36	FAHFA(18:1-O-18:3)	OAHALA	629.5	633.5	302.2	**3**	2317	2418	2462									
37	FAHFA(18:2-O-18:3)	LAHALA	627.5	631.5	302.2	**5**	2271	2314	2364	2413	2455							
38	FAHFA(20:5-O-18:3)	EPAHALA	649.5	653.5	302.2	**4**	2261	2279	2317	2356								
39	FAHFA(20:3-O-20:1)	ETAHEA	685.5	689.5	334.2	**1**	2650											
40	FAHFA(18:2-O-20:2)	LAHEDA	657.5	661.5	332.2	**3**	2548	2586	2740									
41	FAHFA(14:0-O-12:0)	MAHDA	497.5	501.5	224.2	**1**	2204											
42	FAHFA(15:1-O-12:0)	PDEAHDA	509.5	513.5	224.2	**1**	1878											
43	FAHFA(16:1-O-12:0)	POHDA	523.5	527.5	224.2	**1**	2046											
44	FAHFA(16:0-O-12:0)	PAHDA	525.5	529.5	224.2	**3**	2204	2407	2464									
45	FAHFA(17:1-O-12:0)	HDEAHDA	537.5	541.5	224.2	**1**	2051											
46	FAHFA(18:0-O-12:0)	SAHDA	553.5	557.5	224.2	**3**	2393	2599	2608									
47	FAHFA(18:1-O-12:0)	OAHDA	551.5	555.5	224.2	**2**	2420	2472										
48	FAHFA(18:2-O-12:0)	LAHDA	549.5	553.5	224.2	**3**	2142	2260	2321									
49	FAHFA(18:3-O-12:0)	ALAHDA	547.5	551.5	224.2	**2**	1973	2135										
50	FAHFA(22:2-O-12:0)	DDAHDA	605.5	609.5	224.2	**4**	2540	2571	2608	2717								
51	FAHFA(14:0-O-14:0)	MAHMA	525.5	529.5	252.2	**3**	2162	2255	2403									
52	FAHFA(16:0-O-14:0)	PAHMA	553.5	557.5	252.2	**8**	1875	2349	2456	2481	2517	2549	2602	2660				
53	FAHFA(18:0-O-14:0)	SAHMA	581.5	585.5	252.2	**3**	2548	2783	2833									
54	FAHFA(18:1-O-14:0)	OAHMA	579.5	583.5	252.2	**3**	2459	2610	2667									
55	FAHFA(18:2-O-14:0)	LAHMA	577.5	581.5	252.2	**2**	2458	2522										
56	FAHFA(18:3-O-14:0)	ALAHMA	575.5	579.5	252.2	**1**	2325											
57	FAHFA(20:2-O-14:0)	EDAHMA	605.5	609.5	252.2	**1**	2705											
58	FAHFA(16:0-O-15:0)	PAHPDA	567.5	571.5	266.2	**4**	2169	2433	2698	2751								
59	FAHFA(18:0-O-15:0)	SAHPDA	595.5	599.5	266.2	**1**	2916											
60	FAHFA(18:1-O-15:0)	OAHPDA	593.5	597.5	266.2	**1**	2756											
61	FAHFA(18:2-O-15:0)	LAHPDA	591.5	595.5	266.2	**6**	2044	2064	2088	2526	2556	2615						
62	FAHFA(18:3-O-15:0)	ALAHPDA	589.5	593.5	266.2	**1**	2483											
63	FAHFA(15:0-O-16:0)	PDAHPA	567.6	571.6	280.3	**1**	2751											
64	FAHFA(16:0-O-16:0)	PAHPA	581.6	585.6	280.3	**9**	2512	2565	2586	2614	2643	2671	2702	2785	2836			
65	FAHFA(18:0-O-16:0)	SAHPA	609.6	613.6	280.3	**6**	2704	2744	2791	2816	2843	2994						
66	FAHFA(18:1-O-16:0)	OAHPA	607.6	611.6	280.3	**4**	2522	2704	2789	2840								
67	FAHFA(18:2-O-16:0)	LAHPA	605.6	609.6	280.3	**2**	2652	2705										
68	FAHFA(18:3-O-16:0)	ALAHPA	603.6	607.6	280.3	**1**	2581											
69	FAHFA(14:0-O-18:0)	MAHSA	581.8	585.8	308.3	**5**	2483	2521	2557	2585	2612							
70	FAHFA(16:1-O-18:0)	POHSA	607.8	611.8	308.3	**1**	2529											
71	FAHFA(16:0-O-18:0)	PAHSA	609.8	613.8	308.3	**11**	2668	2700	2742	2761	2789	2817	2846	2873	2905	2951	2996	
72	FAHFA(18:0-O-18:0)	SAHSA	637.8	641.8	308.3	**5**	2830	2868	2902	2925	2950							
73	FAHFA(18:1-O-18:0)	OAHSA	635.8	639.8	308.3	**4**	2699	2717	2760	2787								
74	FAHFA(18:3-O-18:0)	ALAHSA	631.8	635.8	308.3	**2**	2436	2719										
75	FAHFA(22:6-O-18:0)	DHAHSA	681.8	685.8	308.3	**1**	2421											
76	FAHFA(15:1-O-19:0)	PDEAHNDA	607.8	611.8	322.3	**1**	2716											
77	FAHFA(16:0-O-19:0)	PAHNDA	623.8	627.8	322.3	**5**	2216	2233	2251	2288	2318							
78	FAHFA(18:1-O-19:0)	OAHNDA	649.8	653.8	322.3	**5**	2304	2323	2347	2363	2402							
79	FAHFA(18:2-O-19:0)	LAHNDA	647.8	651.8	322.3	**5**	2155	2176	2205	2226	2250							
80	FAHFA(20:3-O-19:0)	ETAHNDA	673.8	677.8	322.3	**2**	2248	2286										
81	FAHFA(18:0-O-20:0)	SAHAA	665.8	669.8	336.3	**1**	2984											
82	FAHFA(18:1-O-20:0)	OAHAA	663.8	667.7	336.3	**1**	2911											
83	FAHFA(20:1-O-20:0)	EAHAA	691.8	695.8	336.3	**1**	3056											
84	FAHFA(20:2-O-20:0)	EDAHAA	689.8	693.8	336.3	**1**	2911											
85	FAHFA(20:3-O-20:0)	ETAHAA	687.5	691.5	336.3	**2**	2659	2782										

t, retention index

Lycium chinense

NO.	Normal name	FAHFA ID	*m/z* (DMED labeling)	*m/z* (d_4-DMED labeling)	Product ion	Total number	*t1*	*t2*	*t3*	*t4*	*t5*	*t6*	*t7*	*t8*	*t9*	*t10*	*t11*
1	FAHFA(15:0-O-14:1)	PDAHMO	537.5	541.5	250.2	**1**	1935										
2	FAHFA(15:1-O-14:1)	PDEAHMO	535.5	539.5	250.2	**1**	1919										
3	FAHFA(16:0-O-14:1)	PAHMO	551.5	555.5	250.2	**5**	2298	2372	2422	2467	2506						
4	FAHFA(18:1-O-14:1)	OAHMO	577.5	581.5	250.2	**1**	2429										
5	FAHFA(18:2-O-14:1)	LAHMO	575.5	579.5	250.2	**5**	2140	2231	2276	2320	2364						
6	FAHFA(18:3-O-14:1)	ALAHMO	573.5	577.5	250.2	**1**	2134										
7	FAHFA(20:2-O-14:1)	EDAHMO	603.5	607.5	250.2	**1**	2383										
8	FAHFA(14:1-O-14:2)	MOHTDA	519.5	523.5	248.2	**1**	2345										
9	FAHFA(18:1-O-14:2)	OAHTDA	575.5	579.5	248.2	**2**	2069	2355									
10	FAHFA(20:0-O-14:2)	AAHTDA	605.5	609.5	248.2	**3**	2026	2565	2612								
11	FAHFA(20:1-O-14:2)	EAHTDA	603.5	607.5	248.2	**1**	2387										
12	FAHFA(22:1-O-14:2)	DEAHTDA	631.5	635.5	248.2	**3**	2480	2568	2615								
13	FAHFA(22:2-O-14:2)	DDAHTDA	629.5	633.5	248.2	**3**	2336	2425	2472								
14	FAHFA(14:0-O-14:3)	MAHTTA	519.5	523.5	246.2	**1**	2025										
15	FAHFA(16:0-O-14:3)	PAHTTA	547.5	551.5	246.2	**5**	2073	2125	2167	2220	2337						
16	FAHFA(18:0-O-14:3)	SAHTTA	575.5	579.5	246.2	**1**	2125										
17	FAHFA(22:2-O-14:3)	DDAHTTA	627.5	631.5	246.2	**1**	2313										
18	FAHFA(14:0-O-16:1)	MAHPO	551.5	555.5	278.2	**1**	2559										
19	FAHFA(16:0-O-16:1)	PAHPO	579.5	583.5	278.2	**8**	2432	2491	2536	2572	2636	2683	2713	2743			
20	FAHFA(18:0-O-16:1)	SAHPO	607.5	611.5	278.2	**1**	2903										
21	FAHFA(18:1-O-16:1)	OAHPO	605.5	609.5	278.2	**8**	2436	2532	2578	2609	2642	2688	2718	2749			
22	FAHFA(18:2-O-16:1)	LAHPO	603.5	607.5	278.2	**9**	2292	2344	2380	2432	2501	2524	2550	2577	2609		
23	FAHFA(18:3-O-16:1)	ALAHPO	601.5	605.5	278.2	**1**	2482										
24	FAHFA(20:5-O-16:1)	EPAHPO	625.5	629.5	278.2	**5**	2222	2241	2311	2335	2363						
25	FAHFA(16:0-O-16:2)	PAHHDA	577.5	581.5	276.2	**2**	2457	2530									
26	FAHFA(18:0-O-16:2)	SAHHDA	605.5	609.5	276.2	**1**	2568										
27	FAHFA(18:1-O-16:2)	OAHHDA	603.5	607.5	276.2	**2**	2459	2541									
28	FAHFA(18:2-O-16:2)	LAHHDA	601.5	605.5	276.2	**3**	2308	2357	2388								
29	FAHFA(20:2-O-16:2)	EDAHHDA	629.5	633.5	276.2	**1**	2426										
30	FAHFA(14:0-O-16:3)	MAHHTA	547.5	551.5	274.2	**1**	1955										
31	FAHFA(16:0-O-16:3)	PAHHTA	575.5	579.5	274.2	**2**	2034	2125									
32	FAHFA(18:0-O-16:3)	SAHHTA	603.5	607.5	274.2	**1**	2456										
33	FAHFA(20:1-O-16:3)	EAHHTA	629.5	633.5	274.2	**2**	2426	2459									
34	FAHFA(20:2-O-16:3)	EDAHHTA	627.5	631.5	274.2	**1**	2314										
35	FAHFA(14:0-O-18:1)	MAHOA	579.5	583.5	306.2	**7**	2389	2429	2468	2490	514.0 2840	2539	2625	2678			
36	FAHFA(16:1-O-18:1)	POHOA	605.5	609.5	306.2	**8**	2401	2439	2478	2526	2568	2614	2637	2701			
37	FAHFA(16:0-O-18:1)	PAHOA	607.5	611.5	306.2	**7**	2572	2612	2649	2692	2712	2799	2845				
38	FAHFA(18:0-O-18:1)	SAHOA	635.5	639.5	306.2	**5**	2614	2749	2786	2856	2997						
39	FAHFA(18:1-O-18:1)	OAHOA	633.5	637.5	306.2	**7**	2473	2578	2614	2649	2691	2808	2849				
40	FAHFA(18:2-O-18:1)	LAHOA	631.5	635.5	306.2	**6**	2443	2474	2510	2551	2679	2719					
41	FAHFA(20:0-O-18:1)	AAHOA	663.5	667.5	306.2	**2**	2897	2935									
42	FAHFA(20:1-O-18:1)	EAHOA	661.5	665.5	306.2	**2**	2744	2777									
43	FAHFA(20:5-O-18:1)	EPAHOA	653.5	657.5	306.2	**4**	2417	2463	2588	2636							
44	FAHFA(22:0-O-18:1)	BAHOA	691.5	695.5	306.2	**1**	3039										
45	FAHFA(22:1-O-18:1)	DEAHOA	689.5	693.5	306.2	**1**	2883										
46	FAHFA(22:2-O-18:1)	DDAHOA	687.5	691.5	306.2	**1**	2763										
47	FAHFA(14:0-O-18:2)	MAHLA	577.5	581.5	304.2	**5**	2296	2342	2377	2427	2538						
48	FAHFA(15:0-O-18:2)	PDAHLA	591.5	595.5	304.2	**3**	2442	2475	2526								
49	FAHFA(15:1-O-18:2)	PDEAHLA	589.5	593.5	304.5	**3**	2279	2326	2358								
50	FAHFA(16:1-O-18:2)	POHLA	603.5	607.5	304.5	**1**	2387										
51	FAHFA(16:0-O-18:2)	PAHLA	605.5	609.5	304.5	**7**	2472	2486	2531	2567	2614	2678	2715				
52	FAHFA(17:0-O-18:2)	HDAHLA	619.5	623.5	304.65	**1**	2656										
53	FAHFA(17:1-O-18:2)	HDEAHLA	617.5	621.5	304.74	**2**	2484	2524									
54	FAHFA(18:0-O-18:2)	SAHLA	633.5	637.5	304.83	**4**	2569	2746	2783	2876							
55	FAHFA(18:1-O-18:2)	OAHLA	631.5	635.5	304.92	**6**	2426	2480	2534	2569	2616	2722					
56	FAHFA(18:2-O-18:2)	LAHLA	629.5	633.5	305.01	**4**	2336	2384	2425	2472							
57	FAHFA(20:0-O-18:2)	AAHLA	661.5	665.5	305.1	**4**	2813	2900	2934	3021							
58	FAHFA(20:1-O-18:2)	EAHLA	659.5	663.5	305.19	**1**	2731										
59	FAHFA(20:2-O-18:2)	EDAHLA	657.5	661.5	305.28	**2**	2122	2598									
60	FAHFA(20:5-O-18:2)	EPAHLA	651.5	655.5	305.37	**4**	2411	2431	2488	2538							
61	FAHFA(22:0-O-18:2)	BAHLA	689.5	693.5	305.46	**3**	2961	3043	3070								
62	FAHFA(14:0-O-18:3)	MAHALA	575.5	579.5	302.2	**2**	2233	2268									
63	FAHFA(15:0-O-18:3)	PDAHALA	589.5	593.5	302.2	**2**	1958	2358									
64	FAHFA(16:1-O-18:3)	POHALA	601.5	605.5	302.2	**2**	2250	2279									
65	FAHFA(16:0-O-18:3)	PAHALA	603.5	607.5	302.2	**4**	2387	2420	2456	2595							
66	FAHFA(17:0-O-18:3)	HDAHALA	617.5	621.5	302.2	**2**	2515	2549									
67	FAHFA(18:0-O-18:3)	SAHALA	631.5	635.5	302.2	**4**	2568	2602	2637	2764							
68	FAHFA(18:1-O-18:3)	OAHALA	629.5	633.5	302.2	**6**	2313	2396	2427	2459	2495	2604					
69	FAHFA(18:2-O-18:3)	LAHALA	627.5	631.5	302.2	**6**	2259	2284	2313	2348	2420	2462					
70	FAHFA(20:0-O-18:3)	AAHALA	659.5	663.5	302.2	**2**	2747	2804									
71	FAHFA(22:0-O-18:3)	BAHALA	687.5	691.5	302.2	**3**	2898	2924	2956								
72	FAHFA(22:2-O-18:3)	DDAHALA	683.5	687.5	302.2	**1**	2597										
73	FAHFA(16:0-O-20:1)	PAHEA	635.5	639.5	334.2	**1**	2945										
74	FAHFA(16:0-O-20:2)	PAHEDA	633.5	637.5	332.2	**1**	2819										
75	FAHFA(16:0-O-22:6)	PAHDHA	653.5	657.5	352.2	**2**	2125	2143									
76	FAHFA(18:1-O-22:6)	OAHDHA	679.5	683.5	352.2	**4**	2132	2147	2499	2538							
77	FAHFA(14:0-O-12:0)	MAHDA	497.5	501.5	224.2	**2**	2029	2224									
78	FAHFA(16:1-O-12:0)	POHDA	523.5	527.5	224.2	**1**	2243										
79	FAHFA(16:0-O-12:0)	PAHDA	525.5	529.5	224.2	**6**	1918	2222	2243	2269	2423	2480					
80	FAHFA(18:0-O-12:0)	SAHDA	553.5	557.5	224.2	**4**	2413	2432	2609	2667							
81	FAHFA(18:1-O-12:0)	OAHDA	551.5	555.5	224.2	**7**	1901	1922	2234	2253	2276	2430	2490				
82	FAHFA(18:2-O-12:0)	LAHDA	549.5	553.5	224.2	**4**	2082	2114	2283	2340							
83	FAHFA(18:3-O-12:0)	ALAHDA	547.5	551.5	224.2	**2**	1965	2141									
84	FAHFA(14:0-O-14:0)	MAHMA	525.5	529.5	252.2	**2**	2151	2418									
85	FAHFA(14:1-O-14:0)	MOHMA	523.5	527.5	252.2	**1**	2255										
86	FAHFA(16:1-O-14:0)	POHMA	551.5	555.5	252.2	**1**	2435										
87	FAHFA(16:0-O-14:0)	PAHMA	553.5	557.5	252.2	**4**	2366	2395	2610	2665							
88	FAHFA(18:0-O-14:0)	SAHMA	581.5	585.5	252.2	**2**	2789	2837									
89	FAHFA(18:1-O-14:0)	OAHMA	579.5	583.5	252.2	**4**	1910	2375	2618	2675							
90	FAHFA(18:2-O-14:0)	LAHMA	577.5	581.5	252.2	**4**	2230	2260	2473	2533							
91	FAHFA(18:3-O-14:0)	ALAHMA	575.5	579.5	252.2	**2**	2096	2342									
92	FAHFA(16:0-O-15:0)	PAHPDA	567.5	571.5	266.2	**3**	2442	2699	2752								
93	FAHFA(18:2-O-15:0)	LAHPDA	591.5	595.5	266.2	**2**	2303	2619									
94	FAHFA(14:0-O-16:0)	MAHPA	553.6	557.6	280.3	**10**	2334	2376	2398	2430	2461	2488	2520	2555	2608	2663	
95	FAHFA(15:0-O-16:0)	PDAHPA	567.6	571.6	280.3	**11**	2390	2429	2454	2471	2497	2528	2555	2581	2612	2701	2752
96	FAHFA(15:1-O-16:0)	PDEAHPA	565.6	569.6	280.3	**3**	2313	2602	2659								
97	FAHFA(16:1-O-16:0)	POHPA	579.6	583.6	280.3	**4**	2349	2413	2621	2678							
98	FAHFA(16:0-O-16:0)	PAHPA	581.6	585.6	280.3	**10**	2523	2569	2589	2616	2645	2675	2702	2731	2787	2835	
99	FAHFA(18:1-O-16:0)	OAHPA	607.6	611.6	280.3	**8**	2383	2529	2585	2619	2647	2705	2787	2836			
100	FAHFA(18:2-O-16:0)	LAHPA	605.6	609.6	280.3	**9**	2384	2424	2448	2472	2501	2531	2593	2655	2707		
101	FAHFA(18:3-O-16:0)	ALAHPA	603.6	607.6	280.3	**2**	2263	2584									
102	FAHFA(18:4-O-16:0)	SDAHPA	601.6	605.6	280.3	**1**	2593										
103	FAHFA(20:0-O-16:0)	AAHPA	637.6	641.6	280.3	**1**	2858										
104	FAHFA(20:1-O-16:0)	EAHPA	635.6	639.6	280.3	**1**	2693										
105	FAHFA(20:2-O-16:0)	EDAHPA	633.6	637.6	280.3	**1**	2556										

106	FAHFA(20:3-O-16:0)	ETAHPA	631.6	635.6	280.3	**1**	2438								
107	FAHFA(22:0-O-16:0)	BAHPA	665.6	669.6	280.3	**1**	3002								
108	FAHFA(22:2-O-16:0)	DDAHPA	661.5	665.5	280.3	**1**	2716								
109	FAHFA(18:1-O-17:0)	OAHHDA	621.6	625.6	294.3	**2**	2080	2133							
110	FAHFA(22:0-O-17:0)	BAHHDA	679.6	683.6	294.3	**3**	2679	2701	2725						
111	FAHFA(14:0-O-18:0)	MAHSA	581.8	585.8	308.3	**2**	2586	2613							
112	FAHFA(16:1-O-18:0)	POHSA	607.8	611.8	308.3	**4**	2540	2593	2794	2838					
113	FAHFA(16:0-O-18:0)	PAHSA	609.8	613.8	308.3	**9**	2575	2615	2678	2761	2790	2844	2870	2949	2991
114	FAHFA(18:0-O-18:0)	SAHSA	637.8	641.8	308.3	**4**	2826	2869	2921	2945					
115	FAHFA(18:1-O-18:0)	OAHSA	635.8	639.8	308.3	**6**	2618	2679	2708	2760	2789	2950			
116	FAHFA(18:2-O-18:0)	LAHSA	633.8	637.8	308.3	**8**	2443	2474	2543	2575	2624	2652	2822	2870	
117	FAHFA(18:3-O-18:0)	ALAHSA	631.8	635.8	308.3	**3**	2429	2449	2500						
118	FAHFA(22:1-O-18:0)	DEAHSA	691.8	695.8	308.3	**1**	2985								
119	FAHFA(14:0-O-19:0)	MAHNDA	595.8	599.8	322.3	**4**	2037	2059	2104	2145					
120	FAHFA(15:1-O-19:0)	PDEAHNDA	607.8	611.8	322.3	**2**	2476	2717							
121	FAHFA(16:1-O-19:0)	POHNDA	621.8	625.8	322.3	**5**	2061	2075	2123	2148	2395				
122	FAHFA(16:0-O-19:0)	PAHNDA	623.8	627.8	322.3	**7**	2145	2228	2248	2301	2331	2354	2394		
123	FAHFA(18:0-O-19:0)	SAHNDA	651.8	655.8	322.3	**7**	2260	2314	2363	2414	2434	2490	2540		
124	FAHFA(18:1-O-19:0)	OAHNDA	649.8	653.8	322.3	**8**	2086	2108	2147	2241	2260	2313	2363	2407	
125	FAHFA(18:2-O-19:0)	LAHNDA	647.8	651.8	322.3	**5**	2088	2107	2146	2219	2409				
126	FAHFA(20:0-O-19:0)	AAHNDA	679.8	683.8	322.3	**6**	2534	2590	2609	2660	2711	2750			
127	FAHFA(22:0-O-19:0)	BAHNDA	707.8	711.8	322.3	**7**	2455	2704	2754	2769	2820	2872	2901		
128	FAHFA(22:1-O-19:0)	DEAHNDA	705.8	709.8	322.3	**1**	2317								
129	FAHFA(22:6-O-19:0)	DHAHNDA	695.8	699.8	322.3	**1**	2134								
130	FAHFA(16:1-O-20:0)	POHAA	635.8	639.8	336.3	**1**	2952								
131	FAHFA(18:2-O-20:0)	LAHAA	661.8	665.8	336.3	**6**	1908	1960	2707	2748	2976	3018			
132	FAHFA(18:1-O-21:0)	OAHHEA	677.8	681.8	350.3	**4**	2053	2098	2345	3191					
133	FAHFA(18:2-O-21:0)	LAHHEA	675.8	679.8	350.3	**1**	3086								

t , retention index

Coptis chinensis

NO.	Normal name	FAHFA ID	*m/z* (DMED labeling)	*m/z* (d_4-DMED labeling)	Product ion	Total number	*t1*	*t2*	*t3*	*t4*	*t5*	*t6*	*t7*	*t8*	*t9*	*t10*
1	FAHFA(17:0-O-14:2)	HDAHTDA	563.5	567.5	248.2	**1**	2086									
2	FAHFA(18:1-O-14:2)	OAHTDA	575.5	579.5	248.2	**1**	1991									
3	FAHFA(20:0-O-14:2)	AAHTDA	605.5	609.5	248.2	**1**	2563									
4	FAHFA(17:0-O-14:3)	HDAHTTA	561.5	565.5	246.2	**2**	2084	2166								
5	FAHFA(16:1-O-16:1)	POHPO	577.5	581.5	278.2	**2**	2292	2333								
6	FAHFA(16:0-O-16:1)	PAHPO	579.5	583.5	278.2	**2**	2475	2513								
7	FAHFA(18:1-O-16:1)	OAHPO	605.5	609.5	278.2	**2**	2479	2517								
8	FAHFA(18:2-O-16:1)	LAHPO	603.5	607.5	278.2	**8**	2282	2327	2364	2489	2543	2570	2604	2629		
9	FAHFA(20:4-O-16:1)	ARAHPO	627.5	631.5	278.2	**1**	2257									
10	FAHFA(16:1-O-18:1)	POHOA	605.5	609.5	306.2	**6**	2402	2433	2473	2494	2517	2770				
11	FAHFA(16:0-O-18:1)	PAHOA	607.5	611.5	306.2	**9**	2566	2605	2641	2664	2683	2714	2796	2846	2928	
12	FAHFA(18:1-O-18:1)	OAHOA	633.5	637.5	306.2	**6**	2434	2568	2608	2642	2684	2716				
13	FAHFA(18:2-O-18:1)	LAHOA	631.5	635.5	306.2	**4**	2435	2497	2548	2801						
14	FAHFA(22:1-O-18:1)	DEAHOA	689.5	693.5	306.2	**1**	2892									
15	FAHFA(14:0-O-18:2)	MAHLA	577.5	581.5	304.2	**1**	2372									
16	FAHFA(16:1-O-18:2)	POHLA	603.5	607.5	304.2	**4**	2347	2388	2436	2552						
17	FAHFA(16:0-O-18:2)	PAHLA	605.5	609.5	304.2	**3**	2563	2609	2716							
18	FAHFA(17:1-O-18:2)	HDEAHLA	617.5	621.5	304.2	**2**	2480	2519								
19	FAHFA(18:0-O-18:2)	SAHLA	633.5	637.5	304.2	**2**	2738	2781								
20	FAHFA(18:1-O-18:2)	OAHLA	631.5	635.5	304.2	**3**	2421	2566	2610							
21	FAHFA(18:2-O-18:2)	LAHLA	629.5	633.5	304.2	**2**	2422	2468								
22	FAHFA(22:0-O-18:2)	BAHLA	689.5	693.5	304.2	**1**	3051									
23	FAHFA(22:1-O-18:2)	DEAHLA	687.5	691.5	304.2	**1**	2792									
24	FAHFA(22:2-O-18:2)	DDAHLA	685.5	689.5	304.2	**1**	2275									
25	FAHFA(16:1-O-18:3)	POHALA	601.5	605.5	302.2	**3**	2239	2277	2306							
26	FAHFA(16:0-O-18:3)	PAHALA	603.5	607.5	302.2	**3**	2412	2451	2479							
27	FAHFA(18:1-O-18:3)	OAHALA	629.5	633.5	302.2	**4**	2274	2314	2420	2458						
28	FAHFA(18:2-O-18:3)	LAHALA	627.5	631.5	302.2	**3**	2272	2312	2339							
29	FAHFA(15:0-O-22:6)	PDAHDHA	639.5	643.5	352.2	**1**	2717									
30	FAHFA(15:1-O-22:6)	PDEAHDHA	637.5	641.5	352.2	**1**	2613									
31	FAHFA(17:1-O-22:6)	HDEAHDHA	665.5	669.5	352.2	**1**	2788									
32	FAHFA(14:0-O-12:0)	MAHDA	497.5	501.5	224.2	**1**	2217									
33	FAHFA(16:0-O-12:0)	PAHDA	525.5	529.5	224.2	**3**	2214	2422	2483							
34	FAHFA(17:0-O-12:0)	HDAHDA	539.5	543.5	224.2	**1**	2386									
35	FAHFA(17:1-O-12:0)	HDEAHDA	537.5	541.5	224.2	**1**	2368									
36	FAHFA(18:0-O-12:0)	SAHDA	553.5	557.5	224.2	**2**	2617	2670								
37	FAHFA(18:2-O-12:0)	LAHDA	549.5	553.5	224.2	**3**	2081	2279	2339							
38	FAHFA(14:0-O-14:0)	MAHMA	525.5	529.5	252.2	**1**	2407									
39	FAHFA(14:1-O-14:0)	MOHMA	523.5	527.5	252.2	**2**	2239	2377								
40	FAHFA(15:1-O-14:0)	PDEAHMA	537.5	541.5	252.2	**1**	2401									
41	FAHFA(16:1-O-14:0)	POHMA	551.5	555.5	252.2	**1**	2458									
42	FAHFA(16:0-O-14:0)	PAHMA	553.5	557.5	252.2	**2**	2603	2659								
43	FAHFA(18:0-O-14:0)	SAHMA	581.5	585.5	252.2	**1**	2781									
44	FAHFA(18:1-O-14:0)	OAHMA	579.5	583.5	252.2	**2**	2610	2666								
45	FAHFA(18:2-O-14:0)	LAHMA	577.5	581.5	252.2	**2**	2461	2522								
46	FAHFA(18:3-O-14:0)	ALAHMA	575.5	579.5	252.2	**1**	2331									
47	FAHFA(20:0-O-14:0)	AAHMA	609.5	613.5	252.2	**1**	2567									
48	FAHFA(15:0-O-15:0)	PDAHPDA	553.5	557.5	266.2	**1**	2548									
49	FAHFA(15:1-O-15:0)	PDEAHPDA	551.5	555.5	266.2	**1**	2438									
50	FAHFA(16:0-O-15:0)	PAHPDA	567.5	571.5	266.2	**2**	2663	2721								
51	FAHFA(18:0-O-15:0)	SAHPDA	595.5	599.5	266.2	**1**	2888									
52	FAHFA(18:1-O-15:0)	OAHPDA	593.5	597.5	266.2	**2**	2668	2726								
53	FAHFA(18:2-O-15:0)	LAHPDA	591.5	595.5	266.2	**3**	2530	2588	2614							
54	FAHFA(18:3-O-15:0)	ALAHPDA	589.5	593.5	266.2	**1**	2459									
55	FAHFA(15:0-O-16:0)	PDAHPA	567.6	571.6	280.3	**1**	2664									
56	FAHFA(16:1-O-16:0)	POHPA	579.6	583.6	280.3	**4**	2347	2388	2432	2666						
57	FAHFA(16:0-O-16:0)	PAHPA	581.6	585.6	280.3	**6**	2387	2516	2563	2613	2781	2833				
58	FAHFA(17:0-O-16:0)	HDAHPA	595.5	599.5	280.3	**1**	1974									
59	FAHFA(18:0-O-16:0)	SAHPA	609.6	613.6	280.3	**1**	2738									
60	FAHFA(18:1-O-16:0)	OAHPA	607.6	611.6	280.3	**10**	2418	2521	2565	2613	2642	2669	2704	2732	2788	2837
61	FAHFA(18:2-O-16:0)	LAHPA	605.6	609.5	280.3	**5**	2381	2419	2649	2676	2707					
62	FAHFA(18:3-O-16:0)	ALAHPA	603.6	607.6	280.3	**2**	2258	2287								
63	FAHFA(20:1-O-16:0)	EAHPA	635.6	639.6	280.3	**1**	2735									
64	FAHFA(20:3-O-16:0)	ETAHPA	631.6	635.6	280.3	**2**	2351	2456								
65	FAHFA(20:4-O-16:0)	ARAHPA	629.6	633.6	280.3	**1**	2351									
66	FAHFA(20:5-O-16:0)	EPAHPA	627.6	631.6	208.3	**2**	2229	2357								
67	FAHFA(15:0-O-17:0)	PDAHHDA	581.6	585.6	294.3	**1**	2728									
68	FAHFA(15:1-O-17:0)	PDEAHHDA	579.6	583.6	294.3	**1**	2626									
69	FAHFA(16:0-O-17:0)	PAHHDA	581.6	585.6	294.3	**1**	2727									
70	FAHFA(18:2-O-17:0)	LAHHDA	619.6	623.6	294.3	**3**	2710	2757	2787							
71	FAHFA(20:0-O-17:0)	AAHHDA	651.6	655.6	294.3	**1**	2218									
72	FAHFA(14:0-O-18:0)	MAHSA	581.8	585.8	308.3	**2**	2578	2784								
73	FAHFA(15:0-O-18:0)	PDAHSA	595.8	599.8	308.3	**1**	2188									
74	FAHFA(16:1-O-18:0)	POHSA	607.8	611.8	308.3	**5**	2526	2565	2592	2618	2793					
75	FAHFA(16:0-O-18:0)	PAHSA	609.8	613.8	308.3	**5**	2676	2733	2759	2782	2949					
76	FAHFA(17:0-O-18:0)	HDAHSA	623.8	627.8	308.3	**2**	2333	2362								
77	FAHFA(18:0-O-18:0)	SAHSA	637.8	641.8	308.3	**1**	2308									
78	FAHFA(18:1-O-18:0)	OAHSA	635.8	639.8	308.3	**4**	2680	2757	2786	2951						
79	FAHFA(18:2-O-18:0)	LAHSA	633.8	637.8	308.3	**3**	2543	2617	2825							
80	FAHFA(18:3-O-18:0)	ALAHSA	631.8	635.8	308.3	**3**	2424	2493	2706							
81	FAHFA(20:5-O-18:0)	EPAHSA	655.8	659.8	308.3	**1**	2932									
82	FAHFA(22:6-O-18:0)	DHAHSA	681.8	685.8	308.3	**4**	2203	2230	2565	2931						
83	FAHFA(16:1-O-19:0)	POHNDA	621.8	625.8	322.3	**4**	2052	2070	2117	2159						
84	FAHFA(17:0-O-19:0)	HDAHNDA	637.8	641.8	322.3	**1**	1862									
85	FAHFA(18:0-O-19:0)	SAHNDA	651.8	655.8	322.3	**2**	2481	2534								
86	FAHFA(18:1-O-19:0)	OAHNDA	649.8	653.8	322.3	**4**	2227	2245	2303	2354						
87	FAHFA(18:2-O-19:0)	LAHNDA	647.8	651.8	322.3	**4**	2083	2105	2150	2206						
88	FAHFA(18:3-O-19:0)	ALAHNDA	645.8	649.8	322.3	**5**	1979	1993	2040	2079	2112					
89	FAHFA(16:1-O-20:0)	POHAA	635.8	639.8	336.3	**1**	2678									
90	FAHFA(16:0-O-20:0)	PAHAA	637.8	641.8	336.3	**1**	2828									
91	FAHFA(18:1-O-20:0)	OAHAA	663.8	667.8	336.3	**1**	2831									
92	FAHFA(18:2-O-20:0)	LAHAA	661.8	665.8	336.3	**3**	2681	2707	3025							
93	FAHFA(18:3-O-20:0)	ALAHAA	659.8	663.8	336.3	**2**	2565	2592								
94	FAHFA(16:1-O-21:0)	POHHEA	649.8	653.8	350.3	**4**	1965	2734	2762	3068						
95	FAHFA(18:1-O-21:0)	OAHHEA	677.8	681.8	350.3	**1**	3201									
96	FAHFA(18:2-O-21:0)	LAHHEA	675.8	679.8	350.3	**1**	3093									
97	FAHFA(18:3-O-21:0)	ALAHHEA	673.8	677.8	350.3	**1**	2994									

t, retention index

Tomato (*Lycopersicon esculentum*)

NO.	Normal name	FAHFA ID	*m/z* (DMED labeling)	*m/z* (d_4-DMED labeling)	Product ion	Total number	*t1*	*t2*	*t3*	*t4*	*t5*	*t6*	*t7*	*t8*	*t9*	*t10*	*t11*
1	FAHFA(14:0-O-16:1)	MAHPO	551.5	555.5	278.2	**2**	2418	2451									
2	FAHFA(16:1-O-16:1)	POHPO	577.5	581.5	278.2	**2**	2264	2290									
3	FAHFA(17:0-O-16:1)	HDAHPO	593.5	597.5	278.2	**1**	2244										
4	FAHFA(16:1-O-16:2)	POHHDA	575.5	579.5	276.2	**1**	2208										
5	FAHFA(16:0-O-16:3)	PAHHTA	575.5	579.5	274.2	**1**	2133										
6	FAHFA(16:0-O-18:1)	PAHOA	607.5	611.5	306.2	**1**	2612										
7	FAHFA(18:0-O-18:1)	SAHOA	635.5	639.5	306.2	**2**	2613	2788									
8	FAHFA(18:1-O-18:1)	OAHOA	633.5	637.5	306.2	**3**	2468	2614	2646								
9	FAHFA(18:2-O-18:1)	LAHOA	631.5	635.5	306.2	**2**	2466	2495									
10	FAHFA(20:0-O-18:1)	AAHOA	663.5	667.5	306.2	**1**	2945										
11	FAHFA(20:1-O-18:1)	EAHOA	661.5	665.5	306.2	**1**	2781										
12	FAHFA(22:6-O-18:1)	DHAHOA	679.5	683.5	306.2	**1**	2251										
13	FAHFA(14:0-O-18:2)	MAHLA	577.5	581.5	304.2	**2**	2372	2517									
14	FAHFA(14:1-O-18:2)	MOHLA	575.5	579.5	304.2	**1**	2361										
15	FAHFA(15:0-O-18:2)	PDAHLA	591.5	595.5	304.2	**1**	2577										
16	FAHFA(16:0-O-18:2)	PAHLA	605.5	609.5	304.2	**3**	2566	2615	2711								
17	FAHFA(18:0-O-18:2)	SAHLA	633.5	637.5	304.2	**2**	2743	2784									
18	FAHFA(18:2-O-18:2)	LAHLA	629.5	633.5	304.2	**2**	2328	2421									
19	FAHFA(14:0-O-18:3)	MAHALA	575.5	579.5	302.2	**1**	2255										
20	FAHFA(16:0-O-18:3)	PAHALA	603.5	607.5	302.2	**1**	2471										
21	FAHFA(18:2-O-18:3)	LAHALA	627.5	631.5	302.2	**2**	2248	2307									
22	FAHFA(14:0-O-12:0)	MAHDA	497.5	501.5	224.2	**2**	1986	2016									
23	FAHFA(15:0-O-12:0)	PDAHDA	511.5	515.5	224.2	**2**	2073	2105									
24	FAHFA(16:1-O-12:0)	POHDA	523.5	527.5	224.2	**1**	2044										
25	FAHFA(16:0-O-12:0)	PAHDA	525.5	529.5	224.2	**3**	2200	2406	2463								
26	FAHFA(18:0-O-12:0)	SAHDA	553.5	557.5	224.2	**3**	2394	2600	2659								
27	FAHFA(14:0-O-14:0)	MAHMA	525.5	529.5	252.2	**2**	2158	2404									
28	FAHFA(16:1-O-14:0)	POHMA	551.5	555.5	252.2	**2**	2187	2380									
29	FAHFA(16:0-O-14:0)	PAHMA	553.5	557.5	252.2	**3**	2353	2601	2659								
30	FAHFA(18:0-O-14:0)	SAHMA	581.5	585.5	252.2	**3**	2547	2787	2834								
31	FAHFA(16:0-O-15:0)	PAHPDA	567.5	571.5	266.2	**3**	2437	2699	2750								
32	FAHFA(18:0-O-15:0)	SAHPDA	595.5	599.5	266.2	**3**	2624	2874	2917								
33	FAHFA(16:1-O-16:0)	POHPA	579.6	583.6	280.3	**1**	2346										
34	FAHFA(16:0-O-16:0)	PAHPA	581.6	585.6	280.3	**11**	2513	2537	2565	2587	2614	2642	2672	2702	2735	2787	2839
35	FAHFA(18:0-O-16:0)	SAHPA	609.6	613.6	280.3	**11**	2692	2717	2739	2767	2790	2815	2843	2872	2901	2947	2995
36	FAHFA(18:2-O-16:0)	LAHPA	605.6	609.6	280.3	**1**	2379										
37	FAHFA(18:3-O-16:0)	ALAHPA	603.6	607.6	280.3	**2**	2254	2580									
38	FAHFA(22:6-O-16:0)	DHAHPA	653.6	657.6	280.3	**2**	2792	2829									
39	FAHFA(16:1-O-18:0)	POHSA	607.8	611.8	308.3	**1**	2513										
40	FAHFA(16:0-O-18:0)	PAHSA	609.8	613.8	308.3	**10**	2669	2699	2767	2792	2818	2847	2876	2911	2951	3000	
41	FAHFA(18:0-O-18:0)	SAHSA	637.8	641.8	308.3	**9**	2834	2867	2927	2948	3003	3028	3057	3100	3140		
42	FAHFA(22:6-O-18:0)	DHAHSA	681.8	685.8	308.3	**1**	2424										
43	FAHFA(18:2-O-19:0)	LAHNDA	647.8	651.8	322.3	**2**	2156	2208									
44	FAHFA(16:0-O-20:0)	PAHAA	637.8	641.8	336.3	**2**	2825	3140									
45	FAHFA(18:0-O-20:0)	SAHAA	665.8	669.8	336.3	**2**	2980	3268									
46	FAHFA(18:1-O-20:0)	OAHAA	663.8	667.8	336.3	**1**	2778										
47	FAHFA(20:1-O-20:0)	EAHAA	691.8	695.8	336.3	**1**	2933										
48	FAHFA(16:0-O-21:0)	PAHHEA	651.8	655.8	350.3	**1**	3203										
49	FAHFA(18:0-O-21:0)	SAHHEA	679.8	683.8	350.3	**1**	3328										

t , retention index

Apple (*Malus pumila*)

NO.	Normal name	FAHFA ID	*m/z* (DMED labeling)	*m/z* (d_4-DMED labeling)	Product ion	Total number	*t1*	*t2*	*t3*	*t4*	*t5*	*t6*	*t7*	*t8*	*t9*	*t10*	*t11*
1	FAHFA(16:1-O-16:1)	POHPO	577.5	581.5	278.2	**2**	2263	2293									
2	FAHFA(18:0-O-16:1)	SAHPO	607.5	611.5	278.2	**1**	2116										
3	FAHFA(20:5-O-16:2)	EPAHHDA	623.5	627.5	276.2	**4**	1987	2012	2035	2074							
4	FAHFA(20:5-O-16:3)	EPAHHTA	621.5	625.5	274.2	**2**	2027	2053									
5	FAHFA(14:1-O-18:1)	MOHOA	577.5	581.5	306.2	**1**	2232										
6	FAHFA(16:0-O-18:1)	PAHOA	607.5	611.5	306.2	**5**	2574	2614	2697	2814	2855						
7	FAHFA(18:0-O-18:1)	SAHOA	635.5	639.5	306.2	**1**	2788										
8	FAHFA(18:1-O-18:1)	OAHOA	633.5	637.5	306.2	**5**	2470	2576	2615	2649	2697						
9	FAHFA(18:2-O-18:1)	LAHOA	631.5	635.5	306.2	**3**	2440	2470	2497								
10	FAHFA(20:1-O-18:1)	EAHOA	661.5	665.5	306.2	**1**	2784										
11	FAHFA(22:2-O-18:1)	DDAHOA	687.5	691.5	306.2	**1**	2666										
12	FAHFA(22:6-O-18:1)	DHAHOA	679.5	683.5	306.2	**1**	2246										
13	FAHFA(16:0-O-18:2)	PAHLA	605.5	609.5	304.2	**5**	2468	2570	2613	2683	2719						
14	FAHFA(18:0-O-18:2)	SAHLA	633.5	637.5	304.2	**2**	2750	2786									
15	FAHFA(18:1-O-18:2)	OAHLA	631.5	635.5	304.2	**4**	2478	2530	2570	2616							
16	FAHFA(18:2-O-18:2)	LAHLA	629.5	633.5	304.2	**4**	2328	2377	2424	2468							
17	FAHFA(18:2-O-18:3)	LAHALA	627.5	631.5	302.2	**1**	2248										
18	FAHFA(14:0-O-12:0)	MAHDA	497.5	501.5	224.2	**2**	1988	2020									
19	FAHFA(15:0-O-12:0)	PDAHDA	511.5	515.5	224.2	**2**	2077	2109									
20	FAHFA(16:1-O-12:0)	POHDA	523.5	527.5	224.2	**2**	2026	2048									
21	FAHFA(16:0-O-12:0)	PAHDA	525.5	529.5	224.2	**3**	2205	2413	2468								
22	FAHFA(18:0-O-12:0)	SAHDA	553.5	557.5	224.2	**3**	2399	2603	2661								
23	FAHFA(14:0-O-14:0)	MAHMA	525.5	529.5	252.2	**3**	2137	2158	2405								
24	FAHFA(15:0-O-14:0)	PDAHMA	539.5	543.5	252.2	**3**	2223	2261	2313								
25	FAHFA(16:1-O-14:0)	POHMA	551.5	555.5	252.2	**1**	2193										
26	FAHFA(16:0-O-14:0)	PAHMA	553.5	557.5	252.2	**7**	2354	2437	2488	2522	2552	2601	2664				
27	FAHFA(18:0-O-14:0)	SAHMA	581.5	585.5	252.2	**3**	2546	2783	2840								
28	FAHFA(15:1-O-16:0)	PDEAHPA	565.6	569.6	280.3	**1**	2781										
29	FAHFA(16:0-O-16:0)	PAHPA	581.6	585.6	280.3	**10**	2514	2539	2573	2615	2647	2676	2704	2738	2788	2844	
30	FAHFA(17:1-O-16:0)	HDEAHPA	593.6	597.6	280.3	**1**	2944										
31	FAHFA(18:0-O-16:0)	SAHPA	609.6	613.6	280.3	**11**	2694	2719	2746	2773	2794	2824	2848	2876	2908	2952	3000
32	FAHFA(18:1-O-16:0)	OAHPA	607.6	613.6	280.3	**2**	2425	2528									
33	FAHFA(18:2-O-16:0)	LAHPA	605.6	609.6	280.3	**1**	2376										
34	FAHFA(20:3-O-16:0)	ETAHPA	663.6	667.6	280.3	**1**	2782										
35	FAHFA(18:0-O-17:0)	SAHHDA	623.6	627.6	294.3	**3**	2713	2767	2877								
36	FAHFA(18:1-O-17:0)	OAHHDA	621.6	625.6	294.3	**1**	2608										
37	FAHFA(18:2-O-17:0)	LAHHDA	619.6	623.6	294.3	**1**	2463										
38	FAHFA(15:1-O-18:0)	PDEAHSA	593.8	597.8	308.3	**1**	2947										
39	FAHFA(16:0-O-18:0)	PAHSA	609.8	613.8	308.3	**11**	2672	2697	2740	2768	2790	2821	2849	2878	2911	2957	3003
40	FAHFA(17:1-O-18:0)	HDEAHSA	621.8	625.8	308.3	**1**	3099										
41	FAHFA(18:0-O-18:0)	SAHSA	637.8	641.8	308.3	**5**	2837	2871	2928	2954	3146						
42	FAHFA(20:1-O-18:0)	EAHSA	663.8	667.8	308.3	**1**	2781										
43	FAHFA(20:2-O-18:0)	EDAHSA	661.8	665.8	308.3	**1**	2900										
44	FAHFA(22:1-O-18:0)	DEAHSA	691.8	695.8	308.3	**1**	2936										
45	FAHFA(22:2-O-18:0)	DDAHSA	689.8	693.8	308.3	**1**	2795										
46	FAHFA(18:2-O-19:0)	LAHNDA	647.8	651.8	322.3	**4**	2087	2106	2153	2207							
47	FAHFA(22:0-O-19:0)	BAHNDA	707.8	711.8	322.3	**1**	2450										
48	FAHFA(22:1-O-19:0)	DEAHNDA	705.8	709.8	322.3	**1**	2304										
49	FAHFA(14:1-O-20:0)	MOHAA	607.8	611.8	336.3	**1**	2427										
50	FAHFA(16:1-O-20:0)	POHAA	635.8	639.8	336.3	**1**	2611										
51	FAHFA(18:1-O-20:0)	OAHAA	663.8	667.8	336.3	**1**	2781										
52	FAHFA(20:1-O-20:0)	EAHAA	691.8	695.8	336.3	**1**	2935										
53	FAHFA(20:2-O-20:0)	EDAHAA	689.8	693.8	336.3	**1**	2794										
54	FAHFA(20:3-O-20:0)	ETAHAA	687.5	691.8	336.3	**1**	2665										
55	FAHFA(22:1-O-20:0)	DEAHAA	719.8	723.8	336.3	**1**	3077										
56	FAHFA(16:0-O-21:0)	PAHHEA	651.8	655.8	350.3	**3**	2914	3132	3216								

t , retention index

Peanut (*Arachis hypogaea*)

NO.	Normal name	FAHFA ID	*m/z* (DMED labeling)	*m/z* (d_4-DMED labeling)	Product ion	Total number	*t1*	*t2*	*t3*	*t4*
1	FAHFA(16:1-O-18:1)	POHOA	605.5	609.5	306.2	**1**	2404			
2	FAHFA(18:1-O-18:1)	OAHOA	633.5	637.5	306.2	**1**	2614			
3	FAHFA(18:2-O-18:1)	LAHOA	631.5	635.5	306.2	**1**	2467			
4	FAHFA(16:0-O-18:2)	PAHLA	605.5	609.5	304.2	**3**	2466	2568	2614	
5	FAHFA(18:1-O-18:2)	OAHLA	631.5	635.5	304.2	**2**	2423	2572		
6	FAHFA(18:2-O-18:2)	LAHLA	629.5	633.5	304.2	**2**	2325	2423		
7	FAHFA(18:2-O-18:3)	LAHALA	627.5	631.5	302.2	**2**	2273	2314		
8	FAHFA(16:0-O-16:0)	PAHPA	581.6	585.6	280.3	**1**	2512			
9	FAHFA(18:2-O-16:0)	LAHPA	605.6	609.6	280.3	**1**	2362			
10	FAHFA(16:1-O-18:0)	POHSA	607.5	611.8	308.3	**1**	2545			
11	FAHFA(18:1-O-18:0)	OAHSA	635.8	639.8	308.3	**1**	2760			
12	FAHFA(18:2-O-18:0)	LAHSA	633.8	637.8	308.3	**1**	2624			
13	FAHFA(18:3-O-18:0)	ALAHSA	631.8	635.8	308.3	**1**	2456			
14	FAHFA(16:0-O-19:0)	PAHNDA	623.8	627.8	322.3	**4**	2209	2229	2286	2336
15	FAHFA(18:1-O-19:0)	OAHNDA	649.8	653.8	322.3	**4**	2222	2240	2297	2346
16	FAHFA(18:2-O-19:0)	LAHNDA	647.8	651.8	322.3	**4**	2077	2094	2135	2196
17	FAHFA(16:1-O-20:0)	POHAA	635.8	639.8	336.3	**1**	2813			
18	FAHFA(16:0-O-20:0)	PAHAA	637.8	641.8	336.3	**1**	2928			

t , retention index

Black Sesame (*Sesamum indicum*)

NO.	Normal name	FAHFA ID	*m/z* (DMED labeling)	*m/z* (d_4-DMED labeling)	Product ion	Total number	*t1*	*t2*	*t3*	*t4*	*t5*	*t6*	*t7*	*t8*	*t9*
1	FAHFA(18:2-O-16:1)	LAHPO	603.5	607.5	278.2	**1**	2438								
2	FAHFA(18:1-O-18:1)	OAHOA	633.5	637.5	306.2	**4**	2606	2642	2682	2718					
3	FAHFA(18:2-O-18:1)	LAHOA	631.5	635.5	306.2	**4**	2438	2467	2501	2560					
4	FAHFA(16:0-O-18:2)	PAHLA	605.5	609.5	304.2	**2**	2565	2608							
5	FAHFA(18:2-O-18:2)	LAHLA	629.5	633.5	304.2	**2**	2432	2467							
6	FAHFA(18:2-O-20:2)	LAHEDA	657.5	661.5	332.2	**2**	2034	2060							
7	FAHFA(22:0-O-20:4)	BAHARA	713.5	717.5	328.2	**1**	1976								
8	FAHFA(18:1-O-14:0)	OAHMA	579.5	583.5	252.2	**1**	1882								
9	FAHFA(18:2-O-14:0)	LAHMA	577.5	581.5	252.2	**1**	2468								
10	FAHFA(18:1-O-16:0)	OAHPA	607.6	611.6	280.3	**2**	2736	2786							
11	FAHFA(18:1-O-17:0)	OAHHDA	621.6	625.6	294.3	**1**	2373								
12	FAHFA(18:2-O-17:0)	LAHHDA	619.6	623.6	294.3	**1**	2218								
13	FAHFA(18:2-O-18:0)	LAHSA	633.8	637.8	308.3	**1**	2621								
14	FAHFA(16:0-O-19:0)	PAHNDA	623.8	627.8	322.3	**3**	2212	2290	2341						
15	FAHFA(18:1-O-19:0)	OAHNDA	649.8	653.8	322.3	**9**	2083	2104	2154	2201	2226	2245	2301	2354	2450
16	FAHFA(18:2-O-19:0)	LAHNDA	647.8	651.5	322.3	**5**	2084	2102	2154	2201	2299				

t , retention index

Wheat (*Triticum aestivum*)

NO.	Normal name	FAHFA ID	*m/z* (DMED labeling)	*m/z* (d_4-DMED labeling)	Product ion	Total number	*t1*	*t2*	*t3*	*t4*	*t5*	*t6*	*t7*	*t8*	*t9*	*t10*	*t11*	*t12*	*t13*	*t14*
1	FAHFA(18:2-O-14:1)	LAHMO	575.5	579.5	250.2	**3**	2142	2217	2269											
2	FAHFA(20:0-O-14:1)	AAHMO	607.5	611.5	250.2	**1**	2574													
3	FAHFA(20:1-O-14:1)	EAHMO	605.5	609.5	250.2	**1**	2564													
4	FAHFA(22:2-O-14:1)	DDAHMO	631.5	635.5	250.2	**2**	2439	2568												
5	FAHFA(18:2-O-14:2)	LAHTDA	573.5	577.5	248.2	**1**	2200													
6	FAHFA(20:0-O-14:2)	AAHTDA	605.5	609.5	248.2	**2**	2563	2617												
7	FAHFA(22:1-O-14:2)	DEAHTDA	631.5	635.5	248.2	**3**	2421	2566	2620											
8	FAHFA(22:2-O-14:2)	DDAHTDA	629.5	633.5	248.2	**4**	2334	2383	2420	2472										
9	FAHFA(22:6-O-14:2)	DHAHTDA	621.5	625.5	248.2	**1**	2122													
10	FAHFA(22:1-O-14:3)	DEAHTTA	629.5	633.5	246.2	**2**	2420	2470												
11	FAHFA(22:2-O-14:3)	DDAHTTA	627.5	631.5	246.2	**2**	2279	2320												
12	FAHFA(16:0-O-16:1)	PAHPO	579.5	583.5	246.2	**4**	1860	2537	2650	2745										
13	FAHFA(18:1-O-16:1)	OAHPO	605.5	609.5	246.2	**3**	1886	2534	2647											
14	FAHFA(18:2-O-16:1)	LAHPO	603.5	607.5	246.2	**5**	2280	2379	2404	2505	2578									
15	FAHFA(16:0-O-16:2)	PAHHDA	577.5	581.5	276.2	**2**	2393	2449												
16	FAHFA(18:0-O-16:2)	SAHHDA	605.6	609.6	276.2	**2**	2565	2618												
17	FAHFA(18:1-O-16:2)	OAHHDA	603.6	607.6	276.2	**2**	2402	2460												
18	FAHFA(18:2-O-16:2)	LAHHDA	601.5	605.5	276.2	**2**	2248	2308												
19	FAHFA(20:1-O-16:2)	EAHHDA	631.5	635.5	276.2	**1**	2571													
20	FAHFA(20:2-O-16:2)	EDAHHDA	629.5	633.5	276.2	**2**	2419	2472												
21	FAHFA(18:0-O-16:3)	SAHHTA	603.5	607.5	274.2	**2**	2413	2459												
22	FAHFA(20:1-O-16:3)	EAHHTA	629.5	633.5	274.2	**2**	2420	2468												
23	FAHFA(20:2-O-16:3)	EDAHHTA	637.5	641.5	274.2	**2**	2279	2318												
24	FAHFA(20:5-O-16:3)	EPAHHTA	621.5	625.5	274.2	**2**	2120	2146												
25	FAHFA(22:6-O-16:3)	DHAHHTA	647.5	651.5	274.2	**1**	2132													
26	FAHFA(14:0-O-18:1)	MAHOA	579.5	583.5	306.2	**2**	2388	2515												
27	FAHFA(16:1-O-18:1)	POHOA	605.5	609.5	306.2	**4**	2413	2525	2564	2619										
28	FAHFA(16:0-O-18:1)	PAHOA	607.5	613.5	306.2	**5**	2572	2618	2651	2686	2706									
29	FAHFA(18:1-O-18:1)	OAHOA	633.5	637.5	306.2	**5**	2580	2618	2652	2686	2706									
30	FAHFA(18:2-O-18:1)	LAHOA	631.5	635.5	306.2	**5**	2418	2440	2468	2507	2554									
31	FAHFA(20:1-O-18:1)	EAHOA	661.5	665.5	306.2	**3**	2745	2766	2862											
32	FAHFA(22:6-O-18:1)	DHAHOA	679.5	983.5	306.2	**6**	2403	2431	2504	2538	2586	2635								
33	FAHFA(14:0-O-18:2)	MAHLA	577.5	581.5	304.2	**3**	2338	2375	2420											
34	FAHFA(15:0-O-18:2)	PDAHLA	591.5	595.5	304.2	**3**	2435	2475	2523											
35	FAHFA(15:1-O-18:2)	PDEAHLA	589.5	593.5	304.2	**2**	2323	2360												
36	FAHFA(16:1-O-18:2)	POHLA	603.5	607.5	304.2	**1**	2387													
37	FAHFA(16:0-O-18:2)	PAHLA	605.5	609.5	304.2	**2**	2564	2618												
38	FAHFA(17:1-O-18:2)	HDEAHLA	617.5	621.5	304.2	**1**	2482													
39	FAHFA(18:1-O-18:2)	OAHLA	631.5	635.5	304.2	**5**	2419	2473	2533	2567	2620									
40	FAHFA(18:2-O-18:2)	LAHLA	629.5	633.5	304.2	**3**	2382	2420	2473											
41	FAHFA(20:0-O-18:2)	AAHLA	661.5	665.5	304.2	**2**	2742	2908												
42	FAHFA(20:1-O-18:2)	EAHLA	659.5	663.5	304.2	**2**	2743	2783												
43	FAHFA(20:2-O-18:2)	EDAHLA	657.5	661.5	304.2	**1**	2591													
44	FAHFA(22:0-O-18:2)	BAHLA	689.5	693.5	304.2	**1**	3054													
45	FAHFA(22:1-O-18:2)	DEAHLA	687.5	691.5	304.2	**2**	2896	2933												
46	FAHFA(22:6-O-18:2)	DHAHLA	677.5	681.5	304.2	**6**	2320	2337	2407	2431	2490	2539								
47	FAHFA(14:0-O-18:3)	MAHALA	575.5	579.5	302.2	**2**	2218	2264												
48	FAHFA(16:0-O-18:3)	PAHALA	603.5	607.5	302.2	**3**	2384	2413	2460											
49	FAHFA(18:1-O-18:3)	OAHALA	629.5	633.5	302.2	**4**	2279	2319	2421	2465										
50	FAHFA(18:2-O-18:3)	LAHALA	627.5	631.5	302.2	**2**	2278	2319												
51	FAHFA(20:1-O-18:3)	EAHALA	657.5	661.5	302.2	**1**	2643													
52	FAHFA(22:6-O-18:3)	DHAHALA	675.5	679.5	302.2	**3**	2207	2314	2340											
53	FAHFA(18:2-O-20:1)	LAHEA	659.5	663.5	334.2	**5**	2676	2705	2751	2788	2826									
54	FAHFA(16:0-O-20:2)	PAHEDA	633.5	637.5	332.2	**3**	2683	2707	2738											
55	FAHFA(18:1-O-20:2)	OAHEDA	659.5	663.5	332.2	**1**	2684													
56	FAHFA(18:2-O-20:2)	LAHEDA	657.5	661.5	332.2	**1**	2548													
57	FAHFA(16:0-O-22:6)	PAHDHA	653.5	657.5	352.2	**3**	2461	2498	2536											
58	FAHFA(18:1-O-22:6)	OAHDHA	679.5	683.5	352.2	**6**	2352	2395	2434	2467	2503	2542								
59	FAHFA(18:3-O-22:6)	ALAHDHA	675.5	679.5	352.2	**2**	2224	2274												
60	FAHFA(16:0-O-12:0)	PAHDA	525.5	529.5	224.2	**2**	2223	2427												
61	FAHFA(18:2-O-12:0)	LAHDA	549.5	553.5	224.2	**5**	2090	2164	2229	2282	2347									
62	FAHFA(22:2-O-12:0)	DDAHDA	605.5	609.5	224.2	**1**	2580													
63	FAHFA(14:0-O-14:0)	MAHMA	525.5	529.5	252.2	**1**	2420													
64	FAHFA(16:0-O-14:0)	PAHMA	553.5	557.5	252.2	**9**	1908	2366	2472	2500	2532	2564	2606	2625	2675					
65	FAHFA(18:0-O-14:0)	SAHMA	581.5	586.5	252.2	**1**	2796													
66	FAHFA(18:1-O-14:0)	OAHMA	579.5	583.5	252.2	**7**	1922	2378	2477	2513	2535	2568	2630							
67	FAHFA(18:2-O-14:0)	LAHMA	577.5	581.5	252.2	**8**	1819	2225	2296	2352	2379	2415	2479	2542						
68	FAHFA(18:3-O-14:0)	ALAHMA	575.5	579.5	252.2	**3**	2220	2284	2346											
69	FAHFA(16:0-O-15:0)	PAHPDA	567.5	571.5	266.2	**5**	2437	2535	2585	2655	2761									
70	FAHFA(18:1-O-15:0)	OAHPDA	593.5	597.5	266.2	**5**	2447	2537	2586	2657	2766									
71	FAHFA(18:2-O-15:0)	LAHPDA	591.5	595.5	266.2	**6**	1989	2300	2436	2474	2511	2629								
72	FAHFA(14:0-O-16:0)	MAHPA	553.6	557.6	280.3	**5**	2337	2377	2403	2459	2489									
73	FAHFA(15:0-O-16:0)	PDAHPA	567.6	571.6	280.3	**3**	2422	2505	2581											
74	FAHFA(15:1-O-16:0)	PDEAHPA	565.6	569.6	280.3	**1**	2430													
75	FAHFA(16:1-O-16:0)	POHPA	579.6	583.6	280.3	**3**	2354	2418	2516											
76	FAHFA(16:0-O-16:0)	PAHPA	581.6	585.6	280.3	**3**	2522	2593	2679											
77	FAHFA(17:0-O-16:0)	HDAHPA	595.6	599.6	280.3	**3**	2133	2684	2765											
78	FAHFA(17:1-O-16:0)	HDEAHPA	593.6	597.6	280.3	**1**	2588													
79	FAHFA(18:1-O-16:0)	OAHPA	607.6	611.6	280.3	**8**	2533	2593	2651	2680	2711	2746	2798	2848						
80	FAHFA(18:2-O-16:0)	LAHPA	605.6	609.6	280.3	**6**	2385	2449	2507	2535	2594	2712								
81	FAHFA(18:3-O-16:0)	ALAHPA	603.6	607.6	280.3	**1**	2399													
82	FAHFA(20:1-O-16:0)	EAHPA	635.6	639.6	280.3	**1**	2842													
83	FAHFA(20:2-O-16:0)	EDAHPA	633.6	637.6	280.3	**1**	2709													
84	FAHFA(20:3-O-16:0)	ETAHPA	663.6	667.6	280.3	**1**	2991													
85	FAHFA(18:0-O-17:0)	SAHHDA	623.6	627.6	294.3	**2**	2216	2236												
86	FAHFA(18:2-O-17:0)	LAHHDA	619.6	623.6	294.3	**8**	1976	2011	2036	2050	2464	2625	2749	2798						
87	FAHFA(20:1-O-17:0)	EAHHDA	649.6	653.6	294.3	**4**	2228	2248	2311	2357										
88	FAHFA(20:2-O-17:0)	EDAHHDA	647.6	651.6	294.3	**4**	2084	2105	2154	2209										
89	FAHFA(16:1-O-18:0)	POHSA	607.8	611.8	308.3	**1**	2560													
90	FAHFA(16:0-O-18:0)	PAHSA	609.8	613.8	308.3	**8**	2145	2208	2255	2575	2677	2706	2763	2795						
91	FAHFA(18:1-O-18:0)	OAHSA	635.8	639.8	308.3	**12**	2155	2582	2675	2705	2763	2792	2822	2849	2876	2907	2955	2999		
92	FAHFA(18:2-O-18:0)	LAHSA	633.8	637.8	308.3	**14**	2031	2040	2077	2125	2441	2552	2624	2654	2679	2707	2742	2775	2828	2878
93	FAHFA(14:0-O-19:0)	MAHNDA	595.8	599.8	322.3	**4**	2042	2057	2098	2148										
94	FAHFA(15:0-O-19:0)	PDAHNDA	609.8	613.8	322.3	**4**	2126	2141	2202	2245										
95	FAHFA(15:1-O-19:0)	PDEAHNDA	607.8	613.8	322.3	**2**	2001	2023												
96	FAHFA(16:1-O-19:0)	POHNDA	621.8	625.8	322.3	**4**	2060	2072	2123	2163										
97	FAHFA(16:0-O-19:0)	PAHNDA	623.8	627.8	322.3	**4**	2216	2235	2298	2347										
98	FAHFA(17:0-O-19:0)	HDAHNDA	637.8	641.8	322.3	**4**	2317	2337	2391	2443										
99	FAHFA(17:1-O-19:0)	HDEAHNDA	653.8	657.8	322.3	**3**	2141	2151	2213											
100	FAHFA(18:1-O-19:0)	OAHNDA	649.8	653.8	322.3	**6**	2082	2104	2225	2250	2321	2356								
101	FAHFA(18:2-O-19:0)	LAHNDA	647.8	651.8	322.3	**4**	2083	2104	2156	2208										
102	FAHFA(18:3-O-19:0)	ALAHNDA	645.8	649.8	322.3	**4**	1983	1996	2048	2078										
103	FAHFA(20:0-O-19:0)	AAHNDA	679.8	683.8	322.3	**6**	2409	2432	2589	2617	2668	2709								
104	FAHFA(20:1-O-19:0)	EAHNDA	677.8	681.8	322.3	**6**	2322	2338	2409	2432	2495	2540								
105	FAHFA(20:2-O-19:0)	EDAHNDA	675.8	679.8	322.3	**6**	2174	2195	2266	2284	2341	2386								
106	FAHFA(22:0-O-19:0)	BAHNDA	707.8	711.8	322.3	**7**	2457	2520	2585	2759	2778	2828	2877							
107	FAHFA(22:1-O-19:0)	DEAHNDA	705.8	709.8	322.3	**6**	2313	2375	2586	2613	2663	2707								
108	FAHFA(22:6-O-19:0)	DHAHNDA	695.8	699.8	322.3	**1**	2389													
109	FAHFA(18:2-O-20:0)	LAHAA	661.8	665.8	336.3	**3**	1920	1950	3024											
110	FAHFA(16:0-O-21:0)	PAHHEA	651.8	655.8	350.3	**1**	2335													
111	FAHFA(18:1-O-21:0)	OAHHEA	677.8	681.8	350.3	**1**	2342													
112	FAHFA(18:2-O-21:0)	LAHHEA	675.8	679.8	350.3	**3**	2201	2245	2290											

t , retention index

Rice (*Oryza sativa*)

NO.	Normal name	FAHFA ID	*m/z* (DMED labeling)	*m/z* (*d* 4-DMED labeling)	Product ion	Total number	*t1*	*t2*	*t3*	*t4*	*t5*	*t6*	*t7*
1	FAHFA(22:1-O-14:2)	DEAHTDA	631.5	635.5	248.2	**1**	2557						
2	FAHFA(22:2-O-14:2)	DDAHTDA	629.5	633.5	248.2	**1**	2411						
3	FAHFA(20:0-O-16:1)	AAHPO	635.5	639.5	278.2	**1**	2256						
4	FAHFA(14:0-O-18:1)	MAHOA	579.5	583.5	306.2	**3**	2360	2395	2485				
5	FAHFA(16:0-O-18:1)	PAHOA	607.5	613.5	306.2	**3**	2547	2586	2671				
6	FAHFA(18:1-O-18:1)	OAHOA	633.5	637.5	306.2	**4**	2550	2586	2621	2672			
7	FAHFA(18:2-O-18:1)	LAHOA	631.5	635.5	306.2	**5**	2411	2438	2476	2527	2539		
8	FAHFA(14:0-O-18:2)	MAHLA	577.5	581.5	304.2	**2**	2341	2394					
9	FAHFA(16:0-O-18:2)	PAHLA	605.5	609.5	304.2	**3**	2494	2536	2582				
10	FAHFA(18:1-O-18:2)	OAHLA	631.5	635.5	304.2	**5**	2393	2439	2498	2539	2585		
11	FAHFA(18:2-O-18:2)	LAHLA	629.5	633.5	304.2	**3**	2350	2393	2438				
12	FAHFA(16:0-O-18:3)	PAHALA	603.5	607.5	302.2	**2**	2383	2422					
13	FAHFA(18:1-O-18:3)	OAHALA	629.5	633.5	302.2	**2**	2391	2429					
14	FAHFA(18:2-O-18:3)	LAHALA	627.5	631.5	302.2	**2**	2243	2282					
15	FAHFA(18:1-O-22:6)	OAHDHA	679.5	683.5	352.2	**2**	2449	2492					
16	FAHFA(14:0-O-14:0)	MAHMA	525.5	529.5	252.2	**1**	2417						
17	FAHFA(14:0-O-16:0)	MAHPA	553.6	557.6	280.3	**1**	2338						
18	FAHFA(16:0-O-16:0)	PAHPA	581.6	585.6	280.3	**2**	2525	2676					
19	FAHFA(18:1-O-16:0)	OAHPA	607.6	611.6	280.3	**2**	2533	2676					
20	FAHFA(18:2-O-16:0)	LAHPA	605.6	609.6	280.3	**7**	2388	2476	2504	2535	2598	2662	2714
21	FAHFA(18:3-O-16:0)	ALAHPA	603.6	607.6	280.3	**2**	2263	2402					
22	FAHFA(20:2-O-17:0)	EDAHHDA	647.6	651.6	294.3	**2**	2163	2210					
23	FAHFA(16:1-O-18:0)	POHSA	607.8	611.8	308.3	**1**	2565						
24	FAHFA(18:2-O-18:0)	LAHSA	633.8	637.8	308.3	**5**	2444	2553	2580	2627	2652		
25	FAHFA(14:0-O-19:0)	MAHNDA	595.8	599.8	322.3	**4**	2023	2044	2098	2146			
26	FAHFA(16:1-O-19:0)	POHNDA	621.8	625.8	322.3	**2**	2119	2165					
27	FAHFA(16:0-O-19:0)	PAHNDA	623.8	627.8	322.3	**5**	2154	2215	2235	2294	2342		
28	FAHFA(18:1-O-19:0)	OAHNDA	649.8	653.8	322.3	**5**	2158	2230	2249	2306	2356		
29	FAHFA(18:2-O-19:0)	LAHNDA	647.8	651.8	322.3	**5**	2028	2084	2103	2157	2207		
30	FAHFA(18:3-O-19:0)	ALAHNDA	645.8	649.8	322.3	**2**	2034	2078					

t, retention index

Egg

NO.	Normal name	FAHFA ID	*m/z* (DMED labeling)	*m/z* (d_4-DMED labeling)	Product ion	Total number	*t1*	*t2*	*t3*	*t4*	*t5*	*t6*	*t7*	*t8*	*t9*	*t10*
1	FAHFA(16:0-O-14:1)	PAHMO	551.5	555.5	250.2	**1**	1748									
2	FAHFA(18:0-O-14:1)	SAHMO	579.5	583.5	250.2	**2**	1818	1906								
3	FAHFA(18:1-O-14:1)	OAHMO	577.5	581.5	250.2	**2**	1761	1907								
4	FAHFA(18:2-O-14:1)	LAHMO	575.5	579.5	250.2	**1**	1760									
5	FAHFA(16:0-O-14:2)	PAHTDA	549.5	553.5	248.2	**1**	1747									
6	FAHFA(17:0-O-14:2)	HDAHTDA	563.5	567.5	248.2	**1**	1813									
7	FAHFA(18:0-O-14:2)	SAHTDA	577.5	581.5	248.2	**3**	1760	1817	1904							
8	FAHFA(18:1-O-14:2)	OAHTDA	575.5	579.5	248.2	**1**	1759									
9	FAHFA(20:0-O-14:2)	AAHTDA	605.5	609.5	248.2	**2**	2008	2098								
10	FAHFA(20:1-O-14:2)	EAHTDA	603.5	608.5	248.2	**1**	1918									
11	FAHFA(22:6-O-14:2)	DHAHTDA	621.5	625.5	248.2	**1**	1813									
12	FAHFA(18:2-O-18:2)	LAHLA	629.5	633.5	304.2	**1**	2393									
13	FAHFA(16:0-O-12:0)	PAHDA	525.5	529.5	224.2	**1**	2198									
14	FAHFA(15:1-O-15:0)	PDEAHPDA	551.5	555.5	266.2	**1**	1738									
15	FAHFA(17:1-O-15:0)	HDEAHPDA	579.5	583.5	266.2	**1**	1898									
16	FAHFA(22:1-O-15:0)	DEAHPDA	649.5	653.5	266.2	**1**	1995									
17	FAHFA(16:0-O-16:0)	PAHPA	581.6	585.6	280.3	**10**	2494	2542	2566	2590	2615	2646	2678	2707	2758	2810
18	FAHFA(18:0-O-16:0)	SAHPA	609.6	613.6	280.3	**5**	2762	2787	2814	2917	2962					
19	FAHFA(18:1-O-16:0)	OAHPA	607.6	613.6	280.3	**3**	2644	2761	2809							
20	FAHFA(16:0-O-18:0)	PAHSA	609.8	613.8	308.3	**8**	2633	2662	2681	2701	2724	2753	2914	2958		
21	FAHFA(18:0-O-18:0)	SAHSA	637.8	641.8	308.3	**5**	2843	2885	2909	3059	3101					
22	FAHFA(18:1-O-18:0)	OAHSA	635.8	639.8	308.3	**4**	2721	2749	2911	2954						
23	FAHFA(18:1-O-19:0)	OAHNDA	649.8	653.8	322.3	**2**	2268	2324								
24	FAHFA(18:1-O-20:0)	OAHAA	663.8	667.8	336.3	**1**	2728									
25	FAHFA(20:1-O-20:0)	EAHAA	691.8	695.8	336.3	**1**	2878									
26	FAHFA(20:2-O-20:0)	EDAHAA	689.8	693.8	336.3	**1**	2737									

t , retention index

Fish (*Carassius auratus*)

NO.	Normal name	FAHFA ID	*m/z* (DMED labeling)	*m/z* (d_4-DMED labeling)	Product ion	Total number	*t1*	*t2*	*t3*	*t4*	*t5*	*t6*	*t7*	*t8*	*t9*	*t10*	*t11*
1	FAHFA(18:3-O-14:1)	ALAHMO	573.5	577.5	250.2	**1**	2057										
2	FAHFA(18:3-O-14:2)	ALAHTDA	571.5	575.5	248.2	**1**	1904										
3	FAHFA(15:1-O-20:1)	PDEAHEA	619.5	623.5	334.2	**1**	1896										
4	FAHFA(17:1-O-20:1)	HDEAHEA	647.5	651.5	334.2	**1**	2101										
5	FAHFA(20:1-O-20:1)	EAHEA	689.5	693.5	334.2	**1**	1967										
6	FAHFA(22:1-O-20:1)	DEAHEA	717.5	721.5	334.2	**2**	1874	2139									
7	FAHFA(22:2-O-20:1)	DDAHEA	715.5	719.5	334.2	**1**	1977										
8	FAHFA(15:1-O-20:2)	PDEAHEDA	617.5	621.5	332.2	**1**	1927										
9	FAHFA(17:1-O-20:2)	HDEAHEDA	645.5	649.5	332.2	**1**	2100										
10	FAHFA(18:2-O-20:2)	LAHEDA	657.5	661.5	332.2	**2**	2163	2197									
11	FAHFA(18:4-O-20:2)	SDAHEDA	653.5	657.5	332.2	**1**	1898										
12	FAHFA(20:0-O-20:2)	AAHEDA	689.5	693.5	332.2	**3**	1818	1865	1966								
13	FAHFA(20:1-O-20:2)	EAHEDA	687.5	691.5	332.2	**2**	1930	1966									
14	FAHFA(22:1-O-20:2)	DEAHEDA	715.5	719.5	332.2	**3**	1873	2091	2140								
15	FAHFA(22:2-O-20:2)	DDAHEDA	713.5	717.5	332.2	**1**	1976										
16	FAHFA(16:0-O-12:0)	PAHDA	525.5	529.5	224.2	**1**	2228										
17	FAHFA(22:6-O-12:0)	DHAHDA	597.5	601.5	224.2	**1**	2463										
18	FAHFA(16:0-O-14:0)	PAHMA	553.5	557.5	252.2	**1**	2378										
19	FAHFA(22:6-O-14:0)	DHAHMA	625.5	629.5	252.2	**1**	2639										
20	FAHFA(16:0-O-16:0)	PAHPA	581.6	585.6	280.3	**11**	2523	2549	2580	2601	2629	2658	2686	2717	2718	2798	2853
21	FAHFA(18:0-O-16:0)	SAHPA	609.6	613.6	280.3	**2**	2960	3006									
22	FAHFA(18:1-O-16:0)	OAHPA	607.6	611.6	280.3	**1**	2856										
23	FAHFA(22:6-O-16:0)	DHAHPA	653.6	657.6	280.3	**2**	2805	2846									
24	FAHFA(18:1-O-18:0)	OAHSA	635.8	639.8	308.3	**1**	2767										
25	FAHFA(22:6-O-18:0)	DHAHSA	681.8	685.8	308.3	**2**	2958	2992									

t , retention index
