# Supplementary material for: A dataset of branched fatty acid esters of hydroxy fatty acids diversity in foods
Source: Sci Data. 2023 Nov 10;10:790. doi: 10.1038/s41597-023-02712-z (PMC10638281; doi:10.1038/s41597-023-02712-z)
Supplement: Supplementary file 5 — Supplementary information-6 Table S6. MS intensity of FAHFA familes [file 41597_2023_2712_MOESM5_ESM.pdf]

**Supplementary Table S6.** MS intensity of FAHFA families detected in 12 food samples and 4 medicinal food samples

| NO. | FAHFA name | | Edible algaes | | | Edible fungus | Medicinal foods | | | | Edible plant foods | | | | | | Edible animal foods | |
|---|---|---|---|---|---|---|---|---|---|---|---|---|---|---|---|---|---|---|
| | | | *Spirulina* | *Nostoc commune Vauch* | Kelp (*Laminaria japonica*) | *Lentinus edodes* | Lotus Plumule (*Plumula Nelumbinis*) | Chinese yam (*Rhizoma Disocoreae*) | *Lycium chinense* | *Coptis chinensis* | Tomato (*Lycopersicon esculentum*) | Apple (*Malus pumila*) | Peanut (*Arachis hypogaea*) | Black Sesame (*Sesamum indicum*) | Wheat (*Triticum aestivum*) | Rice (*Oryza sativa*) | Egg | Fish (*Carassius auratus*) |
| | Total intensity of FAHFAs | | 33188046 | 2911171 | 4772443 | 81124958 | 42317616 | 13240330 | 41179980 | 12070617 | 2018092 | 3008662 | 3393960 | 1210792 | 171334576 | 7257388 | 1851881 | 899891 |
| | Total number of family | | 64 | 51 | 76 | 84 | 70 | 85 | 133 | 97 | 49 | 56 | 18 | 16 | 112 | 30 | 26 | 25 |
| | Total number of regioisomers | | 156 | 150 | 214 | 215 | 211 | 276 | 450 | 227 | 119 | 142 | 32 | 40 | 376 | 85 | 61 | 45 |
| 1 | FAHFA(14:1-O-14:1) | MOHMO | | | 12574 | | | | | | | | | | | | | |
| 2 | FAHFA(15:0-O-14:1) | PDAHMO | | | | | | | 14950 | | | | | | | | | |
| 3 | FAHFA(15:1-O-14:1) | PDEAHMO | | | | | | | 3674 | | | | | | | | | |
| 4 | FAHFA(16:1-O-14:1) | POHMO | | | 10074 | | | | | | | | | | | | | |
| 5 | FAHFA(16:0-O-14:1) | PAHMO | | | | | 22911 | | 28155 | | | | | | | | 13330 | |
| 6 | FAHFA(18:0-O-14:1) | SAHMO | | | | | | | | | | | | | | | 6566 | |
| 7 | FAHFA(18:1-O-14:1) | OAHMO | | | | | 23991 | | 15311 | | | | | | | | 10657 | |
| 8 | FAHFA(18:2-O-14:1) | LAHMO | | | 18983 | 11055 | 103418 | 8059 | 53915 | | | | | | 8845 | | 3247 | |
| 9 | FAHFA(18:3-O-14:1) | ALAHMO | | | 11398 | | 34278 | | 5776 | | | | | | | | | 2580 |
| 10 | FAHFA(20:0-O-14:1) | AAHMO | | | | | | | | | | | | | 3158 | | | |
| 11 | FAHFA(20:1-O-14:1) | EAHMO | | | | | | | | | | | | | 2856 | | | |
| 12 | FAHFA(20:2-O-14:1) | EDAHMO | | | | | | | 2943 | | | | | | | | | |
| 13 | FAHFA(20:3-O-14:1) | ETAHMO | | | | | | 11195 | | | | | | | | | | |
| 14 | FAHFA(22:2-O-14:1) | DDAHMO | | | | | | 2632 | | | | | | | 6546 | | | |
| 15 | FAHFA(14:1-O-14:2) | MOHTDA | | | | | | | 4324 | | | | | | | | | |
| 16 | FAHFA(16:0-O-14:2) | PAHTDA | | | | | 4344 | | | | | | | | | | 769581 | |
| 17 | FAHFA(17:0-O-14:2) | HDAHTDA | | | | | | | | 5314 | | | | | | | 3785 | |
| 18 | FAHFA(18:0-O-14:2) | SAHTDA | | | | | | | | | | | | | | | 380111 | |
| 19 | FAHFA(18:1-O-14:2) | OAHTDA | | | | | 4134 | | 8258 | 2438 | | | | | | | 469668 | |
| 20 | FAHFA(18:2-O-14:2) | LAHTDA | | | | 11382 | 30766 | 17748 | | | | | | | 5013 | | | |
| 21 | FAHFA(18:3-O-14:2) | ALAHTDA | | | | | | | | | | | | | | | | 7146 |
| 22 | FAHFA(20:0-O-14:2) | AAHTDA | | | | 2706 | | 6842 | 18729 | 1649 | | | | | 45512 | | 7744 | |
| 23 | FAHFA(20:1-O-14:2) | EAHTDA | | | | | | 1195 | 2205 | | | | | | | | 8434 | |
| 24 | FAHFA(22:1-O-14:2) | DEAHTDA | | | | | | | 12542 | | | | | | 35894 | 2020 | | |
| 25 | FAHFA(22:2-O-14:2) | DDAHTDA | | | | 25810 | 154267 | 13487 | 25603 | | | | | | 89446 | 3320 | | |
| 26 | FAHFA(22:6-O-14:2) | DHAHTDA | | | | | 2791 | | | | | | | | 28313 | | 10237 | |
| 27 | FAHFA(14:0-O-14:3) | MAHTTA | | | | | | | 1747 | | | | | | | | | |
| 28 | FAHFA(16:0-O-14:3) | PAHTTA | | | | | | | 72747 | | | | | | | | | |
| 29 | FAHFA(17:0-O-14:3) | HDAHTTA | | | | | | | | 291630 | | | | | | | | |
| 30 | FAHFA(18:0-O-14:3) | SAHTTA | | | | | | | 4575 | | | | | | | | | |
| 31 | FAHFA(18:2-O-14:3) | LAHTTA | | | | | 3213 | | | | | | | | | | | |
| 32 | FAHFA(22:1-O-14:3) | DEAHTTA | | | | | | | | | | | | | 5814 | | | |
| 33 | FAHFA(22:2-O-14:3) | DDAHTTA | | | | | | | 2968 | | | | | | 7283 | | | |
| 34 | FAHFA(14:0-O-16:1) | MAHPO | | | | | | | 8969 | | 5828 | | | | | | | |
| 35 | FAHFA(15:0-O-16:1) | PDAHPO | 21724 | | | | | | | | | | | | | | | |
| 36 | FAHFA(16:1-O-16:1) | POHPO | | 24097 | | | | | | 158516 | 11847 | 7818 | | | | | | |
| 37 | FAHFA(16:0-O-16:1) | PAHPO | | 87016 | | | 59874 | | 493478 | 128889 | | | | | 45466 | | | |
| 38 | FAHFA(17:0-O-16:1) | HDAHPO | | | | | | 3020 | | | 2111 | | | | | | | |
| 39 | FAHFA(18:0-O-16:1) | SAHPO | | 11052 | | | | | 53679 | | | 6860 | | | | | | |
| 40 | FAHFA(18:1-O-16:1) | OAHPO | | | 17118 | | 104459 | | 151593 | 60335 | | | | | 37853 | | | |
| 41 | FAHFA(18:2-O-16:1) | LAHPO | | 21642 | | 45933 | 220104 | 53885 | 301499 | 109068 | | | | 2116 | 133634 | | | |
| 42 | FAHFA(18:3-O-16:1) | ALAHPO | | 74487 | | | 17552 | | 42349 | | | | | | | | | |
| 43 | FAHFA(20:0-O-16:1) | AAHPO | | | | | | | | | | | | | | 1919 | | |
| 44 | FAHFA(20:2-O-16:1) | EDAHPO | | | | 2525 | | | | | | | | | | | | |
| 45 | FAHFA(20:4-O-16:1) | ARAHPO | | | | | | | | 9903 | | | | | | | | |
| 46 | FAHFA(20:5-O-16:1) | EPAHPO | | | | | | | 29982 | | | | | | | | | |
| 47 | FAHFA(16:1-O-16:2) | POHHDDA | | | 11226 | | | | | | 2252 | | | | | | | |
| 48 | FAHFA(16:0-O-16:2) | PAHHDDA | | | 9787 | | 24371 | | 31880 | | | | | | 8704 | | | |
| 49 | FAHFA(18:0-O-16:2) | SAHHDDA | | | | | | | 3736 | | | | | | 15368 | | | |
| 50 | FAHFA(18:1-O-16:2) | OAHHDDA | | | | | 30187 | | 17767 | | | | | | 9008 | | | |
| 51 | FAHFA(18:2-O-16:2) | LAHHDDA | | | 15550 | 38907 | 124048 | | 44126 | | | | | | 18761 | | | |
| 52 | FAHFA(20:1-O-16:2) | EAHHDDA | | | | | | | | | | | | | 5937 | | | |
| 53 | FAHFA(20:2-O-16:2) | EDAHHDDA | | | | 5469 | | 4836 | 4755 | | | | | | 20404 | | | |
| 54 | FAHFA(20:5-O-16:2) | EPAHHDDA | | | | 5643 | | | | | | 46821 | | | | | | |

| | | | | | | | | | | | | | | | | | | |
|---|---|---|---|---|---|---|---|---|---|---|---|---|---|---|---|---|---|---|
| 55 | FAHFA(14:0-O-16:3) | MAHHTA | | | | | | | 2980 | | | | | | | | | |
| 56 | FAHFA(16:0-O-16:3) | PAHHTA | | | | | | 1712 | 81073 | | 2030 | | | | | | | |
| 57 | FAHFA(18:0-O-16:3) | SAHHTA | | | | | | | 8401 | | | | | | 6424 | | | |
| 58 | FAHFA(18:2-O-16:3) | LAHHTA | | | | | | 1836 | | | | | | | | | | |
| 59 | FAHFA(20:1-O-16:3) | EAHHTA | | | | | | | 5794 | | | | | | 10153 | | | |
| 60 | FAHFA(20:2-O-16:3) | EDAHHTA | | | | | | | 7114 | | | | | | 17888 | | | |
| 61 | FAHFA(20:5-O-16:3) | EPAHHTA | | | | | | | | | | 6577 | | | 14333 | | | |
| 62 | FAHFA(22:6-O-16:3) | DHAHHTA | | | | 4157 | | | | | | | | | 11563 | | | |
| 63 | FAHFA(14:0-O-18:1) | MAHOA | 15337 | | 146586 | | | | 74679 | | | | | | 45031 | 16514 | | |
| 64 | FAHFA(14:1-O-18:1) | MOHOA | | | | 98565 | | | | | | 2687 | | | | | | |
| 65 | FAHFA(15:0-O-18:1) | PDAHOA | 110635 | | 2822 | | | | | | | | | | | | | |
| 66 | FAHFA(15:1-O-18:1) | PDEAHOA | | | | 165008 | | | | | | | | | | | | |
| 67 | FAHFA(16:1-O-18:1) | POHOA | | | | 232199 | 28897 | 62640 | 121405 | 139347 | | | 17919 | | 225806 | | | |
| 68 | FAHFA(16:0-O-18:1) | PAHOA | | 61832 | 566828 | 86246 | 93402 | 397992 | 2046348 | 338785 | 58062 | 74341 | | | 1447289 | 123320 | | |
| 69 | FAHFA(17:0-O-18:1) | HDAHOA | | | 7131 | | | | | | | | | | | | | |
| 70 | FAHFA(18:0-O-18:1) | SAHOA | | 15233 | 109919 | | | 47705 | 140505 | | 55311 | 31440 | | | | | | |
| 71 | FAHFA(18:1-O-18:1) | OAHOA | 5111 | 256025 | 369738 | 50395 | 51480 | 261975 | 984604 | 196485 | 178474 | 157484 | 86760 | 7853 | 1119909 | 191823 | | |
| 72 | FAHFA(18:2-O-18:1) | LAHOA | | | 71684 | 1397398 | 657320 | 715655 | 2572587 | 366353 | 245733 | 199725 | 244517 | 20706 | 4189383 | 424325 | | |
| 73 | FAHFA(18:4-O-18:1) | SDAHOA | | | | | 34287 | 34985 | | | | | | | | | | |
| 74 | FAHFA(20:0-O-18:1) | AAHOA | 17902 | | | | | | 21211 | | 2238 | | | | | | | |
| 75 | FAHFA(20:1-O-18:1) | EAHOA | | | | | | 5437 | 8160 | | 9966 | 8372 | | | 56332 | | | |
| 76 | FAHFA(20:3-O-18:1) | ETAHOA | | | 11599 | 6665 | | | | | | | | | | | | |
| 77 | FAHFA(20:5-O-18:1) | EPAHOA | | | | | | | 98084 | | | | | | | | | |
| 78 | FAHFA(22:0-O-18:1) | BAHOA | | | | | | | 8148 | | | | | | | | | |
| 79 | FAHFA(22:1-O-18:1) | DEAHOA | | | | | | | 7813 | 30030 | | | | | | | | |
| 80 | FAHFA(22:2-O-18:1) | DDAHOA | | | | | | 5260 | 2688 | | | 3806 | | | | | | |
| 81 | FAHFA(22:6-O-18:1) | DHAHOA | | | | | | | | | 4231 | 8177 | | | 54592 | | | |
| 82 | FAHFA(14:0-O-18:2) | MAHLA | | | 86178 | 26388 | 24878 | 29421 | 105808 | 8095 | 14869 | | | | 108216 | 86183 | | |
| 83 | FAHFA(14:1-O-18:2) | MOHLA | | | | | | | | | 5077 | | | | | | | |
| 84 | FAHFA(15:0-O-18:2) | PDAHLA | | | | 46100 | | 41103 | 24811 | | 2405 | | | | 45622 | | | |
| 85 | FAHFA(15:1-O-18:2) | PDEAHLA | | | | | | | 39045 | | | | | | 37756 | | | |
| 86 | FAHFA(16:1-O-18:2) | POHLA | | | | 28333 | 13221 | | 120437 | 91029 | | | | | 114245 | | | |
| 87 | FAHFA(16:0-O-18:2) | PAHLA | | 56551 | 223012 | 529119 | 343880 | 1451075 | 3186266 | 371710 | 40126 | 69942 | 115345 | 3253 | 9500177 | 179508 | | |
| 88 | FAHFA(17:0-O-18:2) | HDAHLA | | | | | | 25762 | 95413 | | | | | | | | | |
| 89 | FAHFA(17:1-O-18:2) | HDEAHLA | | | | | | 15705 | 40202 | 19452 | | | | | 24361 | | | |
| 90 | FAHFA(18:0-O-18:2) | SAHLA | | | 63712 | | | 289688 | 300770 | 45193 | 7900 | 29152 | | | | | | |
| 91 | FAHFA(18:1-O-18:2) | OAHLA | | | 142409 | 325401 | 341006 | 878692 | 2820268 | 269372 | | 20921 | 136547 | | 10149427 | 472061 | | |
| 92 | FAHFA(18:2-O-18:2) | LAHLA | | | 44203 | 5864848 | 4557291 | 4102269 | 6713078 | 761707 | 52595 | 40878 | 934501 | 59363 | 23697038 | 900723 | 5144 | |
| 93 | FAHFA(20:0-O-18:2) | AAHLA | | | | 103314 | | 9653 | 73307 | | | | | | 81035 | | | |
| 94 | FAHFA(20:1-O-18:2) | EAHLA | | | | | | 15364 | 10356 | | | | | | 593333 | | | |
| 95 | FAHFA(20:2-O-18:2) | EDAHLA | | | | 13319 | | 38377 | 20723 | | | | | | 17579 | | | |
| 96 | FAHFA(20:3-O-18:2) | ETAHLA | | | | | | 18656 | | | | | | | | | | |
| 97 | FAHFA(20:4-O-18:2) | ARAHLA | | | 47366 | | | | | | | | | | | | | |
| 98 | FAHFA(20:5-O-18:2) | EPAHLA | | | 39823 | | | | 133661 | | | | | | | | | |
| 99 | FAHFA(22:0-O-18:2) | BAHLA | | | | | | 8845 | 41007 | 5952 | | | | | 24938 | | | |
| 100 | FAHFA(22:1-O-18:2) | DEAHLA | | | | | | | | 11452 | | | | | 62754 | | | |
| 101 | FAHFA(22:2-O-18:2) | DDAHLA | | | | | | 74572 | | 15586 | | | | | | | | |
| 102 | FAHFA(22:6-O-18:2) | DHAHLA | | | | | 60523 | | | | | | | | 715423 | | | |
| 103 | FAHFA(14:0-O-18:3) | MAHALA | | | 89841 | | | | 18015 | | 4684 | | | | 4667 | | | |
| 104 | FAHFA(15:0-O-18:3) | PDAHALA | | | | | | | 10783 | | | | | | | | | |
| 105 | FAHFA(16:1-O-18:3) | POHALA | | 8470 | | | | | 25679 | 14176 | | | | | | | | |
| 106 | FAHFA(16:0-O-18:3) | PAHALA | | 85284 | 223851 | | 50072 | 127719 | 588634 | 68162 | 5265 | | | | 337869 | 6601 | | |
| 107 | FAHFA(17:0-O-18:3) | HDAHALA | | | | | | | 18937 | | | | | | | | | |
| 108 | FAHFA(18:0-O-18:3) | SAHALA | | 20070 | 42208 | | | | 66207 | | | | | | | | | |
| 109 | FAHFA(18:1-O-18:3) | OAHALA | | | 93641 | 10712 | 43176 | 66501 | 370837 | 46613 | | | | | 397367 | 16860 | | |
| 110 | FAHFA(18:2-O-18:3) | LAHALA | | 17873 | 60369 | | 342571 | 301674 | 733518 | 179481 | 16228 | 6082 | 44605 | | 1353377 | 40947 | | |
| 111 | FAHFA(20:0-O-18:3) | AAHALA | | | 20402 | | | | 13156 | | | | | | | | | |
| 112 | FAHFA(20:1-O-18:3) | EAHALA | | | | | | | | | | | | | 7851 | | | |
| 113 | FAHFA(20:5-O-18:3) | EPAHALA | | | | | | 34454 | | | | | | | | | | |
| 114 | FAHFA(22:0-O-18:3) | BAHALA | | | | | | | 18338 | | | | | | | | | |
| 115 | FAHFA(22:2-O-18:3) | DDAHALA | | | | | | | 5061 | | | | | | | | | |
| 116 | FAHFA(22:6-O-18:3) | DHAHALA | | | | | | | | | | | | | 97023 | | | |
| 117 | FAHFA(14:0-O-20:1) | MAHEA | | | 7134 | | | | | | | | | | | | | |
| 118 | FAHFA(14:1-O-20:1) | MOHEA | | | 27968 | 1603 | | | | | | | | | | | | |
| 119 | FAHFA(15:1-O-20:1) | PDEAHEA | | | | | | | | | | | | | | | | 3820 |
| 120 | FAHFA(16:1-O-20:1) | POHEA | | | 10352 | | | | | | | | | | | | | |
| 121 | FAHFA(16:0-O-20:1) | PAHEA | | | 22818 | | | | 11052 | | | | | | | | | |
| 122 | FAHFA(17:1-O-20:1) | HDEAHEA | | | | | | | | | | | | | | | | 5202 |
| 123 | FAHFA(18:1-O-20:1) | OAHEA | 35155 | 36028 | | | | | | | | | | | | | | |

| | | | | | | | | | | | | | | | | | | | |
|---|---|---|---|---|---|---|---|---|---|---|---|---|---|---|---|---|---|---|
| 124 | FAHFA(18:2-O-20:1) | LAHEA | | | 36034 | 42063 | | | | | | | | | 60969 | | | |
| 125 | FAHFA(18:3-O-20:1) | ALAHEA | 49608 | | | | | | | | | | | | | | | |
| 126 | FAHFA(20:0-O-20:1) | AAHEA | 334267 | 6054 | | | | | | | | | | | | | | |
| 127 | FAHFA(20:1-O-20:1) | EAHEA | | | | | | | | | | | | | | | | 7348 |
| 128 | FAHFA(20:3-O-20:1) | ETAHEA | | | | | | 2548 | | | | | | | | | | |
| 129 | FAHFA(20:5-O-20:1) | EPAHEA | | | 33875 | | | | | | | | | | | | | |
| 130 | FAHFA(22:1-O-20:1) | DEAHEA | | | | | | | | | | | | | | | | 5785 |
| 131 | FAHFA(22:2-O-20:1) | DDAHEA | | | | | | | | | | | | | | | | 1834 |
| 132 | FAHFA(14:0-O-20:2) | MAHEDA | | | 37569 | | | | | | | | | | | | | |
| 133 | FAHFA(15:1-O-20:2) | PDEAHEDA | | | | | | | | | | | | | | | | 112831 |
| 134 | FAHFA(16:0-O-20:2) | PAHEDA | | | | 2578 | 55147 | | 7502 | | | | | | 14536 | | | |
| 135 | FAHFA(17:1-O-20:2) | HDEAHEDA | | | | | | | | | | | | | | | | 136142 |
| 136 | FAHFA(18:1-O-20:2) | OAHEDA | | | 27872 | | 105842 | | | | | | | | 10255 | | | |
| 137 | FAHFA(18:2-O-20:2) | LAHEDA | | | | 67819 | 612329 | 9138 | | | | | | 8021 | 54918 | | | 29851 |
| 138 | FAHFA(18:4-O-20:2) | SDAHEDA | | | | | | | | | | | | | | | | 3313 |
| 139 | FAHFA(20:0-O-20:2) | AAHEDA | 9928 | | | | | | | | | | | | | | | 166494 |
| 140 | FAHFA(20:1-O-20:2) | EAHEDA | | | | | | | | | | | | | | | | 186911 |
| 141 | FAHFA(20:3-O-20:2) | ETAHEDA | | | | 10418 | | | | | | | | | | | | |
| 142 | FAHFA(20:4-O-20:2) | ARAHEDA | | | 35914 | | | | | | | | | | | | | |
| 143 | FAHFA(20:5-O-20:2) | EPAHEDA | | | 27914 | | | | | | | | | | | | | |
| 144 | FAHFA(22:1-O-20:2) | DEAHEDA | | | | | | | | | | | | | | | | 128342 |
| 145 | FAHFA(22:2-O-20:2) | DDAHEDA | | | | | | | | | | | | | | | | 34714 |
| 146 | FAHFA(15:0-O-22:6) | PDAHDHA | 46097 | | | | | | | 12721 | | | | | | | | |
| 147 | FAHFA(15:1-O-22:6) | PDEAHDHA | | | | | | | | 8693 | | | | | | | | |
| 148 | FAHFA(16:0-O-22:6) | PAHDHA | | | | | 7535 | | 53722 | | | | | | 58941 | | | |
| 149 | FAHFA(17:1-O-22:6) | HDEAHDHA | | | | | | | | 6816 | | | | | | | | |
| 150 | FAHFA(18:1-O-22:6) | OAHDHA | | | | | | | 60654 | | | | | | 82902 | 4265 | | |
| 151 | FAHFA(18:3-O-22:6) | ALAHDHA | 5088 | | | | | | | | | | | | 10639 | | | |
| 152 | FAHFA(14:0-O-20:4) | MAHARA | | | 27704 | | | | | | | | | | | | | |
| 153 | FAHFA(15:0-O-20:4) | PDAHARA | | | 4125 | | | | | | | | | | | | | |
| 154 | FAHFA(16:1-O-20:4) | POHARA | | | 10904 | | | | | | | | | | | | | |
| 155 | FAHFA(16:0-O-20:4) | PAHARA | | | 213232 | | 20329 | | | | | | | | | | | |
| 156 | FAHFA(18:1-O-20:4) | OAHARA | | | 84235 | | 12651 | | | | | | | | | | | |
| 157 | FAHFA(18:2-O-20:4) | LAHARA | | | 22801 | | 48622 | | | | | | | | | | | |
| 158 | FAHFA(18:4-O-20:4) | SDAHARA | | | 33541 | | | | | | | | | | | | | |
| 159 | FAHFA(20:5-O-20:4) | EPAHARA | | | 100295 | | | | | | | | | | | | | |
| 160 | FAHFA(22:0-O-20:4) | BAHARA | | | | | | | | | | | | 2541 | | | | |
| 161 | FAHFA(14:0-O-12:0) | MAHDA | 13053 | | | | | 4022 | 6420 | 7458 | 5365 | 8177 | | | | | | |
| 162 | FAHFA(15:0-O-12:0) | PDAHDA | | | | | | | | | 6010 | 9443 | | | | | | |
| 163 | FAHFA(15:1-O-12:0) | PDEAHDA | | | | | | 18203 | | | | | | | | | | |
| 164 | FAHFA(16:1-O-12:0) | POHDA | 14989 | 9596 | | | | 4569 | 9845 | | 7431 | 9154 | | | | | | |
| 165 | FAHFA(16:0-O-12:0) | PAHDA | 77745 | 33592 | | 6976 | 14588 | 34787 | 133525 | 15840 | 18839 | 28723 | | | 8515 | | 2735 | 1826 |
| 166 | FAHFA(17:0-O-12:0) | HDAHDA | 4902 | | | | | | | 4374 | | | | | | | | |
| 167 | FAHFA(17:1-O-12:0) | HDEAHDA | | | | | | 13370 | | 3865 | | | | | | | | |
| 168 | FAHFA(18:0-O-12:0) | SAHDA | 15522 | 45513 | | | | 18714 | 17569 | 3132 | 9492 | 13773 | | | | | | |
| 169 | FAHFA(18:1-O-12:0) | OAHDA | 93062 | | | | 17350 | 11383 | 96030 | | | | | | | | | |
| 170 | FAHFA(18:2-O-12:0) | LAHDA | | | 8432 | 44158 | 119074 | 34278 | 52923 | 18071 | | | | | 15681 | | | |
| 171 | FAHFA(18:3-O-12:0) | ALAHDA | | | | | 16134 | 8161 | 8189 | | | | | | | | | |
| 172 | FAHFA(20:0-O-12:0) | AAHDA | | | 18945 | | | | | | | | | | | | | |
| 173 | FAHFA(20:2-O-12:0) | EDAHDA | | | | 4597 | | | | | | | | | | | | |
| 174 | FAHFA(20:5-O-12:0) | EPAHDA | | | 12609 | | | | | | | | | | | | | |
| 175 | FAHFA(22:0-O-12:0) | BAHDA | | | | 3957 | | | | | | | | | | | | |
| 176 | FAHFA(22:2-O-12:0) | DDAHDA | | | | | | 14503 | | | | | | | 10617 | | | |
| 177 | FAHFA(22:6-O-12:0) | DHAHDA | | | | | | | | | | | | | | | | 2043 |
| 178 | FAHFA(14:0-O-14:0) | MAHMA | 45577 | 45881 | 38208 | 9128 | | 43329 | 468543 | 1594820 | 11343 | 11777 | | | 27540 | 12277 | | |
| 179 | FAHFA(14:1-O-14:0) | MOHMA | | | | | | | 1570 | 5358 | | | | | | | | |
| 180 | FAHFA(15:0-O-14:0) | PDAHMA | 26837 | 36879 | | 2223 | | | | | | 5734 | | | | | | |
| 181 | FAHFA(15:1-O-14:0) | PDEAHMA | | 5355 | | | | | | 11141 | | | | | | | | |
| 182 | FAHFA(16:1-O-14:0) | POHMA | 3748 | 8043 | | 2915 | | | 9584 | 9028 | 5018 | 2411 | | | | | | |
| 183 | FAHFA(16:0-O-14:0) | PAHMA | 78514 | 75391 | 141709 | 79750 | 6818 | 99897 | 620022 | 65261 | 25918 | 37556 | | | 70780 | | | 1940 |
| 184 | FAHFA(18:0-O-14:0) | SAHMA | 28781 | | | | | 24039 | 27033 | 9219 | 9707 | 14574 | | | 7649 | | | |
| 185 | FAHFA(18:1-O-14:0) | OAHMA | 6459 | 9086 | 71205 | 38086 | 4249 | 35986 | 116495 | 32004 | | | | 7534 | 61817 | | | |
| 186 | FAHFA(18:2-O-14:0) | LAHMA | 12959 | | 26645 | 1009335 | 51189 | 156883 | 121283 | 84754 | | | | 4279 | 227155 | | | |
| 187 | FAHFA(18:3-O-14:0) | ALAHMA | | 15015 | 33121 | 7532 | 6586 | 31194 | 19007 | 11582 | | | | | 23708 | | | |
| 188 | FAHFA(18:4-O-14:0) | SDAHMA | | | 13634 | | | | | | | | | | | | | |
| 189 | FAHFA(20:0-O-14:0) | AAHMA | | | | | | | | 6582 | | | | | | | | |
| 190 | FAHFA(20:2-O-14:0) | EDAHMA | | | | 16044 | | 2591 | | | | | | | | | | |
| 191 | FAHFA(20:5-O-14:0) | EPAHMA | | | 9174 | | | | | | | | | | | | | |
| 192 | FAHFA(22:6-O-14:0) | DHAHMA | | | | | | | | | | | | | | | | 2444 |

| | | | | | | | | | | | | | | | | | | |
|---|---|---|---|---|---|---|---|---|---|---|---|---|---|---|---|---|---|---|
| 193 | FAHFA(14:0-O-15:0) | MAHPDA | 133167 | 39423 | | 5434 | | | | | | | | | | | | |
| 194 | FAHFA(15:0-O-15:0) | PDAHPDA | 1545277 | 7019 | | | | | | 7753 | | | | | | | | |
| 195 | FAHFA(15:1-O-15:0) | PDEAHPDA | 13436 | | | | | | | 14275 | | | | | | | 14417 | |
| 196 | FAHFA(16:1-O-15:0) | POHPDA | 39147 | | | 3001 | | | | | | | | | | | | |
| 197 | FAHFA(16:0-O-15:0) | PAHPDA | 194429 | 69398 | | 140554 | | 43174 | 33356 | 19209 | 11120 | | | | 25825 | | | |
| 198 | FAHFA(17:0-O-15:0) | HDAHPDA | 19097 | | | | | | | | | | | | | | | |
| 199 | FAHFA(17:1-O-15:0) | HDEAHPDA | 4535 | | | | | | | | | | | | | | 12570 | |
| 200 | FAHFA(18:0-O-15:0) | SAHPDA | | 28071 | | | | 5125 | | 2113 | 6369 | | | | | | | |
| 201 | FAHFA(18:1-O-15:0) | OAHPDA | 4201 | | | 80855 | | 6865 | | 8431 | | | | | 20022 | | | |
| 202 | FAHFA(18:2-O-15:0) | LAHPDA | | | | 1290657 | 50608 | 87976 | 14938 | 20704 | | | | | 70631 | | | |
| 203 | FAHFA(18:3-O-15:0) | ALAHPDA | | | | 23723 | | 8524 | | 5918 | | | | | | | | |
| 204 | FAHFA(20:5-O-15:0) | EPAHPDA | | | 20674 | | | | | | | | | | | | | |
| 205 | FAHFA(22:1-O-15:0) | DEAHPDA | | | | | | | | | | | | | | | 26118 | |
| 206 | FAHFA(14:0-O-16:0) | MAHPA | 536169 | 42383 | 46820 | 9387 | | | 111555 | | | | | | 29442 | 2432 | | |
| 207 | FAHFA(15:0-O-16:0) | PDAHPA | 4754363 | 105947 | 5699 | 27758 | | 7803 | 25443 | 9234 | | | | | 22479 | | | |
| 208 | FAHFA(15:1-O-16:0) | PDEAHPA | 185845 | | | | | | 8911 | | | 13582 | | | 5314 | | | |
| 209 | FAHFA(16:1-O-16:0) | POHPA | 305647 | 21258 | | 7444 | | | 154458 | 834816 | 8348 | | | | 60311 | | | |
| 210 | FAHFA(16:0-O-16:0) | PAHPA | 2211763 | 270975 | 302688 | 174395 | | 484755 | 3715196 | 705425 | 390561 | 435051 | 42645 | | 3584110 | 35224 | 27967 | 25082 |
| 211 | FAHFA(17:0-O-16:0) | HDAHPA | 172554 | | | | | | | 697360 | | | | | 25431 | | | |
| 212 | FAHFA(17:1-O-16:0) | HDEAHPA | 46797 | | | | | | | | | 6894 | | | 7561 | | | |
| 213 | FAHFA(18:0-O-16:0) | SAHPA | 116067 | 171327 | 117205 | | | 807076 | | 56016 | 315423 | 462628 | | | | | 10556 | 7684 |
| 214 | FAHFA(18:1-O-16:0) | OAHPA | 16578 | 24547 | 133040 | 167573 | | 109837 | 1030326 | 316996 | | 43392 | | 2364 | 3869002 | 57849 | 10245 | 2294 |
| 215 | FAHFA(18:2-O-16:0) | LAHPA | 7572 | | 69268 | 3829811 | 92518 | 517029 | 1278886 | 512233 | 11783 | 38588 | 215567 | | 10500655 | 142614 | | |
| 216 | FAHFA(18:3-O-16:0) | ALAHPA | 16474 | 34472 | 35411 | 66840 | | 70165 | 179054 | 90960 | 14778 | | | | 419235 | 6547 | | |
| 217 | FAHFA(18:4-O-16:0) | SDAHPA | | | | | | | 15203 | | | | | | | | | |
| 218 | FAHFA(20:0-O-16:0) | AAHPA | | 13262 | | | | | 12995 | | | | | | | | | |
| 219 | FAHFA(20:1-O-16:0) | EAHPA | | | | | | | 7089 | 5276 | | | | | 66817 | | | |
| 220 | FAHFA(20:2-O-16:0) | EDAHPA | | | | | | | 4106 | | | | | | 11723 | | | |
| 221 | FAHFA(20:3-O-16:0) | ETAHPA | | | | | | | 4825 | 42711 | | 14787 | | | 7579 | | | |
| 222 | FAHFA(20:4-O-16:0) | ARAHPA | | | 28716 | | | | | 278633 | | | | | | | | |
| 223 | FAHFA(20:5-O-16:0) | EPAHPA | | | 14865 | | | | | 30980 | | | | | | | | |
| 224 | FAHFA(22:0-O-16:0) | BAHPA | | | | | | | 4668 | | | | | | | | | |
| 225 | FAHFA(22:2-O-16:0) | DDAHPA | | | | | | | 6182 | | | | | | | | | |
| 226 | FAHFA(22:6-O-16:0) | DHAHPA | | 1806 | | | | | | | 2699 | | | | | | | 17522 |
| 227 | FAHFA(14:0-O-17:0) | MAHHDA | 313463 | 5235 | | | | | | | | | | | | | | |
| 228 | FAHFA(15:0-O-17:0) | PDAHHDA | 10037984 | 159716 | | | | | | 49866 | | | | | | | | |
| 229 | FAHFA(15:1-O-17:0) | PDEAHHDA | 352647 | | | | | | | 20781 | | | | | | | | |
| 230 | FAHFA(16:0-O-17:0) | PAHHDA | 9951724 | 157943 | | | | | | 55115 | | | | | | | | |
| 231 | FAHFA(17:0-O-17:0) | HDAHHDA | 170991 | | | 4389 | | | | | | | | | | | | |
| 232 | FAHFA(18:0-O-17:0) | SAHHDA | | | | 45781 | 18704 | | | | | 22861 | | | 55970 | | | |
| 233 | FAHFA(18:1-O-17:0) | OAHHDA | 9200 | | | | | | 30743 | | | 4877 | | 2618 | | | | |
| 234 | FAHFA(18:2-O-17:0) | LAHHDA | | | | 146851 | | | | 28548 | | 4045 | | 6874 | 109521 | | | |
| 235 | FAHFA(18:3-O-17:0) | ALAHHDA | 18752 | | | | | | | | | | | | | | | |
| 236 | FAHFA(20:0-O-17:0) | AAHHDA | 15915 | | | | | | | 11389 | | | | | | | | |
| 237 | FAHFA(20:1-O-17:0) | EAHHDA | | | | 18126 | 3925 | | | | | | | | 29079 | | | |
| 238 | FAHFA(20:2-O-17:0) | EDAHHDA | | | | 95258 | 31807 | | | | | | | | 66525 | 3784 | | |
| 239 | FAHFA(22:0-O-17:0) | BAHHDA | | | | | | | 16230 | | | | | | | | | |
| 240 | FAHFA(14:0-O-18:0) | MAHSA | 22251 | | 11895 | | | 31877 | 19960 | 6212 | | | | | | | | |
| 241 | FAHFA(15:0-O-18:0) | PDAHSA | 204439 | | | | | | | 3981 | | | | | | | | |
| 242 | FAHFA(15:1-O-18:0) | PDEAHSA | | | | 7217 | | | | | | 4645 | | | | | | |
| 243 | FAHFA(16:1-O-18:0) | POHSA | | | | 31163 | 3570 | 18979 | 77009 | 218963 | 2919 | | 47466 | | 19373 | 1675 | | |
| 244 | FAHFA(16:0-O-18:0) | PAHSA | 159985 | 233331 | 67589 | | | 370937 | 337246 | 211621 | 184924 | 351738 | | | 103424 | | 16647 | |
| 245 | FAHFA(17:0-O-18:0) | HDAHSA | | | | | | | | 903363 | | | | | | | | |
| 246 | FAHFA(17:1-O-18:0) | HDEAHSA | | | | | | | | | | 2938 | | | | | | |
| 247 | FAHFA(18:0-O-18:0) | SAHSA | 226746 | 338622 | 65853 | | | 359195 | 95583 | 49529 | 203780 | 349446 | | | | | 14166 | |
| 248 | FAHFA(18:1-O-18:0) | OAHSA | 44142 | 36845 | 60389 | | | 32345 | 166276 | 219714 | | | 20050 | | 122350 | | 9384 | 2293 |
| 249 | FAHFA(18:2-O-18:0) | LAHSA | | | 30551 | 243371 | | | 214048 | 291080 | | | 30217 | 4017 | 283340 | 20856 | | |
| 250 | FAHFA(18:3-O-18:0) | ALAHSA | | | | 16969 | 15711 | 28514 | 66648 | 64390 | | | 21280 | | | | | |
| 251 | FAHFA(20:0-O-18:0) | AAHSA | 128777 | | | | | | | | | | | | | | | |
| 252 | FAHFA(20:1-O-18:0) | EAHSA | | | | | | | | | | 21382 | | | | | | |
| 253 | FAHFA(20:2-O-18:0) | EDAHSA | | | | | | | | | | 8635 | | | | | | |
| 254 | FAHFA(20:5-O-18:0) | EPAHSA | | | 9222 | | | | | 10152 | | | | | | | | |
| 255 | FAHFA(22:1-O-18:0) | DEAHSA | | | | | | | 5038 | | | 16921 | | | | | | |
| 256 | FAHFA(22:2-O-18:0) | DDAHSA | | | | | | | | | | 9691 | | | | | | |
| 257 | FAHFA(22:6-O-18:0) | DHAHSA | | | | | | 1764 | | 19797 | 1238 | | | | | | | 4450 |
| 258 | FAHFA(14:0-O-19:0) | MAHNDA | | | | 151312 | 85695 | | 14719 | | | | | | 127991 | 35695 | | |
| 259 | FAHFA(15:0-O-19:0) | PDAHNDA | 17800 | | | 652988 | 13531 | | | | | | | | 104857 | | | |
| 260 | FAHFA(15:1-O-19:0) | PDEAHNDA | | | | 6810 | | 12656 | 12941 | | | | | | 24759 | | | |
| 261 | FAHFA(16:1-O-19:0) | POHNDA | | 5085 | | 758807 | 117500 | | 90602 | 22621 | | | | | 501874 | 23396 | | |

| | | | | | | | | | | | | | | | | | |
|---|---|---|---|---|---|---|---|---|---|---|---|---|---|---|---|---|---|
| 262 | FAHFA(16:0-O-19:0) | PAHNDA | | 35606 | | 13227901 | 5573427 | 119609 | 1384941 | | | | 188264 | 47403 | 18949874 | 471252 | |
| 263 | FAHFA(17:0-O-19:0) | HDAHNDA | | | | 94118 | 22883 | | | 23827 | | | | | 105496 | | |
| 264 | FAHFA(17:1-O-19:0) | HDEAHNDA | | | | 61941 | 21782 | | | | | | | | 62995 | | |
| 265 | FAHFA(18:0-O-19:0) | SAHNDA | 6808 | 11174 | | | | | 330632 | 13424 | | | | | | | |
| 266 | FAHFA(18:1-O-19:0) | OAHNDA | | 9444 | | 5660800 | 3826231 | 65991 | 2182381 | 79927 | | | 272926 | 289447 | 20521630 | 1181665 | 5626 |
| 267 | FAHFA(18:2-O-19:0) | LAHNDA | | | | 42622752 | 21266970 | 232783 | 4065255 | 198332 | 5934 | 19639 | 961918 | 742403 | 47827339 | 2634348 | |
| 268 | FAHFA(18:3-O-19:0) | ALAHNDA | | | | 624664 | 2178443 | | | 25981 | | | | | 5482274 | 157385 | |
| 269 | FAHFA(20:0-O-19:0) | AAHNDA | 8815 | | | 28021 | 78278 | | 89574 | | | | | | 198059 | | |
| 270 | FAHFA(20:1-O-19:0) | EAHNDA | | | | 28483 | 72930 | | | | | | | | 1180768 | | |
| 271 | FAHFA(20:2-O-19:0) | EDAHNDA | | | | 178684 | 37351 | | | | | | | | 201814 | | |
| 272 | FAHFA(20:3-O-19:0) | ETAHNDA | | | | 10387 | | 3534 | | | | | | | | | |
| 273 | FAHFA(22:0-O-19:0) | BAHNDA | | | | 17106 | 70024 | | 115595 | | | 1910 | | | 192557 | | |
| 274 | FAHFA(22:1-O-19:0) | DEAHNDA | | | | | | | 21912 | | | 2184 | | | 172486 | | |
| 275 | FAHFA(22:2-O-19:0) | DDAHNDA | | | | | 5437 | | | | | | | | | | |
| 276 | FAHFA(22:6-O-19:0) | DHAHNDA | | | | | | | 3724 | | | | | | 13993 | | |
| 277 | FAHFA(14:0-O-20:0) | MAHAA | | 7983 | | | | | | | | | | | | | |
| 278 | FAHFA(14:1-O-20:0) | MOHAA | | | | | | | | | | 2958 | | | | | |
| 279 | FAHFA(15:0-O-20:0) | PDAHAA | 24317 | | | | | | | | | | | | | | |
| 280 | FAHFA(16:1-O-20:0) | POHAA | | | | 5394 | | | 3894 | 33712 | | 6760 | 8665 | | | | |
| 281 | FAHFA(16:0-O-20:0) | PAHAA | | | 12019 | | | | | 21282 | 10952 | | 4768 | | | | |
| 282 | FAHFA(18:0-O-20:0) | SAHAA | | | | | | 9269 | | | 9560 | | | | | | |
| 283 | FAHFA(18:1-O-20:0) | OAHAA | | 5760 | 28322 | | | 13638 | | 29857 | 3119 | 151913 | | | | | 4954 |
| 284 | FAHFA(18:2-O-20:0) | LAHAA | | | | 17302 | | | 110747 | 79566 | | | | | 83910 | | |
| 285 | FAHFA(18:3-O-20:0) | ALAHAA | | | | | | | | 18055 | | | | | | | |
| 286 | FAHFA(20:0-O-20:0) | AAHAA | 55974 | | | | | | | | | | | | | | |
| 287 | FAHFA(20:1-O-20:0) | EAHAA | | | | | | 4455 | | | 1670 | 80705 | | | | | 3743 |
| 288 | FAHFA(20:2-O-20:0) | EDAHAA | | | | | | 6215 | | | | 32387 | | | | | 4249 |
| 289 | FAHFA(20:3-O-20:0) | ETAHAA | | | | | | 25019 | | | | 18312 | | | | | |
| 290 | FAHFA(22:1-O-20:0) | DEAHAA | | | | | | | | | | 7705 | | | | | |
| 291 | FAHFA(15:0-O-21:0) | PDAHHEA | 13670 | | | | | | | | | | | | | | |
| 292 | FAHFA(16:1-O-21:0) | POHHEA | | | | | | | | 10123 | | | | | | | |
| 293 | FAHFA(16:0-O-21:0) | PAHHEA | | 8474 | 4216 | 6996 | 3672 | | | | 5121 | 9683 | | | 53476 | | |
| 294 | FAHFA(18:0-O-21:0) | SAHHEA | | | | | | | | | 1439 | | | | | | |
| 295 | FAHFA(18:1-O-21:0) | OAHHEA | | | | | 2112 | | 14024 | 5005 | | | | | 47197 | | |
| 296 | FAHFA(18:2-O-21:0) | LAHHEA | | | | 47156 | 19352 | | 13953 | 23079 | | | | | 116310 | | |
| 297 | FAHFA(18:3-O-21:0) | ALAHHEA | | | | | | | | 4841 | | | | | | | |
| 298 | FAHFA(21:0-O-21:0) | AAHHEA | 7528 | | | | | | | | | | | | | | |

Highlighted in orange were first reported
