# Supplementary material for: A dataset of branched fatty acid esters of hydroxy fatty acids diversity in foods
Source: Sci Data. 2023 Nov 10;10:790. doi: 10.1038/s41597-023-02712-z (PMC10638281; doi:10.1038/s41597-023-02712-z)
Supplement: Supplementary file 6 — Supplementary information-7 Table S7. MS intensity of FAHFA regioisomers [file 41597_2023_2712_MOESM6_ESM.pdf]

Supplementary Table S7. MS intensity of FAHFA regioisomers in 12 food samples and 4 medicinal food samples

Spirulina

NO.	Normal name	FAHFA ID	*m/z* (DMED labeling)	*m/z* (d_4-DMED labeling)	Product ion	Regioisomer number	*I1*	*I2*	*I3*	*I4*	*I5*	*I6*	*I7*	*I8*	*I9*	*I10*	*I11*	*I12*
1	FAHFA(15:0-O-16:1)	PDAHPO	565.5	569.5	278.2	2	18488	3236										
2	FAHFA(14:0-O-18:1)	MAHOA	579.5	583.5	306.2	2	6207	9130										
3	FAHFA(15:0-O-18:1)	PDAHOA	593.5	597.5	306.2	1	110635											
4	FAHFA(18:1-O-18:1)	OAHOA	633.5	637.5	306.2	1	5111											
5	FAHFA(20:0-O-18:1)	AAHOA	663.5	667.5	306.2	2	10514	7388										
6	FAHFA(18:1-O-20:1)	OAHEA	661.5	666.5	334.2	1	35155											
7	FAHFA(18:3-O-20:1)	ALAHEA	637.5	641.5	334.2	3	18700	14683	16225									
8	FAHFA(20:0-O-20:1)	AAHEA	691.5	695.5	334.2	2	292149	42118										
9	FAHFA(20:0-O-20:2)	AAHEDA	689.5	693.5	332.2	1	9928											
10	FAHFA(15:0-O-22:6)	PDAHDHA	639.5	643.5	352.2	3	6825	32968	6304									
11	FAHFA(18:3-O-22:6)	ALAHDHA	675.5	679.5	352.2	1	5088											
12	FAHFA(14:0-O-12:0)	MAHDA	497.5	501.5	224.2	3	1765	7295	3993									
13	FAHFA(16:1-O-12:0)	POHDA	523.5	527.5	224.2	2	5100	9889										
14	FAHFA(16:0-O-12:0)	PAHDA	525.5	529.5	224.2	2	9432	68313										
15	FAHFA(17:0-O-12:0)	HDAHDA	539.5	543.5	224.2	2	1726	3176										
16	FAHFA(18:0-O-12:0)	SAHDA	553.5	557.5	224.2	4	2713	2040	2676	8093								
17	FAHFA(18:1-O-12:0)	OAHDA	551.5	556.5	224.2	2	89955	3107										
18	FAHFA(14:0-O-14:0)	MAHMA	525.5	529.5	252.2	4	1918	4556	20881	18222								
19	FAHFA(15:0-O-14:0)	PDAHMA	539.5	543.5	252.2	2	9482	17355										
20	FAHFA(16:1-O-14:0)	POHMA	551.5	555.5	252.2	2	1691	2057										
21	FAHFA(16:0-O-14:0)	PAHMA	553.5	557.5	252.2	5	5609	5594	35721	3785	27805							
22	FAHFA(18:0-O-14:0)	SAHMA	581.5	585.5	252.2	3	8080	12318	8383									
23	FAHFA(18:1-O-14:0)	OAHMA	579.5	583.5	252.2	1	6459											
24	FAHFA(18:2-O-14:0)	LAHMA	577.5	581.5	252.2	1	12959											
25	FAHFA(14:0-O-15:0)	MAHPDA	539.5	543.5	266.2	3	78663	49619	4885									
26	FAHFA(15:0-O-15:0)	PDAHPDA	553.5	557.5	266.2	2	1472356	72921										
27	FAHFA(15:1-O-15:0)	PDEAHPDA	551.5	555.5	266.2	3	6535	4843	2058									
28	FAHFA(16:1-O-15:0)	POHPDA	565.5	569.5	266.2	1	39147											
29	FAHFA(16:0-O-15:0)	PAHPDA	567.5	571.5	266.2	5	6620	5083	96563	73845	12318							
30	FAHFA(17:0-O-15:0)	HDAHPDA	581.5	585.5	266.2	2	10093	9004										
31	FAHFA(17:1-O-15:0)	HDEAHPDA	579.5	583.5	266.2	1	4535											
32	FAHFA(18:1-O-15:0)	OAHPDA	593.5	597.5	266.2	1	4201											
33	FAHFA(14:0-O-16:0)	MAHPA	553.6	557.6	280.3	3	285963	124402	125804									
34	FAHFA(15:0-O-16:0)	PDAHPA	567.6	571.6	280.3	2	3662061	1092302										
35	FAHFA(15:1-O-16:0)	PDEAHPA	565.6	569.6	280.3	4	60754	11617	52036	61438								
36	FAHFA(16:1-O-16:0)	POHPA	579.6	583.6	280.3	1	305647											
37	FAHFA(16:0-O-16:0)	PAHPA	581.6	585.6	280.3	3	280769	204345	1726649									
38	FAHFA(17:0-O-16:0)	HDAHPA	595.6	599.6	280.3	5	5410	62065	12708	23860	68511							
39	FAHFA(17:1-O-16:0)	HDEAHPA	593.6	597.6	280.3	2	35444	11353										
40	FAHFA(18:0-O-16:0)	SAHPA	609.6	613.6	280.3	9	10186	23300	11716	9777	12450	18315	6028	18650	5645			
41	FAHFA(18:1-O-16:0)	OAHPA	607.6	611.6	280.3	2	4143	12435										
42	FAHFA(18:2-O-16:0)	LAHPA	605.6	609.6	280.3	1	7572											
43	FAHFA(18:3-O-16:0)	ALAHPA	603.6	607.6	280.3	1	16474											
44	FAHFA(14:0-O-17:0)	MAHHDA	567.6	571.6	294.3	2	123162	190301										
45	FAHFA(15:0-O-17:0)	PDAHHDA	581.6	585.6	294.3	2	9768417	269567										
46	FAHFA(15:1-O-17:0)	PDEAHHDA	579.6	583.6	294.3	2	48881	303766										
47	FAHFA(16:0-O-17:0)	PAHHDA	581.6	585.6	294.3	2	9694128	257596										
48	FAHFA(17:0-O-17:0)	HDAHHDA	609.6	613.6	294.3	3	26679	137570	6742									
49	FAHFA(18:1-O-17:0)	OAHHDA	621.6	625.6	294.3	1	9200											
50	FAHFA(18:3-O-17:0)	ALAHHDA	617.6	621.6	294.3	1	18752											
51	FAHFA(20:0-O-17:0)	AAHHDA	651.6	655.6	294.3	1	15915											
52	FAHFA(14:0-O-18:0)	MAHSA	581.8	585.8	308.3	2	7432	14819										
53	FAHFA(15:0-O-18:0)	PDAHSA	595.8	599.8	308.3	2	132376	72063										
54	FAHFA(16:0-O-18:0)	PAHSA	609.8	613.8	308.3	11	18166	10743	15625	13271	6514	15155	19667	4149	7228	5666	38881	4920
55	FAHFA(18:0-O-18:0)	SAHSA	637.8	641.8	308.3	11	28187	18502	36960	39162	15553	31978	35576	6492	3706	3336	7294	
56	FAHFA(18:1-O-18:0)	OAHSA	635.8	639.8	308.3	1	44142											
57	FAHFA(20:0-O-18:0)	AAHSA	665.8	669.8	308.3	1	128777											
58	FAHFA(15:0-O-19:0)	PDAHNDA	609.8	613.8	322.3	1	17800											
59	FAHFA(18:0-O-19:0)	SAHNDA	651.8	655.8	322.3	1	6808											
60	FAHFA(20:0-O-19:0)	AAHNDA	679.8	683.8	322.3	1	8815											
61	FAHFA(15:0-O-20:0)	PDAHAA	623.8	627.8	336.3	2	22115	2202										
62	FAHFA(20:0-O-20:0)	AAHAA	693.8	697.8	336.3	2	14877	41097										
63	FAHFA(15:0-O-21:0)	PDAHHEA	637.8	641.8	350.3	2	8036	5634										
64	FAHFA(21:0-O-21:0)	AAHHEA	707.8	713.8	350.3	1	7528											

I, intensity (cps)

Nostoc commune Vauch

NO.	Normal name	FAHFA ID	*m/z* (DMED labeling)	*m/z* (d_4-DMED labeling)	Product ion	Regioisomer number	*I1*	*I2*	*I3*	*I4*	*I5*	*I6*	*I7*	*I8*	*I9*	*I10*	*I11*	*I12*
1	FAHFA(16:1-O-16:1)	POHPO	577.5	581.5	278.2	2	13557	10540										
2	FAHFA(16:0-O-16:1)	PAHPO	579.5	583.5	278.2	4	9605	47740	22340	7331								
3	FAHFA(18:0-O-16:1)	SAHPO	607.5	611.5	278.2	2	2756	8296										
4	FAHFA(18:2-O-16:1)	LAHPO	603.5	607.5	278.2	2	12648	8994										
5	FAHFA(18:3-O-16:1)	ALAHPO	601.5	605.5	278.2	2	47377	27110										
6	FAHFA(16:0-O-18:1)	PAHOA	607.5	611.5	306.2	4	7765	15550	12792	25725								
7	FAHFA(18:0-O-18:1)	SAHOA	635.5	639.5	306.2	3	6476	3408	5349									
8	FAHFA(18:1-O-18:1)	OAHOA	633.5	637.5	306.2	1	256025											
9	FAHFA(16:0-O-18:2)	PAHLA	605.5	609.5	304.2	6	6266	5913	13599	19588	3898	7287						
10	FAHFA(16:1-O-18:3)	POHALA	601.5	605.5	302.2	1	8470											
11	FAHFA(16:0-O-18:3)	PAHALA	603.5	607.5	302.2	5	20555	12627	34978	13723	3401							
12	FAHFA(18:0-O-18:3)	SAHALA	631.5	635.5	302.2	4	8658	3470	3044	4898								
13	FAHFA(18:2-O-18:3)	LAHALA	627.5	631.5	302.2	2	3202	14671										
14	FAHFA(18:1-O-20:1)	OAHEA	661.5	665.5	334.2	1	36028											
15	FAHFA(20:0-O-20:1)	AAHEA	691.5	695.5	334.2	1	6054											
16	FAHFA(16:1-O-12:0)	POHDA	523.5	527.5	224.2	3	1252	5422	2922									
17	FAHFA(16:0-O-12:0)	PAHDA	525.5	529.5	224.2	4	12743	12015	4022	4812								
18	FAHFA(18:0-O-12:0)	SAHDA	553.5	557.5	224.2	4	31135	5795	4040	4543								
19	FAHFA(14:0-O-14:0)	MAHMA	525.5	529.5	224.2	3	5751	18445	21685									
20	FAHFA(15:0-O-14:0)	PDAHMA	539.5	543.5	224.2	2	3723	33156										
21	FAHFA(15:1-O-14:0)	PDEAHMA	537.5	541.5	224.2	1	5355											
22	FAHFA(16:1-O-14:0)	POHMA	551.5	555.5	224.2	3	2446	2115	3482									
23	FAHFA(16:0-O-14:0)	PAHMA	553.5	557.5	224.2	6	8336	5949	5639	31574	21726	2167						
24	FAHFA(18:1-O-14:0)	OAHMA	579.5	583.5	224.2	2	1567	7519										
25	FAHFA(18:3-O-14:0)	ALAHMA	575.5	579.5	224.2	2	6602	8413										
26	FAHFA(14:0-O-15:0)	MAHPDA	539.5	543.5	266.2	2	23900	15523										
27	FAHFA(15:0-O-15:0)	PDAHPDA	553.5	557.5	266.2	1	7019											
28	FAHFA(16:0-O-15:0)	PAHPDA	567.5	571.5	266.2	5	14029	10829	14839	15615	14086							
29	FAHFA(18:0-O-15:0)	SAHPDA	595.5	599.5	266.2	4	12399	7504	4471	3697								
30	FAHFA(14:0-O-16:0)	MAHPA	553.6	557.6	280.3	2	12294	30089										
31	FAHFA(15:0-O-16:0)	PDAHPA	567.6	571.6	280.3	3	79481	15744	10722									
32	FAHFA(16:1-O-16:0)	POHPA	579.6	583.6	280.3	3	7993	5531	7734									
33	FAHFA(16:0-O-16:0)	PAHPA	581.6	585.6	280.3	12	50649	30072	15122	15214	13303	21167	15523	22653	1087	7395	62913	15877
34	FAHFA(18:0-O-16:0)	SAHPA	609.6	613.6	280.3	10	19815	34486	17622	9589	17955	17118	23419	5612	6363	19348		
35	FAHFA(18:1-O-16:0)	OAHPA	607.6	611.6	280.3	1	24547											
36	FAHFA(18:3-O-16:0)	ALAHPA	603.6	607.6	280.3	4	9154	6790	12367	6161								
37	FAHFA(20:0-O-16:0)	AAHPA	637.6	641.6	280.3	1	13262											
38	FAHFA(22:6-O-16:0)	DHAHPA	653.6	657.6	280.3	1	1806											
39	FAHFA(14:0-O-17:0)	MAHHDA	567.6	571.6	294.3	1	5235											
40	FAHFA(15:0-O-17:0)	PDAHHDA	581.6	585.6	294.3	1	159716											
41	FAHFA(16:0-O-17:0)	PAHHDA	581.6	585.6	294.3	1	157943											
42	FAHFA(16:0-O-18:0)	PAHSA	609.8	613.8	308.3	9	29683	17599	22703	25355	14766	32366	63400	6310	21149			
43	FAHFA(18:0-O-18:0)	SAHSA	637.8	641.8	308.3	9	40490	26504	53418	52825	23700	49846	76588	8409	6842			
44	FAHFA(18:1-O-18:0)	OAHSA	635.8	639.8	308.3	2	7972	28873										
45	FAHFA(16:1-O-19:0)	POHNDA	621.8	625.8	322.3	1	5085											
46	FAHFA(16:0-O-19:0)	PAHNDA	623.8	627.8	322.3	1	35606											
47	FAHFA(18:0-O-19:0)	SAHNDA	651.8	655.8	322.3	1	11174											
48	FAHFA(18:1-O-19:0)	OAHNDA	649.8	653.8	322.3	1	9444											
49	FAHFA(14:0-O-20:0)	MAHAA	609.8	613.8	336.3	1	7983											
50	FAHFA(18:1-O-20:0)	OAHAA	663.8	667.8	336.3	2	3612	2148										
51	FAHFA(16:0-O-21:0)	PAHHEA	651.8	655.8	350.3	1	8474											

I, intensity (cps)

Kelp (*Laminaria japonica*)

NO.	Normal name	FAHFA ID	*m/z* (DMED labeling)	*m/z* (d_4-DMED labeling)	Product ion	Regioisomer number	*I1*	*I2*	*I3*	*I4*	*I5*	*I6*	*I7*	*I8*	*I9*	*I10*
1	FAHFA(14:1-O-14:1)	MOHMO	521.5	525.5	250.2	2	3661	8913								
2	FAHFA(16:1-O-14:1)	POHMO	549.5	553.5	250.2	2	3146	6928								
3	FAHFA(18:2-O-14:1)	LAHMO	575.5	579.5	250.2	2	4128	14855								
4	FAHFA(18:3-O-14:1)	ALAHMO	573.5	577.5	250.2	2	3519	7879								
5	FAHFA(18:1-O-16:1)	OAHPO	605.5	609.5	278.2	2	10707	6411								
6	FAHFA(16:1-O-16:2)	POHHDA	575.5	579.5	276.2	2	2713	8513								
7	FAHFA(16:0-O-16:2)	PAHHDA	577.5	581.5	276.2	2	3500	6287								
8	FAHFA(18:2-O-16:2)	LAHHDA	601.5	605.5	276.2	2	5370	10180								
9	FAHFA(14:0-O-18:1)	MAHOA	579.5	583.5	306.2	5	6014	53699	33024	40740	13109					
10	FAHFA(15:0-O-18:1)	PDAHOA	593.5	597.5	306.2	1	2822									
11	FAHFA(16:0-O-18:1)	PAHOA	607.5	611.5	306.2	3	304455	108069	154304							
12	FAHFA(17:0-O-18:1)	HDAHOA	621.5	625.5	306.2	1	7131									
13	FAHFA(18:0-O-18:1)	SAHOA	635.5	639.5	306.2	2	89666	20253								
14	FAHFA(18:1-O-18:1)	OAHOA	633.5	637.5	306.2	5	8752	229372	10210	49738	71666					
15	FAHFA(18:2-O-18:1)	LAHOA	631.5	635.5	306.2	1	71684									
16	FAHFA(20:3-O-18:1)	ETAHOA	657.5	661.5	306.2	1	11599									
17	FAHFA(14:0-O-18:2)	MAHLA	577.5	581.5	304.2	6	13995	4066	22506	19165	21658	4788				
18	FAHFA(16:0-O-18:2)	PAHLA	605.5	609.5	304.2	6	27337	27531	25933	65596	70588	6027				
19	FAHFA(18:0-O-18:2)	SAHLA	633.5	637.5	304.2	6	5777	10376	6335	10802	6175	24247				
20	FAHFA(18:1-O-18:2)	OAHLA	631.5	635.5	304.2	4	18206	17988	48766	57449						
21	FAHFA(18:2-O-18:2)	LAHLA	629.5	633.5	304.2	4	7519	6770	17115	12799						
22	FAHFA(20:4-O-18:2)	ARAHLA	653.5	657.5	304.2	2	29311	18055								
23	FAHFA(20:5-O-18:2)	EPAHLA	651.5	655.5	304.2	2	24340	15483								
24	FAHFA(14:0-O-18:3)	MAHALA	575.5	579.5	304.2	2	14333	75508								
25	FAHFA(16:0-O-18:3)	PAHALA	603.5	607.5	302.2	2	22544	201307								
26	FAHFA(18:0-O-18:3)	SAHALA	631.5	635.5	302.2	1	42208									
27	FAHFA(18:1-O-18:3)	OAHALA	629.5	633.5	302.2	1	93641									
28	FAHFA(18:2-O-18:3)	LAHALA	627.5	631.5	302.2	2	14177	46192								
29	FAHFA(20:0-O-18:3)	AAHALA	659.5	663.5	302.2	1	20402									
30	FAHFA(14:0-O-20:1)	MAHEA	607.5	611.5	334.2	1	7134									
31	FAHFA(14:1-O-20:1)	MOHEA	605.5	609.5	334.2	2	9242	18726								
32	FAHFA(16:1-O-20:1)	POHEA	633.5	637.5	334.2	2	2783	7569								
33	FAHFA(16:0-O-20:1)	PAHEA	635.5	639.5	334.2	1	22818									
34	FAHFA(18:2-O-20:1)	LAHEA	659.5	663.5	334.2	3	14809	8166	13059							
35	FAHFA(20:5-O-20:1)	EPAHEA	681.5	685.5	334.2	2	11771	22104								
36	FAHFA(14:0-O-20:2)	MAHEDA	605.5	609.5	332.2	6	7104	11150	6868	4728	4304	3415				
37	FAHFA(18:1-O-20:2)	OAHEDA	659.5	663.5	332.2	4	10230	6701	6936	4005						
38	FAHFA(20:4-O-20:2)	ARAHEDA	681.5	685.5	332.2	4	7868	15040	6098	6908						
39	FAHFA(20:5-O-20:2)	EPAHEDA	679.5	683.5	332.2	3	7754	14772	5388							
40	FAHFA(14:0-O-20:4)	MAHARA	601.5	605.5	328.2	1	27704									
41	FAHFA(15:0-O-20:4)	PDAHARA	615.3	619.3	328.2	1	4125									
42	FAHFA(16:1-O-20:4)	POHARA	627.5	631.5	328.2	1	10904									
43	FAHFA(16:0-O-20:4)	PAHARA	629.5	633.5	328.2	5	9907	15070	34994	132355	20906					
44	FAHFA(18:1-O-20:4)	OAHARA	655.5	659.5	328.2	1	84235									
45	FAHFA(18:2-O-20:4)	LAHARA	653.5	657.5	328.2	1	22801									
46	FAHFA(18:4-O-20:4)	SDAHARA	649.5	653.5	328.2	1	33541									
47	FAHFA(20:5-O-20:4)	EPAHARA	675.5	679.5	328.2	1	100295									
48	FAHFA(18:2-O-12:0)	LAHDA	549.5	553.5	224.2	1	8432									
49	FAHFA(20:0-O-12:0)	AAHDA	581.5	585.5	224.2	1	18945									
50	FAHFA(20:5-O-12:0)	EPAHDA	571.5	575.5	224.2	1	12609									
51	FAHFA(14:0-O-14:0)	MAHMA	525.5	529.5	252.2	6	4001	9913	1884	2301	4928	15181				
52	FAHFA(16:0-O-14:0)	PAHMA	553.5	557.5	252.2	8	4788	22254	7174	8335	4437	5555	25408	63758		
53	FAHFA(18:1-O-14:0)	OAHMA	579.5	583.5	252.2	6	6981	4024	2256	2444	8323	47177				
54	FAHFA(18:2-O-14:0)	LAHMA	577.5	581.5	252.2	1	26645									
55	FAHFA(18:3-O-14:0)	ALAHMA	575.5	579.5	252.2	1	33121									
56	FAHFA(18:4-O-14:0)	SDAHMA	573.5	577.5	252.2	1	13634									
57	FAHFA(20:5-O-14:0)	EPAHMA	599.5	603.5	252.2	1	9174									
58	FAHFA(20:5-O-15:0)	EPAHPDA	613.5	617.5	266.2	1	20674									
59	FAHFA(14:0-O-16:0)	MAHPA	553.6	557.6	280.3	9	4213	6824	4804	2193	1374	3370	1397	4565	18080	
60	FAHFA(15:0-O-16:0)	PDAHPA	567.6	571.6	280.3	1	5699									
61	FAHFA(16:0-O-16:0)	PAHPA	581.6	585.6	280.3	10	23132	54135	36880	33010	30554	22684	23349	8816	31486	38642
62	FAHFA(18:0-O-16:0)	SAHPA	609.6	613.6	280.3	9	11811	21774	23815	12480	6646	9227	8881	2771	19800	
63	FAHFA(18:1-O-16:0)	OAHPA	607.6	611.6	280.3	8	39017	24158	15193	14387	8983	13149	4444	13709		
64	FAHFA(18:2-O-16:0)	LAHPA	605.6	609.6	280.3	9	5650	2708	13477	14103	5353	5051	2994	4168	15764	
65	FAHFA(18:3-O-16:0)	ALAHPA	603.6	607.6	280.3	1	35411									
66	FAHFA(20:4-O-16:0)	ARAHPA	629.6	633.6	280.3	1	28716									
67	FAHFA(20:5-O-16:0)	EPAHPA	627.6	631.6	280.3	1	14865									
68	FAHFA(14:0-O-18:0)	MAHSA	581.8	585.8	308.3	1	11895									
69	FAHFA(16:0-O-18:0)	PAHSA	609.8	613.8	308.3	6	8319	8594	4211	3782	6850	35833				
70	FAHFA(18:0-O-18:0)	SAHSA	637.8	641.8	308.3	8	3495	8957	5847	9038	4697	2920	7066	23833		
71	FAHFA(18:1-O-18:0)	OAHSA	635.8	639.8	308.3	2	8433	51956								
72	FAHFA(18:2-O-18:0)	LAHSA	633.8	637.8	308.3	2	14688	15863								
73	FAHFA(20:5-O-18:0)	EPAHSA	655.8	659.8	308.3	1	9222									
74	FAHFA(16:0-O-20:0)	PAHAA	637.8	641.8	336.3	1	12019									
75	FAHFA(18:1-O-20:0)	OAHAA	663.8	667.8	336.3	1	28322									
76	FAHFA(16:0-O-21:0)	PAHHEA	651.8	655.8	350.3	1	4216									

I, intensity (cps)

Lentinus edodes

NO.	Normal name	FAHFA ID	*m/z* (DMED labeling)	*m/z* (d_4-DMED labeling)	Product ion	Regioisomer number	*I1*	*I2*	*I3*	*I4*	*I5*	*I6*	*I7*	*I8*	*I9*	*I10*	*I11*
1	FAHFA(18:2-O-14:1)	LAHMO	575.5	579.5	250.2	3	2082	4885	4088								
2	FAHFA(18:2-O-14:2)	LAHTDA	573.5	577.5	248.2	3	3015	2750	5617								
3	FAHFA(20:0-O-14:2)	AAHTDA	605.5	609.5	248.2	2	1390	1316									
4	FAHFA(22:2-O-14:2)	DDAHTDA	629.5	633.5	248.2	3	5053	11241	9516								
5	FAHFA(18:2-O-16:1)	LAHPO	603.5	607.5	278.2	6	8046	3232	6701	9305	4720	13929					
6	FAHFA(20:2-O-16:1)	EDAHPO	631.5	635.5	278.2	1	2525										
7	FAHFA(18:2-O-16:2)	LAHHDA	601.5	605.5	276.2	7	2456	5422	5399	6879	12424	3995	2332				
8	FAHFA(20:2-O-16:2)	EDAHHDA	629.5	633.5	276.2	3	3166	1484	819								
9	FAHFA(20:5-O-16:2)	EPAHHDA	623.5	627.5	276.2	2	3148	2495									
10	FAHFA(22:6-O-16:3)	DHAHHTA	647.5	651.5	274.2	2	2160	1997									
11	FAHFA(14:1-O-18:1)	MOHOA	577.5	581.5	306.2	2	77792	20773									
12	FAHFA(15:1-O-18:1)	PDEAHOA	591.5	595.5	306.2	2	140565	24443									
13	FAHFA(16:1-O-18:1)	POHOA	605.5	609.5	306.2	2	196604	35595									
14	FAHFA(16:0-O-18:1)	PAHOA	607.5	611.5	306.2	6	9188	20242	10401	15707	12856	17852					
15	FAHFA(18:1-O-18:1)	OAHOA	633.5	637.5	306.2	3	13247	17180	19968								
16	FAHFA(18:2-O-18:1)	LAHOA	631.5	635.5	306.2	6	125506	324123	236240	107018	46702	557809					
17	FAHFA(20:3-O-18:1)	ETAHOA	689.5	693.5	306.2	1	6665										
18	FAHFA(14:0-O-18:2)	MAHLA	577.5	581.5	304.2	3	11118	10715	4555								
19	FAHFA(15:0-O-18:2)	PDAHLA	591.5	595.5	304.2	3	23598	15998	6504								
20	FAHFA(16:1-O-18:2)	POHLA	603.5	607.5	304.2	3	11460	12240	4633								
21	FAHFA(16:0-O-18:2)	PAHLA	605.5	609.5	304.2	4	12833	292200	158370	65716							
22	FAHFA(18:1-O-18:2)	OAHLA	631.5	635.5	304.2	6	79927	73070	8439	104806	48029	11130					
23	FAHFA(18:2-O-18:2)	LAHLA	629.5	633.5	304.2	5	229395	2926945	2159773	311275	237460						
24	FAHFA(20:0-O-18:2)	AAHLA	661.5	665.5	304.2	2	49187	54127									
25	FAHFA(20:2-O-18:2)	EDAHLA	657.5	661.5	304.2	1	13319										
26	FAHFA(18:1-O-18:3)	OAHALA	629.5	633.5	302.2	2	6169	4543									
27	FAHFA(14:1-O-20:1)	MOHEA	605.5	609.5	334.2	1	1603										
28	FAHFA(18:2-O-20:1)	LAHEA	659.5	663.5	334.2	2	19204	22859									
29	FAHFA(16:0-O-20:2)	PAHEDA	633.5	637.5	332.2	1	2578										
30	FAHFA(18:2-O-20:2)	LAHEDA	657.5	661.5	332.2	1	67819										
31	FAHFA(20:3-O-20:2)	ETAHEDA	683.5	687.5	332.2	1	10418										
32	FAHFA(16:0-O-12:0)	PAHDA	525.5	529.5	224.2	2	2512	4464									
33	FAHFA(18:2-O-12:0)	LAHDA	549.5	553.5	224.2	3	5206	8067	30885								
34	FAHFA(20:2-O-12:0)	EDAHDA	577.5	581.5	224.2	1	4597										
35	FAHFA(22:0-O-12:0)	BAHDA	609.5	613.5	224.2	2	2485	1472									
36	FAHFA(14:0-O-14:0)	MAHMA	525.5	529.5	252.2	2	5890	3238									
37	FAHFA(15:0-O-14:0)	PDAHMA	539.5	543.5	252.2	1	2223										
38	FAHFA(16:1-O-14:0)	POHMA	551.5	555.5	252.2	1	2915										
39	FAHFA(16:0-O-14:0)	PAHMA	553.5	557.5	252.2	3	6163	38587	35000								
40	FAHFA(18:1-O-14:0)	OAHMA	579.5	583.5	252.2	2	28548	9538									
41	FAHFA(18:2-O-14:0)	LAHMA	577.5	581.5	252.2	1	1009335										
42	FAHFA(18:3-O-14:0)	ALAHMA	575.5	579.5	252.2	1	7532										
43	FAHFA(20:2-O-14:0)	EDAHMA	605.5	609.5	252.2	1	16044										
44	FAHFA(14:0-O-15:0)	MAHPDA	539.5	543.5	266.2	1	5434										
45	FAHFA(16:1-O-15:0)	POHPDA	565.5	569.5	266.2	1	3001										
46	FAHFA(16:0-O-15:0)	PAHPDA	567.5	571.5	266.2	1	140554										
47	FAHFA(18:1-O-15:0)	OAHPDA	593.5	597.5	266.2	2	34153	46702									
48	FAHFA(18:2-O-15:0)	LAHPDA	591.5	595.5	266.2	1	1290657										
49	FAHFA(18:3-O-15:0)	ALAHPDA	589.5	593.5	266.2	3	3568	3394	16761								
50	FAHFA(14:0-O-16:0)	MAHPA	553.6	557.6	280.3	2	3311	6076									
51	FAHFA(15:0-O-16:0)	PDAHPA	567.6	571.6	280.3	2	6315	21443									
52	FAHFA(16:1-O-16:0)	POHPA	579.6	583.6	280.3	1	7444										
53	FAHFA(16:0-O-16:0)	PAHPA	581.6	585.6	280.3	2	68033	106362									
54	FAHFA(18:1-O-16:0)	OAHPA	607.6	611.6	280.3	4	22290	26105	90823	28355							
55	FAHFA(18:2-O-16:0)	LAHPA	605.6	609.6	280.3	1	3829811										
56	FAHFA(18:3-O-16:0)	ALAHPA	603.6	607.6	280.3	6	3840	8502	9945	6508	5582	32463					
57	FAHFA(17:0-O-17:0)	HDAHHDA	609.6	613.6	294.3	2	1590	2799									
58	FAHFA(18:0-O-17:0)	SAHHDA	623.6	627.6	294.3	2	22406	23375									
59	FAHFA(18:2-O-17:0)	LAHHDA	619.6	623.6	294.3	9	7101	29119	39236	13967	17104	7459	15430	8767	8668		
60	FAHFA(20:1-O-17:0)	EAHHDA	649.6	653.6	294.3	4	4835	4739	4178	4374							
61	FAHFA(20:2-O-17:0)	EDAHHDA	647.6	651.6	294.3	2	52857	42401									
62	FAHFA(15:1-O-18:0)	PDEAHSA	593.8	597.8	308.3	2	1659	5558									
63	FAHFA(16:1-O-18:0)	POHSA	607.8	611.8	308.3	2	7610	23553									
64	FAHFA(18:2-O-18:0)	LAHSA	633.8	637.8	308.3	11	56738	58753	11632	5198	3903	37996	4602	5587	15218	14913	28831
65	FAHFA(18:3-O-18:0)	ALAHSA	631.8	635.8	308.3	2	5327	11642									
66	FAHFA(14:0-O-19:0)	MAHNDA	595.8	599.8	322.3	2	93353	57959									
67	FAHFA(15:0-O-19:0)	PDAHNDA	609.8	613.8	322.3	2	290256	362732									
68	FAHFA(15:1-O-19:0)	PDEAHNDA	607.8	611.8	322.3	1	6810										
69	FAHFA(16:1-O-19:0)	POHNDA	621.8	625.8	322.3	2	426112	332695									
70	FAHFA(16:0-O-19:0)	PAHNDA	623.8	627.8	322.3	2	7167243	6060658									
71	FAHFA(17:0-O-19:0)	HDAHNDA	637.8	641.8	322.3	2	47385	46733									
72	FAHFA(17:1-O-19:0)	HDEAHNDA	635.8	639.8	322.3	2	23008	38933									
73	FAHFA(18:1-O-19:0)	OAHNDA	649.8	653.8	322.3	4	965775	667151	2112222	1915652							
74	FAHFA(18:2-O-19:0)	LAHNDA	647.8	651.8	322.3	4	1258970	1363782	20000000	20000000							
75	FAHFA(18:3-O-19:0)	ALAHNDA	645.8	649.8	322.3	2	320942	303722									
76	FAHFA(20:0-O-19:0)	AAHNDA	679.8	683.8	322.3	4	5456	7554	4822	10189							
77	FAHFA(20:1-O-19:0)	EAHNDA	677.8	681.8	322.3	2	14993	13490									
78	FAHFA(20:2-O-19:0)	EDAHNDA	675.8	679.8	322.3	4	38639	37495	53666	48884							
79	FAHFA(20:3-O-19:0)	ETAHNDA	673.8	677.8	322.3	1	10387										
80	FAHFA(22:0-O-19:0)	BAHNDA	707.8	711.8	322.3	2	11452	5654									
81	FAHFA(16:1-O-20:0)	POHAA	635.8	639.8	336.3	1	5394										
82	FAHFA(18:2-O-20:0)	LAHAA	661.8	665.8	336.3	2	6582	10720									
83	FAHFA(16:0-O-21:0)	PAHHEA	651.8	655.8	350.3	2	3464	3532									
84	FAHFA(18:2-O-21:0)	LAHHEA	675.8	679.8	350.3	2	27022	20134									

I, intensity (cps)

Lotus Plumule (*Plumula Nelumbinis*)

NO.	Normal name	FAHFA ID	*m/z* (DMED labeling)	*m/z* (d_4-DMED labeling)	Product ion	Regioisomer number	*I1*	*I2*	*I3*	*I4*	*I5*	*I6*	*I7*	*I8*
1	FAHFA(16:0-O-14:1)	PAHMO	551.5	555.5	250.2	6	1098	948	3232	3251	11595	2787		
2	FAHFA(18:1-O-14:1)	OAHMO	577.5	581.5	250.2	4	2643	4577	14247	2524				
3	FAHFA(18:2-O-14:1)	LAHMO	575.5	579.5	250.2	5	16178	54669	3592	6138	22841			
4	FAHFA(18:3-O-14:1)	ALAHMO	573.5	577.5	250.2	3	4135	2881	27262					
5	FAHFA(16:0-O-14:2)	PAHTDA	549.5	553.5	248.2	1	4344							
6	FAHFA(18:1-O-14:2)	OAHTDA	575.5	579.5	248.2	1	4134							
7	FAHFA(18:2-O-14:2)	LAHTDA	573.5	577.5	248.2	1	30766							
8	FAHFA(22:2-O-14:2)	DDAHTDA	629.5	633.5	248.2	2	152532	1735						
9	FAHFA(22:6-O-14:2)	DHAHTDA	621.5	625.5	248.2	1	2791							
10	FAHFA(18:2-O-14:3)	LAHTTA	571.5	575.5	246.2	1	3213							
11	FAHFA(16:0-O-16:1)	PAHPO	579.5	583.5	278.2	2	45139	14735						
12	FAHFA(18:1-O-16:1)	OAHPO	605.5	609.5	278.2	1	104459							
13	FAHFA(18:2-O-16:1)	LAHPO	603.5	607.5	278.2	4	161126	19516	29561	9901				
14	FAHFA(18:3-O-16:1)	ALAHPO	601.5	605.5	278.2	1	17552							
15	FAHFA(16:0-O-16:2)	PAHHDA	577.5	581.5	276.2	2	3289	21082						
16	FAHFA(18:1-O-16:2)	OAHHDA	603.5	607.5	276.2	3	3516	2701	23970					
17	FAHFA(18:2-O-16:2)	LAHHDA	601.5	605.5	276.2	5	112299	2413	4290	3309	1737			
18	FAHFA(16:1-O-18:1)	POHOA	605.5	609.5	306.2	5	1739	7021	3290	11760	5087			
19	FAHFA(16:0-O-18:1)	PAHOA	607.5	611.5	306.2	5	38072	10412	16284	7318	21316			
20	FAHFA(18:1-O-18:1)	OAHOA	633.5	637.5	306.2	1	51480							
21	FAHFA(18:2-O-18:1)	LAHOA	631.5	635.5	306.2	7	321507	118884	78122	37411	56448	33059	11889	
22	FAHFA(18:4-O-18:1)	SDAHOA	627.5	631.5	306.2	2	14193	20094						
23	FAHFA(14:0-O-18:2)	MAHLA	577.5	581.5	304.2	2	21662	3216						
24	FAHFA(16:1-O-18:2)	POHLA	603.5	607.5	304.2	1	13221							
25	FAHFA(16:0-O-18:2)	PAHLA	605.5	609.5	304.2	7	19624	5179	9259	8480	196774	91417	13147	
26	FAHFA(18:1-O-18:2)	OAHLA	631.5	635.5	304.2	5	101045	20984	163817	49919	5241			
27	FAHFA(18:2-O-18:2)	LAHLA	629.5	633.5	304.2	7	96269	40336	65973	3744663	505527	19367	85156	
28	FAHFA(22:6-O-18:2)	DHAHLA	677.5	681.5	304.2	6	4371	5616	20240	23681	3981	2634		
29	FAHFA(16:0-O-18:3)	PAHALA	603.5	607.5	302.2	3	33609	14934	1529					
30	FAHFA(18:1-O-18:3)	OAHALA	629.5	633.5	302.2	3	6102	24135	12939					
31	FAHFA(18:2-O-18:3)	LAHALA	627.5	631.5	302.2	4	230820	99467	5136	7148				
32	FAHFA(16:0-O-20:2)	PAHEDA	633.5	637.5	332.2	2	24990	30157						
33	FAHFA(18:1-O-20:2)	OAHEDA	659.5	663.5	332.2	4	10625	13189	34770	47258				
34	FAHFA(18:2-O-20:2)	LAHEDA	657.5	661.5	332.2	3	28105	219254	364970					
35	FAHFA(16:0-O-22:6)	PAHDHA	653.5	657.5	352.2	2	4028	3507						
36	FAHFA(16:0-O-20:4)	PAHARA	629.5	633.5	328.2	1	20329							
37	FAHFA(18:1-O-20:4)	OAHARA	655.5	659.5	328.2	1	12651							
38	FAHFA(18:2-O-20:4)	LAHARA	653.5	657.5	328.2	1	48622							
39	FAHFA(16:0-O-12:0)	PAHDA	525.5	529.5	224.2	1	14588							
40	FAHFA(18:1-O-12:0)	OAHDA	551.5	555.5	224.2	2	3259	14091						
41	FAHFA(18:2-O-12:0)	LAHDA	549.5	553.5	224.2	2	9929	109145						
42	FAHFA(18:3-O-12:0)	ALAHDA	547.5	551.5	224.2	1	16134							
43	FAHFA(16:0-O-14:0)	PAHMA	553.5	557.5	252.2	1	6818							
44	FAHFA(18:1-O-14:0)	OAHMA	579.5	583.5	252.2	1	4249							
45	FAHFA(18:2-O-14:0)	LAHMA	577.5	581.5	252.2	1	51189							
46	FAHFA(18:3-O-14:0)	ALAHMA	575.5	579.5	252.2	1	6586							
47	FAHFA(18:2-O-15:0)	LAHPDA	591.5	599.5	266.2	5	11242	26285	2829	7718	2534			
48	FAHFA(18:2-O-16:0)	LAHPA	605.6	609.6	280.3	8	24560	5738	4444	4860	12174	5740	6809	28193
49	FAHFA(18:0-O-17:0)	SAHHDA	623.6	627.6	294.3	4	7671	7003	1859	2171				
50	FAHFA(20:1-O-17:0)	EAHHDA	649.6	653.6	294.3	2	1909	2016						
51	FAHFA(20:2-O-17:0)	EDAHHDA	647.6	651.6	294.3	4	13476	12969	2874	2488				
52	FAHFA(16:1-O-18:0)	POHSA	607.8	611.8	308.3	1	3570							
53	FAHFA(18:3-O-18:0)	ALAHSA	631.8	635.8	308.3	2	3074	12637						
54	FAHFA(14:0-O-19:0)	MAHNDA	595.8	599.8	322.3	4	35680	38134	6237	5644				
55	FAHFA(15:0-O-19:0)	PDAHNDA	609.8	613.8	322.3	4	3316	5404	766	4045				
56	FAHFA(16:1-O-19:0)	POHNDA	621.8	625.8	322.3	4	46209	53147	9747	8397				
57	FAHFA(16:0-O-19:0)	PAHNDA	623.8	627.8	322.3	4	2392637	2312282	517260	351248				
58	FAHFA(17:0-O-19:0)	HDAHNDA	637.8	641.8	322.3	2	10102	12781						
59	FAHFA(17:1-O-19:0)	HDEAHNDA	635.8	639.8	322.3	3	3795	7948	10039					
60	FAHFA(18:1-O-19:0)	OAHNDA	649.8	653.8	322.3	8	344038	255555	54786	44783	1169717	1414300	312891	230161
61	FAHFA(18:2-O-19:0)	LAHNDA	647.8	651.8	322.3	4	9121615	9003835	1685771	1455749				
62	FAHFA(18:3-O-19:0)	ALAHNDA	645.8	679.8	322.3	4	891821	1021823	139277	125522				
63	FAHFA(20:0-O-19:0)	AAHNDA	679.8	683.8	322.3	4	32816	36170	4400	4892				
64	FAHFA(20:1-O-19:0)	EAHNDA	677.8	681.8	322.3	4	31389	32565	5659	3317				
65	FAHFA(20:2-O-19:0)	EDAHNDA	675.8	679.8	322.3	5	9801	6640	8103	10788	2019			
66	FAHFA(22:0-O-19:0)	BAHNDA	707.8	711.8	322.3	4	28295	34168	4436	3125				
67	FAHFA(22:2-O-19:0)	DDAHNDA	703.8	707.8	322.3	1	5437							
68	FAHFA(16:0-O-21:0)	PAHHEA	651.8	655.8	350.3	1	3672							
69	FAHFA(18:1-O-21:0)	OAHHEA	677.8	681.8	350.3	1	2112							
70	FAHFA(18:2-O-21:0)	LAHHEA	675.8	679.8	350.3	3	14778	2309	2265					

I, intensity (cps)

Chinese yam (*Rhizoma Disocoreae*)

NO.	Normal name	FAHFA ID	*m/z* (DMED labeling)	*m/z* (d_4-DMED labeling)	Product ion	Regioisomer number	*I1*	*I2*	*I3*	*I4*	*I5*	*I6*	*I7*	*I8*	*I9*	*I10*	*I11*	*I12*
1	FAHFA(18:2-O-14:1)	LAHMO	575.5	579.5	250.2	3	4371	1737	1951									
2	FAHFA(20:3-O-14:1)	ETAHMO	601.5	605.5	250.2	2	4544	6651										
3	FAHFA(22:2-O-14:1)	DDAHMO	631.5	635.5	250.2	1	2632											
4	FAHFA(18:2-O-14:2)	LAHTDA	573.5	577.5	248.2	4	2662	1702	11102	2282								
5	FAHFA(20:0-O-14:2)	AAHTDA	605.5	609.5	248.2	2	3351	3491										
6	FAHFA(20:1-O-14:2)	EAHTDA	603.5	607.5	248.2	1	1195											
7	FAHFA(22:2-O-14:2)	DDAHTDA	629.5	633.5	248.2	3	975	8202	4310									
8	FAHFA(17:0-O-16:1)	HDAHPO	593.5	597.5	278.2	1	3020											
9	FAHFA(18:2-O-16:1)	LAHPO	603.5	607.5	278.2	11	2146	11724	8399	4581	3736	3746	4455	5549	3156	3600	2793	
10	FAHFA(20:2-O-16:2)	EDAHHDA	629.5	633.5	276.2	3	2295	1242	1299									
11	FAHFA(16:0-O-16:3)	PAHHTA	575.5	579.5	276.2	1	1712											
12	FAHFA(18:2-O-16:3)	LAHHTA	599.5	603.5	276.2	1	1836											
13	FAHFA(16:1-O-18:1)	POHOA	605.5	609.5	306.2	4	33427	12229	11807	5177								
14	FAHFA(16:0-O-18:1)	PAHOA	607.5	611.5	306.2	12	1468	2868	2277	2113	36683	69368	17096	31132	55924	61822	57828	59413
15	FAHFA(18:0-O-18:1)	SAHOA	635.5	639.5	306.2	4	8247	23869	8580	7009								
16	FAHFA(18:1-O-18:1)	OAHOA	633.5	637.5	306.2	9	35706	86759	14396	18559	30978	36599	6143	19108	13727			
17	FAHFA(18:2-O-18:1)	LAHOA	631.5	635.5	306.2	9	6262	88870	2.00E+05	51919	89988	131359	12383	54622	80252			
18	FAHFA(18:4-O-18:1)	SDAHOA	627.5	631.5	306.2	3	18460	8971	7554									
19	FAHFA(20:1-O-18:1)	EAHOA	661.5	665.5	306.2	1	5437											
20	FAHFA(22:2-O-18:1)	DDAHOA	687.5	691.5	306.2	1	5260											
21	FAHFA(14:0-O-18:2)	MAHLA	577.5	581.5	304.2	5	3168	1360	8262	12925	3706							
22	FAHFA(15:0-O-18:2)	PDAHLA	591.5	595.5	304.2	5	1399	17353	12354	1245	8752							
23	FAHFA(16:0-O-18:2)	PAHLA	605.5	609.5	304.2	6	11745	65132	555125	602527	12846	203700						
24	FAHFA(17:0-O-18:2)	HDAHLA	619.5	623.5	304.2	2	16499	9263										
25	FAHFA(17:1-O-18:2)	HDEAHLA	617.5	621.5	304.2	2	8871	6834										
26	FAHFA(18:0-O-18:2)	SAHLA	633.5	637.5	304.2	3	134085	118323	37280									
27	FAHFA(18:1-O-18:2)	OAHLA	631.5	635.5	304.2	6	67418	56727	61884	363372	278128	51163						
28	FAHFA(18:2-O-18:2)	LAHLA	629.5	632.5	304.2	4	232269	2.30E+06	1.30E+06	2.70E+05								
29	FAHFA(20:0-O-18:2)	AAHLA	661.5	665.5	304.2	2	6617	3036										
30	FAHFA(20:1-O-18:2)	EAHLA	659.5	663.5	304.2	2	9113	6251										
31	FAHFA(20:2-O-18:2)	EDAHLA	657.5	661.5	304.2	3	27719	6549	4109									
32	FAHFA(20:3-O-18:2)	ETAHLA	655.5	659.5	304.2	2	10918	7738										
33	FAHFA(22:0-O-18:2)	BAHLA	689.5	693.5	304.2	2	5658	3187										
34	FAHFA(22:2-O-18:2)	DDAHLA	685.5	689.5	304.2	4	5763	25096	37879	5834								
35	FAHFA(16:0-O-18:3)	PAHALA	603.5	607.5	302.2	5	22857	25250	45011	13517	21084							
36	FAHFA(18:1-O-18:3)	OAHALA	629.5	633.5	302.2	3	8826	22717	34958									
37	FAHFA(18:2-O-18:3)	LAHALA	627.5	631.5	302.2	5	83521	183937	5569	753	27894							
38	FAHFA(20:5-O-18:3)	EPAHALA	649.5	653.5	302.2	4	7103	4549	11805	10997								
39	FAHFA(20:3-O-20:1)	ETAHEA	685.5	689.5	334.2	1	2548											
40	FAHFA(18:2-O-20:2)	LAHEDA	657.5	661.5	332.2	3	2863	4688	1587									
41	FAHFA(14:0-O-12:0)	MAHDA	497.5	501.5	224.2	1	4022											
42	FAHFA(15:1-O-12:0)	PDEAHDA	509.5	513.5	224.2	1	18203											
43	FAHFA(16:1-O-12:0)	POHDA	523.5	527.5	224.2	1	4569											
44	FAHFA(16:0-O-12:0)	PAHDA	525.5	529.5	224.2	3	12059	14676	8052									
45	FAHFA(17:1-O-12:0)	HDEAHDA	537.5	541.5	224.2	1	13370											
46	FAHFA(18:0-O-12:0)	SAHDA	553.5	557.5	224.2	3	6213	6223	6278									
47	FAHFA(18:1-O-12:0)	OAHDA	551.5	555.5	224.2	2	8315	3068										
48	FAHFA(18:2-O-12:0)	LAHDA	549.5	553.5	224.2	3	2231	23121	8926									
49	FAHFA(18:3-O-12:0)	ALAHDA	547.5	551.5	224.2	2	3856	4305										
50	FAHFA(22:2-O-12:0)	DDAHDA	605.5	609.5	224.2	4	11784	1107	707	1025								
51	FAHFA(14:0-O-14:0)	MAHMA	525.5	529.5	252.2	3	2400	3956	36973									
52	FAHFA(16:0-O-14:0)	PAHMA	553.5	557.5	252.2	8	1136	7060	1746	1505	1025	2106	80211	5108				
53	FAHFA(18:0-O-14:0)	SAHMA	581.5	585.5	252.2	3	5393	14230	4416									
54	FAHFA(18:1-O-14:0)	OAHMA	579.5	583.5	252.2	3	6614	28394	978									
55	FAHFA(18:2-O-14:0)	LAHMA	577.5	581.5	252.2	2	151300	5583										
56	FAHFA(18:3-O-14:0)	ALAHMA	575.5	579.5	252.2	1	31194											
57	FAHFA(20:2-O-14:0)	EDAHMA	605.5	609.5	252.2	1	2591											
58	FAHFA(16:0-O-15:0)	PAHPDA	567.5	571.5	266.2	4	2275	3411	2663	34825								
59	FAHFA(18:0-O-15:0)	SAHPDA	595.5	599.5	266.2	1	5125											
60	FAHFA(18:1-O-15:0)	OAHPDA	593.5	597.5	266.2	1	6865											
61	FAHFA(18:2-O-15:0)	LAHPDA	591.5	595.5	266.2	6	13981	4968	8545	1752	3054	55676						
62	FAHFA(18:3-O-15:0)	ALAHPDA	589.5	593.5	266.2	1	8524											
63	FAHFA(15:0-O-16:0)	PDAHPA	567.6	571.6	280.3	1	7803											
64	FAHFA(16:0-O-16:0)	PAHPA	581.6	585.6	280.3	9	63200	18643	18181	20822	15674	29150	13313	19816	285956			
65	FAHFA(18:0-O-16:0)	SAHPA	609.6	613.6	280.3	6	640793	43629	26084	16325	36751	43494						
66	FAHFA(18:1-O-16:0)	OAHPA	607.6	611.6	280.3	4	8276	13658	7404	80499								
67	FAHFA(18:2-O-16:0)	LAHPA	605.6	609.6	280.3	2	25617	491412										
68	FAHFA(18:3-O-16:0)	ALAHPA	603.6	607.6	280.3	1	70165											
69	FAHFA(14:0-O-18:0)	MAHSA	581.8	585.8	308.3	5	6063	5284	2017	8998	9515							
70	FAHFA(16:1-O-18:0)	POHSA	607.8	611.8	308.3	1	18979											
71	FAHFA(16:0-O-18:0)	PAHSA	609.8	613.8	308.3	11	78685	86414	29125	70818	64542	13794	8327	5199	2470	3436	8127	
72	FAHFA(18:0-O-18:0)	SAHSA	637.8	641.8	308.3	5	77370	112066	40690	66067	63002							
73	FAHFA(18:1-O-18:0)	OAHSA	635.8	639.8	308.3	4	5151	5373	14707	7114								
74	FAHFA(18:3-O-18:0)	ALAHSA	631.8	635.8	308.3	2	26454	2060										
75	FAHFA(22:6-O-18:0)	DHAHSA	681.8	685.8	308.3	1	1764											
76	FAHFA(15:1-O-19:0)	PDEAHNDA	607.8	611.8	322.3	1	12656											
77	FAHFA(16:0-O-19:0)	PAHNDA	623.8	627.8	322.3	5	34811	39687	17746	13290	14075							
78	FAHFA(18:1-O-19:0)	OAHNDA	649.8	653.8	322.3	5	19656	21161	8367	7535	9272							
79	FAHFA(18:2-O-19:0)	LAHNDA	647.8	651.8	322.3	5	76462	82033	26864	14271	33153							
80	FAHFA(20:3-O-19:0)	ETAHNDA	673.8	677.8	322.3	2	2544	990										
81	FAHFA(18:0-O-20:0)	SAHAA	665.8	669.8	336.3	1	9269											
82	FAHFA(18:1-O-20:0)	OAHAA	663.8	667.7	336.3	1	13638											
83	FAHFA(20:1-O-20:0)	EAHAA	691.8	695.8	336.3	1	4455											
84	FAHFA(20:2-O-20:0)	EDAHAA	689.8	693.8	336.3	1	6215											
85	FAHFA(20:3-O-20:0)	ETAHAA	687.5	691.5	336.3	2	1398	23621										

I, intensity (cps)

Lycium chinense

NO.	Normal name	FAHFA ID	*m/z* (DMED labeling)	*m/z* (d_4-DMED labeling)	Product ion	Regioisomer number	*I1*	*I2*	*I3*	*I4*	*I5*	*I6*	*I7*	*I8*	*I9*	*I10*	*I11*
1	FAHFA(15:0-O-14:1)	PDAHMO	537.5	541.5	250.2	1	14950										
2	FAHFA(15:1-O-14:1)	PDEAHMO	535.5	539.5	250.2	1	3674										
3	FAHFA(16:0-O-14:1)	PAHMO	551.5	555.5	250.2	5	4407	2574	13358	3340	4476						
4	FAHFA(18:1-O-14:1)	OAHMO	577.5	581.5	250.2	1	15311										
5	FAHFA(18:2-O-14:1)	LAHMO	575.5	579.5	250.2	5	6917	8320	31904	2840	3934						
6	FAHFA(18:3-O-14:1)	ALAHMO	573.5	577.5	250.2	1	5776										
7	FAHFA(20:2-O-14:1)	EDAHMO	603.5	607.5	250.2	1	2943										
8	FAHFA(14:1-O-14:2)	MOHTDA	519.5	523.5	248.2	1	4324										
9	FAHFA(18:1-O-14:2)	OAHTDA	575.5	579.5	248.2	2	3632	4626									
10	FAHFA(20:0-O-14:2)	AAHTDA	605.5	609.5	248.2	3	2521	13372	2836								
11	FAHFA(20:1-O-14:2)	EAHTDA	603.5	607.5	248.2	1	2205										
12	FAHFA(22:1-O-14:2)	DEAHTDA	631.5	635.5	248.2	3	1325	10102	1115								
13	FAHFA(22:2-O-14:2)	DDAHTDA	629.5	633.5	248.2	3	3130	20691	1782								
14	FAHFA(14:0-O-14:3)	MAHTTA	519.5	523.5	246.2	1	1747										
15	FAHFA(16:0-O-14:3)	PAHTTA	547.5	551.5	246.2	5	3908	10518	15002	41096	2223						
16	FAHFA(18:0-O-14:3)	SAHTTA	575.5	579.5	246.2	1	4575										
17	FAHFA(22:2-O-14:3)	DDAHTTA	627.5	631.5	246.2	1	2968										
18	FAHFA(14:0-O-16:1)	MAHPO	551.5	555.5	278.2	1	8969										
19	FAHFA(16:0-O-16:1)	PAHPO	579.5	583.5	278.2	8	9442	18448	92646	10703	36135	18428	14423	293253			
20	FAHFA(18:0-O-16:1)	SAHPO	607.5	611.5	278.2	1	53679										
21	FAHFA(18:1-O-16:1)	OAHPO	605.5	609.5	278.2	8	11237	11254	6770	7668	10446	5159	5758	93301			
22	FAHFA(18:2-O-16:1)	LAHPO	603.5	607.5	278.2	9	9994	7209	18833	19407	10357	5556	7041	6567	216535		
23	FAHFA(18:3-O-16:1)	ALAHPO	601.5	605.5	278.2	1	42349										
24	FAHFA(20:5-O-16:1)	EPAHPO	625.5	629.5	278.2	5	6255	4646	8466	7076	3539						
25	FAHFA(16:0-O-16:2)	PAHHDA	577.5	581.5	276.2	2	28350	3530									
26	FAHFA(18:0-O-16:2)	SAHHDA	605.5	609.5	276.2	1	3736										
27	FAHFA(18:1-O-16:2)	OAHHDA	603.5	607.5	276.2	2	15701	2066									
28	FAHFA(18:2-O-16:2)	LAHHDA	601.5	605.5	276.2	3	35782	4689	3655								
29	FAHFA(20:2-O-16:2)	EDAHHDA	629.5	633.5	276.2	1	4755										
30	FAHFA(14:0-O-16:3)	MAHHTA	547.5	551.5	274.2	1	2980										
31	FAHFA(16:0-O-16:3)	PAHHTA	575.5	579.5	274.2	2	9333	71740									
32	FAHFA(18:0-O-16:3)	SAHHTA	603.5	607.5	274.2	1	8401										
33	FAHFA(20:1-O-16:3)	EAHHTA	629.5	633.5	274.2	2	2120	3674									
34	FAHFA(20:2-O-16:3)	EDAHHTA	627.5	631.5	274.2	1	7114										
35	FAHFA(14:0-O-18:1)	MAHOA	579.5	583.5	306.2	7	9172	25743	11887	3159	11464	7041	6213				
36	FAHFA(16:1-O-18:1)	POHOA	605.5	609.5	306.2	8	23662	13894	7612	9173	49462	7967	4512	5123			
37	FAHFA(16:0-O-18:1)	PAHOA	607.5	611.5	306.2	7	280835	751723	226243	303731	180309	96007	207500				
38	FAHFA(18:0-O-18:1)	SAHOA	635.5	639.5	306.2	5	14640	19500	50711	30773	24881						
39	FAHFA(18:1-O-18:1)	OAHOA	633.5	637.5	306.2	7	46882	204996	311578	90447	233994	27781	68926				
40	FAHFA(18:2-O-18:1)	LAHOA	631.5	635.5	306.2	6	399653	1524598	150854	360478	29417	107587					
41	FAHFA(20:0-O-18:1)	AAHOA	663.5	667.5	306.2	2	13784	7427									
42	FAHFA(20:1-O-18:1)	EAHOA	661.5	665.5	306.2	2	4515	3645									
43	FAHFA(20:5-O-18:1)	EPAHOA	653.5	657.5	306.2	4	9524	13064	30502	44994							
44	FAHFA(22:0-O-18:1)	BAHOA	691.5	695.5	306.2	1	8148										
45	FAHFA(22:1-O-18:1)	DEAHOA	689.5	693.5	306.2	1	7813										
46	FAHFA(22:2-O-18:1)	DDAHOA	687.5	691.5	306.2	1	2688										
47	FAHFA(14:0-O-18:2)	MAHLA	577.5	581.5	304.2	5	6672	4812	78752	11110	4462						
48	FAHFA(15:0-O-18:2)	PDAHLA	591.5	595.5	304.2	3	2619	20228	1964								
49	FAHFA(15:1-O-18:2)	PDEAHLA	589.5	593.5	304.5	3	2730	5386	30929								
50	FAHFA(16:1-O-18:2)	POHLA	603.5	607.5	304.5	1	120437										
51	FAHFA(16:0-O-18:2)	PAHLA	605.5	609.5	304.5	7	64211	75241	63871	2415526	408517	25773	133127				
52	FAHFA(17:0-O-18:2)	HDAHLA	619.5	623.5	304.65	1	95413										
53	FAHFA(17:1-O-18:2)	HDEAHLA	617.5	621.5	304.74	2	30921	9281									
54	FAHFA(18:0-O-18:2)	SAHLA	633.5	637.5	304.83	4	61302	167478	50273	21717							
55	FAHFA(18:1-O-18:2)	OAHLA	631.5	635.5	304.92	6	156052	72036	36608	2218520	286926	50126					
56	FAHFA(18:2-O-18:2)	LAHLA	629.5	633.5	305.01	4	137052	58591	5893666	623769							
57	FAHFA(20:0-O-18:2)	AAHLA	661.5	665.5	305.1	4	3129	59354	8019	2805							
58	FAHFA(20:1-O-18:2)	EAHLA	659.5	663.5	305.19	1	10356										
59	FAHFA(20:2-O-18:2)	EDAHLA	657.5	661.5	305.28	2	11230	9493									
60	FAHFA(20:5-O-18:2)	EPAHLA	651.5	655.5	305.37	4	34357	54261	20273	24770							
61	FAHFA(22:0-O-18:2)	BAHLA	689.5	693.5	305.46	3	3006	32920	5081								
62	FAHFA(14:0-O-18:3)	MAHALA	575.5	579.5	302.2	2	4291	13724									
63	FAHFA(15:0-O-18:3)	PDAHALA	589.5	593.5	302.2	2	7568	3215									
64	FAHFA(16:1-O-18:3)	POHALA	601.5	605.5	302.2	2	4087	21592									
65	FAHFA(16:0-O-18:3)	PAHALA	603.5	607.5	302.2	4	32837	107776	414936	33085							
66	FAHFA(17:0-O-18:3)	HDAHALA	617.5	621.5	302.2	2	5429	13508									
67	FAHFA(18:0-O-18:3)	SAHALA	631.5	635.5	302.2	4	6700	9179	43192	7136							
68	FAHFA(18:1-O-18:3)	OAHALA	629.5	633.5	302.2	6	13294	21573	41482	263415	16635	14438					
69	FAHFA(18:2-O-18:3)	LAHALA	627.5	631.5	302.2	6	52058	82160	535833	34700	7088	21679					
70	FAHFA(20:0-O-18:3)	AAHALA	659.5	663.5	302.2	2	4986	8170									
71	FAHFA(22:0-O-18:3)	BAHALA	687.5	691.5	302.2	3	6877	3626	7835								
72	FAHFA(22:2-O-18:3)	DDAHALA	683.5	687.5	302.2	1	5061										
73	FAHFA(16:0-O-20:1)	PAHEA	635.5	639.5	334.2	1	11052										
74	FAHFA(16:0-O-20:2)	PAHEDA	633.5	637.5	332.2	1	7502										
75	FAHFA(16:0-O-22:6)	PAHDHA	653.5	657.5	352.2	2	22546	31176									
76	FAHFA(18:1-O-22:6)	OAHDHA	679.5	683.5	352.2	4	19033	27375	6546	7700							
77	FAHFA(14:0-O-12:0)	MAHDA	497.5	501.5	224.2	2	2233	4187									
78	FAHFA(16:1-O-12:0)	POHDA	523.5	527.5	224.2	1	9845										
79	FAHFA(16:0-O-12:0)	PAHDA	525.5	529.5	224.2	6	16989	9590	8759	10970	78657	8560					
80	FAHFA(18:0-O-12:0)	SAHDA	553.5	557.5	224.2	4	2405	2069	10791	2304							
81	FAHFA(18:1-O-12:0)	OAHDA	551.5	555.5	224.2	7	3293	10063	9005	6115	7331	57226	2997				
82	FAHFA(18:2-O-12:0)	LAHDA	549.5	553.5	224.2	4	15073	3814	29988	4048							
83	FAHFA(18:3-O-12:0)	ALAHDA	547.5	551.5	224.2	2	3584	4605									
84	FAHFA(14:0-O-14:0)	MAHMA	525.5	529.5	252.2	2	11722	456821									
85	FAHFA(14:1-O-14:0)	MOHMA	523.5	527.5	252.2	1	1570										
86	FAHFA(16:1-O-14:0)	POHMA	551.5	555.5	252.2	1	9584										
87	FAHFA(16:0-O-14:0)	PAHMA	553.5	557.5	252.2	4	43497	16357	541756	18412							
88	FAHFA(18:0-O-14:0)	SAHMA	581.5	585.5	252.2	2	19904	7129									
89	FAHFA(18:1-O-14:0)	OAHMA	579.5	583.5	252.2	4	5254	13024	92063	6154							
90	FAHFA(18:2-O-14:0)	LAHMA	577.5	581.5	252.2	4	25229	6519	80467	9068							
91	FAHFA(18:3-O-14:0)	ALAHMA	575.5	579.5	252.2	2	3932	15075									
92	FAHFA(16:0-O-15:0)	PAHPDA	567.5	571.5	266.2	3	18460	8077	6819								
93	FAHFA(18:2-O-15:0)	LAHPDA	591.5	595.5	266.2	2	8849	6089									
94	FAHFA(14:0-O-16:0)	MAHPA	553.6	557.6	280.3	10	15720	2834	4574	2843	2386	3800	2285	8749	49355	19009	
95	FAHFA(15:0-O-16:0)	PDAHPA	567.6	571.6	280.3	11	3079	4360	2136	1801	1702	1583	1487	1509	1196	3691	2899
96	FAHFA(15:1-O-16:0)	PDEAHPA	565.6	569.6	280.3	3	2257	3961	2693								
97	FAHFA(16:1-O-16:0)	POHPA	579.6	583.6	280.3	4	24107	7172	61822	61357							
98	FAHFA(16:0-O-16:0)	PAHPA	581.6	585.6	280.3	10	484666	42748	98296	67794	38671	67256	44546	45427	2229018	596774	
99	FAHFA(18:1-O-16:0)	OAHPA	607.6	611.6	280.3	8	12569	322839	123990	19231	10342	19841	168517	352997			

100	FAHFA(18:2-O-16:0)	LAHPA	605.6	609.6	280.3	9	493029	13597	35071	16457	14676	14711	16210	117989	557146
101	FAHFA(18:3-O-16:0)	ALAHPA	603.6	607.6	280.3	2	94846	84208							
102	FAHFA(18:4-O-16:0)	SDAHPA	601.6	605.6	280.3	1	15203								
103	FAHFA(20:0-O-16:0)	AAHPA	637.6	641.6	280.3	1	12995								
104	FAHFA(20:1-O-16:0)	EAHPA	635.6	639.6	280.3	1	7089								
105	FAHFA(20:2-O-16:0)	EDAHPA	633.6	637.6	280.3	1	4106								
106	FAHFA(20:3-O-16:0)	ETAHPA	631.6	635.6	280.3	1	4825								
107	FAHFA(22:0-O-16:0)	BAHPA	665.6	669.6	280.3	1	4668								
108	FAHFA(22:2-O-16:0)	DDAHPA	661.5	665.5	280.3	1	6182								
109	FAHFA(18:1-O-17:0)	OAHHDA	621.6	625.6	294.3	2	13341	17402							
110	FAHFA(22:0-O-17:0)	BAHHDA	679.6	683.6	294.3	3	10204	2958	3068						
111	FAHFA(14:0-O-18:0)	MAHSA	581.8	585.8	308.3	2	13502	6458							
112	FAHFA(16:1-O-18:0)	POHSA	607.8	611.8	308.3	4	44634	22122	4793	5460					
113	FAHFA(16:0-O-18:0)	PAHSA	609.8	613.8	308.3	9	5291	14515	34308	173584	42618	9182	3900	47740	6108
114	FAHFA(18:0-O-18:0)	SAHSA	637.8	641.8	308.3	4	32409	26341	22592	14241					
115	FAHFA(18:1-O-18:0)	OAHSA	635.8	639.8	308.3	6	8565	17588	14931	99605	9254	16333			
116	FAHFA(18:2-O-18:0)	LAHSA	633.8	637.8	308.3	8	8545	29033	28799	22524	85925	10701	17517	11004	
117	FAHFA(18:3-O-18:0)	ALAHSA	631.8	635.8	308.3	3	11126	33451	22071						
118	FAHFA(22:1-O-18:0)	DEAHSA	691.8	695.8	308.3	1	5038								
119	FAHFA(14:0-O-19:0)	MAHNDA	595.8	599.8	322.3	4	3205	4250	3598	3666					
120	FAHFA(15:1-O-19:0)	PDEAHNDA	607.8	611.8	322.3	2	1256	11685							
121	FAHFA(16:1-O-19:0)	POHNDA	621.8	625.8	322.3	5	16538	18504	11202	7364	36994				
122	FAHFA(16:0-O-19:0)	PAHNDA	623.8	627.8	322.3	7	16458	383036	387789	250146	41659	236184	69669		
123	FAHFA(18:0-O-19:0)	SAHNDA	651.8	655.8	322.3	7	33151	19825	17346	71985	81554	62606	44165		
124	FAHFA(18:1-O-19:0)	OAHNDA	649.8	653.8	322.3	8	35560	47185	29299	591824	712338	331568	376592	58015	
125	FAHFA(18:2-O-19:0)	LAHNDA	647.8	651.8	322.3	5	1007297	1444280	932438	642314	38926				
126	FAHFA(20:0-O-19:0)	AAHNDA	679.8	683.8	322.3	6	6657	20863	23247	21783	12908	4116			
127	FAHFA(22:0-O-19:0)	BAHNDA	707.8	711.8	322.3	7	19464	11990	18355	15927	37474	7997	4388		
128	FAHFA(22:1-O-19:0)	DEAHNDA	705.8	709.8	322.3	1	21912								
129	FAHFA(22:6-O-19:0)	DHAHNDA	695.8	699.8	322.3	1	3724								
130	FAHFA(16:1-O-20:0)	POHAA	635.8	639.8	336.3	1	3894								
131	FAHFA(18:2-O-20:0)	LAHAA	661.8	665.8	336.3	6	14173	11184	8155	9584	18504	49147			
132	FAHFA(18:1-O-21:0)	OAHHEA	677.8	681.8	350.3	4	2586	2904	1616	6918					
133	FAHFA(18:2-O-21:0)	LAHHEA	675.8	679.8	350.3	1	13953								

I, intensity (cps)

Coptis chinensis

NO.	Normal name	FAHFA ID	*m/z* (DMED labeling)	*m/z* (d_4-DMED labeling)	Product ion	Regioisomer number	*I1*	*I2*	*I3*	*I4*	*I5*	*I6*	*I7*	*I8*	*I9*	*I10*
1	FAHFA(17:0-O-14:2)	HDAHTDA	563.5	567.5	248.2	1	5314									
2	FAHFA(18:1-O-14:2)	OAHTDA	575.5	579.5	248.2	1	2438									
3	FAHFA(20:0-O-14:2)	AAHTDA	605.5	609.5	248.2	1	1649									
4	FAHFA(17:0-O-14:3)	HDAHTTA	561.5	565.5	246.2	2	271563	20067								
5	FAHFA(16:1-O-16:1)	POHPO	577.5	581.5	278.2	2	104794	53722								
6	FAHFA(16:0-O-16:1)	PAHPO	579.5	583.5	278.2	2	90985	37904								
7	FAHFA(18:1-O-16:1)	OAHPO	605.5	609.5	278.2	2	42215	18120								
8	FAHFA(18:2-O-16:1)	LAHPO	603.5	607.5	278.2	8	3458	50384	18495	4444	5068	3421	14138	9660		
9	FAHFA(20:4-O-16:1)	ARAHPO	627.5	631.5	278.2	1	9903									
10	FAHFA(16:1-O-18:1)	POHOA	605.5	609.5	306.2	6	52722	21835	24364	14415	21149	4862				
11	FAHFA(16:0-O-18:1)	PAHOA	607.5	611.5	306.2	9	110552	39114	48788	25007	54000	36395	5965	6662	12302	
12	FAHFA(18:1-O-18:1)	OAHOA	633.5	637.5	306.2	6	12111	49763	25221	25070	51432	32888				
13	FAHFA(18:2-O-18:1)	LAHOA	631.5	635.5	306.2	4	225743	35750	77307	27553						
14	FAHFA(22:1-O-18:1)	DEAHOA	689.5	693.5	306.2	1	30030									
15	FAHFA(14:0-O-18:2)	MAHLA	577.5	581.5	304.2	1	8095									
16	FAHFA(16:1-O-18:2)	POHLA	603.5	607.5	304.2	4	2700	60294	24779	3256						
17	FAHFA(16:0-O-18:2)	PAHLA	605.5	609.5	304.2	3	290976	71261	9473							
18	FAHFA(17:1-O-18:2)	HDEAHLA	617.5	621.5	304.2	2	10045	9407								
19	FAHFA(18:0-O-18:2)	SAHLA	633.5	637.5	304.2	2	31858	13335								
20	FAHFA(18:1-O-18:2)	OAHLA	631.5	635.5	304.2	3	20399	204776	44197							
21	FAHFA(18:2-O-18:2)	LAHLA	629.5	633.5	304.2	2	669752	91955								
22	FAHFA(22:0-O-18:2)	BAHLA	689.5	693.5	304.2	1	5952									
23	FAHFA(22:1-O-18:2)	DEAHLA	687.5	691.5	304.2	1	11452									
24	FAHFA(22:2-O-18:2)	DDAHLA	685.5	689.5	304.2	1	15586									
25	FAHFA(16:1-O-18:3)	POHALA	601.5	605.5	302.2	3	7537	5184	1455							
26	FAHFA(16:0-O-18:3)	PAHALA	603.5	607.5	302.2	3	35670	27001	5491							
27	FAHFA(18:1-O-18:3)	OAHALA	629.5	633.5	302.2	4	2823	2453	26195	15142						
28	FAHFA(18:2-O-18:3)	LAHALA	627.5	631.5	302.2	3	95060	74298	10123							
29	FAHFA(15:0-O-22:6)	PDAHDHA	639.5	643.5	352.2	1	12721									
30	FAHFA(15:1-O-22:6)	PDEAHDHA	637.5	641.5	352.2	1	8693									
31	FAHFA(17:1-O-22:6)	HDEAHDHA	665.5	669.5	352.2	1	6816									
32	FAHFA(14:0-O-12:0)	MAHDA	497.5	501.5	224.2	1	7458									
33	FAHFA(16:0-O-12:0)	PAHDA	525.5	529.5	224.2	3	2247	6866	6727							
34	FAHFA(17:0-O-12:0)	HDAHDA	539.5	543.5	224.2	1	4374									
35	FAHFA(17:1-O-12:0)	HDEAHDA	537.5	541.5	224.2	1	3865									
36	FAHFA(18:0-O-12:0)	SAHDA	553.5	557.5	224.2	2	1054	2078								
37	FAHFA(18:2-O-12:0)	LAHDA	549.5	553.5	224.2	3	3685	2827	11559							
38	FAHFA(14:0-O-14:0)	MAHMA	525.5	529.5	252.2	1	1594820									
39	FAHFA(14:1-O-14:0)	MOHMA	523.5	527.5	252.2	2	3853	1505								
40	FAHFA(15:1-O-14:0)	PDEAHMA	537.5	541.5	252.2	1	11141									
41	FAHFA(16:1-O-14:0)	POHMA	551.5	555.5	252.2	1	9028									
42	FAHFA(16:0-O-14:0)	PAHMA	553.5	557.5	252.2	2	60509	4752								
43	FAHFA(18:0-O-14:0)	SAHMA	581.5	585.5	252.2	1	9219									
44	FAHFA(18:1-O-14:0)	OAHMA	579.5	583.5	252.2	2	29179	2825								
45	FAHFA(18:2-O-14:0)	LAHMA	577.5	581.5	252.2	2	77876	6878								
46	FAHFA(18:3-O-14:0)	ALAHMA	575.5	579.5	252.2	1	11582									
47	FAHFA(20:0-O-14:0)	AAHMA	609.5	613.5	252.2	1	6582									
48	FAHFA(15:0-O-15:0)	PDAHPDA	553.5	557.5	266.2	1	7753									
49	FAHFA(15:1-O-15:0)	PDEAHPDA	551.5	555.5	266.2	1	14275									
50	FAHFA(16:0-O-15:0)	PAHPDA	567.5	571.5	266.2	2	4385	14824								
51	FAHFA(18:0-O-15:0)	SAHPDA	595.5	599.5	266.2	1	2113									
52	FAHFA(18:1-O-15:0)	OAHPDA	593.5	597.5	266.2	2	2418	6013								
53	FAHFA(18:2-O-15:0)	LAHPDA	591.5	595.5	266.2	3	5021	8737	6946							
54	FAHFA(18:3-O-15:0)	ALAHPDA	589.5	593.5	266.2	1	5918									
55	FAHFA(15:0-O-16:0)	PDAHPA	567.6	571.6	280.3	1	9234									
56	FAHFA(16:1-O-16:0)	POHPA	579.6	583.6	280.3	4	33537	710574	55539	35166						
57	FAHFA(16:0-O-16:0)	PAHPA	581.6	585.6	280.3	6	19313	39062	541277	16907	16845	72021				
58	FAHFA(17:0-O-16:0)	HDAHPA	595.5	599.5	280.3	1	697360									
59	FAHFA(18:0-O-16:0)	SAHPA	609.6	613.6	280.3	1	56016									
60	FAHFA(18:1-O-16:0)	OAHPA	607.6	611.6	280.3	10	6907	30925	199527	10391	4811	3609	3006	3171	12653	41996
61	FAHFA(18:2-O-16:0)	LAHPA	605.6	609.5	280.3	5	67117	319406	19231	17516	88963					
62	FAHFA(18:3-O-16:0)	ALAHPA	603.6	607.6	280.3	2	18675	72285								
63	FAHFA(20:1-O-16:0)	EAHPA	635.6	639.6	280.3	1	5276									
64	FAHFA(20:3-O-16:0)	ETAHPA	631.6	635.6	280.3	2	9564	33147								
65	FAHFA(20:4-O-16:0)	ARAHPA	629.6	633.6	280.3	1	278633									
66	FAHFA(20:5-O-16:0)	EPAHPA	627.6	631.6	208.3	2	20248	10732								
67	FAHFA(15:0-O-17:0)	PDAHHDA	581.6	585.6	294.3	1	49866									
68	FAHFA(15:1-O-17:0)	PDEAHHDA	579.6	583.6	294.3	1	20781									
69	FAHFA(16:0-O-17:0)	PAHHDA	581.6	585.6	294.3	1	55115									
70	FAHFA(18:2-O-17:0)	LAHHDA	619.6	623.6	294.3	3	9251	11602	7695							
71	FAHFA(20:0-O-17:0)	AAHHDA	651.6	655.6	294.3	1	11389									
72	FAHFA(14:0-O-18:0)	MAHSA	581.8	585.8	308.3	2	3525	2687								
73	FAHFA(15:0-O-18:0)	PDAHSA	595.8	599.8	308.3	1	3981									
74	FAHFA(16:1-O-18:0)	POHSA	607.8	611.8	308.3	5	47795	43049	50017	21985	56117					
75	FAHFA(16:0-O-18:0)	PAHSA	609.8	613.8	308.3	5	21571	26812	81315	15154	66769					
76	FAHFA(17:0-O-18:0)	HDAHSA	623.8	627.8	308.3	2	78359	825004								
77	FAHFA(18:0-O-18:0)	SAHSA	637.8	641.8	308.3	1	49529									
78	FAHFA(18:1-O-18:0)	OAHSA	635.8	639.8	308.3	4	17115	135772	23694	43133						
79	FAHFA(18:2-O-18:0)	LAHSA	633.8	637.8	308.3	3	38705	81466	170909							
80	FAHFA(18:3-O-18:0)	ALAHSA	631.8	635.8	308.3	3	10068	18080	36242							
81	FAHFA(20:5-O-18:0)	EPAHSA	655.8	659.8	308.3	1	10152									
82	FAHFA(22:6-O-18:0)	DHAHSA	681.8	685.8	308.3	4	2537	9364	3077	4819						
83	FAHFA(16:1-O-19:0)	POHNDA	621.8	625.8	322.3	4	4291	4469	7973	5888						
84	FAHFA(17:0-O-19:0)	HDAHNDA	637.8	641.8	322.3	1	23827									
85	FAHFA(18:0-O-19:0)	SAHNDA	651.8	655.8	322.3	2	6781	6643								
86	FAHFA(18:1-O-19:0)	OAHNDA	649.8	653.8	322.3	4	9651	12334	30000	27942						
87	FAHFA(18:2-O-19:0)	LAHNDA	647.8	651.8	322.3	4	23875	27107	84623	62727						
88	FAHFA(18:3-O-19:0)	ALAHNDA	645.8	649.8	322.3	5	4435	4276	7270	6831	3169					
89	FAHFA(16:1-O-20:0)	POHAA	635.8	639.8	336.3	1	33712									
90	FAHFA(16:0-O-20:0)	PAHAA	637.8	641.8	336.3	1	21282									
91	FAHFA(18:1-O-20:0)	OAHAA	663.8	667.8	336.3	1	29857									
92	FAHFA(18:2-O-20:0)	LAHAA	661.8	665.8	336.3	3	14077	55388	10101							
93	FAHFA(18:3-O-20:0)	ALAHAA	659.8	663.8	336.3	2	2885	15170								
94	FAHFA(16:1-O-21:0)	POHHEA	649.8	653.8	350.3	4	2985	1224	1530	4384						
95	FAHFA(18:1-O-21:0)	OAHHEA	677.8	681.8	350.3	1	5005									
96	FAHFA(18:2-O-21:0)	LAHHEA	675.8	679.8	350.3	1	23079									

97	FAHFA(18:3-O-21:0)	ALAHHEA	673.8	677.8	350.3	1	4841

I, intensity (cps)

Tomato (*Lycopersicon esculentum*)

NO.	Normal name	FAHFA ID	*m/z* (DMED labeling)	*m/z* (d_4-DMED labeling)	Product ion	Regioisomer number	*I1*	*I2*	*I3*	*I4*	*I5*	*I6*	*I7*	*I8*	*I9*	*I10*	*I11*
1	FAHFA(14:0-O-16:1)	MAHPO	551.5	555.5	278.2	2	2238	3590									
2	FAHFA(16:1-O-16:1)	POHPO	577.5	581.5	278.2	2	6842	5005									
3	FAHFA(17:0-O-16:1)	HDAHPO	593.5	597.5	278.2	1	2111										
4	FAHFA(16:1-O-16:2)	POHHDA	575.5	579.5	276.2	1	2252										
5	FAHFA(16:0-O-16:3)	PAHHTA	575.5	579.5	274.2	1	2030										
6	FAHFA(16:0-O-18:1)	PAHOA	607.5	611.5	306.2	1	58062										
7	FAHFA(18:0-O-18:1)	SAHOA	635.5	639.5	306.2	2	5333	49978									
8	FAHFA(18:1-O-18:1)	OAHOA	633.5	637.5	306.2	3	5345	162032	11097								
9	FAHFA(18:2-O-18:1)	LAHOA	631.5	635.5	306.2	2	210062	35671									
10	FAHFA(20:0-O-18:1)	AAHOA	663.5	667.5	306.2	1	2238										
11	FAHFA(20:1-O-18:1)	EAHOA	661.5	665.5	306.2	1	9966										
12	FAHFA(22:6-O-18:1)	DHAHOA	679.5	683.5	306.2	1	4231										
13	FAHFA(14:0-O-18:2)	MAHLA	577.5	581.5	304.2	2	11011	3858									
14	FAHFA(14:1-O-18:2)	MOHLA	575.5	579.5	304.2	1	5077										
15	FAHFA(15:0-O-18:2)	PDAHLA	591.5	595.5	304.2	1	2405										
16	FAHFA(16:0-O-18:2)	PAHLA	605.5	609.5	304.2	3	26999	9877	3250								
17	FAHFA(18:0-O-18:2)	SAHLA	633.5	637.5	304.2	2	4566	3334									
18	FAHFA(18:2-O-18:2)	LAHLA	629.5	633.5	304.2	2	4006	48589									
19	FAHFA(14:0-O-18:3)	MAHALA	575.5	579.5	302.2	1	4684										
20	FAHFA(16:0-O-18:3)	PAHALA	603.5	607.5	302.2	1	5265										
21	FAHFA(18:2-O-18:3)	LAHALA	627.5	631.5	302.2	2	5511	10717									
22	FAHFA(14:0-O-12:0)	MAHDA	497.5	501.5	224.2	2	1062	4303									
23	FAHFA(15:0-O-12:0)	PDAHDA	511.5	515.5	224.2	2	3771	2239									
24	FAHFA(16:1-O-12:0)	POHDA	523.5	527.5	224.2	1	7431										
25	FAHFA(16:0-O-12:0)	PAHDA	525.5	529.5	224.2	3	12149	2770	3920								
26	FAHFA(18:0-O-12:0)	SAHDA	553.5	557.5	224.2	3	5037	1995	2460								
27	FAHFA(14:0-O-14:0)	MAHMA	525.5	529.5	252.2	2	6907	4436									
28	FAHFA(16:1-O-14:0)	POHMA	551.5	555.5	252.2	2	3977	1041									
29	FAHFA(16:0-O-14:0)	PAHMA	553.5	557.5	252.2	3	10049	8192	7677								
30	FAHFA(18:0-O-14:0)	SAHMA	581.5	585.5	252.2	3	3095	2568	4044								
31	FAHFA(16:0-O-15:0)	PAHPDA	567.5	571.5	266.2	3	3088	3217	4815								
32	FAHFA(18:0-O-15:0)	SAHPDA	595.5	599.5	266.2	3	2007	1582	2780								
33	FAHFA(16:1-O-16:0)	POHPA	579.6	583.6	280.3	1	8348										
34	FAHFA(16:0-O-16:0)	PAHPA	581.6	585.6	280.3	11	60796	40955	25621	22407	22994	20292	24287	14816	9532	19667	129194
35	FAHFA(18:0-O-16:0)	SAHPA	609.6	613.6	280.3	11	65890	44359	25749	17333	20817	18883	22150	11557	7313	13133	68239
36	FAHFA(18:2-O-16:0)	LAHPA	605.6	609.6	280.3	1	11783										
37	FAHFA(18:3-O-16:0)	ALAHPA	603.6	607.6	280.3	2	12152	2626									
38	FAHFA(22:6-O-16:0)	DHAHPA	653.6	657.6	280.3	2	1763	936									
39	FAHFA(16:1-O-18:0)	POHSA	607.8	611.8	308.3	1	2919										
40	FAHFA(16:0-O-18:0)	PAHSA	609.8	613.8	308.3	10	41438	30427	27558	26638	8951	7348	6419	3855	6790	25500	
41	FAHFA(18:0-O-18:0)	SAHSA	637.8	641.8	308.3	9	63792	46666	32166	33354	6800	5105	4212	3649	8036		
42	FAHFA(22:6-O-18:0)	DHAHSA	681.8	685.8	308.3	1	1238										
43	FAHFA(18:2-O-19:0)	LAHNDA	647.8	651.8	322.3	2	2933	3001									
44	FAHFA(16:0-O-20:0)	PAHAA	637.8	641.8	336.3	2	2089	8863									
45	FAHFA(18:0-O-20:0)	SAHAA	665.8	669.8	336.3	2	5934	3626									
46	FAHFA(18:1-O-20:0)	OAHAA	663.8	667.8	336.3	1	3119										
47	FAHFA(20:1-O-20:0)	EAHAA	691.8	695.8	336.3	1	1670										
48	FAHFA(16:0-O-21:0)	PAHHEA	651.8	655.8	350.3	1	5121										
49	FAHFA(18:0-O-21:0)	SAHHEA	679.8	683.8	350.3	1	1439										

I, intensity (cps)

Apple (*Malus pumila*)

NO.	Normal name	FAHFA ID	*m/z* (DMED labeling)	*m/z* (d_4-DMED labeling)	Product ion	Regioisomer number	*I1*	*I2*	*I3*	*I4*	*I5*	*I6*	*I7*	*I8*	*I9*	*I10*	*I11*
1	FAHFA(16:1-O-16:1)	POHPO	577.5	581.5	278.2	2	4087	3731									
2	FAHFA(18:0-O-16:1)	SAHPO	607.5	611.5	278.2	1	6860										
3	FAHFA(20:5-O-16:2)	EPAHHDA	623.5	627.5	276.2	4	12076	12923	17895	3927							
4	FAHFA(20:5-O-16:3)	EPAHHTA	621.5	625.5	274.2	2	2390	4187									
5	FAHFA(14:1-O-18:1)	MOHOA	577.5	581.5	306.2	1	2687										
6	FAHFA(16:0-O-18:1)	PAHOA	607.5	611.5	306.2	5	5407	46092	14233	5331	3278						
7	FAHFA(18:0-O-18:1)	SAHOA	635.5	639.5	306.2	1	31440										
8	FAHFA(18:1-O-18:1)	OAHOA	633.5	637.5	306.2	5	4892	4310	124783	9655	13844						
9	FAHFA(18:2-O-18:1)	LAHOA	631.5	635.5	306.2	3	6925	164804	27996								
10	FAHFA(20:1-O-18:1)	EAHOA	661.5	665.5	306.2	1	8372										
11	FAHFA(22:2-O-18:1)	DDAHOA	687.5	691.5	306.2	1	3806										
12	FAHFA(22:6-O-18:1)	DHAHOA	679.5	683.5	306.2	1	8177										
13	FAHFA(16:0-O-18:2)	PAHLA	605.5	609.5	304.2	5	2542	33067	27218	2750	4365						
14	FAHFA(18:0-O-18:2)	SAHLA	633.5	637.5	304.2	2	18572	10580									
15	FAHFA(18:1-O-18:2)	OAHLA	631.5	635.5	304.2	4	7314	1619	8463	3525							
16	FAHFA(18:2-O-18:2)	LAHLA	629.5	633.5	304.2	4	8720	1696	24800	5662							
17	FAHFA(18:2-O-18:3)	LAHALA	627.5	631.5	302.2	1	6082										
18	FAHFA(14:0-O-12:0)	MAHDA	497.5	501.5	224.2	2	1692	6485									
19	FAHFA(15:0-O-12:0)	PDAHDA	511.5	515.5	224.2	2	6160	3283									
20	FAHFA(16:1-O-12:0)	POHDA	523.5	527.5	224.2	2	1414	7740									
21	FAHFA(16:0-O-12:0)	PAHDA	525.5	529.5	224.2	3	17666	7195	3862								
22	FAHFA(18:0-O-12:0)	SAHDA	553.5	557.5	224.2	3	6469	4116	3188								
23	FAHFA(14:0-O-14:0)	MAHMA	525.5	529.5	252.2	3	2219	5382	4176								
24	FAHFA(15:0-O-14:0)	PDAHMA	539.5	543.5	252.2	3	2985	1736	1013								
25	FAHFA(16:1-O-14:0)	POHMA	551.5	555.5	252.2	1	2411										
26	FAHFA(16:0-O-14:0)	PAHMA	553.5	557.5	252.2	7	12907	2276	1791	1235	2103	9101	8143				
27	FAHFA(18:0-O-14:0)	SAHMA	581.5	585.5	252.2	3	5188	4614	4772								
28	FAHFA(15:1-O-16:0)	PDEAHPA	565.6	569.6	280.3	1	13582										
29	FAHFA(16:0-O-16:0)	PAHPA	581.6	585.6	280.3	10	97997	50887	31800	27015	24444	37045	16224	11571	23445	114623	
30	FAHFA(17:1-O-16:0)	HDEAHPA	593.6	597.6	280.3	1	6894										
31	FAHFA(18:0-O-16:0)	SAHPA	609.6	613.6	280.3	11	131558	60818	40143	23431	25305	24839	41897	14215	9255	18722	72445
32	FAHFA(18:1-O-16:0)	OAHPA	607.6	613.6	280.3	2	2904	40488									
33	FAHFA(18:2-O-16:0)	LAHPA	605.6	609.6	280.3	1	38588										
34	FAHFA(20:3-O-16:0)	ETAHPA	663.6	667.6	280.3	1	14787										
35	FAHFA(18:0-O-17:0)	SAHHDA	623.6	627.6	294.3	3	10290	8298	4273								
36	FAHFA(18:1-O-17:0)	OAHHDA	621.6	625.6	294.3	1	4877										
37	FAHFA(18:2-O-17:0)	LAHHDA	619.6	623.6	294.3	1	4045										
38	FAHFA(15:1-O-18:0)	PDEAHSA	593.8	597.8	308.3	1	4645										
39	FAHFA(16:0-O-18:0)	PAHSA	609.8	613.8	308.3	11	93005	62445	25716	37020	39337	12093	11639	7514	4234	12149	46586
40	FAHFA(17:1-O-18:0)	HDEAHSA	621.8	625.8	308.3	1	2938										
41	FAHFA(18:0-O-18:0)	SAHSA	637.8	641.8	308.3	5	127117	95786	54030	51728	20785						
42	FAHFA(20:1-O-18:0)	EAHSA	663.8	667.8	308.3	1	21382										
43	FAHFA(20:2-O-18:0)	EDAHSA	661.8	665.8	308.3	1	8635										
44	FAHFA(22:1-O-18:0)	DEAHSA	691.8	695.8	308.3	1	16921										
45	FAHFA(22:2-O-18:0)	DDAHSA	689.8	693.8	308.3	1	9691										
46	FAHFA(18:2-O-19:0)	LAHNDA	647.8	651.8	322.3	4	1666	2019	10624	5330							
47	FAHFA(22:0-O-19:0)	BAHNDA	707.8	711.8	322.3	1	1910										
48	FAHFA(22:1-O-19:0)	DEAHNDA	705.8	709.8	322.3	1	2184										
49	FAHFA(14:1-O-20:0)	MOHAA	607.8	611.8	336.3	1	2958										
50	FAHFA(16:1-O-20:0)	POHAA	635.8	639.8	336.3	1	6760										
51	FAHFA(18:1-O-20:0)	OAHAA	663.8	667.8	336.3	1	151913										
52	FAHFA(20:1-O-20:0)	EAHAA	691.8	695.8	336.3	1	80705										
53	FAHFA(20:2-O-20:0)	EDAHAA	689.8	693.8	336.3	1	32387										
54	FAHFA(20:3-O-20:0)	ETAHAA	687.5	691.8	336.3	1	18312										
55	FAHFA(22:1-O-20:0)	DEAHAA	719.8	723.8	336.3	1	7705										
56	FAHFA(16:0-O-21:0)	PAHHEA	651.8	655.8	350.3	3	2259	5464	1960								

I, intensity (cps)

Peanut (*Arachis hypogaea*)

NO.	Normal name	FAHFA ID	*m/z* (DMED labeling)	*m/z* (d_4-DMED labeling)	Product ion	Regioisomer number	*I1*	*I2*	*I3*	*I4*
1	FAHFA(16:1-O-18:1)	POHOA	605.5	609.5	306.2	1	17919			
2	FAHFA(18:1-O-18:1)	OAHOA	633.5	637.5	306.2	1	86760			
3	FAHFA(18:2-O-18:1)	LAHOA	631.5	635.5	306.2	1	244517			
4	FAHFA(16:0-O-18:2)	PAHLA	605.5	609.5	304.2	3	25043	80320	9982	
5	FAHFA(18:1-O-18:2)	OAHLA	631.5	635.5	304.2	2	20690	115857		
6	FAHFA(18:2-O-18:2)	LAHLA	629.5	633.5	304.2	2	203977	730524		
7	FAHFA(18:2-O-18:3)	LAHALA	627.5	631.5	302.2	2	24688	19917		
8	FAHFA(16:0-O-16:0)	PAHPA	581.6	585.6	280.3	1	42645			
9	FAHFA(18:2-O-16:0)	LAHPA	605.6	609.6	280.3	1	215567			
10	FAHFA(16:1-O-18:0)	POHSA	607.5	611.8	308.3	1	47466			
11	FAHFA(18:1-O-18:0)	OAHSA	635.8	639.8	308.3	1	20050			
12	FAHFA(18:2-O-18:0)	LAHSA	633.8	637.8	308.3	1	30217			
13	FAHFA(18:3-O-18:0)	ALAHSA	631.8	635.8	308.3	1	21280			
14	FAHFA(16:0-O-19:0)	PAHNDA	623.8	627.8	322.3	4	46365	53725	47524	40650
15	FAHFA(18:1-O-19:0)	OAHNDA	649.8	653.8	322.3	4	35178	40162	102897	94689
16	FAHFA(18:2-O-19:0)	LAHNDA	647.8	651.8	322.3	4	205295	231532	334691	190400
17	FAHFA(16:1-O-20:0)	POHAA	635.8	639.8	336.3	1	8665			
18	FAHFA(16:0-O-20:0)	PAHAA	637.8	641.8	336.3	1	4768			

I, intensity (cps)

Black Sesame (*Sesamum indicum*)

NO.	Normal name	FAHFA ID	*m/z* (DMED labeling)	*m/z* (d_4-DMED labeling)	Product ion	Regioisomer number	*I1*	*I2*	*I3*	*I4*	*I5*	*I6*	*I7*	*I8*	*I9*
1	FAHFA(18:2-O-16:1)	LAHPO	603.5	607.5	278.2	1	2116								
2	FAHFA(18:1-O-18:1)	OAHOA	633.5	637.5	306.2	4	2403	1095	2560	1795					
3	FAHFA(18:2-O-18:1)	LAHOA	631.5	635.5	306.2	4	3406	5416	2688	9196					
4	FAHFA(16:0-O-18:2)	PAHLA	605.5	609.5	304.2	2	2225	1028							
5	FAHFA(18:2-O-18:2)	LAHLA	629.5	633.5	304.2	2	51558	7805							
6	FAHFA(18:2-O-20:2)	LAHEDA	657.5	661.5	332.2	2	4292	3729							
7	FAHFA(22:0-O-20:4)	BAHARA	713.5	717.5	328.2	1	2541								
8	FAHFA(18:1-O-14:0)	OAHMA	579.5	583.5	252.2	1	7534								
9	FAHFA(18:2-O-14:0)	LAHMA	577.5	581.5	252.2	1	4279								
10	FAHFA(18:1-O-16:0)	OAHPA	607.6	611.6	280.3	2	978	1386							
11	FAHFA(18:1-O-17:0)	OAHHDA	621.6	625.6	294.3	1	2618								
12	FAHFA(18:2-O-17:0)	LAHHDA	619.6	623.6	294.3	1	6874								
13	FAHFA(18:2-O-18:0)	LAHSA	633.8	637.8	308.3	1	4017								
14	FAHFA(16:0-O-19:0)	PAHNDA	623.8	627.8	322.3	3	19992	16283	11128						
15	FAHFA(18:1-O-19:0)	OAHNDA	649.8	653.8	322.3	9	5956	6390	4793	3600	71145	82261	65991	45441	3870
16	FAHFA(18:2-O-19:0)	LAHNDA	647.8	651.5	322.3	5	203951	220006	179369	128028	11049				

I, intensity (cps)

Wheat (*Triticum aestivum*)

NO.	Normal name	FAHFA ID	*m/z* (DMED labeling)	*m/z* (d_4-DMED labeling)	Product ion	Regioisomer number	*I1*	*I2*	*I3*	*I4*	*I5*	*I6*	*I7*	*I8*	*I9*	*I10*	*I11*	*I12*	*I13*	*I14*
1	FAHFA(18:2-O-14:1)	LAHMO	575.5	579.5	250.2	3	1821	2652	4372											
2	FAHFA(20:0-O-14:1)	AAHMO	607.5	611.5	250.2	1	3158													
3	FAHFA(20:1-O-14:1)	EAHMO	605.5	609.5	250.2	1	2856													
4	FAHFA(22:2-O-14:1)	DDAHMO	631.5	635.5	250.2	2	4374	2172												
5	FAHFA(18:2-O-14:2)	LAHTDA	573.5	577.5	248.2	1	5013													
6	FAHFA(20:0-O-14:2)	AAHTDA	605.5	609.5	248.2	2	41781	3731												
7	FAHFA(22:1-O-14:2)	DEAHTDA	631.5	635.5	248.2	3	2844	30653	2397											
8	FAHFA(22:2-O-14:2)	DDAHTDA	629.5	633.5	248.2	4	2862	2246	73614	10724										
9	FAHFA(22:6-O-14:2)	DHAHTDA	621.5	625.5	248.2	1	28313													
10	FAHFA(22:1-O-14:3)	DEAHTTA	629.5	633.5	246.2	2	4266	1548												
11	FAHFA(22:2-O-14:3)	DDAHTTA	627.5	631.5	246.2	2	4458	2825												
12	FAHFA(16:0-O-16:1)	PAHPO	579.5	583.5	246.2	4	6333	26528	9639	2966										
13	FAHFA(18:1-O-16:1)	OAHPO	605.5	609.5	246.2	3	10772	21437	5644											
14	FAHFA(18:2-O-16:1)	LAHPO	603.5	607.5	246.2	5	6391	70105	30178	18628	8332									
15	FAHFA(16:0-O-16:2)	PAHHDA	577.5	581.5	276.2	2	6080	2624												
16	FAHFA(18:0-O-16:2)	SAHHDA	605.6	609.6	276.2	2	14198	1170												
17	FAHFA(18:1-O-16:2)	OAHHDA	603.6	607.6	276.2	2	5741	3267												
18	FAHFA(18:2-O-16:2)	LAHHDA	601.5	605.5	276.2	2	11580	7181												
19	FAHFA(20:1-O-16:2)	EAHHDA	631.5	635.5	276.2	1	5937													
20	FAHFA(20:2-O-16:2)	EDAHHDA	629.5	633.5	276.2	2	17633	2771												
21	FAHFA(18:0-O-16:3)	SAHHTA	603.5	607.5	274.2	2	3366	3058												
22	FAHFA(20:1-O-16:3)	EAHHTA	629.5	633.5	274.2	2	7011	3142												
23	FAHFA(20:2-O-16:3)	EDAHHTA	637.5	641.5	274.2	2	11727	6161												
24	FAHFA(20:5-O-16:3)	EPAHHTA	621.5	625.5	274.2	2	9870	4463												
25	FAHFA(22:6-O-16:3)	DHAHHTA	647.5	651.5	274.2	1	11563													
26	FAHFA(14:0-O-18:1)	MAHOA	579.5	583.5	306.2	2	23837	21194												
27	FAHFA(16:1-O-18:1)	POHOA	605.5	609.5	306.2	4	34021	12516	166181	13088										
28	FAHFA(16:0-O-18:1)	PAHOA	607.5	613.5	306.2	5	729365	59500	101763	183152	373509									
29	FAHFA(18:1-O-18:1)	OAHOA	633.5	637.5	306.2	5	470825	47407	85043	137476	379158									
30	FAHFA(18:2-O-18:1)	LAHOA	631.5	635.5	306.2	5	646365	1610452	270627	266428	1395511									
31	FAHFA(20:1-O-18:1)	EAHOA	661.5	665.5	306.2	3	22134	21732	12466											
32	FAHFA(22:6-O-18:1)	DHAHOA	679.5	983.5	306.2	6	9841	9299	5309	5010	11224	13909								
33	FAHFA(14:0-O-18:2)	MAHLA	577.5	581.5	304.2	3	4260	90761	13195											
34	FAHFA(15:0-O-18:2)	PDAHLA	591.5	595.5	304.2	3	2544	36701	6377											
35	FAHFA(15:1-O-18:2)	PDEAHLA	589.5	593.5	304.2	2	31984	5772												
36	FAHFA(16:1-O-18:2)	POHLA	603.5	607.5	304.2	1	114245													
37	FAHFA(16:0-O-18:2)	PAHLA	605.5	609.5	304.2	2	8825645	674532												
38	FAHFA(17:1-O-18:2)	HDEAHLA	617.5	621.5	304.2	1	24361													
39	FAHFA(18:1-O-18:2)	OAHLA	631.5	635.5	304.2	5	564868	120567	139896	8693986	630110									
40	FAHFA(18:2-O-18:2)	LAHLA	629.5	633.5	304.2	3	471771	20000000	3225267											
41	FAHFA(20:0-O-18:2)	AAHLA	661.5	665.5	304.2	2	18787	62248												
42	FAHFA(20:1-O-18:2)	EAHLA	659.5	663.5	304.2	2	562056	31277												
43	FAHFA(20:2-O-18:2)	EDAHLA	657.5	661.5	304.2	1	17579													
44	FAHFA(22:0-O-18:2)	BAHLA	689.5	693.5	304.2	1	24938													
45	FAHFA(22:1-O-18:2)	DEAHLA	687.5	691.5	304.2	2	58566	4188												
46	FAHFA(22:6-O-18:2)	DHAHLA	677.5	681.5	304.2	6	45188	56126	252012	320176	12916	29005								
47	FAHFA(14:0-O-18:3)	MAHALA	575.5	579.5	302.2	2	2450	2217												
48	FAHFA(16:0-O-18:3)	PAHALA	603.5	607.5	302.2	3	6651	171369	159849											
49	FAHFA(18:1-O-18:3)	OAHALA	629.5	633.5	302.2	4	23344	13966	184994	175063										
50	FAHFA(18:2-O-18:3)	LAHALA	627.5	631.5	302.2	2	849215	504162												
51	FAHFA(20:1-O-18:3)	EAHALA	657.5	661.5	302.2	1	7851													
52	FAHFA(22:6-O-18:3)	DHAHALA	675.5	679.5	302.2	3	8158	65161	23704											
53	FAHFA(18:2-O-20:1)	LAHEA	659.5	663.5	334.2	5	45677	4638	2076	3483	5095									
54	FAHFA(16:0-O-20:2)	PAHEDA	633.5	637.5	332.2	3	10949	2404	1183											
55	FAHFA(18:1-O-20:2)	OAHEDA	659.5	663.5	332.2	1	10255													
56	FAHFA(18:2-O-20:2)	LAHEDA	657.5	661.5	332.2	1	54918													
57	FAHFA(16:0-O-22:6)	PAHDHA	653.5	657.5	352.2	3	4906	20579	33456											
58	FAHFA(18:1-O-22:6)	OAHDHA	679.5	683.5	352.2	6	5324	2879	2801	2754	33090	36054								
59	FAHFA(18:3-O-22:6)	ALAHDHA	675.5	679.5	352.2	2	4193	6446												
60	FAHFA(16:0-O-12:0)	PAHDA	525.5	529.5	224.2	2	5616	2899												
61	FAHFA(18:2-O-12:0)	LAHDA	549.5	553.5	224.2	5	6172	1980	1092	4929	1508									
62	FAHFA(22:2-O-12:0)	DDAHDA	605.5	609.5	224.2	1	10617													
63	FAHFA(14:0-O-14:0)	MAHMA	525.5	529.5	252.2	1	27540													
64	FAHFA(16:0-O-14:0)	PAHMA	553.5	557.5	252.2	9	14118	8791	2323	3302	5030	12982	10203	9667	4364					
65	FAHFA(18:0-O-14:0)	SAHMA	581.5	586.5	252.2	1	7649													
66	FAHFA(18:1-O-14:0)	OAHMA	579.5	583.5	252.2	7	7325	7125	4508	3016	4694	8874	26275							
67	FAHFA(18:2-O-14:0)	LAHMA	577.5	581.5	252.2	8	25331	14014	4590	7753	7300	44364	115560	8243						
68	FAHFA(18:3-O-14:0)	ALAHMA	575.5	579.5	252.2	3	9335	3027	11346											
69	FAHFA(16:0-O-15:0)	PAHPDA	567.5	571.5	266.2	5	5215	5693	4033	5783	5101									
70	FAHFA(18:1-O-15:0)	OAHPDA	593.5	597.5	266.2	5	4945	3343	4366	4058	3310									
71	FAHFA(18:2-O-15:0)	LAHPDA	591.5	595.5	266.2	6	6667	12656	10965	12935	18849	8559								
72	FAHFA(14:0-O-16:0)	MAHPA	553.6	557.6	280.3	5	6664	3052	9887	1828	8011									
73	FAHFA(15:0-O-16:0)	PDAHPA	567.6	571.6	280.3	3	3485	5550	13444											
74	FAHFA(15:1-O-16:0)	PDEAHPA	565.6	569.6	280.3	1	5314													
75	FAHFA(16:1-O-16:0)	POHPA	579.6	583.6	280.3	3	7110	5368	47833											
76	FAHFA(16:0-O-16:0)	PAHPA	581.6	585.6	280.3	3	158226	136443	3289441											
77	FAHFA(17:0-O-16:0)	HDAHPA	595.6	599.6	280.3	3	5410	7621	12400											
78	FAHFA(17:1-O-16:0)	HDEAHPA	593.6	597.6	280.3	1	7561													
79	FAHFA(18:1-O-16:0)	OAHPA	607.6	611.6	280.3	8	335283	121880	59920	3199997	35527	53165	24464	38766						
80	FAHFA(18:2-O-16:0)	LAHPA	605.6	609.6	280.3	6	350703	152262	185822	9392453	155512	263903								
81	FAHFA(18:3-O-16:0)	ALAHPA	603.6	607.6	280.3	1	419235													
82	FAHFA(20:1-O-16:0)	EAHPA	635.6	639.6	280.3	1	66817													
83	FAHFA(20:2-O-16:0)	EDAHPA	633.6	637.6	280.3	1	11723													
84	FAHFA(20:3-O-16:0)	ETAHPA	663.6	667.6	280.3	1	7579													
85	FAHFA(18:0-O-17:0)	SAHHDA	623.6	627.6	294.3	2	30716	25254												
86	FAHFA(18:2-O-17:0)	LAHHDA	619.6	623.6	294.3	8	39648	15886	9260	12389	1266	12596	12678	5798						
87	FAHFA(20:1-O-17:0)	EAHHDA	649.6	653.6	294.3	4	12603	11187	2774	2515										
88	FAHFA(20:2-O-17:0)	EDAHHDA	647.6	651.6	294.3	4	27520	25720	6840	6445										
89	FAHFA(16:1-O-18:0)	POHSA	607.8	611.8	308.3	1	19373													
90	FAHFA(16:0-O-18:0)	PAHSA	609.8	613.8	308.3	8	5344	2233	2787	12356	21776	11377	12710	34841						
91	FAHFA(18:1-O-18:0)	OAHSA	635.8	639.8	308.3	12	6112	9881	18404	13672	14754	40453	4720	2340	2472	1912	4683	2947		
92	FAHFA(18:2-O-18:0)	LAHSA	633.8	637.8	308.3	14	19788	23952	8477	5094	29089	37480	23973	90329	8460	5165	2525	5211	9083	14714
93	FAHFA(14:0-O-19:0)	MAHNDA	595.8	599.8	322.3	4	51174	51540	11813	13464										
94	FAHFA(15:0-O-19:0)	PDAHNDA	609.8	613.8	322.3	4	33539	42515	15772	13031										
95	FAHFA(15:1-O-19:0)	PDEAHNDA	607.8	613.8	322.3	2	10715	14044												
96	FAHFA(16:1-O-19:0)	POHNDA	621.8	625.8	322.3	4	180178	248428	38724	34544										
97	FAHFA(16:0-O-19:0)	PAHNDA	623.8	627.8	322.3	4	8863690	7548747	1174033	1363404										
98	FAHFA(17:0-O-19:0)	HDAHNDA	637.8	641.8	322.3	4	35440	48048	11981	10027										
99	FAHFA(17:1-O-19:0)	HDEAHNDA	653.8	657.8	322.3	3	26529	27579	8887											
100	FAHFA(18:1-O-19:0)	OAHNDA	649.8	653.8	322.3	6	833448	530213	7636548	9415112	1140445	965864								
101	FAHFA(18:2-O-19:0)	LAHNDA	647.8	651.8	322.3	4	19819256	19957869	4559724	3490490										
102	FAHFA(18:3-O-19:0)	ALAHNDA	645.8	649.8	322.3	4	2562593	1580106	836029	503546										
103	FAHFA(20:0-O-19:0)	AAHNDA	679.8	683.8	322.3	6	20774	23372	76819	50955	19909	6230								
104	FAHFA(20:1-O-19:0)	EAHNDA	677.8	681.8	322.3	6	14383	14761	522581	540341	38400	50302								
105	FAHFA(20:2-O-19:0)	EDAHNDA	675.8	679.8	322.3	6	36223	33244	53347	62767	9950	6283								
106	FAHFA(22:0-O-19:0)	BAHNDA	707.8	711.8	322.3	7	12444	4625	4785	78598	70381	11636	10088							
107	FAHFA(22:1-O-19:0)	DEAHNDA	705.8	709.8	322.3	6	17797	7264	101620	26017	11787	8001								
108	FAHFA(22:6-O-19:0)	DHAHNDA	695.8	699.8	322.3	1	13993													
109	FAHFA(18:2-O-20:0)	LAHAA	661.8	665.8	336.3	3	23623	23323	36964											
110	FAHFA(16:0-O-21:0)	PAHHEA	651.8	655.8	350.3	1	53476													
111	FAHFA(18:1-O-21:0)	OAHHEA	677.8	681.8	350.3	1	47197													
112	FAHFA(18:2-O-21:0)	LAHHEA	675.8	679.8	350.3	3	104014	6307	5989											

I, intensity (cps)

Rice (*Oryza sativa*)

NO.	Normal name	FAHFA ID	*m/z* (DMED labeling)	*m/z* (d_4-DMED labeling)	Product ion	Regioisomer number	*I1*	*I2*	*I3*	*I4*	*I5*	*I6*	*I7*
1	FAHFA(22:1-O-14:2)	DEAHTDA	631.5	635.5	248.2	1	2020						
2	FAHFA(22:2-O-14:2)	DDAHTDA	629.5	633.5	248.2	1	3320						
3	FAHFA(20:0-O-16:1)	AAHPO	635.5	639.5	278.2	1	1919						
4	FAHFA(14:0-O-18:1)	MAHOA	579.5	583.5	306.2	3	9633	3641	3240				
5	FAHFA(16:0-O-18:1)	PAHOA	607.5	613.5	306.2	3	84921	7243	31156				
6	FAHFA(18:1-O-18:1)	OAHOA	633.5	637.5	306.2	4	115502	7425	7937	60959			
7	FAHFA(18:2-O-18:1)	LAHOA	631.5	635.5	306.2	5	219079	30253	12747	81980	80266		
8	FAHFA(14:0-O-18:2)	MAHLA	577.5	581.5	304.2	2	80959	5224					
9	FAHFA(16:0-O-18:2)	PAHLA	605.5	609.5	304.2	3	4996	147577	26935				
10	FAHFA(18:1-O-18:2)	OAHLA	631.5	635.5	304.2	5	20433	7799	11977	389253	42599		
11	FAHFA(18:2-O-18:2)	LAHLA	629.5	633.5	304.2	3	31065	743088	126570				
12	FAHFA(16:0-O-18:3)	PAHALA	603.5	607.5	302.2	2	3885	2716					
13	FAHFA(18:1-O-18:3)	OAHALA	629.5	633.5	302.2	2	10291	6569					
14	FAHFA(18:2-O-18:3)	LAHALA	627.5	631.5	302.2	2	24414	16533					
15	FAHFA(18:1-O-22:6)	OAHDHA	679.5	683.5	352.2	2	2555	1710					
16	FAHFA(14:0-O-14:0)	MAHMA	525.5	529.5	252.2	1	12277						
17	FAHFA(14:0-O-16:0)	MAHPA	553.6	557.6	280.3	1	2432						
18	FAHFA(16:0-O-16:0)	PAHPA	581.6	585.6	280.3	2	17976	17248					
19	FAHFA(18:1-O-16:0)	OAHPA	607.6	611.6	280.3	2	33061	24788					
20	FAHFA(18:2-O-16:0)	LAHPA	605.6	609.6	280.3	7	69460	5445	3305	49049	6946	5748	2661
21	FAHFA(18:3-O-16:0)	ALAHPA	603.6	607.6	280.3	2	3260	3287					
22	FAHFA(20:2-O-17:0)	EDAHHDA	647.6	651.6	294.3	2	2178	1606					
23	FAHFA(16:1-O-18:0)	POHSA	607.8	611.8	308.3	1	1675						
24	FAHFA(18:2-O-18:0)	LAHSA	633.8	637.8	308.3	5	4835	4096	2877	3859	5189		
25	FAHFA(14:0-O-19:0)	MAHNDA	595.8	599.8	322.3	4	5551	3591	14616	11937			
26	FAHFA(16:1-O-19:0)	POHNDA	621.8	625.8	322.3	2	9266	14130					
27	FAHFA(16:0-O-19:0)	PAHNDA	623.8	627.8	322.3	5	7079	41617	46842	207073	168641		
28	FAHFA(18:1-O-19:0)	OAHNDA	649.8	653.8	322.3	5	40748	144140	149236	476579	370962		
29	FAHFA(18:2-O-19:0)	LAHNDA	647.8	651.8	322.3	5	21099	207370	215589	1421650	768640		
30	FAHFA(18:3-O-19:0)	ALAHNDA	645.8	649.8	322.3	2	91669	65716					

I, intensity (cps)

Egg

NO.	Normal name	FAHFA ID	*m/z* (DMED labeling)	*m/z* (d_4-DMED labeling)	Product ion	Regioisomer number	*I1*	*I2*	*I3*	*I4*	*I5*	*I6*	*I7*	*I8*	*I9*	*I10*
1	FAHFA(16:0-O-14:1)	PAHMO	551.5	555.5	250.2	1	13330									
2	FAHFA(18:0-O-14:1)	SAHMO	579.5	583.5	250.2	2	1190	5376								
3	FAHFA(18:1-O-14:1)	OAHMO	577.5	581.5	250.2	2	7514	3143								
4	FAHFA(18:2-O-14:1)	LAHMO	575.5	579.5	250.2	1	3247									
5	FAHFA(16:0-O-14:2)	PAHTDA	549.5	553.5	248.2	1	769581									
6	FAHFA(17:0-O-14:2)	HDAHTDA	563.5	567.5	248.2	1	3785									
7	FAHFA(18:0-O-14:2)	SAHTDA	577.5	581.5	248.2	3	16917	67111	296083							
8	FAHFA(18:1-O-14:2)	OAHTDA	575.5	579.5	248.2	1	469668									
9	FAHFA(20:0-O-14:2)	AAHTDA	605.5	609.5	248.2	2	870	6874								
10	FAHFA(20:1-O-14:2)	EAHTDA	603.5	608.5	248.2	1	8434									
11	FAHFA(22:6-O-14:2)	DHAHTDA	621.5	625.5	248.2	1	10237									
12	FAHFA(18:2-O-18:2)	LAHLA	629.5	633.5	304.2	1	5144									
13	FAHFA(16:0-O-12:0)	PAHDA	525.5	529.5	224.2	1	2735									
14	FAHFA(15:1-O-15:0)	PDEAHPDA	551.5	555.5	266.2	1	14417									
15	FAHFA(17:1-O-15:0)	HDEAHPDA	579.5	583.5	266.2	1	12570									
16	FAHFA(22:1-O-15:0)	DEAHPDA	649.5	653.5	266.2	1	26118									
17	FAHFA(16:0-O-16:0)	PAHPA	581.6	585.6	280.3	10	2888	1663	2225	1505	1004	3516	1569	1833	3845	7919
18	FAHFA(18:0-O-16:0)	SAHPA	609.6	613.6	280.3	5	1680	1540	2510	1723	3103					
19	FAHFA(18:1-O-16:0)	OAHPA	607.6	613.6	280.3	3	2123	2445	5677							
20	FAHFA(16:0-O-18:0)	PAHSA	609.8	613.8	308.3	8	1717	1743	1971	1045	3124	2209	3208	1630		
21	FAHFA(18:0-O-18:0)	SAHSA	637.8	641.8	308.3	5	4625	3065	2906	2252	1318					
22	FAHFA(18:1-O-18:0)	OAHSA	635.8	639.8	308.3	4	2464	1504	4067	1349						
23	FAHFA(18:1-O-19:0)	OAHNDA	649.8	653.8	322.3	2	2512	3114								
24	FAHFA(18:1-O-20:0)	OAHAA	663.8	667.8	336.3	1	4954									
25	FAHFA(20:1-O-20:0)	EAHAA	691.8	695.8	336.3	1	3743									
26	FAHFA(20:2-O-20:0)	EDAHAA	689.8	693.8	336.3	1	4249									

I, intensity (cps)

Fish (*Carassius auratus*)

NO.	Normal name	FAHFA ID	*m/z* (DMED labeling)	*m/z* (d_4-DMED labeling)	Product ion	Regioisomer number	*I1*	*I2*	*I3*	*I4*	*I5*	*I6*	*I7*	*I8*	*I9*	*I10*	*I11*
1	FAHFA(18:3-O-14:1)	ALAHMO	573.5	577.5	250.2	1	2580										
2	FAHFA(18:3-O-14:2)	ALAHTDA	571.5	575.5	248.2	1	7146										
3	FAHFA(15:1-O-20:1)	PDEAHEA	619.5	623.5	334.2	1	3820										
4	FAHFA(17:1-O-20:1)	HDEAHEA	647.5	651.5	334.2	1	5202										
5	FAHFA(20:1-O-20:1)	EAHEA	689.5	693.5	334.2	1	7348										
6	FAHFA(22:1-O-20:1)	DEAHEA	717.5	721.5	334.2	2	1871	3914									
7	FAHFA(22:2-O-20:1)	DDAHEA	715.5	719.5	334.2	1	1834										
8	FAHFA(15:1-O-20:2)	PDEAHEDA	617.5	621.5	332.2	1	112831										
9	FAHFA(17:1-O-20:2)	HDEAHEDA	645.5	649.5	332.2	1	136142										
10	FAHFA(18:2-O-20:2)	LAHEDA	657.5	661.5	332.2	2	6028	23823									
11	FAHFA(18:4-O-20:2)	SDAHEDA	653.5	657.5	332.2	1	3313										
12	FAHFA(20:0-O-20:2)	AAHEDA	689.5	693.5	332.2	3	70017	91719	4758								
13	FAHFA(20:1-O-20:2)	EAHEDA	687.5	691.5	332.2	2	31766	155145									
14	FAHFA(22:1-O-20:2)	DEAHEDA	715.5	719.5	332.2	3	43983	19401	64958								
15	FAHFA(22:2-O-20:2)	DDAHEDA	713.5	717.5	332.2	1	34714										
16	FAHFA(16:0-O-12:0)	PAHDA	525.5	529.5	224.2	1	1826										
17	FAHFA(22:6-O-12:0)	DHAHDA	597.5	601.5	224.2	1	2043										
18	FAHFA(16:0-O-14:0)	PAHMA	553.5	557.5	252.2	1	1940										
19	FAHFA(22:6-O-14:0)	DHAHMA	625.5	629.5	252.2	1	2444										
20	FAHFA(16:0-O-16:0)	PAHPA	581.6	585.6	280.3	11	3183	1722	1118	900	1419	1265	1774	957	957	1615	10172
21	FAHFA(18:0-O-16:0)	SAHPA	609.6	613.6	280.3	2	1340	6344									
22	FAHFA(18:1-O-16:0)	OAHPA	607.6	611.6	280.3	1	2294										
23	FAHFA(22:6-O-16:0)	DHAHPA	653.6	657.6	280.3	2	12149	5373									
24	FAHFA(18:1-O-18:0)	OAHSA	635.8	639.8	308.3	1	2293										
25	FAHFA(22:6-O-18:0)	DHAHSA	681.8	685.8	308.3	2	3298	1152									

I, intensity (cps)
