# Supplementary material for: A dataset of branched fatty acid esters of hydroxy fatty acids diversity in foods
Source: Sci Data. 2023 Nov 10;10:790. doi: 10.1038/s41597-023-02712-z (PMC10638281; doi:10.1038/s41597-023-02712-z)
Supplement: Supplementary file 7 — Supplementary information-8 Table S8. Identified FAHFA regioisomers list [file 41597_2023_2712_MOESM7_ESM.pdf]

Supplementary Table S8. Identified FAHFA regioisomers list

NO.	FAHFAs	MRM transition (DMED labeling)	MRM transition (d_4-DMED labeling)	Retention index (DMED lableing)	Monoisotopic Mass （unlabeled）	Molecular Formula	SMILES	InChIKey
1	12-MAHMA	525.5→252.2	529.5→252.2	2149	454.4022	C28H54O4	CCCCCCCCCCCCCC(=O)OC(CC)CCCCCCCCCCC(=O)O	HJJGZLHKJLSGLK-UHFFFAOYSA-N
2	11-MAHMA	525.5→252.2	529.5→252.2	2163	454.4022	C28H54O4	CCCCCCCCCCCCCC(=O)OC(CCC)CCCCCCCCCC(=O)O	VZSCHBMOABJRPX-UHFFFAOYSA-N
3	10-MAHMA	525.5→252.2	529.5→252.2	2196	454.4022	C28H54O4	CCCCCCCCCCCCCC(=O)OC(CCCC)CCCCCCCCC(=O)O	PEOJOXYCMSXYRI-UHFFFAOYSA-N
4	9-MAHMA	525.5→252.2	529.5→252.2	2220	454.4022	C28H54O4	CCCCCCCCCCCCCC(=O)OC(CCCCC)CCCCCCCC(=O)O	IHKNEWAXALARRP-UHFFFAOYSA-N
5	8-MAHMA	525.5→252.2	529.5→252.2	2251	454.4022	C28H54O4	CCCCCCCCCCCCCC(=O)OC(CCCCCC)CCCCCCC(=O)O	WMOXMSBRUFIUPL-UHFFFAOYSA-N
6	7-MAHMA	525.5→252.2	529.5→252.2	2278	454.4022	C28H54O4	CCCCCCCCCCCCCC(=O)OC(CCCCCCC)CCCCCC(=O)O	ZYOPGQRZKXBEKB-UHFFFAOYSA-N
7	6-MAHMA	525.5→252.2	529.5→252.2	2307	454.4022	C28H54O4	CCCCCCCCCCCCCC(=O)OC(CCCCCCCC)CCCCC(=O)O	GWEDXXWKMASNKO-UHFFFAOYSA-N
8	5-MAHMA	525.5→252.2	529.5→252.2	2336	454.4022	C28H54O4	CCCCCCCCCCCCCC(=O)OC(CCCCCCCCC)CCCC(=O)O	ODCLJRIUYLLLRH-UHFFFAOYSA-N
9	4-MAHMA	525.5→252.2	529.5→252.2	2370	454.4022	C28H54O4	CCCCCCCCCCCCCC(=O)OC(CCCCCCCCCC)CCC(=O)O	XDLPIAAUTXLIFM-UHFFFAOYSA-N
10	3-MAHMA	525.5→252.2	529.5→252.2	2391	454.4022	C28H54O4	CCCCCCCCCCCCCC(=O)OC(CCCCCCCCCCC)CC(=O)O	IYESSKQVAKTOQB-UHFFFAOYSA-N
11	11-PAHMA	553.5→252.2	557.5→252.2	2352	482.4335	C30H58O4	CCCCCCCCCCCCCCCC(=O)OC(CCC)CCCCCCCCCC(=O)O	BRHYNQKPLVBLHW-UHFFFAOYSA-N
12	10-PAHMA	553.5→252.2	557.5→252.2	2393	482.4335	C30H58O4	CCCCCCCCCCCCCCCC(=O)OC(CCCC)CCCCCCCCC(=O)O	DYQFPWPFIGGWSX-UHFFFAOYSA-N
13	9-PAHMA	553.5→252.2	557.5→252.2	2418	482.4335	C30H58O4	CCCCCCCCCCCCCCCC(=O)OC(CCCCC)CCCCCCCC(=O)O	ADZIVQSYWSLHHX-UHFFFAOYSA-N
14	8-PAHMA	553.5→252.2	557.5→252.2	2455	482.4335	C30H58O4	CCCCCCCCCCCCCCCC(=O)OC(CCCCCC)CCCCCCC(=O)O	UQSDBWUCIRXRDO-UHFFFAOYSA-N
15	7-PAHMA	553.5→252.2	557.5→252.2	2484	482.4335	C30H58O4	CCCCCCCCCCCCCCCC(=O)OC(CCCCCCC)CCCCCC(=O)O	HIVSIVNAOGNSPG-UHFFFAOYSA-N
16	6-PAHMA	553.5→252.2	557.5→252.2	2515	482.4335	C30H58O4	CCCCCCCCCCCCCCCC(=O)OC(CCCCCCCC)CCCCC(=O)O	DEHAXENVRQJDNJ-UHFFFAOYSA-N
17	5-PAHMA	553.5→252.2	557.5→252.2	2536	482.4335	C30H58O4	CCCCCCCCCCCCCCCC(=O)OC(CCCCCCCCC)CCCC(=O)O	GZSCEQSBGJUGAA-UHFFFAOYSA-N
18	4-PAHMA	553.5→252.2	557.5→252.2	2572	482.4335	C30H58O4	CCCCCCCCCCCCCCCC(=O)OC(CCCCCCCCCC)CCC(=O)O	FWJFGEWJLTWRFD-UHFFFAOYSA-N
19	3-PAHMA	553.5→252.2	557.5→252.2	2601	482.4335	C30H58O4	CCCCCCCCCCCCCCCC(=O)OC(CCCCCCCCCCC)CC(=O)O	LBKJVDBQLKKIPF-UHFFFAOYSA-N
20	3-PDAHMA	539.5→252.2	543.5→252.2	2504	468.4179	C29H56O4	CCCCCCCCCCCCCCC(=O)OC(CCCCCCCCCCC)CC(=O)O	HRGHHUSGRYOUQT-UHFFFAOYSA-N
21	4-PDAHMA	539.5→252.2	543.5→252.2	2474	468.4179	C29H56O4	CCCCCCCCCCCCCCC(=O)OC(CCCCCCCCCC)CCC(=O)O	MUQOEFFRAWBQQK-UHFFFAOYSA-N
22	5-PDAHMA	539.5→252.2	543.5→252.2	2447	468.4179	C29H56O4	CCCCCCCCCCCCCCC(=O)OC(CCCCCCCCC)CCCC(=O)O	GAOLFPUOZNZNNM-UHFFFAOYSA-N
23	10-SAHMA	581.5→252.5	585.5→252.5	2583	510.4648	C32H62O4	CCCCCCCCCCCCCCCCCC(=O)OC(CCCC)CCCCCCCCC(=O)O	YTSABFXBGKBOPQ-UHFFFAOYSA-N
24	9-SAHMA	581.5→252.5	585.5→252.5	2608	510.4648	C32H62O4	CCCCCCCCCCCCCCCCCC(=O)OC(CCCCC)CCCCCCCC(=O)O	QOKJPCRPQUZMKK-UHFFFAOYSA-N
25	5-SAHMA	581.5→252.5	585.5→252.5	2720	510.4648	C32H62O4	CCCCCCCCCCCCCCCCCC(=O)OC(CCCCCCCCC)CCCC(=O)O	ATXZYOLUWOGARZ-UHFFFAOYSA-N
26	4-SAHMA	581.5→252.5	585.5→252.5	2754	510.4648	C32H62O4	CCCCCCCCCCCCCCCCCC(=O)OC(CCCCCCCCCC)CCC(=O)O	FPPDMZVFWADWLL-UHFFFAOYSA-N
27	3-SAHMA	581.5→252.5	585.5→252.5	2784	510.4648	C32H62O4	CCCCCCCCCCCCCCCCCC(=O)OC(CCCCCCCCCCC)CC(=O)O	JXDNKRJCONJZEL-UHFFFAOYSA-N
28	3-MAHPA	553.6→280.3	557.6→280.3	2601	482.4335	C30H58O4	CCCCCCCCCCCCCC(CC(=O)O)OC(=O)CCCCCCCCCCCCC	VSFGVNXNEZONDG-UHFFFAOYSA-N
29	4-MAHPA	553.6→280.3	557.6→280.3	2573	482.4335	C30H58O4	CCCCCCCCCCCCCC(=O)OC(CCCCCCCCCCCC)CCC(=O)O	RAZXBIYAGBKCQN-UHFFFAOYSA-N
30	5-MAHPA	553.6→280.3	557.6→280.3	2538	482.4335	C30H58O4	CCCCCCCCCCCCCC(=O)OC(CCCCCCCCCCC)CCCC(=O)O	RDMQIABVBPGPQO-UHFFFAOYSA-N
31	6-MAHPA	553.6→280.3	557.6→280.3	2510	482.4335	C30H58O4	CCCCCCCCCCCCCC(=O)OC(CCCCCCCCCC)CCCCC(=O)O	JWRNJBQCCCLVLW-UHFFFAOYSA-N
32	7-MAHPA	553.6→280.3	557.6→280.3	2479	482.4335	C30H58O4	CCCCCCCCCCCCCC(=O)OC(CCCCCCCCC)CCCCCC(=O)O	BZFVPEAOYDLDER-UHFFFAOYSA-N
33	8-MAHPA	553.6→280.3	557.6→280.3	2447	482.4335	C30H58O4	CCCCCCCCCCCCCC(=O)OC(CCCCCCCC)CCCCCCC(=O)O	WSWGTPCCNLTBHI-UHFFFAOYSA-N
34	9-MAHPA	553.6→280.3	557.6→280.3	2413	482.4335	C30H58O4	CCCCCCCCCCCCCC(=O)OC(CCCCCCC)CCCCCCCC(=O)O	SNRHTFPCAKXVHV-UHFFFAOYSA-N
35	10-MAHPA	553.6→280.3	557.6→280.3	2385	482.4335	C30H58O4	CCCCCCCCCCCCCC(=O)OC(CCCCCC)CCCCCCCCC(=O)O	LOJDMJNXHRPEJR-UHFFFAOYSA-N
36	11-MAHPA	553.6→280.3	557.6→280.3	2366	482.4335	C30H58O4	CCCCCCCCCCCCCC(=O)OC(CCCCC)CCCCCCCCCC(=O)O	XGUHYROOAJXDNG-UHFFFAOYSA-N
37	12-MAHPA	553.6→280.3	557.6→280.3	2334	482.4335	C30H58O4	CCCCCCCCCCCCCC(=O)OC(CCCC)CCCCCCCCCCC(=O)O	BNMKJLAEHWAAFS-UHFFFAOYSA-N
38	3-PDAHPA	567.6→280.3	571.6→280.3	2691	496.4492	C31H60O4	CCCCCCCCCCCCCCC(=O)OC(CCCCCCCCCCCCC)CC(=O)O	YIMMRWKQOFEMSN-UHFFFAOYSA-N
39	4-PDAHPA	567.6→280.3	571.6→280.3	2664	496.4492	C31H60O4	CCCCCCCCCCCCCCC(=O)OC(CCCCCCCCCCCC)CCC(=O)O	ZIRJUDKHWFJWFJ-UHFFFAOYSA-N
40	5-PDAHPA	567.6→280.3	571.6→280.3	2635	496.4492	C31H60O4	CCCCCCCCCCCCCCC(=O)OC(CCCCCCCCCCC)CCCC(=O)O	IOOQLHSIAOWEQT-UHFFFAOYSA-N
41	6-PDAHPA	567.6→280.3	571.6→280.3	2600	496.4492	C31H60O4	CCCCCCCCCCCCCCC(=O)OC(CCCCCCCCCC)CCCCC(=O)O	QQLBPLLTVHQKJU-UHFFFAOYSA-N
42	7-PDAHPA	567.6→280.3	571.6→280.3	2578	496.4492	C31H60O4	CCCCCCCCCCCCCCC(=O)OC(CCCCCCCCC)CCCCCC(=O)O	JMYMQUBOBJDKHE-UHFFFAOYSA-N
43	9-PDAHPA	567.6→280.3	571.6→280.3	2510	496.4492	C31H60O4	CCCCCCCCCCCCCCC(=O)OC(CCCCCCC)CCCCCCCC(=O)O	KJHFLWNKUPFGKA-UHFFFAOYSA-N
44	10-PDAHPA	567.6→280.3	571.6→280.3	2483	496.4492	C31H60O4	CCCCCCCCCCCCCCC(=O)OC(CCCCCC)CCCCCCCCC(=O)O	CNZXEKAPQAJBTK-UHFFFAOYSA-N
45	12-PDAHPA	567.6→280.3	571.6→280.3	2423	496.4492	C31H60O4	CCCCCCCCCCCCCCC(=O)OC(CCCC)CCCCCCCCCCC(=O)O	TUKDDXXAXBOFLN-UHFFFAOYSA-N
46	3-PAHPA	581.6→280.3	585.6→280.3	2787	510.4648	C32H62O4	CCCCCCCCCCCCCCCC(=O)OC(CCCCCCCCCCCCC)CC(=O)O	QXMZBFFWEBPYSW-UHFFFAOYSA-N
47	4-PAHPA	581.6→280.3	585.6→280.3	2748	510.4648	C32H62O4	CCCCCCCCCCCCCCCC(=O)OC(CCCCCCCCCCCC)CCC(=O)O	ULCMMLZLJPIJGI-UHFFFAOYSA-N
48	5-PAHPA	581.6→280.3	585.6→280.3	2723	510.4648	C32H62O4	CCCCCCCCCCCCCCCC(=O)OC(CCCCCCCCCCC)CCCC(=O)O	KBWQFRVOCVTZTO-UHFFFAOYSA-N
49	6-PAHPA	581.6→280.3	585.6→280.3	2696	510.4648	C32H62O4	CCCCCCCCCCCCCCCC(=O)OC(CCCCCCCCCC)CCCCC(=O)O	FCYNAPQNVXGTPE-UHFFFAOYSA-N
50	7-PAHPA	581.6→280.3	585.6→280.3	2664	510.4648	C32H62O4	CCCCCCCCCCCCCCCC(=O)OC(CCCCCCCCC)CCCCCC(=O)O	HKXPTEIGDMWSCZ-UHFFFAOYSA-N
51	8-PAHPA	581.6→280.3	585.6→280.3	2637	510.4648	C32H62O4	CCCCCCCCCCCCCCCC(=O)OC(CCCCCCCC)CCCCCCC(=O)O	JEOXCIJAORYAMX-UHFFFAOYSA-N
52	9-PAHPA	581.6→280.3	585.6→280.3	2603	510.4648	C32H62O4	CCCCCCCCCCCCCCCC(=O)OC(CCCCCCC)CCCCCCCC(=O)O	DPFVDUJFYPYAOO-UHFFFAOYSA-N

53	10-PAHPA	581.6→280.3	585.6→280.3	2576	510.4648	$C_{32}H_{62}O_4$	CCCCCCCCCCCCCCCC(=O)OC(CCCCCC)CCCCCCCCC(=O)O	WWOQHWCTUBCRSA-UHFFFAOYSA-N
54	11-PAHPA	581.6→280.3	585.6→280.3	2545	510.4648	$C_{32}H_{62}O_4$	CCCCCCCCCCCCCCCC(=O)OC(CCCCC)CCCCCCCCCC(=O)O	JBFRCUPOANWJTN-UHFFFAOYSA-N
55	12-PAHPA	581.6→280.3	585.6→280.3	2515	510.4648	$C_{32}H_{62}O_4$	CCCCCCCCCCCCCCCC(=O)OC(CCCC)CCCCCCCCCCC(=O)O	HXDZEKSDFMQDDU-UHFFFAOYSA-N
56	13-PAHPA	581.6→280.3	585.6→280.3	2498	510.4648	$C_{32}H_{62}O_4$	CCCCCCCCCCCCCCCC(=O)OC(CCC)CCCCCCCCCCCC(=O)O	IHNHFVFOGDZMEW-UHFFFAOYSA-N
57	3-HDAHPA	595.6→280.3	599.6→280.3	2874	524.4805	$C_{33}H_{64}O_4$	CCCCCCCCCCCCCCCCC(=O)OC(CCCCCCCCCCCCC)CC(=O)O	DZOMUETWDKRLPQ-UHFFFAOYSA-N
58	4-HDAHPA	595.6→280.3	599.6→280.3	2845	525.4805	$C_{33}H_{64}O_4$	CCCCCCCCCCCCCCCCC(=O)OC(CCCCCCCCCCCC)CCC(=O)O	FWKJDDGIUKZEIO-UHFFFAOYSA-N
59	5-HDAHPA	595.6→280.3	599.6→280.3	2815	526.4805	$C_{33}H_{64}O_4$	CCCCCCCCCCCCCCCCC(=O)OC(CCCCCCCCCCC)CCCC(=O)O	OFBQQWSTVAIXMA-UHFFFAOYSA-N
60	7-HDAHPA	595.6→280.3	599.6→280.3	2755	527.4805	$C_{33}H_{64}O_4$	CCCCCCCCCCCCCCCCC(=O)OC(CCCCCCCCC)CCCCCC(=O)O	KMKQSHNGRVTFGM-UHFFFAOYSA-N
61	9-HDAHPA	595.6→280.3	599.6→280.3	2680	528.4805	$C_{33}H_{64}O_4$	CCCCCCCCCCCCCCCCC(=O)OC(CCCCCCC)CCCCCCCC(=O)O	JGKJSQPPGBDHGE-UHFFFAOYSA-N
62	3-SAHPA	609.6→280.3	613.6→280.3	2956	538.4961	$C_{34}H_{66}O_4$	CCCCCCCCCCCCCCCCCC(=O)OC(CCCCCCCCCCCCC)CC(=O)O	RFKVRHXDDSMVRS-UHFFFAOYSA-N
63	4-SAHPA	609.6→280.3	613.6→280.3	2912	538.4961	$C_{34}H_{66}O_4$	CCCCCCCCCCCCCCCCCC(=O)OC(CCCCCCCCCCCC)CCC(=O)O	FZRVRJVYSQXDPU-UHFFFAOYSA-N
64	5-SAHPA	609.6→280.3	613.6→280.3	2894	538.4961	$C_{34}H_{66}O_4$	CCCCCCCCCCCCCCCCCC(=O)OC(CCCCCCCCCCC)CCCC(=O)O	JWJCTGOXQUWCLD-UHFFFAOYSA-N
65	6-SAHPA	609.6→280.3	613.6→280.3	2862	538.4961	$C_{34}H_{66}O_4$	CCCCCCCCCCCCCCCCCC(=O)OC(CCCCCCCCCC)CCCCC(=O)O	QXFZPZGDDJSTQM-UHFFFAOYSA-N
66	7-SAHPA	609.6→280.3	613.6→280.3	2830	538.4961	$C_{34}H_{66}O_4$	CCCCCCCCCCCCCCCCCC(=O)OC(CCCCCCCCC)CCCCCC(=O)O	AWINNZJYDSFBEW-UHFFFAOYSA-N
67	8-SAHPA	609.6→280.3	613.6→280.3	2801	538.4961	$C_{34}H_{66}O_4$	CCCCCCCCCCCCCCCCCC(=O)OC(CCCCCCCC)CCCCCCC(=O)O	RKGTXTPCRDJTLA-UHFFFAOYSA-N
68	9-SAHPA	609.6→280.3	613.6→280.3	2780	538.4961	$C_{34}H_{66}O_4$	CCCCCCCCCCCCCCCCCC(=O)OC(CCCCCCC)CCCCCCCC(=O)O	WHLXDKLTBPWEPM-UHFFFAOYSA-N
69	10-SAHPA	609.6→280.3	613.6→280.3	2752	538.4961	$C_{34}H_{66}O_4$	CCCCCCCCCCCCCCCCCC(=O)OC(CCCCCC)CCCCCCCCC(=O)O	GIDPMACVNDUQAL-UHFFFAOYSA-N
70	11-SAHPA	609.6→280.3	613.6→280.3	2716	538.4961	$C_{34}H_{66}O_4$	CCCCCCCCCCCCCCCCCC(=O)OC(CCCCC)CCCCCCCCCC(=O)O	WUOZOGTVMJXVDI-UHFFFAOYSA-N
71	12-SAHPA	609.6→280.3	613.6→280.3	2695	538.4961	$C_{34}H_{66}O_4$	CCCCCCCCCCCCCCCCCC(=O)OC(CCCC)CCCCCCCCCCC(=O)O	PAPAJXFPYIPNIP-UHFFFAOYSA-N
72	13-SAHPA	609.6→280.3	613.6→280.3	2680	538.4961	$C_{34}H_{66}O_4$	CCCCCCCCCCCCCCCCCC(=O)OC(CCC)CCCCCCCCCCCC(=O)O	IRYKDNPAJYJRSS-UHFFFAOYSA-N
73	13-AAHPA	637.6→280.3	641.6→280.3	2859	566.5274	$C_{36}H_{70}O_4$	CCCCCCCCCCCCCCCCCCCC(=O)OC(CCC)CCCCCCCCCCCC(=O)O	WGDWDXIGJPKLIL-UHFFFAOYSA-N
74	9-MAHSA	581.8→308.5	585.8→308.5	2614	510.4648	$C_{32}H_{62}O_4$	CCCCCCCCCCCCCC(=O)OC(CCCCCCCCC)CCCCCCCC(=O)O	BTPQSCPUUJROHO-UHFFFAOYSA-N
75	10-MAHSA	581.8→308.5	585.8→308.5	2580	510.4648	$C_{32}H_{62}O_4$	CCCCCCCCCCCCCC(=O)OC(CCCCCCCC)CCCCCCCCC(=O)O	FIPSYEUUZDUVPJ-UHFFFAOYSA-N
76	12-MAHSA	581.8→308.5	585.8→308.5	2523	510.4648	$C_{32}H_{62}O_4$	CCCCCCCCCCCCCC(=O)OC(CCCCCC)CCCCCCCCCCC(=O)O	ZWLRESZXRFHFKD-UHFFFAOYSA-N
77	13-MAHSA	581.8→308.5	585.8→308.5	2486	510.4648	$C_{32}H_{62}O_4$	CCCCCCCCCCCCCC(=O)OC(CCCCC)CCCCCCCCCCCC(=O)O	DLVHSZMTBMIIAW-UHFFFAOYSA-N
78	4-PDAHSA	595.8→308.5	599.8→308.5	2850	524.4805	$C_{33}H_{64}O_4$	CCCCCCCCCCCCCCC(CCC(=O)O)OC(=O)CCCCCCCCCCCCCC	JWIAXTLGSTWWFU-UHFFFAOYSA-N
79	5-PDAHSA	595.8→308.5	599.8→308.5	2825	524.4805	$C_{33}H_{64}O_4$	CCCCCCCCCCCCCCC(=O)OC(CCCCCCCCCCCCC)CCCC(=O)O	TYRNUMHGCISNJL-UHFFFAOYSA-N
80	6-PDAHSA	595.8→308.5	599.8→308.5	2787	524.4805	$C_{33}H_{64}O_4$	CCCCCCCCCCCCCCC(=O)OC(CCCCCCCCCCCC)CCCCC(=O)O	KCZZCXCNRRZHRS-UHFFFAOYSA-N
81	7-PDAHSA	595.8→308.5	599.8→308.5	2759	524.4805	$C_{33}H_{64}O_4$	CCCCCCCCCCCCCCC(=O)OC(CCCCCCCCCCC)CCCCCC(=O)O	QOPUDLPZRNXTID-UHFFFAOYSA-N
82	8-PDAHSA	595.8→308.5	599.8→308.5	2725	524.4805	$C_{33}H_{64}O_4$	CCCCCCCCCCCCCCC(=O)OC(CCCCCCCCCC)CCCCCCC(=O)O	COANUTXJQWPRCR-UHFFFAOYSA-N
83	9-PDAHSA	595.8→308.5	599.8→308.5	2699	524.4805	$C_{33}H_{64}O_4$	CCCCCCCCCCCCCCC(=O)OC(CCCCCCCCC)CCCCCCCC(=O)O	YLIVLRPBCWUSPW-UHFFFAOYSA-N
84	10-PDAHSA	595.8→308.5	599.8→308.5	2678	524.4805	$C_{33}H_{64}O_4$	CCCCCCCCCCCCCCC(=O)OC(CCCCCCCC)CCCCCCCCC(=O)O	JRFQEXOQVRFITD-UHFFFAOYSA-N
85	11-PDAHSA	595.8→308.5	599.8→308.5	2642	524.4805	$C_{33}H_{64}O_4$	CCCCCCCCCCCCCCC(=O)OC(CCCCCCC)CCCCCCCCCC(=O)O	JZUWODWTUQAESF-UHFFFAOYSA-N
86	12-PDAHSA	595.8→308.5	599.8→308.5	2617	524.4805	$C_{33}H_{64}O_4$	CCCCCCCCCCCCCCC(=O)OC(CCCCCC)CCCCCCCCCCC(=O)O	XSUVALZHWBJLBX-UHFFFAOYSA-N
87	13-PDAHSA	595.8→308.5	599.8→308.5	2577	524.4805	$C_{33}H_{64}O_4$	CCCCCCCCCCCCCCC(=O)OC(CCCCC)CCCCCCCCCCCC(=O)O	SMOPASQJMCZISP-UHFFFAOYSA-N
88	14-PDAHSA	595.8→308.5	599.8→308.5	2545	524.4805	$C_{33}H_{64}O_4$	CCCCCCCCCCCCCCC(=O)OC(CCCC)CCCCCCCCCCCCC(=O)O	SVNUYGFVNYWLAX-UHFFFAOYSA-N
89	15-PDAHSA	595.8→308.5	599.8→308.5	2522	524.4805	$C_{33}H_{64}O_4$	CCCCCCCCCCCCCCC(=O)OC(CCC)CCCCCCCCCCCCCC(=O)O	NSZWTMZAEPGEBN-UHFFFAOYSA-N
90	3-PAHSA	609.8→308.5	613.8→308.5	2948	538.4961	$C_{34}H_{66}O_4$	CCCCCCCCCCCCCCCC(CC(=O)O)OC(=O)CCCCCCCCCCCCCCC	UKZPVOXSOKDEQO-UHFFFAOYSA-N
91	4-PAHSA	609.8→308.5	613.8→308.5	2915	538.4961	$C_{34}H_{66}O_4$	CCCCCCCCCCCCCCCC(=O)OC(CCCCCCCCCCCCCC)CCC(=O)O	AEEUJWAGGKKECZ-UHFFFAOYSA-N
92	5-PAHSA	609.8→308.5	613.8→308.5	2897	538.4961	$C_{34}H_{66}O_4$	CCCCCCCCCCCCCCCC(=O)OC(CCCCCCCCCCCCC)CCCC(=O)O	QBGKCWKQYJQHJX-UHFFFAOYSA-N
93	6-PAHSA	609.8→308.5	613.8→308.5	2869	538.4961	$C_{34}H_{66}O_4$	CCCCCCCCCCCCCCCC(=O)OC(CCCCCCCCCCCC)CCCCC(=O)O	MAPFRVBMNBJJET-UHFFFAOYSA-N
94	7-PAHSA	609.8→308.5	613.8→308.5	2836	538.4961	$C_{34}H_{66}O_4$	CCCCCCCCCCCCCCCC(=O)OC(CCCCCCCCCCC)CCCCCC(=O)O	USNXMSKYBRAGPQ-UHFFFAOYSA-N
95	8-PAHSA	609.8→308.5	613.8→308.5	2810	538.4961	$C_{34}H_{66}O_4$	CCCCCCCCCCCCCCCC(=O)OC(CCCCCCCCCC)CCCCCCC(=O)O	PXZVHHRFDCMNSG-UHFFFAOYSA-N
96	9-PAHSA	609.8→308.5	613.8→308.5	2779	538.4961	$C_{34}H_{66}O_4$	CCCCCCCCCCCCCCCC(=O)OC(CCCCCCCCC)CCCCCCCC(=O)O	MHQWHZLXDBVXML-UHFFFAOYSA-N
97	10-PAHSA	609.8→308.5	613.8→308.5	2759	538.4961	$C_{34}H_{66}O_4$	CCCCCCCCCCCCCCCC(=O)OC(CCCCCCCC)CCCCCCCCC(=O)O	UVHRDGWHIDFWID-UHFFFAOYSA-N
98	11-PAHSA	609.8→308.5	613.8→308.5	2736	538.4961	$C_{34}H_{66}O_4$	CCCCCCCCCCCCCCCC(=O)OC(CCCCCCC)CCCCCCCCCC(=O)O	SMPUVWFQRGEBKE-UHFFFAOYSA-N
99	12-PAHSA	609.8→308.5	613.8→308.5	2705	538.4961	$C_{34}H_{66}O_4$	CCCCCCCCCCCCCCCC(=O)OC(CCCCCC)CCCCCCCCCCC(=O)O	XXHBLSWAKHZVLN-UHFFFAOYSA-N
100	13-PAHSA	609.8→308.5	613.8→308.5	2675	538.4961	$C_{34}H_{66}O_4$	CCCCCCCCCCCCCCCC(=O)OC(CCCCC)CCCCCCCCCCCC(=O)O	XCOROKUALFOQRK-UHFFFAOYSA-N
101	14-PAHSA	609.8→308.5	613.8→308.5	2655	538.4961	$C_{34}H_{66}O_4$	CCCCCCCCCCCCCCCC(=O)OC(CCCC)CCCCCCCCCCCCC(=O)O	GZRKGWRALYMBHF-UHFFFAOYSA-N
102	5-HDAHSA	623.7→308.5	627.8→308.5	2971	552.5118	$C_{35}H_{68}O_4$	CCCCCCCCCCCCCCCCC(=O)OC(CCCCCCCCCCCCC)CCCC(=O)O	UHLIJFPLQZAGKS-UHFFFAOYSA-N
103	7-HDAHSA	623.7→308.5	627.8→308.5	2918	552.5118	$C_{35}H_{68}O_4$	CCCCCCCCCCCCCCCCC(=O)OC(CCCCCCCCCCC)CCCCCC(=O)O	IFIYDYDBSNSPQB-UHFFFAOYSA-N
104	8-HDAHSA	623.7→308.5	627.8→308.5	2886	552.5118	$C_{35}H_{68}O_4$	CCCCCCCCCCCCCCCCC(=O)OC(CCCCCCCCCC)CCCCCCC(=O)O	KZIUMUBUCNCBMY-UHFFFAOYSA-N
105	9-HDAHSA	623.7→308.5	627.8→308.5	2869	552.5118	$C_{35}H_{68}O_4$	CCCCCCCCCCCCCCCCC(=O)OC(CCCCCCCCC)CCCCCCCC(=O)O	IMSVZRAIPHPOKC-UHFFFAOYSA-N
106	4-SAHSA	637.8→308.5	641.8→308.5	3075	566.5274	$C_{36}H_{70}O_4$	CCCCCCCCCCCCCCCCCC(=O)OC(CCCCCCCCCCCCCC)CCC(=O)O	WKXMFXABPFNGEX-UHFFFAOYSA-N
107	5-SAHSA	637.8→308.5	641.8→308.5	3050	566.5274	$C_{36}H_{70}O_4$	CCCCCCCCCCCCCCCCCC(=O)OC(CCCCCCCCCCCCC)CCCC(=O)O	BSOQNRNPFJEDMC-UHFFFAOYSA-N
108	6-SAHSA	637.8→308.5	641.8→308.5	3025	566.5274	$C_{36}H_{70}O_4$	CCCCCCCCCCCCCCCCCC(=O)OC(CCCCCCCCCCCC)CCCCC(=O)O	FBBUCTRNJXSYIE-UHFFFAOYSA-N
109	7-SAHSA	637.8→308.5	641.8→308.5	2995	566.5274	$C_{36}H_{70}O_4$	CCCCCCCCCCCCCCCCCC(=O)OC(CCCCCCCCCCC)CCCCCC(=O)O	QUZMPBWTOKQCEK-UHFFFAOYSA-N
110	8-SAHSA	637.8→308.5	641.8→308.5	2970	566.5274	$C_{36}H_{70}O_4$	CCCCCCCCCCCCCCCCCC(=O)OC(CCCCCCCCCC)CCCCCCC(=O)O	MFSOFHGBSOJSCQ-UHFFFAOYSA-N
111	9-SAHSA	637.8→308.5	641.8→308.5	2949	566.5274	$C_{36}H_{70}O_4$	CCCCCCCCCCCCCCCCCC(=O)OC(CCCCCCCCC)CCCCCCCC(=O)O	NQJLCZWOOVLQNP-UHFFFAOYSA-N

112	10-SAHSA	637.8→308.5	641.8→308.5	2924	566.5274	C36H70O4	CCCCCCCCCCCCCCCCCC(=O)OC(CCCCCCCC)CCCCCCCCC(=O)O	KARVIOOFYPUCOA-UHFFFAOYSA-N
113	11-SAHSA	637.8→308.5	641.8→308.5	2890	566.5274	C36H70O4	CCCCCCCCCCCCCCCCCC(=O)OC(CCCCCCC)CCCCCCCCCC(=O)O	NYMRYXUNFHXRMB-UHFFFAOYSA-N
114	12-SAHSA	637.8→308.5	641.8→308.5	2872	566.5274	C36H70O4	CCCCCCCCCCCCCCCCCC(=O)OC(CCCCCC)CCCCCCCCCCC(=O)O	HCUIHIKPUYHKSQ-UHFFFAOYSA-N
115	13-SAHSA	637.8→308.5	641.8→308.5	2858	566.5274	C36H70O4	CCCCCCCCCCCCCCCCCC(=O)OC(CCCCC)CCCCCCCCCCCC(=O)O	OCKHONDLGBRMHC-UHFFFAOYSA-N
116	14-SAHSA	637.8→308.5	641.8→308.5	2824	566.5274	C36H70O4	CCCCCCCCCCCCCCCCCC(=O)OC(CCCC)CCCCCCCCCCCCC(=O)O	XRSWGRKOVBYNOR-UHFFFAOYSA-N
117	3-POHSA	607.8→308.5	611.8→308.5	2770	534.4805	C34H64O4	CCCCCCCCCCCCCCCC(CC(=O)O)OC(=O)CCCCCCC/C=C\CCCCCC	GUPSYUQZAZQZLV-PEZBUJJGSA-N
118	5-POHSA	607.8→308.5	611.8→308.5	2730	534.4805	C34H64O4	CCCCCCCCCCCCCC(CCCC(=O)O)OC(=O)CCCCCCC/C=C\CCCCCC	HADGPAWFBKHJNM-SQFISAMPSA-N
119	9-POHSA	607.8→308.5	611.8→308.5	2609	534.4805	C34H64O4	CCCCCCCCCC(CCCCCCCC(=O)O)OC(=O)CCCCCCC/C=C\CCCCCC	VCXRHEIVUHPWLL-SEYXRHQNSA-N
120	10-POHSA	607.8→308.5	611.8→308.5	2578	534.4805	C34H64O4	CCCCCCCCC(CCCCCCCCC(=O)O)OC(=O)CCCCCCC/C=C\CCCCCC	FUMHQIYYIKZIBE-SEYXRHQNSA-N
121	11-POHSA	607.8→308.5	611.8→308.5	2559	534.4805	C34H64O4	CCCCCCCC(CCCCCCCCCC(=O)O)OC(=O)CCCCCCC/C=C\CCCCCC	UHMYYORZPBAFTF-QXMHVHEDSA-N
122	12-POHSA	607.8→308.5	611.8→308.5	2535	534.4805	C34H64O4	CCCCCC/C=C\CCCCCCCC(=O)OC(CCCCCC)CCCCCCCCCCC(=O)O	XSYATLPMKFNWBI-QXMHVHEDSA-N
123	13-POHSA	607.8→308.5	611.8→308.5	2513	534.4805	C34H64O4	CCCCCC/C=C\CCCCCCCC(=O)OC(CCCCC)CCCCCCCCCCCC(=O)O	FHXCZZFHUCAQAA-KHPPLWFESA-N
124	3-OAHSA	635.8→308.5	639.8→308.5	2940	564.5118	C36H68O4	CCCCCCCCCCCCCCCC(CC(=O)O)OC(=O)CCCCCCC/C=C\CCCCCCCC	ZUPUISWRBLIQFZ-ZCXUNETKSA-N
125	4-OAHSA	635.8→308.5	639.8→308.5	2913	564.5118	C36H68O4	CCCCCCCCCCCCCCC(CCC(=O)O)OC(=O)CCCCCCC/C=C\CCCCCCCC	KSSRLILKAPQMSE-ZCXUNETKSA-N
126	5-OAHSA	635.8→308.5	639.8→308.5	2894	564.5118	C36H68O4	CCCCCCCCCCCCCC(CCCC(=O)O)OC(=O)CCCCCCC/C=C\CCCCCCCC	FQZBGGYKEFIGPO-MSUUIHNZSA-N
127	8-OAHSA	635.8→308.5	639.8→308.5	2811	564.5118	C36H68O4	CCCCCCCCCCC(CCCCCCC(=O)O)OC(=O)CCCCCCC/C=C\CCCCCCCC	YVQCSUUWPYOYGC-NXVVXOECSA-N
128	9-OAHSA	635.8→308.5	639.8→308.5	2775	564.5118	C36H68O4	CCCCCCCCCC(CCCCCCCC(=O)O)OC(=O)CCCCCCC/C=C\CCCCCCCC	PGKKGBQMNNEIHV-PFONDFGASA-N
129	10-OAHSA	635.8→308.5	639.8→308.5	2745	564.5118	C36H68O4	CCCCCCCC/C=C\CCCCCCCC(=O)OC(CCCCCCCC)CCCCCCCCC(=O)O	WYKCNLTXWMQKGB-PFONDFGASA-N
130	12-OAHSA	635.8→308.5	639.8→308.5	2705	564.5118	C36H68O4	CCCCCCCC/C=C\CCCCCCCC(=O)OC(CCCCCC)CCCCCCCCCCC(=O)O	OCHJVQODRYVDAA-YPKPFQOOSA-N
131	13-OAHSA	635.8→308.5	639.8→308.5	2684	564.5118	C36H68O4	CCCCCCCC/C=C\CCCCCCCC(=O)OC(CCCCC)CCCCCCCCCCCC(=O)O	RCTXOTTXFKSGGU-SEYXRHQNSA-N
132	14-OAHSA	635.8→308.5	639.8→308.5	2659	564.5118	C36H68O4	CCCCCCCC/C=C\CCCCCCCC(=O)OC(CCCC)CCCCCCCCCCCCC(=O)O	PWUDMIAEMYRYBU-SEYXRHQNSA-N
