# Supplementary material for: A dataset of branched fatty acid esters of hydroxy fatty acids diversity in foods
Source: Sci Data. 2023 Nov 10;10:790. doi: 10.1038/s41597-023-02712-z (PMC10638281; doi:10.1038/s41597-023-02712-z)
Supplement: Supplementary file 8 — Supplementary information-1 Table S1. FAHFA standards list [file 41597_2023_2712_MOESM8_ESM.pdf]

Supplementary Table S1. FAHFA standards list

Family	Formula	Monoisotopic Mass	Regioisomer	SMILES	InChIKey	Structure
PAHSA	C34H66O4	538.4961	13-PAHSA	CCCCCCCCCCCCCCCC(=O)OC(CCCCC)CCCCCCCCCCCCC(=O)O	XCOROKUALFOQRK-UHFFFAOYSA-N	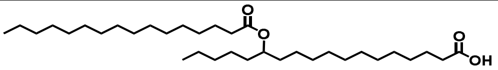
			12-PAHSA	CCCCCCCCCCCCCCCC(=O)OC(CCCCCC)CCCCCCCCCCC(=O)O	XXHBLSWAKHZVLN-UHFFFAOYSA-N	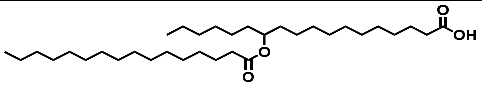
			10-PAHSA	CCCCCCCCCCCCCCCC(=O)OC(CCCCCCCC)CCCCCCCCC(=O)O	UVHRDGWHIDFWID-UHFFFAOYSA-N	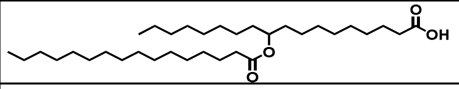
			9-PAHSA	CCCCCCCCCCCCCCCC(=O)OC(CCCCCCCCC)CCCCCCCC(=O)O	MHQWHZLXDBVXML-UHFFFAOYSA-N	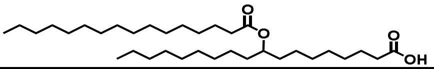
			5-PAHSA	CCCCCCCCCCCCCCCC(=O)OC(CCCCCCCCCCCCC)CCCC(=O)O	QBGKCWKQYJQHJX-UHFFFAOYSA-N	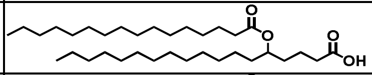
			3-PAHSA	CCCCCCCCCCCCCCCC(CC(=O)O)OC(=O)CCCCCCCCCCCCCCC	UKZPVOXSOKDEQO-UHFFFAOYSA-N	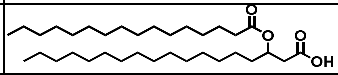
POHSA	C34H64O4	536.4805	13-POHSA	CCCCCC/C=C\CCCCCCCC(=O)OC(CCCCC)CCCCCCCCCCCC(=O)O	FHXCZZFHUCAQAA-KHPPLWFESA-N	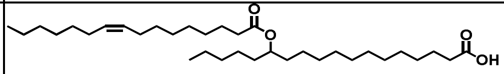
			12-POHSA	CCCCCC/C=C\CCCCCCCC(=O)OC(CCCCCC)CCCCCCCCCCC(=O)O	XSYATLPMKFNWBI-QXMHVHEDSA-N	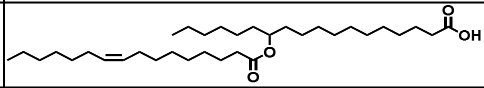
			10-POHSA	CCCCCCCCC(CCCCCCCCC(=O)O)OC(=O)CCCCCCC/C=C\CCCCCC	FUMHQIYYIKZIBE-SEYXRHQNSA-N	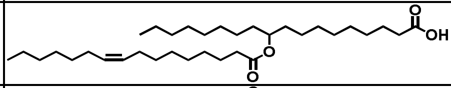
			9-POHSA	CCCCCCCCCC(CCCCCCCC(=O)O)OC(=O)CCCCCCC/C=C\CCCCCC	VCXRHEIVUHPWLL-SEYXRHQNSA-N	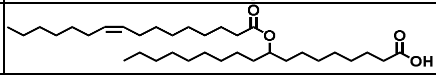
			5-POHSA	CCCCCCCCCCCCCC(CCCC(=O)O)OC(=O)CCCCCCC/C=C\CCCCCC	HADGPAWFBKHJNM-SQFISAMPSA-N	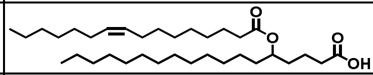
SAHSA	C36H70O4	566.5274	13-SAHSA	CCCCCCCCCCCCCCCCCC(=O)OC(CCCCC)CCCCCCCCCCCC(=O)O	OCKHONDLGBRMHC-UHFFFAOYSA-N	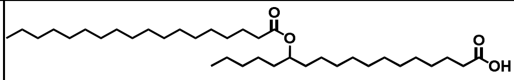
			12-SAHSA	CCCCCCCCCCCCCCCCCC(=O)OC(CCCCCC)CCCCCCCCCCC(=O)O	HCUIHIKPUYHKSQ-UHFFFAOYSA-N	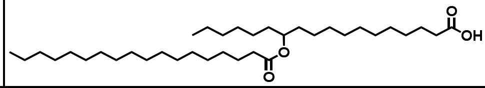
			10-SAHSA	CCCCCCCCCCCCCCCCCC(=O)OC(CCCCCCCC)CCCCCCCCC(=O)O	KARVIOOFYPUCOA-UHFFFAOYSA-N	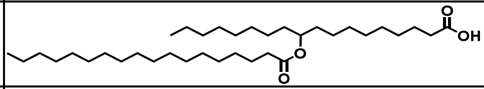
			9-SAHSA	CCCCCCCCCCCCCCCCCC(=O)OC(CCCCCCCCC)CCCCCCCC(=O)O	NQJLCZWOOVLQNP-UHFFFAOYSA-N	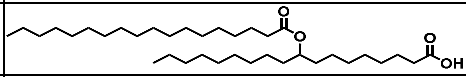
			5-SAHSA	CCCCCCCCCCCCCCCCCC(=O)OC(CCCCCCCCCCCCC)CCCC(=O)O	BSOQNRNPFJEDMC-UHFFFAOYSA-N	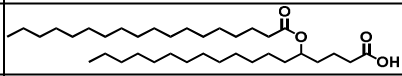
OAHSA	C36H68O4	564.5118	13-OAHSA	CCCCCCCC/C=C\CCCCCCCC(=O)OC(CCCCC)CCCCCCCCCCCC(=O)O	RCTXOTTXFKSGGU-SEYXRHQNSA-N	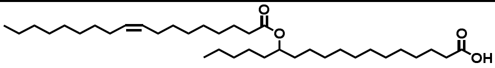
			12-OAHSA	CCCCCCCC/C=C\CCCCCCCC(=O)OC(CCCCCC)CCCCCCCCCCC(=O)O	OCHJVQODRYVDAA-YPKPFQOOSA-N	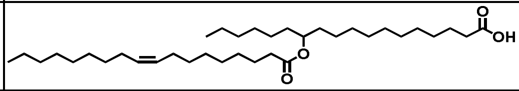
			10-OAHSA	CCCCCCCC/C=C\CCCCCCCC(=O)OC(CCCCCCCC)CCCCCCCCC(=O)O	WYKCNLTXWMQKGB-PFONDFGASA-N	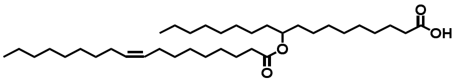
			9-OAHSA	CCCCCCCCCC(CCCCCCCC(=O)O)OC(=O)CCCCCCC/C=C\CCCCCCCC	PGKKGBQMNNEIHV-PFONDFGASA-N	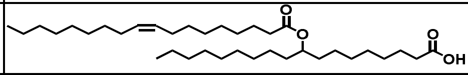
			5-OAHSA	CCCCCCCCCCCCCC(CCCC(=O)O)OC(=O)CCCCCCC/C=C\CCCCCCCC	FQZBGGYKEFIGPO-MSUUIHNZSA-N	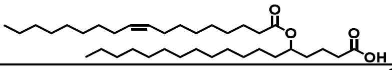
HDAHSA	C35H68O4	552.5118	10-HDAHSA	CCCCCCCCCCCCCCCCC(=O)OC(CCCCCCCC)CCCCCCCCC(=O)O	DNGRCMHKDHYUBW-UHFFFAOYSA-N	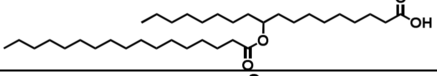
			9-HDAHSA	CCCCCCCCCCCCCCCCC(=O)OC(CCCCCCCCC)CCCCCCCC(=O)O	IMSVZRAIPHPOKC-UHFFFAOYSA-N	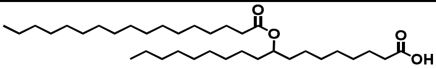
			7-HDAHSA	CCCCCCCCCCCCCCCCC(=O)OC(CCCCCCCCCCC)CCCCCC(=O)O	IFIYDYDBSNSPQB-UHFFFAOYSA-N	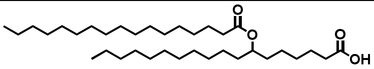

PDAHSA	C33H64O4	524.4805	10-PDAHSA	CCCCCCCCCCCCCCC(=O)OC(CCCCCCCC)CCCCCCCCC(=O)O	JRFQEXOQVRFITD-UHFFFAOYSA-N	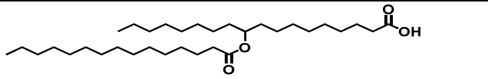
			9-PDAHSA	CCCCCCCCCCCCCCC(=O)OC(CCCCCCCCC)CCCCCCCC(=O)O	YLIVLRPBCWUSPW-UHFFFAOYSA-N	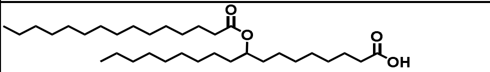
			7-PDAHSA	CCCCCCCCCCCCCCC(=O)OC(CCCCCCCCCCC)CCCCCC(=O)O	QOPUDLPZRNXTID-UHFFFAOYSA-N	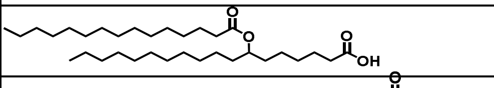
PDAHPA	C31H60O4	496.4492	10-PDAHPA	CCCCCCCCCCCCCCC(=O)OC(CCCCCC)CCCCCCCCC(=O)O	CNZXEKAPQAJBTK-UHFFFAOYSA-N	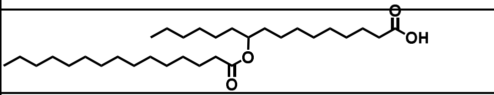
			9-PDAHPA	CCCCCCCCCCCCCCC(=O)OC(CCCCCCC)CCCCCCCC(=O)O	KJHFLWNKUPFGKA-UHFFFAOYSA-N	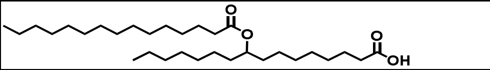
			5-PDAHPA	CCCCCCCCCCCCCCC(=O)OC(CCCCCCCCCCC)CCCC(=O)O	IOOQLHSIAOWEQT-UHFFFAOYSA-N	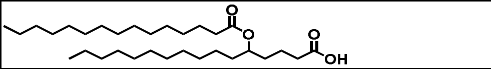
MAHMA	C28H54O4	454.4022	10-MAHMA	CCCCCCCCCCCCCC(=O)OC(CCCC)CCCCCCCCC(=O)O	PEOJOXYCMSXYRI-UHFFFAOYSA-N	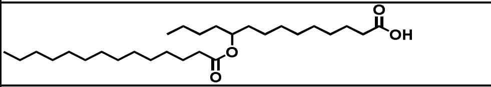
			9-MAHMA	CCCCCCCCCCCCCC(=O)OC(CCCCC)CCCCCCCC(=O)O	IHKNEWAXALARRP-UHFFFAOYSA-N	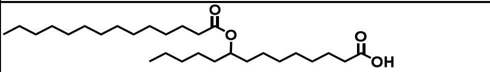
			5-MAHMA	CCCCCCCCCCCCCC(=O)OC(CCCCCCCCC)CCCC(=O)O	ODCLJRIUYLLLRH-UHFFFAOYSA-N	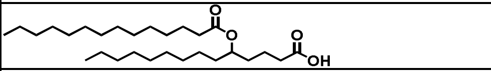
SAHPA	C34H66O4	538.4961	10-SAHPA	CCCCCCCCCCCCCCCCCC(=O)OC(CCCCCC)CCCCCCCCC(=O)O	GIDPMACVNDUQAL-UHFFFAOYSA-N	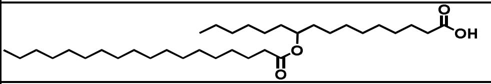
			9-SAHPA	CCCCCCCCCCCCCCCCCC(=O)OC(CCCCCCC)CCCCCCCC(=O)O	WHLXDKLTBPWEPM-UHFFFAOYSA-N	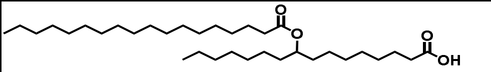
			5-SAHPA	CCCCCCCCCCCCCCCCCC(=O)OC(CCCCCCCCCCC)CCCC(=O)O	JWJCTGOXQUWCLD-UHFFFAOYSA-N	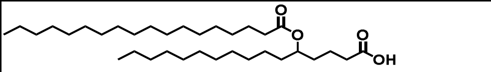
MAHPA	C30H58O4	482.4335	10-MAHPA	CCCCCCCCCCCCCC(=O)OC(CCCCCC)CCCCCCCCC(=O)O	LOJDMJNXHRPEJR-UHFFFAOYSA-N	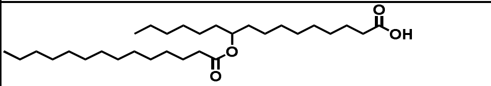
			9-MAHPA	CCCCCCCCCCCCCC(=O)OC(CCCCCCC)CCCCCCCC(=O)O	SNRHTFPCAKXVHV-UHFFFAOYSA-N	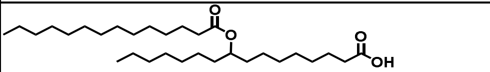
			5-MAHPA	CCCCCCCCCCCCCC(=O)OC(CCCCCCCCCCC)CCCC(=O)O	RDMQIABVBPGPQO-UHFFFAOYSA-N	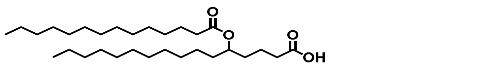
PAHPA	C32H62O4	510.4648	10-PAHPA	CCCCCCCCCCCCCCCC(=O)OC(CCCCCC)CCCCCCCCC(=O)O	WWOQHWCTUBCRSA-UHFFFAOYSA-N	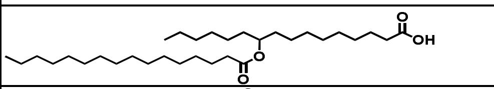
			9-PAHPA	CCCCCCCCCCCCCCCC(=O)OC(CCCCCCC)CCCCCCCC(=O)O	DPFVDUJFYPYAOO-UHFFFAOYSA-N	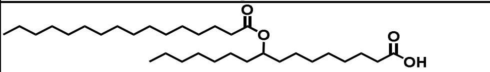
			5-PAHPA	CCCCCCCCCCCCCCCC(=O)OC(CCCCCCCCCCC)CCCC(=O)O	KBWQFRVOCVTZTO-UHFFFAOYSA-N	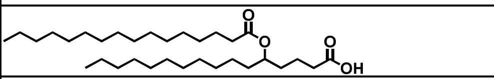
			3-PAHPA	CCCCCCCCCCCCCCCC(=O)OC(CCCCCCCCCCCCC)CC(=O)O	QXMZBFFWEBPYSW-UHFFFAOYSA-N	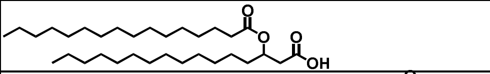
PAHMA	C30H58O4	482.4335	10-PAHMA	CCCCCCCCCCCCCCCC(=O)OC(CCCC)CCCCCCCCC(=O)O	DYQFPWPFIGGWSX-UHFFFAOYSA-N	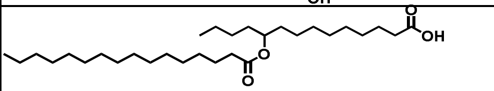
			9-PAHMA	CCCCCCCCCCCCCCCC(=O)OC(CCCCC)CCCCCCCC(=O)O	ADZIVQSYWSLHHX-UHFFFAOYSA-N	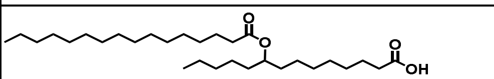
			5-PAHMA	CCCCCCCCCCCCCCCC(=O)OC(CCCCCCCCC)CCCC(=O)O	GZSCEQSBGJUGAA-UHFFFAOYSA-N	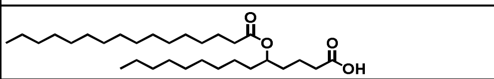
SAHMA	C32H62O4	510.4648	10-SAHMA	CCCCCCCCCCCCCCCCCC(=O)OC(CCCC)CCCCCCCCC(=O)O	YTSABFXBGKBOPQ-UHFFFAOYSA-N	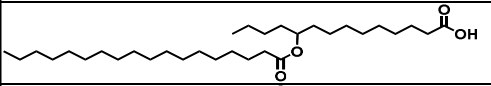
			9-SAHMA	CCCCCCCCCCCCCCCCCC(=O)OC(CCCCC)CCCCCCCC(=O)O	QOKJPCRPQUZMKK-UHFFFAOYSA-N	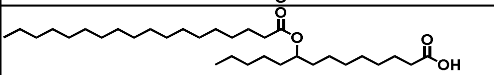
			5-SAHMA	CCCCCCCCCCCCCCCCCC(=O)OC(CCCCCCCCC)CCCC(=O)O	ATXZYOLUWOGARZ-UHFFFAOYSA-N	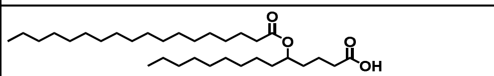
MAHSA	C32H62O4	510.4648	9-MAHSA	CCCCCCCCCCCCCC(=O)OC(CCCCCCCCC)CCCCCCCC(=O)O	BTPQSCPUUJROHO-UHFFFAOYSA-N	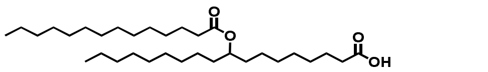
